# Reverse engineering of BNIP3 identifies a mitochondrial protective peptide

**DOI:** 10.1038/s41467-026-73993-2

**Published:** 2026-06-17

**Authors:** Ulrike B. Hendgen-Cotta, Anna Roth, Christine Beuck, Daniel Messiha, Stephan Settelmeier, Shah Bahrullah Shah, Sebastian Korste, Kenny Bravo-Rodriguez, Mike Blueggel, Feyza Cansiz, Luiza Martins Nascentes Melo, Jonas Roesler, Sven W. Meckelmann, Oliver J. Schmitz, Farnusch Kaschani, Markus Kaiser, Sonja Esfeld, Omar El Bounkari, Jürgen Bernhagen, Sophie Brameyer, Kirsten Jung, Linda-Isabell Schmitt, Markus Leo, Tim Hagenacker, Matthias Totzeck, Thomas Minor, Michael Ehrmann, Alpaslan Tasdogan, Peter Bayer, Tienush Rassaf

**Affiliations:** 1 Department of Cardiology and Vascular Medicine, West German Heart and Vascular Center, University Hospital Essen, University of Duisburg-Essen, Essen, Germany; 2 Center of Medical Biotechnology, Research Group Structural and Medicinal Biochemistry, Faculty of Biology, University of Duisburg-Essen, Essen, Germany; 3 Department of Mechanistic Cell Biology, Max Planck Institute of Molecular Physiology, Dortmund, Germany; 4 Department of Dermatology, Medical Faculty, University of Duisburg-Essen & German Cancer Consortium (DKTK), Essen, Germany; 5 Applied Analytical Chemistry, University of Duisburg-Essen, Essen, Germany; 6 Center of Medical Biotechnology, Analytics Core Facility Essen, Faculty of Biology, University of Duisburg-Essen, Essen, Germany; 7 Center of Medical Biotechnology, Chemical Biology, Faculty of Biology, University of Duisburg-Essen, Essen, Germany; 8 Division of Vascular Biology, Institute for Stroke and Dementia Research (ISD), LMU Klinikum, Ludwig-Maximilians-University (LMU) Munich, Munich, Munich, Germany; 9 German Center for Cardiovascular Research (DZHK), Partner Site Munich Heart Alliance, Munich, Germany; 10 Munich Cluster for Systems Neurology (SyNergy), Munich, Germany; 11 Faculty of Biology, Microbiology, Ludwig-Maximilians-University (LMU) Munich, Martinsried, Germany; 12 Department auf Neurology and Center for Translational Neuro and Behavioral Science, Medical Faculty, University Hospital Essen, Essen, Germany; 13 Surgical Research Department, Medical Faculty, University Hospital Essen, Essen, Germany; 14 Center of Medical Biotechnology, Department of Microbiology, Faculty of Biology, University of Duisburg-Essen, Essen, Germany

**Keywords:** Apoptosis, Drug development, Mitochondria

## Abstract

Recent advances in mitochondrial network dynamic and signalling highlight mitochondria as key therapeutic targets across diverse diseases. Yet, high drug development failure rates reflect an incomplete understanding of upstream molecular regulators of mitochondrial fate. Here, we address this gap by reverse engineering of the BH3-only protein BNIP3. Structural modelling and sequence–function analyses of its N-terminus identify a critical functional domain and amino acid hotspots that directly activate BCL-2 executioner proteins, triggering mitochondrial cell death. Leveraging these insights, we develop a BNIP3 antagonist peptide (B-017) that disrupts interactions between BNIP3 and BCL-2 executioner proteins, preserving mitochondrial integrity. B-017 demonstrates target specificity, a favourable safety profile, and robust suppression of cell death signalling in human cells. In clinically relevant animal models, it reduces tissue damage in the heart, brain, and liver. Together, these findings position B-017 as a promising therapeutic candidate targeting mitochondrial dysfunction.

## Abstract (web summary)

Upstream regulators of mitochondrial cell death remain poorly defined yet represent promising therapeutic targets. Here, the authors identify the BNIP3 activation domain responsible for engaging BCL-2 executioner proteins and develop an antagonist peptide that suppresses cell death signalling, thereby reducing cardiac, cerebral, and hepatic injury

## Introduction

A significant number of diseases are associated with an excessive activation of programmed cell death processes^1,2^. Despite a comprehensive understanding of two main mitochondrial pathways, intrinsic apoptosis and mitochondrial permeability transition (mPT)-driven necrosis, in acute and chronic degenerative diseases, the development of an effective therapeutic strategy remains a major challenge^2,3^. Targeting late stages in apoptosis by inhibiting caspases or in necrosis by preventing mPT pore (mPTP) opening has not been achieved in clinical trials^4–6^. One potential explanation for the unsuccessful trials is the irreversible damage to the mitochondria, which has resulted in the demise of the cells. Still, it may be possible to selectively block mitochondrial-driven cell death by targeting molecular events that occur upstream and influence mitochondrial fate. Proteins belonging to the B-cell lymphoma 2 (BCL-2) family, which regulate the mitochondrial apoptotic pathway, represent a promising target in this context^7^. The core of this pathway is constituted by the executioner proteins BCL-2-associated X protein (BAX) and BCL-2 antagonist/killer 1 (BAK1). The penetration of the mitochondrial outer membrane (MOM) by BAX and/or BAK1 results in the irreversible damage of the mitochondria, which results in the release of apoptosis-inducing factors such as cytochrome *c* into the cytosol. This ultimately initiates the final stage of apoptosis through the activation of caspases^8^. Furthermore, BAX and/or BAK1 have been implicated in the regulation of necrotic cell death in ischaemia/reperfusion injury. They sensitise cells to Ca^2+^-induced opening of the mPTP in the mitochondrial inner membrane^9,10^, leading to mitochondrial swelling and rupture^11^. The activity of BAX and BAK1 is contingent upon their activation, which is facilitated by a second class of pro-apoptotic BCL-2 family proteins, the BH3-only proteins such as Bcl-2 interacting mediator of cell death (BIM) and BH3 interacting-domain death agonist (BID)^1,2^. By interacting directly with the effector proteins or associating with anti-apoptotic BCL-2 family proteins, BH3-only proteins regulate the activation of BAX and BAK1, thereby removing their inhibitory binding.

BCL-2/adenovirus E1B 19-kDa interacting protein 3 (BNIP3) is an atypical BH3-only protein, whose divergent BH3 domain is not required for hetero-/homodimerisation and cell death in comparison to the other BH3-only proteins^12^. It has been demonstrated to induce mitochondrial damage and both intrinsic apoptotic and mPTP-driven cell death^13–16^ via signalling pathways, indicating that its mode of action may involve BAX and BAK1. It is crucial to inhibit both apoptotic and necrotic pathways simultaneously, given that both apoptotic and necrotic signalling pathways are simultaneously involved in the pathogenesis of diseases such as ischaemia/reperfusion injury^17–20^. The present study identifies BNIP3 as a promising target for this objective. In order to gain insight into the potential interactions of BNIP3 with BAX and/or BAK1 and the activation of the latter through BNIP3, we conducted investigations to identify amino acid hot spots in BNIP3 that contribute to binding in the interface regions responsible for interaction and activation^21^. In this work, we have developed a BNIP3 antagonist peptide with the potential to protect cells from dysfunction and death.

## Results

### Rationale for structure-based BNIP3 antagonist peptide design

**Fig. 1:**
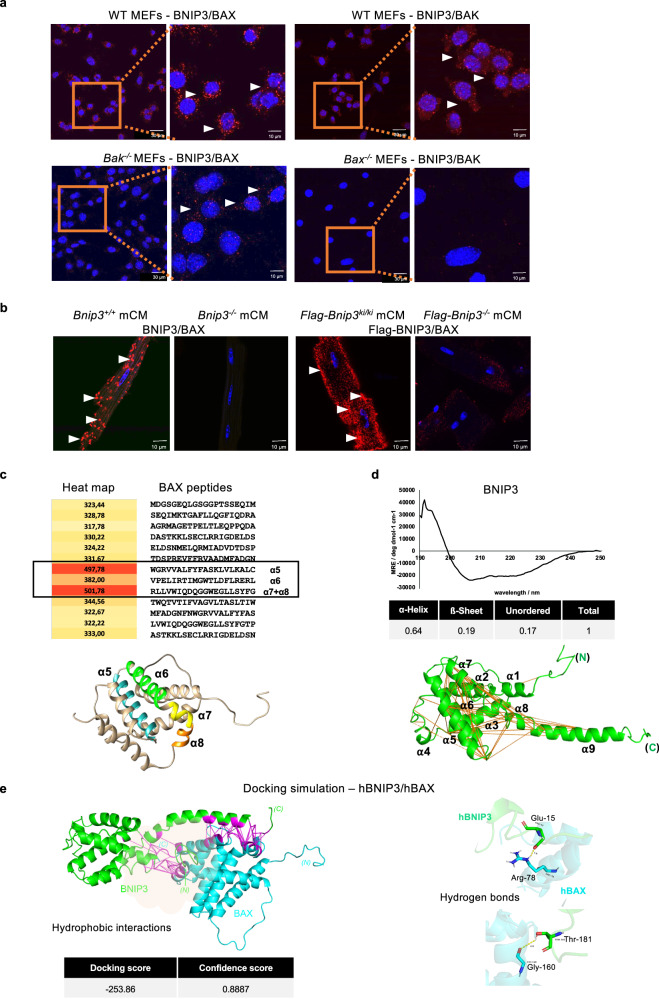
Cellular, computational and in vitro assessment of BNIP3 binding to BAX. **a** Close proximity of BNIP3 to BAX or BAK1 in mouse embryonic fibroblasts (WT MEFs) (top) and in *Bak* knockout (*Bak*^*−/−*^) or *Bax* knockout (*Bax* ^*−/−*^) MEFs (bottom). Interactions of BNIP3 and BAX or BAK1 are indicated by red dots with white arrows. Antibodies against BAX (2D2), BAK1 (4C2) and BNIP3 (1C8) were used along with a pair of secondary antibodies conjugated to complementary oligonucleotides in an in situ proximity ligation assay (PLA). (Nuclei stained with DAPI, blue; scale bars 30 µm and 10 µm, *n* = 3 independent experiments). **b** Close proximity of BNIP3 and BAX in isolated male mouse *Bnip3*^*+/+*^ cardiomyocytes (mCM) (left) and in CM of male *Bnip3-3xFlag-in* (*Flag-Bnip3*^*ki/ki*^) mice (right). (*n* = 3 independent experiments). **c** Fluorescence-labelled recombinant mouse (m)BNIP3 incubated with a library of 13 synthesised BAX peptides immobilised on microarrays to reveal potential interaction sites in BAX. The colour coding ranges from white (low or no intensity), through yellow (medium intensity), to red (high intensity) (top). Structure of mBAX (PDB 4S0O) in ribbon representation with BNIP3 interaction sites coloured blue (α5), green (α6), yellow (α7) and orange (α8) (bottom). (*n* = 3 identical subarrays with mouse IgG controls). **d** Modeller algorithm was used to predict the BNIP3 homology model based on BAX (PDB code 2K7W) as a template, followed by energy minimisation using NAMD 2.9 and CHARMM36 force field. The resulting model agreed well with the circular dichroism (top) and intramolecular cross-linking data obtained with recombinant human BNIP3 (hBNIP3). The DSSO cross-links identified in recombinant hBNIP3 by mass spectrometry were mapped onto the homology model (orange lines), supporting the predicted compact three-dimensional structure (bottom). **e** HDOCK docking of the hBNIP3 homology model and hBAX (PDB code 2k7w, model 1), with a docking score of −253.86 and a confidence score of 0.8887 (hit 1 of the top 10 optimal conformations). The predicted intermolecular hydrophobic interactions were mapped onto the docking model (magenta lines) (left). Furthermore, two hydrogen bonds were predicted (3D detail map) (right). Source data are provided as a Source Data file.

The design of molecules with selective affinity for desired targets associated with the mitochondrial pathway in both apoptotic and necrotic cell death represents a significant challenge in de novo drug design. Given that studies utilising BNIP3 and BAX/BAK1 have demonstrated that their inhibition or genetic deletion impacts apoptotic and necrotic markers^2,17^, it is reasonable to hypothesise that BNIP3, as a BH3-only protein activator, functions upstream of BAX and/or BAK1. Our recent findings demonstrate that recombinant mouse (m) BNIP3 interacts with recombinant mBAX in vitro without stimulus^22^. Prior to examining hydrophobic interactions, which are integral to binding affinity^23,24^, and key interface contact residues in BNIP3 and BAX and/or BAK1, it was necessary to ensure that the interactions occur in cells. To examine whether BNIP3 is in close proximity to BAX and BAK1, we profiled wild-type, *Bax* knockout (*Bax*^*−/−*^), and *Bak* knockout (*Bak*^*−/−*^) mouse embryonic fibroblasts (MEFs) utilising in situ proximity ligation assay under baseline conditions (PLA, Supplementary Fig. 1a, b). We used a novel monoclonal specific antibody against BNIP3 that we generated and validated in *Bnip3* knockout cells and heart tissue (*Bnip3*^*−/−*^) (Supplementary Fig. 2a–e). While BIM and BID require an apoptotic stimulus for interaction with BAX, (Supplementary Fig. 1c), we observed BNIP3 in close proximity to BAX and BAK1 even under baseline conditions (Fig. 1a, left and right, upper panel). The localisation of BNIP3 and BAX was also evident, albeit to a slightly lesser extent, in MEFs lacking BAK1 (Fig. 1a). This may be due to the absence of BAK1. In contrast, no signal for BNIP3/BAK1was detected in MEFs lacking BAX (Fig. 1a, left and right, bottom panel). One potential explanation is that the orientation of BAK1, required for close proximity to BNIP3, may depend on the presence of BAX, highlighting a crucial role of BAX in this interaction. In light of these findings and the recognition that BNIP3 is not in close proximity to the anti-apoptotic protein BCL-2 (Supplementary Fig. 1d), we focused on the interaction between BNIP3 and BAX. Subsequently, this close proximity was validated in cardiomyocytes from wild-type (*Bnip3*^*+/+*^) and *Bnip3* knockout (*Bnip3*^*−/−*^) adult male mouse hearts. The PLA signal was observed in *Bnip3*^*+/+*^ cardiomyocytes (Fig. 1b, left). Conversely, no signal was detected in cardiomyocytes lacking BNIP3 (Fig. 1b, left).

To confirm this hypothesis, we generated a novel 3x*Flag-Bnip3* knock-in mouse model using CRISPR/Cas 9-based technology (*Flag-Bnip3*^*ki/ki*^*, Flag-Bnip3*^*ki/+*^*, Flag-Bnip3*^*−/−*^; Supplementary Fig. 3a–c). Consistently, cardiomyocytes derived from *Flag*-*Bnip3*^*ki/ki*^ mice, but not those of the *Flag-Bnip3* ^*−/−*^ mouse, exhibited Flag-BNIP3/BAX complexes (Fig. 1b, right). A widespread distribution of BNIP3/BAX complexes was also observed in HEK293, MCF-7 and HepG2 cells (Supplementary Fig. 4a–d). Subsequently, we demonstrated that Flag-BNIP3 can precipitate a Flag-BNIP3/BAX complex from *Flag*-*Bnip3*^*ki/ki*^ mouse heart lysates (Supplementary Fig. 5a). Furthermore, co-immunoprecipitation experiments utilising recombinant human and mouse BAX-GST and mBNIP3-His/hBNIP3 untagged in vitro demonstrated that BNIP3 was co-isolated with BAX and vice versa, thereby providing additional evidence to support the BNIP3/BAX interaction (Supplementary Fig. 5b–d). It should be noted that BAX is in its inactive state within the identified BAX/BNIP3 complex (Supplementary Fig. 5e).

To identify potential BNIP3 binding sites in BAX, a protein-peptide interaction study was conducted using a library of 13 synthesised 20-meric BAX peptides immobilised on microarrays (Supplementary Fig. 6a). The peptides were designed to comprise either one of the BAX alpha-helices, or one of the three BH domains (Supplementary Fig. 6b). Fluorescence-labelled recombinant mBNIP3 was incubated on peptide microarrays, and BAX helices α5, α6, and α7/α8 were identified as potential interaction sites with BNIP3 (Fig. 1c). Nevertheless, this interaction may require the tertiary structure of fully folded BAX, which is not present in these peptide fragments. Additional interaction sites might therefore not be detected. Given that the three-dimensional structure of BNIP3 has yet to be fully elucidated, with the exception of its C-terminal transmembrane domain^25,26^, we conducted circular dichroism (CD) spectroscopy of recombinant hBNIP3 and employed template-based and neural network-based modelling methods (computational approaches)^27^ to predict the structure of BNIP3. Given that recombinant hBNIP3 exhibited a comparable secondary structure composition to recombinant hBAX as determined by CD spectroscopy (Supplementary Fig. 7a, b) and that the full three-dimensional structures of other BH3-only proteins were not known with the exception of BID, which, unlike BNIP3, requires cleavage for activation^1^, we decided to use BAX as a template (PDB codes 2K7W, model 1^28^ and 2KA1^26^). The homology model of BNIP3 predicted by the Modeller 9.15 algorithm^29^, followed by energy minimisation using NAMD 2.9^30^ and the CHARMM36 force field^31^ features nine α-helices of variable length representing 65% of the secondary structure. The predicted three-dimensional model demonstrated excellent agreement with the CD data obtained with recombinant hBNIP3 (Fig. 1d, top). To investigate tertiary structure, recombinant hBNIP3 was chemically cross-linked with disuccinimidyl sulfoxide (DSSO), followed by mass spectrometry analysis (XL-MS; Fig. 1d, bottom). The intramolecular cross-links identified in BNIP3 provided corroboration for the predicted compact three-dimensional structure. In contrast, the neural network-based models AlphaFold v.2.2.3 (AlphaFold Database Q12983) and RoseTTAFold (template structure: PDB code 2K7W) yielded predominantly disordered models without defined tertiary structure, consisting of 4–5 α-helices that neither match the secondary structure composition determined by CD spectroscopy nor the intramolecular cross-links (Supplementary Fig. 7c). To further assess the binding mode and key binding site between the homology model of BNIP3 and BAX, we conducted computational docking calculations using HDOCK^32^. The PDB code 2K7W (model 1) was employed as a template structure for BAX given that its nuclear magnetic resonance (NMR) structure is in good agreement with its secondary structure composition of recombinant hBAX, as determined by CD spectroscopy (Supplementary Fig. 7a). The combination of BNIP3 and BAX demonstrated a favourable docking profile, with a calculated docking score of −253.86 and a confidence score of 0.8887. BNIP3 interacts with BAX mainly through hydrophobic interactions, in particular, formed by the N-terminal residues Trp-13, Val-14, Leu-16, and His-17 of BNIP3 with residues in BAX α-helix 5, which is implicated in BAX activation^28,33,34^ (Fig. 1e, left). This interface is further stabilised by a hydrogen bond from Glu-15 in BNIP3 to Arg-78 in BAX (Fig. 1e, right). HDOCK predicts an additional hydrophobic interface involving 20 C-terminal residues of BNIP3, docking against BAX helices 8 and 9. However, this is likely an artefact of the docking of both proteins as monomers, because BNIP3 forms a stable homodimer via the coiled-coil formation of its C-terminal helix, which is also its transmembrane anchor and thus likely not available for interactions with other proteins. A computational docking simulation using HDOCK on BNIP3 and BAK1 did not yield a favourable docking profile and did not converge on one defined binding site (BAK1 PDB code 2JCN; Supplementary Fig. 8). Therefore, our studies focused on the predicted N-terminal interface involved in interaction with BAX (Fig. 1e, left; Supplementary Table 1).

### Development of BNIP3 peptide from the identified activation domain

**Fig. 2:**
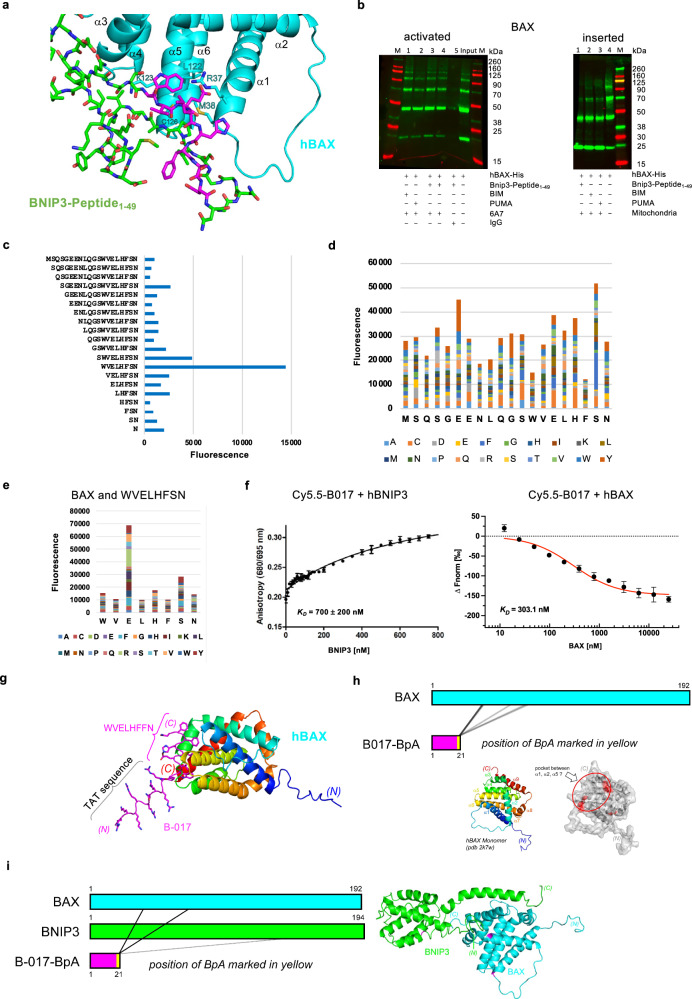
Identification of B-017, a BNIP3 antagonist peptide, by reverse engineering. **a** HDOCK docking of human (h)BAX (PDB code 2K7W) and BNIP3-peptide_1–49_ (hit 3 of top 10 optimal conformations; core binding residues are highlighted in magenta). **b** Immunoblot analyses showing the impact of BNIP3-peptide_1-49_ on the 1st (left) and 2nd (right) activation step of BAX. BIM and PUMA were used as BAX activators. (*n* = 5 independent experiments, representative immunoblots). **c** Identification of crucial N-terminal amino acid (aa) residues required for binding using a protein-peptide microarray. Fluorescence-labelled recombinant mouse (m)BNIP3 was incubated with a library of 65 synthesised peptides representing N-terminal truncations of the BNIP3 sequence MSQSGEENLQGSWVELHFSN. (*n* = 3 identical subarrays with mouse IgG controls). **d** Substitution analysis using fluorescence-labelled recombinant mBNIP3 and a library of 379 synthesised peptides. Single residues of the BNIP3 sequence MSQSGEENLQGSWVELHFSN were exchanged for 20 aa. (*n* = 3 identical subarrays with mouse IgG controls). **e** Substitution analysis with fluorescence-labelled recombinant mBAX and a library of 156 synthesised peptides. Single residues of the BNIP3 sequence WVELHFSN were exchanged for 20 aa. **f** Fluorescence anisotropy assay with titration of increasing recombinant hBNIP3 concentrations to 100 nM Cy5.5-B-017 (left). (Data points represent the mean ± SD, *n* = 3 biological replicates). Microscale thermophoresis dose-response curve of BAX binding to Cy5.5-labelled peptide. A concentration of 200 nM fluorescence-labelled peptide was incubated with serial dilutions of BAX (12.2–25,000 nM). Normalised fluorescence changes were plotted as a function of protein concentration. Data points represent the mean ± SD (*n* = 3 biological replicates). Solid red lines show the fitted binding curves (right). **g** Structure prediction of B-017 interaction with hBAX, using AlphaFold2. **h** Interactions between B-017 and recombinant hBAX, mapped by photo-cross-linking BAX:B-017-BpA complex with BpA, and identification of contacts by mass spectrometry (left). Cross-links in BAX were mapped to the BAX model (PDB code: 4S0O) (right). **i** Interactions between B-017 and BAX/BNIP3 mapped by photo-cross-linking BNIP3/BAX:B-017-BpA complex and identification of contacts by mass spectrometry (left). Cross-links in BAX and BNIP3 mapped to the docking model of the homology model of BNIP3 and BAX (PDB code: 4S0O) (right). Source data are provided as a Source Data file.

Next, we closed the structure-function gap by using the utilisation of the Graph Convolutional Network DeepFRI and the BNIP3 homology model^35^. DeepFRI predicted the structure-based molecular function gene ontology (GO) term protein binding (GO:0005515) for the first up to 50 N-terminal residues of BNIP3 (Supplementary Fig. 9a, template modelling (TM)-score of 0.58). The PredictProtein web server corroborated this prediction for mouse and human BNIP3 (Supplementary Fig. 9b). When overexpressed in HL-1 cells, a BNIP3 mutant lacking the N-terminus exhibited reduced cell death activity compared to full-length BNIP3^36^. We therefore synthesised a peptide consisting of the first 49 amino acids of hBNIP3 (MSQNGAPGMQEESLQGSWVELHFSNNGNGGSVPASVSIYNGDMEKILLD, BNIP3-peptide_1-49_). A computational docking simulation using HDOCK on hBAX (PDB code 2K7W) and BNIP3-peptide_1-49_ predicted an interaction with residues in BAX via α-helix 5 and the N-terminal half of α1-α2 loop, an important domain for its activation^37^ (Fig. 2a, Supplementary Table 2). Amino acid residues in BNIP3-peptide_1–49_ predicted to mediate this interaction overlapped with those predicted for full-length BNIP3 (Trp, Val, Leu, and His, Fig. 1e, left; Supplementary Table 1).

To clarify whether the first N-terminal residues of BNIP3 represent the discrete structural unit that directly can activate BAX, we conducted an established in vitro BAX activity assay utilising BNIP3-peptide_1-49,_ other BH3-only BAX activators (BIM and PUMA) as positive controls^38^, and bovine serum albumin and the antiapoptotic BCL_XL_ as negative controls^2^. Initial conformational change of BAX is evidenced by repositioning of the unstructured loop connecting α-helices 1 and 2^28^. This change is substantiated by the exposure of an epitope in α-helix 1 targeted by the ant-6A7 antibody^39^. To ascertain that the N-terminus of BNIP3 exhibits functional properties, we incubated recombinant hBAX with the BNIP3-peptide_1–49_ or the positive and negative controls. Using the anti-6A7 antibody simultaneously, we detected the epitope exposure as seen with BIM and PUMA (Fig. 2b left, Supplementary Fig. 10a), suggesting that BAX activation had occurred and that BNIP3 has a similar activation capacity as other BAX activators. The second essential step in the activation of BAX is the exposure of α-helix 9, which contains the transmembrane domain that is inserted into the MOM and thereby facilitates BAX interaction with the mitochondria. Antibodies that are specific to α-helix 9 are not available due to the hydrophobic nature of the epitope; however, we evaluated the membrane insertion of BAX by incubating cardiac mitochondria with recombinant hBAX and the BNIP3-peptide_1–49_, as well as with BIM and PUMA. To remove loosely attached BAX, mitochondria were subjected to alkaline extraction. Western blot analysis demonstrated that BNIP3-peptide_1–49_ specifically promotes BAX insertion into the MOM, similar to BIM and PUMA, establishing the N-terminus of BNIP3 as a functional domain (Fig. 2b right; Supplementary Fig. 10b–e). Since a reduced or altered binding motif can compete with its natural counterpart for the active site by binding tightly without undergoing turnover, thereby causing inhibition rather than activation, protein-peptide microarrays (Supplementary Fig 6a) were employed to narrow down the essential amino acids within the predicted specific region of BNIP3-peptide_1-49_ (mBNIP3 amino acids 1–20, Fig. 1e, left, Fig. 2a). In the initial phase of the study, we concentrated on the binding of BNIP3 and incubated fluorescence-labelled recombinant mBNIP3 with a library of 47 mBNIP3 N-terminal-derived truncated synthetic peptides immobilised on microarrays (Supplementary Table 3). The truncated peptide consisting of the amino acids 13–20, WVELHFSN led to the strongest signal with a 14-fold signal increase compared to the non-truncated peptide_1–20_ (Fig. 2c, Supplementary Fig. 11a). Subsequently, we conducted substitution analyses utilising fluorescence-labelled BNIP3 binding to a library of 379 mutated peptides, in which single residues of peptide sequence_1–20_ (Supplementary Table 4) were exchanged for all 20 natural amino acids. Substitution of tryptophan (W13) and phenylalanine (F18) by any canonical amino acids severely impaired BNIP3 binding (Fig. 2d, Supplementary Fig. 11b), indicating that these aromatic residues are crucial. A serine-to-phenylalanine substitution at position 19 (S19F) displayed substantially higher BNIP3 binding, supporting the importance of hydrophobic interactions (Fig. 2d, Supplementary Fig. 11b). Next, we assessed the binding of fluorescence-labelled BNIP3 and BAX, respectively, to a library of 156 peptides in which each residue of WVELHFSN was exchanged for 20 natural amino acids (Supplementary Table 5). The arrays confirmed binding to BNIP3 (Supplementary Fig. 12a) and revealed binding affinity towards BAX (Fig. 2e), both of which were strengthened by the S19F substitution. These binding data were in good agreement with the substitution array using the non-truncated sequence_1–20_ (Fig. 2d). Docking simulations on inactive BAX (PDB code 4S0O) with the sequence WVELHFSN and the sequence with S-to-F substitution, WVELHFFN, with HADDOCK showed that both peptides are predominantly placed over the N-terminal end of the BAX α-helix 5 (Supplementary Fig. 12b).

As our findings indicate that the sequence stretch WVELHFFN binds to BNIP3 and BAX with strong affinity_,_ we conceived a peptide, B-017, composed of this octamer (Supplementary Fig. 13a, top), fused to the HIV-1 TAT protein transduction domain_48-59_ (PTD; GRKKRRQRRRPQ) as a delivery sequence, which enhances solubility and can penetrate cell membranes^40^. CD spectroscopy of chemically synthesised B-017 peptide TAT-WVELHFFN confirmed a random coil conformation (Supplementary Fig. 13a, bottom). To design a dysfunctional peptide as a negative control, we substituted potential interfacial residues, including F19 to S19, F18 to A18 and H17 to A17, based on the alanine scan, thereby generating the TAT-WVELAASN peptide (Supplementary Fig. 13b). Fluorescence-based overlay assays and Western blot analyses verified that B-017 binds to both BNIP3 and BAX (Supplementary Fig. 13c, d). B-017 and the negative control peptide were also taken up by cells, as shown in MCF-7 cells (Supplementary Fig. 13e, f). Fluorescence anisotropy titrations of BNIP3 and BAX to Cy5.5-labelled B-017 yielded a *K*_D_ of 700 ± 200 nM (Fig. 2f, left) and a *K*_D_ = 303 nM (Fig. 2f, right), respectively. Next, modelling of BAX with B-017 using AlphaFold2 v.2.2.3 revealed the binding of B-017 through the octamer WVELHFFN without interaction of the TAT delivery sequence (Fig. 2g). To identify the binding interface of the B-017 with BAX and the BAX/BNIP3, we performed photo-cross-linking with B-017 carrying a photo-reactive-p-benzoyl-L-phenylalanine (BpA) moiety at the C-terminus (B-017-BpA), followed by mass spectrometry (XL-MS)^41^. The photo-cross-linked B-017-BpA:BAX complex formed cross-links to BAX residues M38 and L70 (Fig. 2h). Mapping the cross-links to the BAX structure (PDB code 2K7W), the B-017 binding is localised in a pocket between the BAX helices α1, α2, and α5 (Fig. 2h). Next, we evaluated the B-017-BpA binding by BAX in the context of the ternary complex, including BNIP3. Cross-linking of the ternary complex showed two cross-links between B-017-BpA and BAX (M38, L70) (Fig. 2i) that are consistent with those observed for the binary B-017-BpA:BAX complex (Fig. 2h). Moreover, B-017-BpA also shows a cross-link to BNIP3 (T140) (Fig. 2i).

### Signalling profile of the BNIP3 antagonist peptide B-017

**Fig. 3:**
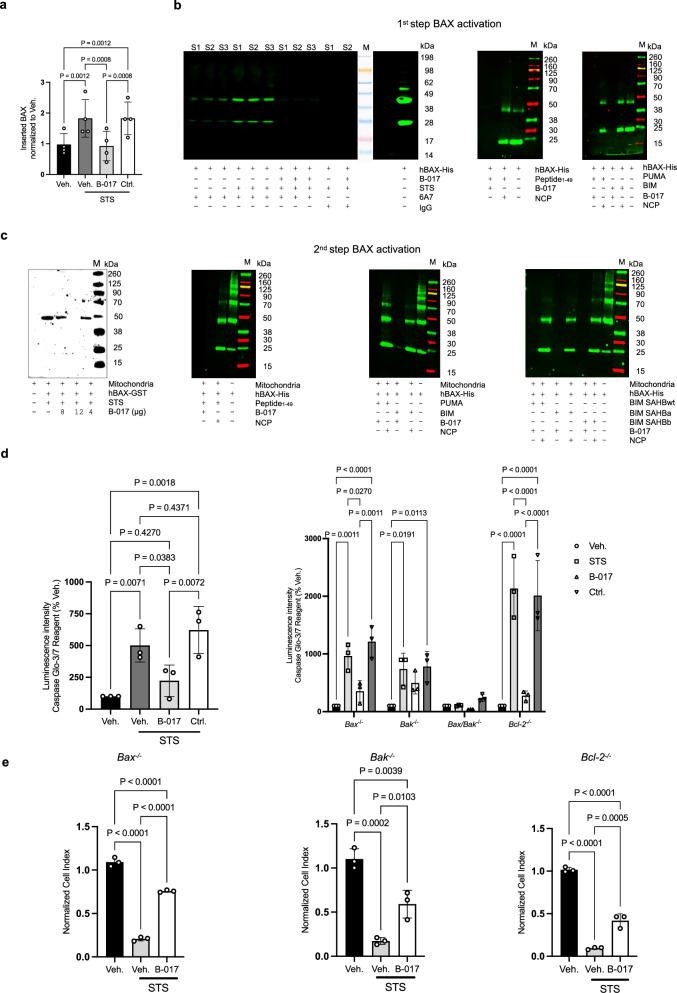
Signalling profile of B-017. **a** Immunoblot analysis showing the effect of B-017 on the signalling of mitochondrial-induced cell death, characterised by BAX insertion into the mitochondrial outer membrane. Wild-type mouse embryonic fibroblasts (WT MEFs, 3 × 10^6^ cells) were exposed to staurosporine (STS) (2 µM) and B-017 (146 µM), vehicle (Veh.), or the negative control peptide (Ctrl., 146 µM). To assess BAX insertion, mitochondria were isolated and subjected to alkaline extraction to remove loosely attached BAX. (*n* = 4 independent experiments, repeated-measures ANOVA, two-sided, data are presented as mean ± SD). **b** Immunoblot analyses showing the impact of B-017 on the 1st activation step of BAX using the 6A7 antibody to detect early conformational changes in BAX. STS, BNIP3-Peptide_1–49_ (Peptide_1–49_), PUMA and BIM were used as stimulation agents; the negative control peptide (NCP) served as a control. (*n* = 3 independent experiments, Supplementary Fig. 14b). **c** Immunoblot analyses showing impact of B-017 on the 2nd activation step, characterised by BAX insertion into the mitochondrial outer membrane (MOM). To assess the percentage of BAX that is inserted into the MOM, mitochondria were treated with strong alkali, which extracts only loosely attached BAX, not that which is inserted. STS, Peptide1–49, PUMA, BIM, and BIM-SAHB variants were used as stimulating agents; NCP served as a control. (Representative immunoblots of *n* = 4 and *n* = 5 independent experiments). **d** Response to B-017 in terms of late-stage cell death signalling, characterised by caspase 3/7-activity, in STS-treated WT MEFs (left) and MEFs lacking BAX, BAK, BAX/BAK, or the anti-apoptotic protein BCL-2 (right). (WT MEFs *n* = 3 independent experiments, Knockout MEFs *n* = 3 experiments with four technical replicates, two-way ANOVA, data are presented as mean ± SD). **e** Cellular response to B-017 treatment in different knockout MEFs exposed to cell death signalling stimulation using live cell impedance measurements. *Bax*^*−/−*^, *Bak*^*−/−*^, and *Bcl-2*^*−/−*^ MEFs were treated simultaneously with 100 nM STS and B-017 (52 µM) or vehicle after reaching a cell index of 1, indicating a stable adhesion to the microtiter plate. (*n* = 3 independent experiments, two-way ANOVA, data are presented as mean ± SD). Source data are provided as a Source Data file.

To establish a link between the efficacy of B-017 and its ability to inhibit BAX activation and prevent its insertion into the outer membrane, we treated wild-type MEFs and MCF-7 cells with the apoptosis-inducing agent staurosporine (1 µM) and B-017 for 4 h. B-017 treatment provided significant protection of the MOM, resulting in reduced levels of inserted BAX compared to mitochondria from wild-type MEFs and MCF-7 cells treated with staurosporine alone. Furthermore, BAX levels in mitochondria from B-017 treated cells were similar to those from cells treated with vehicle or negative control peptide (Fig. 3a, Supplementary Fig. 14a). The negative control peptide showed no protective effect, and no differences were detected between vehicle and negative control peptide, indicating that possible effects of the TAT sequence or a peptide per se can be excluded.

Given the established role of the BAX α1 region in BAX activation^28,42,43^ and the evidence of B-017 binding in the vicinity of helix α1, as revealed by cross-linking analysis, we conducted an in vitro study to investigate the impact of B-017 on BAX activation. We assessed BAX conformational alteration induced by staurosporine, BNIP3-peptide_1-49_ and BAX activators that act at different interaction sites^28,44^, in the presence of the anti-6A7 antibody. B-017 treatment prevented consistent 6A7 epitope exposure (Fig. 3b, Supplementary Fig. 14b), suggesting that BAX activation is successfully inhibited. To evaluate our hypothesis that B-017 also circumvents exposure of α-helix 9 with subsequent membrane insertion of BAX, we incubated cardiac mitochondria and recombinant BAX with staurosporine, BNIP3-peptide_1–49_ or other BAX activators in the presence of B-017. Western blot analysis demonstrated that B-017 consistently markedly inhibits the BAX insertion into the MOM, thereby preventing MOM permeabilisation (Fig. 3c, Supplementary Fig. 14c). It can be inferred that BAX may also be activated indirectly through the inhibition of BCL-2^2,45^. Given that BNIP3 has been observed to heterodimerise with BCL-2 in vitro^12^, we examined the response to B-017 in staurosporine-treated MEFs lacking BCL-2 family members in terms of late-stage cell death signalling (Fig. 3d, Supplementary Fig. 14d). BCL-2 ablation resulted in a substantial increase in caspase activity, which was prominently alleviated by co-treatment with B-017. As anticipated, MEFs lacking BAX exhibited decreased caspase activity compared to wild-type MEFs under staurosporine, and this effect was mitigated by B-017. The knockout of BAK1 in MEFs also resulted in the anticipated decline in caspase activity, which was moderately diminished by B-017 (Fig. 3d, Supplementary Fig. 14d). The results obtained from MEF knockout cells suggest that B-017 has the potential to impede the activity of BAX and BAK1. This finding is further substantiated by a B-017 efficacy assay conducted in the context of cell viability testing following sublethal treatment with staurosporine, as measured using a cell adhesion assay (Methods). In both *Bax*^*−/−*^ and *Bak1*^*−/−*^ MEFs, B-017 displayed protection against staurosporine-induced apoptosis (Fig. 3e). Despite the fact that B-017 does not appear to bind exclusively to BAX, the primary mechanism of action appears to involve this interaction, particularly in light of the requirement of BAX for the interaction of BNIP3 with BAK1 (Fig. 1a)^46,47^.

### B-017 inhibits apoptotic and necrotic signalling pathways in human cells

**Fig. 4:**
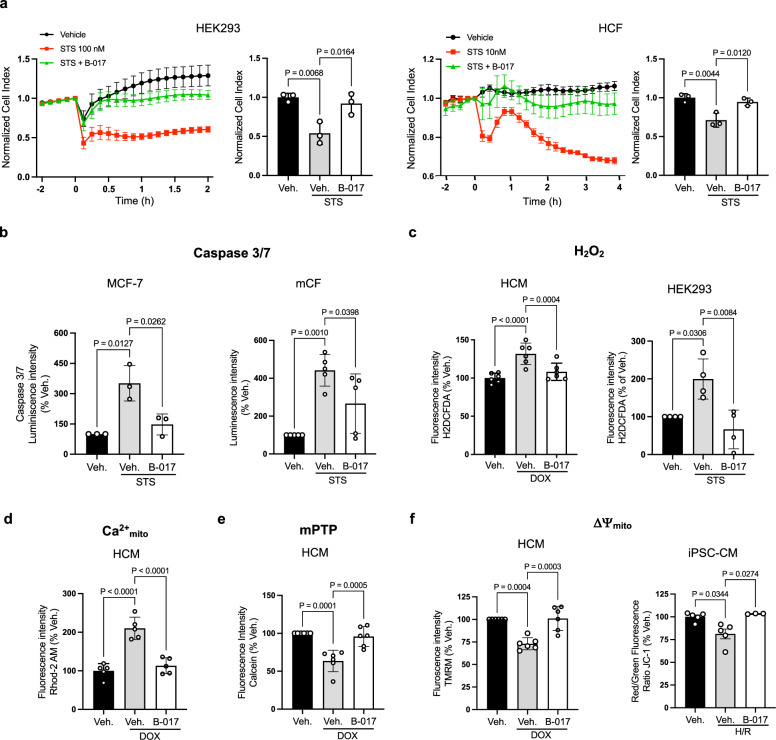
B-017 protects cell viability and mitochondrial health. **a** Cellular response to B-017 treatment in different cell lines exposed to cell death signalling stimulation using live cell impedance measurements. HEK293 cells (8000 cells per well) and human cardiac fibroblasts (HCF; 2000 cells per well) were treated simultaneously with 100 nM or 10 nM staurosporine (STS) and B-017 (52 µM) or vehicle (Veh.) after reaching a cell index of 1, indicating a stable adhesion to a microtiter plate consisting of gold microelectrodes fused to the bottom of the surface. The time point chosen to assess the differences was 1 h after treatment for HEK293 cells and 4 h for HCF to exclude a proliferation effect. (*n* = 3 independent experiments). **b**–**f** Cellular consequences of B-017 treatment in MCF-7 cells, mouse cardiac fibroblasts (mCF), human cardiomyocytes (HCM), HEK293 cells, and induced pluripotent stem cell-derived cardiomyocytes (iPSC-CM), related to **b** caspase 3/7 activity (MCF-7: *n* = 3 independent experiments; mCF: *n* = 5 independent experiments), **c** reactive oxygen species generation (HCM: *n* = 6 independent experiments; HEK293: *n* = 4 independent experiments), **d** mitochondrial Ca^2+^ overload (*n* = 5 independent experiments), **e** formation of the mitochondrial permeability transition pore (mPTP) (*n* = 6 independent experiments), and **f** mitochondrial membrane depolarisation (ΔΨ_mito_) (HCM: *n* = 6 independent experiments and iPSC-CM: *n* = 5 (Veh., Veh. in H/R) and *n* = 3 (B-017 in H/R) independent experiments. STS (500 nM, 2 µM), doxorubicin (DOX, 10 µM), and hypoxia/reoxygenation (H/R) were chosen as cell death signalling inducing agents; B-017 (52 µM) or vehicle was administered concomitantly with the stimulus. Luminescence intensity of caspase-Glo 3/7 reagent was assessed using a luminescence microplate reader, and fluorescence intensity of H_2_DCFDA (H_2_O_2_), rhodamine-2 AM (Ca^2+^_mito_), TMRM (ΔΨ_mito_), and calcein (mPTP) was measured using a fluorescence microplate reader. Fluorescence intensity of H_2_DCFDA (H_2_O_2_) and JC-1 (ΔΨ_mito_) was determined by fluorescence microscopy. One-way ANOVA, data are presented as mean ± SD. Source data are provided as a Source Data file.

To gain insight into the inhibitory effects of B-017, we examined its impact on cell death signalling in several mouse and human cell lines. To determine whether B-017 mitigates staurosporine-induced apoptosis, a cell adhesion assay was employed in HEK293 cells and human cardiac fibroblasts (HCF) using a sublethal concentration of staurosporine (10 nM and 100 nM) and B-017 (52 µM) or vehicle^48^. B-017 treatment resulted in robust maintenance of HEK293 cells and HCF adhesion compared with cells treated with staurosporine alone, with adhesion kinetics resembling those of vehicle-treated cells, as evidenced by one-point analyses (Fig. 4a, left and right, Supplementary Fig. 15a, b). The efficacy of B-017 was also evident in staurosporine-treated HepG2 and MCF-7 cells (Supplementary Fig. 15c, d), indicating that B-017 effectively counteracts staurosporine-impaired cell viability.

The critical regulatory events in mitochondrial-dependent apoptosis and necrosis are the permeabilisation of the mitochondrial outer and inner membranes. Pore formation in the outer membrane allows release of apoptogens such as cytochrome *c*, which activates caspase 3/7^9,49–52^ and generates reactive oxygen species (ROS)^53^. Mitochondrial Ca^2+^ overload sensitises the opening of the mPTP and depolarisation of the mitochondrial inner membrane^9,10,17,51,52,54,55^. To induce the characteristic features of apoptosis and necrosis, cells were exposed to the apoptosis inducer staurosporine (500 nM, 2 µM), doxorubicin (10 µM) and hypoxia/reoxygenation, the latter two stimulating the cells to apoptosis and necrosis^38^. As controls, we used the vehicle and the negative control peptide to exclude potential effects of the TAT sequence or the peptide per se. B-017 potently antagonised apoptotic cell death pathway, as evidenced by its inhibitory effect on caspase 3/7 activity in both MCF-7 and mouse cardiac fibroblasts (mCF) (Fig. 4b) and by reduced ROS generation in human cardiomyocytes (HCM), HEK293 cells (Fig. 4c, Supplementary Fig. 16a), MCF-7 cells, and HCF (Supplementary Fig. 17a). Moreover, B-017 treatment protected mitochondria from Ca^2+^ overload in HCM (Fig. 4d, Supplementary Fig. 17e), hindered mPTP formation in HCM (Fig. 4e), and preserved mitochondrial inner membrane potential in HCM (Fig. 4f left), iPSC-derived cardiomyocytes (Fig. 4f right, Supplementary Fig. 16b), and MCF-7 cells (Supplementary Fig. 17b). No effect of the TAT sequence or the negative control peptide was observed (Supplementary Fig. 17c–e). These findings demonstrate the ability of B-017 to impede the processes of apoptosis and necrosis induced by diverse stimuli in various mouse and human cell types.

### B-017 possess cytoprotective properties in clinically relevant models

Given its ability to inhibit both necrosis and apoptosis in cellular assays, we aimed to establish whether B-017 could be a potential therapeutic agent in clinically relevant situations. Initially, we assessed whether B-017 meets key criteria for a therapeutic agent, such as target specificity and safety. Off-target analyses were performed using microscale thermophoresis and fluorescence anisotropy. Fluorescence-labelled B-017 was titrated with increasing concentrations of the anti-apoptotic BCL-2 family members hBCL-2 and hMCL-1, which inhibit the oligomerisation required for the MOMP formation by activated and already inserted hBAX. With K_D_ values of approximately 1.3 µM for BCL-2 and MCL-1, B-017 bound these proteins with a 4-fold lower affinity than hBAX (K_D_ = 303 nM) (Supplementary Fig. 18a, b). The notion that some binding to BCL-2 and MCL-1 occurred is likely attributable to the propensity of BNIP-3-derived peptides to interact with proteins exhibiting high structural similarity^56^, like BCL-2 family proteins. Consistently, BCL-2 and BAX display similar structures with a root mean square deviation of ~1.9–2.2 Å^57^. By contrast, no binding of B-017 was detected to the structurally unrelated protein glutathione-S- transferase (Supplementary Fig. 18c), supporting a low likelihood of off-target responses. Next, we conducted a toxicological study to evaluate the B-017 safety profile. B-017 did not cause toxicity as inferred from body weight monitoring in female and male rats administered 0, 3, 6 and 12 mg kg^−1^ for 14 days, followed by a 14-day recovery phase: no body weight loss occurred during dosing or recovery (Supplementary Fig. 19). In addition, no B-017-related changes were detected in serum-chemistry and urine parameters, and no visible lesions were observed in examined tissues and organs, including heart, liver, or brain, in either sex (Supplementary Tables 6 and 7, Supplementary Data 2–5). During the dosing phase, treatment-related transient microscopic findings were confined to the injection site and included subcutaneous haemorrhage, mixed-cells inflammation, and necrosis of the skin, subcutaneous tissue and underlying muscle in both sexes (Supplementary Data 4 and 5), accompanied by transient haematological changes (Supplementary Table 8, Supplementary Data 6). All these observations had been resolved by the end of the recovery phase. Systemic exposure to B-017 increased proportionally with dose, with no sex-related differences, and a terminal half-life of 0.3 to 0.6 h (Supplementary Table 9). Taken together, the appreciable target specificity and favourable safety profile support the suitability of B-017 as a potential therapeutic agent.

**Fig. 5:**
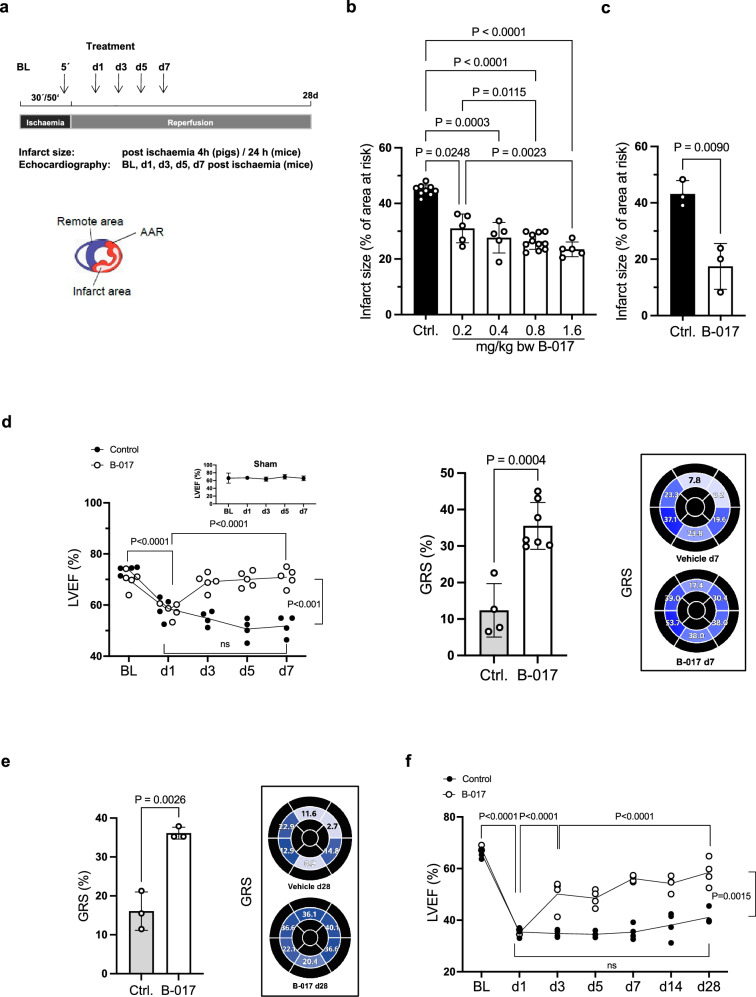
B-017 induces protective biological responses in ischaemia/reperfusion injury. **a** Study design created with Microsoft PowerPoint. Blue: remote area, red: area at risk (AAR), white: infarcted tissue. BL, baseline **b** Mice were subjected to 30 min of ischaemia and 24 h of reperfusion, and injected intracardially with vehicle (0.9% sodium chloride, Ctrl.) or B-017 as indicated, 5 min prior to reperfusion. Infarct sizes were assessed as infarcted tissue/AAR. (*n* = 9 (Ctrl.), *n* = 5 (0.2, 0.4, and 1.6 mg kg^−1^ BW), *n* = 11 (0.8 mg kg^−1^ BW) C57BL/6 J male mice, one-way ANOVA). **c** Therapeutic response to B-017 in pigs exposed to 60 min of ischaemia and 4 h of reperfusion, injected intravenously with 0.9% sodium chloride (Ctrl.) or B-017 (0.075 mg kg^−1^ BW) 5 min prior to reperfusion. (*n* = 3 per group, two-tailed Student’s *t* test). **d**, **e** Therapeutic response of cardiac function to B-017. Mice subjected to 50 min of ischaemia followed by 7 or 28 days of reperfusion were injected with either negative control peptide (Ctrl.) or B-017 (0.8 mg kg^−1^ BW, each) intracardially, 5 min before reperfusion, and intraperitoneally on d1, d3, d5, and d7 after onset of reperfusion. Left ventricular ejection fraction (LVEF) changes, as monitored by serial echocardiographic examinations, were calculated. Myocardial contraction changes were detected by speckle-tracking-based strain analyses on d7 or d28. Global radial strain (GRS) was measured midventricular. Representative 6-segment bull’s eye maps of GRS assessed by using parasternal short-axis imaging on d7 or d28 (7-day cohort: *n* = 4 untreated and *n* = 5 B-017-treated C57BL/6 J male mice; 28-day cohort: *n* = 3 untreated and treated C57BL/6 J male mice). Two-way ANOVA or two-tailed Student’s *t* test. **f** Therapeutic response to B-017 in the development of heart failure. Mice subjected to 50 min of ischaemia and 28 days of reperfusion were injected intraperitoneally with negative control peptide (Ctrl.) or B-017 (0.8 mg kg^−1^ BW, each) on d1, d3, d5, and d7 after the onset of reperfusion. LVEF changes were calculated. (*n* = 4 C57BL/6 J male mice per group). Two-way ANOVA. **b**–**e** Data are presented as mean ± SD. Source data are provided as a Source Data file.

A substantial indicator of potential cell death inhibition is ischaemia/reperfusion injury, which can lead to heart failure after myocardial infarction^17,19,20,58–60^. Reperfusion of ischaemic myocardium is essential for tissue salvage but paradoxically accounts for up to 50% of infarct size^61^, thereby promoting left ventricular (LV) dysfunction, adverse remodelling, and heart failure^62^. Mitochondria are responsible for cardiomyocyte death through necrosis and apoptosis in ischaemia/reperfusion^62^, both initiated by the disintegration of mitochondrial membranes. We therefore subjected C57BL/6 J wild-type mice (12 ± 3 weeks old) to the established in vivo ischaemia/reperfusion model^19,20,59^, with 30 min of left coronary artery (LCA) occlusion followed by reperfusion, and treated mice with B-017 (Fig. 5a, left). Because administration of cardioprotective agents after onset of reperfusion is largely ineffective^63^, we delivered B-017 in the LV cavity 5 min before reperfusion, a clinically feasible time point^62^. To determine myocardial peptide distribution following intracardiac injection, ischaemic C57BL/6 J wild-type mice were injected with fluorescence-labelled B-017 or negative control peptide 5 min before reperfusion. Light sheet microscopy of the entire heart^60^ showed that both peptides were present in the heart 5 min after reperfusion (Supplementary Fig. 20a), including the are at risk, as well as in other organs such as spleen, liver, brain, and plasma (Supplementary Fig. 20b). Given that prognosis following myocardial infarction depends on the amount of dead tissue (infarct size), we proceeded to measure the infarct size per area at risk at 24 h reperfusion. B-017 provided substantial tissue protection, reducing infarct size in a dose-dependent manner by 40% relative to vehicle (Fig. 5b, Supplementary Fig. 20c, d). To exclude effects of the TAT sequence or peptide per se, control groups received intracardially TAT-*ß*-Gal or negative control peptide (TAT-WVELAASN); neither differed from vehicle and neither conferred protection. Sham-operated mice showed no infarct (Supplementary Fig. 20e).

To investigate translational potential, we selected the catheter-based myocardial infarction model in pigs due to similarities in organ size, coronary anatomy, immunology, and physiology with humans^64,65^. Animals underwent 60 min LCA balloon inflation followed by 4 h reperfusion. B-017 or sodium chloride was administered via intravenous bolus injection through the femoral vein 5 min prior to reperfusion. A single intravenous bolus injection of 0.075 mg kg^−1^ of B-017 was sufficient to reduce infarct size by 60% in pigs compared to appropriate controls (Fig. 5c, Supplementary Fig. 20f).

Myocardial ischaemia/reperfusion injury results in impaired LV function, adverse remodelling, and ultimately heart failure^61^. We first assessed whether B-017 affects baseline cardiac function in mice and haemodynamics in dogs, which is crucial for translational research^66^. In healthy animals, B-017 did not impact cardiac function in mice (Supplementary Fig. 21a), as indicated by LV ejection fraction (LVEF)^67^, nor systolic, diastolic, mean arterial pressure, or heart rate to respective pretreatment baseline values in dogs (Supplementary Fig. 21b). In the ischaemia/reperfusion setting, all mice received B-017 or the negative control peptide intracardially 5 min before and intraperitoneally on consecutive days 1, 3, 5, and 7 of reperfusion. Irrespective of treatment, all mice exhibited a comparable decline in systolic LV function on day 1, assessed by LVEF and speckle tracking-based strain. The B-017-treated group demonstrated recovery of LVEF by day 3, whereas the control group exhibited no change (Fig. 5d). In accordance with this, strain analyses, a sensitive tool for detecting regional changes in myocardial contraction, revealed significant improvement in global longitudinal, circumferential, and radial strain on day 7 under B-017 treatment compared to controls (GLS −10% vs. −6%, GCS −22% vs. −10%, GRS 35% vs. 12%). Segmental contractility changes were mainly seen in anterior, anterolateral, and inferolateral segments (Fig. 5c, Supplementary Fig. 21c). Similar findings were obtained in a separate cohort with 28 days of reperfusion (Fig. 5d; GRS 26% vs. 16%). To evaluate the efficacy of B-017 exclusively on heart failure development, mice received either B-017 or a negative control peptide intraperitoneally only after the onset of ischaemia/reperfusion on days 1, 3, 5, and 7. This regimen again showed that B-017-treated mice experienced improvement in LVEF by day 3, with full recovery by day 28, whereas the control group showed no LVEF change (Fig. 5e). This is an initial indication that B-017 may also be beneficial in limiting chronic heart failure. Additional cardiac function indices and standard endpoints, including histological assessment of fibrosis and heart weight/tibia length ratio, are required to substantiate this observation and more comprehensively evaluate cardiac remodelling.

Given the critical role of the mitochondrial-dependent pathway in other forms of ischaemia/reperfusion injury, such as ischaemic stroke, we evaluated B-017 in focal cerebral ischaemia. Mice underwent 30 min transient middle cerebral artery occlusion, followed by 24 h reperfusion, and were monitored for global neurological impairments. Intravenous B-017 given before ischaemia significantly reduced infarct size by 52% compared to controls (Supplementary Fig. 22a, left) and improved Bederson scores (Supplementary Fig. 22a, right, Supplementary Table 10). We then examined the potential therapeutic benefits of B-017 in ischaemia/reperfusion injury in organ transplantation, utilising an established liver reperfusion model^68,69^. Explanted and ischaemically stored rat livers were flushed with saline with or without B-017 (Supplementary Fig. 22b, top). B-017 markedly diminished tissue injury at 120 min of reperfusion, as indicated by reduced hepatocellular injury markers, alanine transaminase (Supplementary Fig. 22b, middle left) and aspartate aminotransferase (Supplementary Fig. 22b, middle right), relative to the negative control peptide. Furthermore, B-017 enhanced functional recuperation, as evidenced by elevated hepatic bile production during reperfusion compared to the negative control peptide (Supplementary Fig. 22b, bottom), pointing to potential advantages of B-017 in organ preservation.

**Fig. 6:**
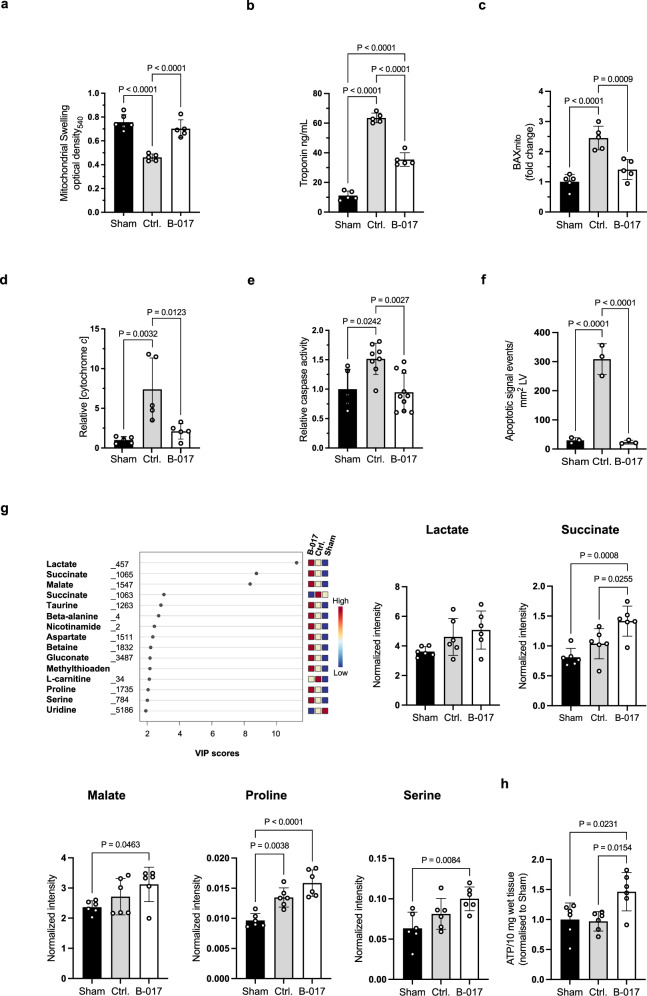
Therapeutic effect of B-017 on cell death signalling. **a**–**h** Therapeutic effect of B-017 on cell death signalling and cardiac metabolomics in ischaemia/reperfusion. Mice were exposed to 30 min of ischaemia and different reperfusion durations. Mice were injected with B-017 (0.8 mg kg^−1^ BW) or negative control peptide (Ctrl., 0.8 mg kg^−1^ BW) 5 min before reperfusion. Sham-operated mice served as controls. The necrotic pathways indicators mitochondrial swelling and cardiac troponin I were measured (**a**) by optical density at 540 nm of mitochondria isolated from the AAR after 10 min of reperfusion (*n* = 5 C57BL/6 J male mice per group) and in serum after 24 h of reperfusion (*n* = 5 C57BL/6 J male mice per group). **b** Higher optical density indicates less swelling. **c**, **d** Immunoblot analyses showing mitochondrial BAX level after 10 min and cytoplasmic cytochrome *c* level after 30 min of reperfusion in the AAR (*n* = 5 C57BL/6 J mice per group). **e** caspase-3 activity at 4 h reperfusion (*n* = 5 (Sham), *n* = 8 (Ctrl.), *n* = 10 (B-017) C57BL/6 J mice per group). **f** Apoptosis at 4 h reperfusion. The total number of apoptotic signal events per left ventricle (LV) was counted in four plane sections of the heart with three slices each (Ctrl. and B-017 group) and one each (sham group), and calculated per 1 mm^2^ of LV tissue. Images were captured using an inverted epifluorescence microscope and processed in ImageJ software 1.52a (NIH). Ctrl., negative control peptide. (*n* = 3 C57BL/6 J male mice per group). **g**, **h** Altered cardiac metabolomics in the area at risk after 60 min of reperfusion following 30 min of ischaemia. Screen of 15 differential metabolites fulfilling the variable importance projection (VIP) criterion with a value ≥ 1 (middle). Bar graphs showing levels of the glycolytic end product lactate, TCA cycle intermediates succinate and malate, the amino acids proline and serine, and **h** adenosine triphosphate (ATP). (*n* = 6 C57BL/6 J male mice per group). **a**–**h** One-way ANOVA. Data are presented as mean ± SD. Source data are provided as a Source Data file.

To demonstrate causality between B-017 and mitochondrial-dependent necrotic and apoptotic pathways in ischaemia/reperfusion injury, we first exposed wild-type C57BL/6 and *Bnip3*^*−/−*^ knockout mice to 30 min ischaemia, followed by 24 h reperfusion. *Bnip3* deletion generally protected cardiac tissue from ischaemia/reperfusion injury, as evidenced by a 51% reduction in infarct size compared to wild-type mice. However, intracardiac treatment of *Bnip3*^*−/−*^ mice with B-017 five minutes before reperfusion provided no additional protection. This finding supports causality between B-017 and the mitochondrial-dependent necrotic and apoptotic pathways and underscores the relationship between B-017’s mode of action and BNIP3/BAX (Supplementary Fig. 23a). We then profiled serum and the reperfused area at risk of hearts from both B-017 treated and untreated wild-type mice for necrotic and apoptotic markers in the context of myocardial infarction. B-017 reduced mitochondrial swelling, accompanied by a significant decrease in serum concentrations of cardiac troponin (cTnI)–a marker of cardiomyocyte necrosis, clinically utilised for the diagnosis of myocardial infarction–compared to controls (Fig. 6a, b, Supplementary Fig. 23b)^70^. A significant reduction in total mitochondrial BAX, comprising loosely attached and inserted forms, was observed following B-017 treatment (Fig. 6c). This resulted in a notable decrease in cytochrome *c* release (Fig. 6d, Supplementary Fig. 23c) and caspase activity (Fig. 6e), and finally in apoptotic cell death (Fig. 6f). These findings suggest that B-017 treatment is a successful in vivo intervention for ischaemia/reperfusion injury, reducing both necrotic and apoptotic cell death signalling pathways. As with other cardiovascular diseases, ischaemia/reperfusion injury also involves a mitochondrial metabolic disorder originating from sudden disruption of metabolism during ischaemia and abrupt restoration of specific metabolic pathways upon reperfusion, which damages mitochondria^71^. To ascertain whether the salvage of mitochondria by B-017 influences cardiac metabolism, we conducted a comprehensive LC-MS/MS-based metabolomic analysis of hearts at 60 min reperfusion with focus on glycolysis, the tricarboxylic acid (TCA) cycle, and amino acid metabolism. Lactate, the glycolytic end product, was increased under B-017 treatment compared to sham and control groups, consistent with recent findings that targeting the pyruvate-lactate metabolism by inhibiting lactate export could be a promising protective therapy^72^ (Fig. 6g). Principal TCA cycle intermediates elevated in B-017-treated hearts relative to control and sham were succinate, fumarate, malate, and the TCA cycle overall rate-determining intermediate α-ketoglutarate (Fig. 6g; Supplementary Fig. 24a, b). Similarly, amino acid metabolism, which is influenced by TCA cycle dysfunction^73^, was regulated by B-017, as evidenced by elevated proline, serine, and asparagine levels compared to non-treated and sham groups (Fig. 6g, Supplementary Fig. 24c). Given the close coupling between TCA cycle activity and myocardial contractile function, and the development of LV dysfunction following ischaemia/reperfusion injury, the elevation of TCA cycle intermediates and amino acid levels under B-017 treatment may reflect a compensatory metabolic response that contributes to preserved contractile function, supported by enhanced ATP generation under B-017 treatment (Fig. 6h).

To sum up, our study shows that B-017 has beneficial effects in ischaemia/reperfusion injury by limiting cell death through apoptosis and necrosis across multiple clinically relevant models.

## Discussion

Mitochondria play a pivotal role in the induction of both physiological and pathological cell death, functioning as essential effectors in this process^1,74^. In the event of damage, they are capable of translating the injury into lethal apoptotic and necrotic pathways, which can be observed in a number of acute and chronic degenerative diseases^58^. In diseases such as ischaemia/reperfusion injury, both forms of cell death are induced simultaneously^17^. Despite promising preclinical results, none of the drug candidates to treat ischaemia/reperfusion injury has been successfully translated into clinical practice^6^. Here, reverse engineering has been employed to enhance comprehension of the structure and function of the regulatory network of BCL-2 family proteins. This has involved a focus on the BH3-only protein BNIP3 and the executioner proteins BAX and BAK1, with the objective of developing a structure-based drug targeting mitochondrial-driven cell death. The application of cellular, computational and experimental binding studies revealed a robust and dominant interaction between BNIP3 and BAX and the functional domain in BNIP3 for activating BAX. The identification of an interface involving the first 20 amino acids of BNIP3 in hydrophobic interactions provided the basis for the design of an 8 amino acid long BNIP3 antagonist peptide, WVELHFFN, which has been demonstrated to efficiently protect mitochondria. Truncation and substitution analyses were conducted on the initial 20 amino acids, resulting in the design of an octamer with high affinity for BNIP3 and BAX. Covalent binding of the cell membrane-penetrating HIV-1 TAT protein transduction domain_48–59_ to the octamer, which overcomes the issue of the cellular uptake of peptides, enables B-017 to reach the intracellular compartment. In vitro assays demonstrated that B-017 binds to BNIP3 and BAX, preventing the direct activation of BAX and its insertion into the mitochondrial outer membrane. This was also observed in cell culture. Furthermore, cell culture experiments demonstrated the inhibition of BAK1 activity. The development of a peptide derived from the N-terminus of hBNIP3 has been shown to facilitate mitochondrial protection by competing with the BAX-activating domain of BNIP3 based on hotspot mimicry of key hydrophobic residues impeding the BAX-initiated point of no return and halting the signalling cascade that culminates in apoptosis and necrosis in human cells (Supplementary Fig. 25a). This has been established in human cardiomyocytes derived from two distinct donors and in diverse human cell lines. The efficacy of B-017 in salvaging heart, brain and allograft tissue was demonstrated in clinically relevant models for ischaemia/reperfusion injury in the settings of myocardial infarction, ischaemic stroke, organ transplant and development of heart failure. B-017 was demonstrated to possess advantageous pharmacological characteristics, which are manifested in the regulation of mitochondrial health. In mouse hearts that underwent myocardial infarction, with a uniform distribution of B-017 throughout the cardiac tissue, the typical hallmarks of both apoptosis and necrosis have been markedly reduced, while the cardiac metabolism has undergone a notable alteration upon B-017 administration. The restoration of cardiac function, which is markedly compromised in ischaemia/reperfusion injury, with B-017 therapy highlights the vital necessity for mitochondrial protection.

In conclusion, the present findings demonstrate that the identified BNIP3-BAX/BAK1 axis represents a crucial master switch for inhibition of mitochondrial-driven apoptosis and necrosis (Supplementary Fig. 25b). Structure-based development led to the design of B-017, a cell-penetrable variant of the BNIP-3 peptide, which is a highly efficacious modulator of this axis with target specificity and a favourable safety profile and may thus be a treatment option for a range of pathological conditions.

## Methods

### Mice

All animal procedures were performed in accordance with institutional guidelines and approval from the local ethics committee in compliance with the European Convention for the Protection of Vertebrate Animals Used for Experimental and other Scientific Purposes (Directive 2010/63/EU; 84-02.04.2014.A144 and 81-02.04.2019.A369, approved by the Landesamt für Natur, Umwelt und Verbraucherschutz Nordrhein-Westfalen, Germany). Unless otherwise specified, male mice aged 12 ± 3 weeks with an average body weight of 30 g were used. C57BL/6 J wild-type mice were obtained from Janvier Labs (France). C57BL/6J-TgH (*Bnip3*^*−/−*^)^13^ and heterozygous crossed *Bnip3-3xFlag-knockin* mice (*Flag-Bnip3*^*ki/ki*^, *Flag-Bnip3*^*ki/+*^, *Flag-Bnip3* ^*−/−*^) were bred and maintained in the local animal house of the University Hospital Essen. All mice were housed under a 12-h light/dark cycle at a temperature of 20-24 °C and humidity of 40% to 60%, with ad libitum access to water and food. During the experiments, mice were monitored daily. At the end of the experiments, the mice were killed by overdosing them with anaesthetic (isoflurane) followed by cervical dislocation. *Bnip3-Flag-knockin* mice in a C57BL/6 J background were generated with the Biocytogen Extreme Genome Editing System (EGE®), a CRISPR/Cas9-based technology (Biocytogen Boston Corp., USA). The design was based on transcript_201 (NM_009760.4, NP_033890.1). To express 3xFlag using the endogenous promotor, the 3xFlag cassette was introduced after the start codon. Positive pups were identified via Southern blotting. PCR was employed for screening using 2 primers (5’-TTGTCCCTCAGTCCAGTGTCGCCTG-3’, 5’-GCCAAGGGCCTGAAATCTGAGCTAC-3’) to generate a 434-bp wild-type allele product and a 545-bp 3xFlag-Bnip3 allele product under the following conditions: 95 °C for 3 min; 95 °C for 15 s, 62 °C for 20 s, 72 °C 1 kb min^−1^, (30 cycles); 72 °C for 7 min. Male and female mice of the F5 generation were used for experiments. Proteins from heart homogenates and pulldown experiments were separated by Bolt 4–12% Bis-Tris Plus Gels (Invitrogen) and transferred onto nitrocellulose membrane (ThermoFisher Scientific). For protein detection, the HRP-labelled mouse monoclonal anti-DYKDDDDK Tag (Flag) antibody (clone 5A8E5, 1:1,000, GenScript, A01428) and the fluorescently labelled monoclonal anti-BNIP3 antibody (clone 1C8, 200 ng ml^−1^, Biocytogen) were used. The membranes were visualised on an Odyssey Imaging System (LI-COR). The pulldown of 3xFlag-BNIP3 from mouse heart lysates was performed with DYKDDDDK Magnetic Agarose (Pierce), according to the manufacturer’s instructions. For Western Blot analysis, the proteins were eluted by boiling in SDS-PAGE sample buffer at 95 °C for 5 min. The magnetic agarose was collected with a magnetic stand.

### Pigs

The myocardial ischaemia/reperfusion injury experiments on naïve Domestic Yorkshire crossbred swines (farm pigs, male, 12–13.5 weeks old, body weight ranging from 37.5 to 40.5 kg) were performed by Charles River Laboratories, Inc. (USA, Testing facility study No. 2865-001) in accordance with The Animal Welfare Act (9 CFR Parts 1, 2, and 3) of the US Department of Agriculture (USDA), and the Guide for the Care and Use of Laboratory Animals, Institute of Laboratory Animal Resources, National Academy Press, Washington, DC, 2011. The protocol and any amendments or procedures involving the care or use of pigs were reviewed and approved by the Testing Facility Institutional Animal Care and Use Committee before the initiation of the study. The animals were individually housed in stainless steel runs with raised flooring. Water was available via an automatic watering system ad libitum. Fluorescent lighting was provided for ~12 h per day. The dark cycle was interrupted intermittently due to study-related activities. Animal welfare, room temperature and humidity were continuously monitored, documented, and maintained to the maximum extent possible within the ranges of 69 °F to 79 °F and 30–70%, respectively. At the end of the experiments, the pigs will not be recovered from anaesthesia and will be euthanised with an injection of sodium pentobarbital intravenously.

### Rats

The liver transplant experiments were assessed on isolated livers, which were excised from Wistar rats (male, body weight ranging from 250 g to 300 g, 8-9 weeks old). Animals were housed in conventional type IV cages. They were equipped with nesting material, gnawing sticks and cardboard tubes. The light/dark cycle was set at 12 h:12 h. The procedure of killing with intracardial injection of potassium chloride in deep isoflurane anaesthesia for organ retrieval according to § 4 Abs. 3 TSG (German Legislation on animal protection) has been approved by the animal welfare officers of the University Hospital Essen. The federal law regulating the protection of animals and the principles of laboratory animal care (NIH publication vol 25, No 28, revised 1996) were respected.

The toxicological studies were carried out on female and male rats (Wistar Han, Beijing Vital River Laboratory Animal Technology Co., Ltd.). Rats aged ~8 weeks with an average body weight of 153.83 to 194.31 g (females) and 239.01 to 284.03 g (males) were used. Females were nulliparous and non-pregnant. Animals were housed in groups (up to 3 animals of the same sex and same dosing group) in solid-bottom cages with corncob bedding, and a water bottle was provided. The room was controlled and monitored for humidity (range 44.4% to 78.1%) and temperature (range 22.3 °C to 24.6 °C). The room was on a 12-hour light/dark cycle, except when interrupted by study activities. During the experiments, animal welfare was monitored daily. At the end of experiments, the rats were anaesthetised by isoflurane and euthanised by exsanguination. The protocol and any amendments or procedures involving the care or use of animals in this study were reviewed and approved by the WuXi AppTec Institutional Animal Care and Use Committee (IACUC) prior to initiation of such procedures. A staff veterinarian monitored the study for animal welfare issues. All applicable portions of the study conformed to the AAALAC International guidelines as reported in the Guide for the Care and Use of Laboratory Animals, National Research Council (2011) and The People’s Republic of China, Ministry of Science & Technology, “Regulations for the Administration of Affairs Concerning Experimental Animals”, 2017.

### Dogs

Cardiovascular effects from the B-017 peptide were evaluated in dogs (conscious Beagle dogs, male, body weight ranging from 9.2 to 10.1 kg, 27–28 weeks old) and performed by Labcorp Early Development Laboratories Ltd. (UK, Labcorp study No. 8453010). All procedures were conducted in accordance with Good Laboratory Practice Regulations 1999, Statutory Instrument 1999 No. 3106 from the United Kingdom Good Laboratory Practice Monitoring Authority. Medicines and Healthcare Products Regulatory Agency (MI-IRA) and Principles on Good Laboratory Practice. ENV/MC/CHEM (98) 17 (revised in 1997, issued January 1998) from the Organisation for Economic Co-Operation and Development (OECD). The study was approved by the Labcorp Animal Welfare and Ethical Review Body (AWERB), Harrogate, United Kingdom. Animals were received from Marshall Bioresource, UK, and were individually housed in a single, exclusive room. They were housed in pens that conform to the Code of Practice for the Housing and Care of Animals Bred, Supplied or Used for Scientific Purposes. Bedding was provided on a daily basis to each cage by Clan Lignocel. Each batch of bedding was analysed for specific contaminations. Main supply water was provided ad libitum via an automatic watering system. Rooms were air-conditioned to provide a minimum of 15 air changes/hour. The temperature was maintained in the specified range of 13 to 23 °C. Fluorescent lighting was controlled automatically to give a cycle of 12 hours of light and 12 hours of dark. At the end of the experiments, animals will be anaesthetised with sodium pentobarbital and exsanguinated.

### Cell lines and isolated cells

Human iPSC-derived ventricular cardiomyocytes (Ax 2505) were obtained from Axol Bioscience, UK, human cardiomyocytes (HCM) from two different donors (C-12810) and HCF (C-12375) from PromoCell, Germany, and mouse embryonic fibroblasts (MEFs: wild-type SV40, ATCC-CRL-2907; *Bcl-2* KO SV40, ATCC-CRL-2908, *Bax* KO SV40, ATCC-CRL-2910, *Bak* KO SV40, ATCCF-CRL-2912, *Bax Bak* KO SV40, ATCC-CRL-2913) from ATCC, USA. All cell lines were cultured according to the manufacturer’s instructions. The absence of BAX in *Bak* KO MEFs and BAK in *Bax* KO MEFs was validated using the anti-BAX antibody (1:1000, C2772, Cell Signaling; anti-rabbit HRP, 1:20,000) and the anti-BAK1 antibody (1:1000, 12105, Cell Signaling). The membranes were incubated for 1 h at room temperature with HRP-conjugated goat anti-rabbit secondary antibody (1:20,000, Cell Signaling 2729) and exposed to Western Sure Pre-Stained Protein Ladder (LI-COR) as instructed by the manufacturer. The membranes were visualised on an Odyssey Imaging System (LI-COR).

HepG2, HEK293 and MCF-7 cell lines were provided by A. Schramm (West German Cancer Center, University Hospital Essen, Germany). HepG2 and HEK293 cells were cultured in DMEM and MCF-7 cells in RPMI supplemented with 10% FCS and 100 IU/ml penicillin and 100 μg ml^−1^ streptomycin, respectively. All cell lines were authenticated by the Leibniz-Institute DSMZ GmbH, Germany, by carrying out DNA profiling using 17 different and highly polymorphic short tandem repeat (STR) loci and testing for the presence of mitochondrial DNA sequences from rodent cells, such as mouse, rat, Chinese and Syrian Hamster. In addition, the mouse embryonic fibroblast cell lines have been subjected to the procedure of Cytochrome C Subunit I DNA barcoding for identification of the species.

For adult cardiomyocytes and fibroblasts isolation^75^, C57BL/6 J wild-type, *Bnip3*^*−/−*^, *Flag-Bnip3*^*ki/ki*^ and *Flag-Bnip3* ^*−/−*^ mice were killed by cervical dislocation. Hearts were perfused with EDTA buffer (130 mM NaCl, 5 mM KCl, 0.5 mM NaH_2_PO_4_, 10 mM HEPES, 10 mM glucose-monohydrate, 10 mM taurine and 5 mM EDTA, pH 7.8) for 2 min inside the animal and then quickly excised and perfused for an additional 6 min to clear all the blood from the hearts. Hearts were then perfused with perfusion buffer (130 mM NaCl, 5 mM KCl, 0.5 mM NaH_2_PO_4_, 10 mM HEPES, 10 mM glucose-monohydrate, 10 mM taurine and 1 mM MgCl_2_, pH 7.8) for 2 min. Digestion was accomplished by adding 4200 U Collagenase type II, 5200 U Collagenase type IV and 0.05 mg/mL Protease XIV to the perfusion buffer. Hearts were digested for 20 min at 37 °C. The cell suspension was filtered through a 100 µm cell strainer and allowed to settle by gravity for 10 min. The cell pellet was resuspended in perfusion buffer, which was mixed with culture medium in stepwise increasing ratios to increase the Ca^2+^ concentration. The supernatant was collected and incubated with magnetic anti-CD31 beads (130-097-418, Miltenyi Biotec) to separate cardiac endothelial cells from fibroblasts. Cardiac fibroblasts were centrifuged at 1,000×*g* for 10 min and plated on cell culture dishes. Cells were cultivated in DMEM/F12 supplemented with 10% FCS, 50 µg ml^−1^ gentamycin and 50 ng ml^−1^ amphotericin B. Cardiomyocytes were plated on cell culture dishes pre-coated with laminin and washed after 2 h to remove dead and non-adherent cells. The cells were cultured in modified medium 199 including Earl’s salts, 2 mM carnitine, 5 mM creatine and 5 mM taurine supplemented with 5% FCS, 50 µg ml^−1^ gentamicin and 50 ng ml^−1^ amphotericin B.

### Recombinant protein

The pTAT-HA-ß-Gal vector was provided by S.F. Dowdy (University of California, San Diego, USA)^76^. ß-galactosidase fused to the HIV-1 TAT protein transduction domain (GRKKRRQRRRPQ) was grown in Escherichia coli (BL21) and expressed with 100 mM IPTG for 48 h at 37 °C. The bacteria were resuspended in PBS (pH 8.0), followed by incubation with 1 mg ml^−1^ lysozyme for 1 h at 4 °C and sonication on ice. After centrifugation at 20,000 × *g* for 50 min at 4 °C, the supernatants were added to columns containing Ni-NTA. The proteins were eluted with 250 mM imidazole in phosphate buffer.

Recombinant mouse BAX, His-tagged (mBAX-His, RPB343Mu02), recombinant mouse BNIP3, 6xHis-tagged (mBNIP3-His, CSB-CF002766), and recombinant human BAX, GST-tagged (hBAX-GST, CSB-C, F002573HU(A4)e0) were obtained from Hölzel Diagnostika (Germany). Recombinant human untagged BNIP3 (RPJ545Hu01) was obtained from Cloud-Clone Corp. (TX, USA) and human BNIP3, GST-tagged (hBNIP3-GST, BNIP3-294H) from Creative Biomart (NY, USA). Recombinant human BIM (1325-BL-050) was obtained from RD Systems (MN, USA), recombinant human PUMA, His-tagged (PDEH100697) from Biomol (Germany), recombinant human BID (AS-617111) from AnaSpec (CA, USA), recombinant human BCL-2, His-tagged (HY-P7537) from MedChemExpress (NJ, USA) and GST-tagged (BCL2-1588HF) from Creative BioMart (NY, USA), recombinant human MCL-1, His-tagged (NBP2515100) from Novus Biologicals (Abingdon, UK).

### Mouse monoclonal anti-BNIP3 antibody

The mouse monoclonal antibody against BNIP3 by single B cell cloning technology using the Beacon Optofluidic system was generated by Biocytogen Boston Corp., USA. Five female SJL-Elite mice (at 8 weeks of age) were immunised with mouse BNIP3 recombinant protein for a total of five rounds of immunisation. At day 60, the best three mice with acceptable titre were sacrificed, and their plasma cells were isolated and enriched from the collected lymph nodes. Plasma cells secreting antigen-specific antibodies were screened by antigen bead assays for mouse BNIP3 binding. The unloaded single plasma cells were sequenced by reverse transcription and PCR to obtain the V region sequence of the heavy and light chains of the antibodies. Antigen-specific bindings were validated by ELISA of culture supernatant from transfected HEK293T cells. 30 positive clones were chosen for Western blot validation against cell lysate from wild-type (*Bnip3*^*+/+*^) or *Bnip3* knockout (*Bnip3*^*−/−*^) mice. Of these, five clones showed strong bands specific for mouse BNIP3 and were transfected into ExpiCHO cells to generate purified antibody. The clone 1C8 was selected for all experiments performed and was used unlabelled and labelled with IRDye 800 CW infra-red dye (LI-COR). Knockout validation to confirm the anti-BNIP3 antibody 1C8 was conducted in isolated *Bnip3*^*−/−*^ mouse cardiomyocytes and mouse heart cytosolic and cytoplasmic fractions. *Bnip3*^*+/+*^ and *Bnip3*^*−/−*^ mouse cardiomyocytes were isolated, fixed in 4% paraformaldehyde, and permeabilised for 10 min with PBS containing 0.5% Triton X-100. Cells were incubated with the anti-BNIP3 antibody 1C8 (1:200, custom-made) overnight at 4 °C. Staining was performed with an Alexa Fluor 488-conjugated goat anti-mouse secondary antibody (1:200, Invitrogen A-11001) and DAPI (Invitrogen, 1:5,000 dilution). Cells were visualised under a confocal laser scanning microscope (Leica SP8, Leica, Germany). To obtain the cytoplasm and cytosol, the hearts were washed in ice-cold homogenisation buffer (250 mM sucrose, 10 mM HEPES, and 1 mM EGTA, pH 7.4), homogenised in 2 ml buffer and centrifuged at 700 × *g* for 10 min at 4 °C to remove unbroken tissue and nuclei. The supernatant was centrifuged at 15,000 × *g* for 10 min at 4 °C to separate cytoplasm from mitochondria. Then, the supernatant was centrifuged at 100,000 × *g* for 30 min at 4 °C to obtain the cytosolic fraction. Protein concentrations were measured using a DC Protein Assay (Bio-Rad Laboratories Inc.). For immunoblot analysis using recombinant proteins, recombinant mBNIP3-His (200 nM) and recombinant mBAX-His (200 nM) were incubated. Samples were diluted in 4× LDS sample buffer and 10× reducing agent (Invitrogen) and boiled at 95 °C for 5 min and loaded onto Bolt 4–12% Bis-Tris Plus Gels (Invitrogen). Western blot analyses with 20 µg, 15 µg and/or 10 µg protein were performed on nitrocellulose membranes after blocking in TBS-T containing 2% ECL prime powder (Cytiva) using the anti-BNIP3 antibody 1C8 (200 ng ml^−1^). The membranes were incubated for 1 h at room temperature with HRP-conjugated goat anti-mouse secondary antibody (IgG2c, 1:100,000; Jackson Immuno, ab98722) and exposed to SuperSignal West Femto Plus Substrate (ThermoFisher Scientific) or Western Sure Pre-Stained Protein Ladder (LI-COR) as instructed by the manufacturer. The membranes were visualised on an Odyssey Imaging System (LI-COR).

### Peptide synthesis

L-amino acid peptides were synthesised by JPT Peptide Technologies (Germany) using solid-phase resin-based methodology and purified to >90% by HPLC (confirmed by mass spectrometry). The N-terminus of the peptides was capped with an acetyl group, and the C-terminus was capped with an amide group. The B-017 sequence, GRKKRRQRRRPQWVELHFFN, consisted of 12 amino acids from the HIV-1 protein transduction domain (PTD)_48–59_ at the N-terminus and eight amino acids 13–20 derived from BNIP3 containing the substitution S19F at the C terminus. The negative control peptide, GRKKRRQRRRPQWVELAASN, consisted of 12 amino acids from the HIV-1 PTD at the N-terminus and eight amino acids 13-20 derived from BNIP3 containing two substitutions, H17A and F18A, at the C-terminus. The BNIP3-Peptide_1-49_ consisted of the first 49 amino acids of mouse BNIP3. For in vivo, in vitro and cell culture experiments, peptides were dissolved in H_2_O and diluted with 0.9% sodium chloride. Wild-type BIM-SAHB (EIWIAQELRXIGDXFNAYYA), BIM-SAHB_a_ (EICIAQELRXIGDXFNAYYA), BIM-SAHB_b_ (EIWIAQELRXIGDXFNAYYC) were synthesised by JPT Peptide Technologies (Germany)^28^.

The peptides used in this study were assessed according to established community guidelines for chemical probes. They exhibit appropriate potency, selectivity, and cellular activity, and suitable controls were included to support target specificity.

### Interaction studies

#### Proximity ligation assay

To assess the close proximity of BNIP3 to BAX, BAK and BCL-2, as well as of BAX to BIM and BID proximity ligation assays were performed in wild-type MEFs, MEFs lacking BAX, BAK, or BCL-2, respectively, *Bnip3*^*+/+*^, *Bnip3*^*−/−*^, *Flag-Bnip3*^*ki/ki*^ and *Flag-Bnip3* ^*−/−*^ cardiomyocytes using Duolink In Situ Red Starter Kit Mouse according to the manufacturer’s protocol (Sigma-Aldrich, DUO92101-1KT) and monoclonal mouse anti-BNIP3 antibody (clone 1C8, 1:200, Biocytogen, custom made), polyclonal rabbit anti-BNIP3 antibody (PA5-11402, 1:100, Invitrogen), polyclonal rabbit anti-BAX antibody (1:100, Cell Signaling Technologies, sc20067), monoclonal rabbit anti-BAK antibody (clone D4E4, Cell Signaling Technologies, 12105), and monoclonal mouse anti-BCL-2 antibody (clone Bcl-2-100, 1:100, Invitrogen, 13-8800), anti-BIM antibody (MA5-14848, 1:200, Invitrogen), and anti-BID (MA5-17034, 1:100, Invitrogen) antibody followed by visualisation using confocal laser microscopy (Leica SP8; Leica, Germany). For stimulation, staurosporine (2 µM) was used.

#### Peptide microarrays

For protein–protein interaction studies, peptide libraries were synthesised and immobilised on microarray slides: (i) the BNIP3/BAX interaction study; (ii) the BNIP3/BNIP3 interaction study, in which C-, N- or C/N-terminal truncations of the wild-type sequence of BNIP3 1–20 were performed; (iii) the BNIP3/BNIP3 interaction study, in which single residues of the wild-type sequence of BNIP3 1–20 were exchanged for 20 natural amino acids; and (iv) the BNIP3/octamer and BAX/octamer interaction studies, in which single residue of wild-type sequence of BNIP3 13–20 were exchanged for 20 natural amino acids. Recombinant mBNIP3-M and recombinant mBAX-His were used at concentrations of 5 µg ml^−1^ and 1 µg ml^−1^. The studies were performed by JPT Peptide Technologies (Berlin, Germany). For fluorescence-labelling, the DyLight Microscale Antibody Labelling Kit (ThermoFisher Scientific) with label DyLight 650 was used. The assay was performed using the automated Tecan HS 4800 microarray processing station. The microarrays were incubated with customer-provided samples diluted in blocking buffer for 2 h at 30 °C. Before each step, the microarrays were washed with washing buffer. The microarrays were scanned using a high-resolution fluorescence scanner. The laser settings and applied resolution were identical for all performed measurements. The resulting images were analysed and quantified using GenePix spot-recognition software (Molecular Devices). The mean signal intensity was extracted (between 0 and 65535 arbitrary units) for each spot. For further data evaluation, the so called MMC2 values were determined. The MMC2 equals the mean value of all three instances on the microarray except for when the coefficient of variation (CV) — the standard deviation divided by the mean value — is >0.5. In this case, the mean of the two closest values (MC2) was assigned to MMC2.

#### Circular dichroism (CD) spectroscopy

CD spectra were recorded on a Jasco J-170 CD spectrometer in a 1 mm quartz cuvette at 37 °C. For the spectrum of BNIP3 and BAX (0.67 µM in 10 mM Tris∙HCl, 1 mM EDTA, pH 8.0), 100 scans were averaged. For the spectrum of B-017 (100 µM in H_2_O), 10 scans were averaged. The secondary structure composition was analysed using DichroWeb, yielding the percentages of α-helices, β-sheets and random coil (http://dichroweb.cryst.bbk.ac.uk/html/home.shtml)^77,78^.

#### BNIP3 3D structure modelling

The structure of BNIP3 was modelled using Modeller 9.15^29^. The template structures corresponded to PDB codes 2K7W^28^ and 2KA1^26^. The created model was energy minimised using NAMD2.9^30^ and the CHARMM36 force field^31^. The AlphaFold model of BNIP3 and the complex of BAX with B-017 were modelled with AlphaFold2 on the ColabFold v1.5.5 server (https://colab.research.google.com/github/sokrypton/ColabFold/blob/main/AlphaFold2.ipynb^79^. The RosettaFold model of BNIP3 was modelled with RoseTTAFold on the Robetta server (https://robetta.bakerlab.org/)^79^.

#### Protein function prediction

For prediction of a functional binding domain in BNIP3, the Graph Convolutional Network DeepFRI server (https://beta.deepfri.flatironinstitute.org/workspace/3HDE54/predictions/QYTKE5)^35^ and the PredictProtein web server (https://predictprotein.org) were used.

#### Docking simulations

Docking simulation on BNIP3 (3D structure modelled with Modeller 9.15) and BAX (PDB code 2K7W, model 1) using HDOCK was performed by Profacgen (USA)^32^. Docking simulations on BAX (PDB code 4S0O) with TAT-WVELHFSN (natural BNIP3 sequence_13–20_) and B-017 were performed using HADDOCK^80^. Docking simulation on BNIP3 (3D structure modelled with Modeller 9.15) and BAK1 (PDB code 2JCN), and BAX (PDB code 2K7W, model 1) with BNIP3-Peptide_1-49_ were performed using HDOCK.

#### Fluorescence anisotropy

Fluorescence anisotropy measurements were carried out on a JASCO FP-8300 fluorescence spectrometer at 25 °C. Cy5.5-labelled B-017 (100 nM in 50 mM Tris, 10 mM Glutathione, pH 8.0) was titrated with human BNIP3, BCL-2 and glutathione-S-transferase and the fluorescence anisotropy r (*l*_ex_ = 680 nm, *l*_em_ = 695 nm) was measured for each step. All titrations were performed in *n* = 3 technical replicates, and data points are shown as means ± standard deviation based on the three experiments. Binding curves of the averaged data were fitted with GraphPad Prism 10.0 (GraphPad) using the quadratic binding equation for a one-site specific binding model, and K_D_ values are given as the fit of the averaged data points ± standard deviation of the fit.

$$r={r}_{0}+{{r}_{\max }\cdot \frac{\left(F+x+{K}_{D}\right)-\sqrt{{\left(F+x+{K}_{D}\right)}^{2}-4\cdot x\cdot F}}{2\cdot F}}_{(1)}$$

with *r* = anisotropy, *r*_0_ = anisotropy without protein, *r*_max_ = maximum anisotropy, *F* = fluorescent peptide concentration (constant), *x* = titrant protein concentration, *K*_D_ = dissociation constant.

#### Microscale thermophoresis (MST)

MST measurements were performed on a Monolith NT.115 instrument equipped with green/red filters (NanoTemper Technologies, Munich, Germany). MST and LED power were set to 20% and 35%, respectively, and all experiments were conducted at 37 °C. MST traces were recorded for 40 s (laser-off: 5 s, laser-on: 30 s, laser-off: 5 s). A 400 nM stock solution of Cy5.5-labelled peptide was prepared in MST buffer (50 mM Tris-HCl, pH 7.4, 150 mM NaCl, 10 mM MgCl₂). For binding analysis, serial dilutions of recombinant human BAX (Abnova, Heidelberg, Germany, cat#H00000581-P01), human His-tagged BCL-2 (MedChemExpress, New Jersey, USA, cat# HY-P7537), and His-tagged human MCL-1 (Novus Biologicals, Abingdon, United Kingdom, cat# NBP2515100) ranging from 12.2 to 25,000 nM were prepared in MST buffer containing 0.2% Tween-20. These were mixed 1:1 with Cy5.5-labelled peptide (final concentration: 200 nM), incubated for 10 min, and loaded into MST capillaries (NanoTemper Technologies, cat# MO-K022). Data points and MST traces were analysed at an MST-on time of 1.5 s using MO. Affinity Analysis v2.2.4 (NanoTemper Technologies). Binding affinities (*K*_D_) were calculated by nonlinear regression assuming a one-site binding model in GraphPad Prism v10.6.1 (GraphPad Software Inc., Boston, USA).

#### Crosslinking (DSSO, BpA)

Intramolecular chemical crosslinking of BNIP3:25 µM BAX and 10 µM BNIP3 were cross-linked with a 50-fold excess of DSSO (50 mM stock in DMSO) in PBS buffer (pH 7.4) at room temperature for 15 min. The reaction was quenched by adding 20 mM Tris∙HCl (pH 8.0) for 15 min at room temperature and submitted for crosslinking mass spectrometry (LC-MS). Furthermore, a sample of the crosslinking reaction was analysed on an SDS-PAGE gel. While BNIP3 showed a good number of intramolecular crosslinks, barely any crosslinks (even intramolecular ones) were found for BAX, likely due to its low number and unfavourable positioning of K residues.

Photo-crosslinking of B-017-BpA with BAX: 25 µM mouse BAX and 25 µM B-017-BpA with a C-terminal photoreactive Benzoyl-phenylalanine (BpA) residue in PBS buffer (pH 7,4) were irradiated at 365 nm for 1 h on ice and then submitted for crosslinking mass spectrometry (CL-MS). To visualise the crosslinked complex, which has a small mass difference compared to BAX alone, on an SDS-PAGE gel, the photo-crosslinking reaction was repeated with B-017-BpA that was labelled with an additional Cy5.5 fluorophore at its N-terminus. A fluorescence image of the gel was obtained on a BioRad GelDoc Imager prior to Coomassie staining.

#### Single-pot solid-phase-enhanced sample preparation (SP3)

Sample preparation for LC/MS/MS is based on the SP3 protocol^81^. 15 µg protein extracts were taken up in 100 µl 1× SP3 lysis buffer (final concentrations: 1% (wt/vol) SDS; 10 mM TCEP; 200 μl 40 mM chloracetamide; 250 mM HEPES pH 8) and heated for 5 min at 90 °C. Next, samples were cooled down to room temperature. 150 µg hydrophobic (#65152105050250) and 150 µg hydrophilic (#45152105050250) SeraMag Speed Beads (Cytiva) (bead to protein ratio 10 to 1) were added and gently mixed for 1 min. Then 100 µL 100% vol/vol Ethanol (EtOH) was added before incubation for 20 min at 24 °C, shaking vigorously (24 °C, 1300 rpm, Thermocycler C, Eppendorf). The beads were collected on a magnet, and the supernatant was aspirated. The beads were then washed four times with 180 µl 80 % EtOH (collection time on the magnet, minimum of 5 min). The beads were finally taken up in 100 µl 25 mM ammonium bicarbonate (ABC) containing 1 µg Trypsin (Protein: Trypsin ratio 30:1). To help bead dissociation, samples were incubated for 5 min in a sonification bath (preheated to 37 °C). Samples were incubated overnight, shaking vigorously (1300 rpm). The next day, samples were acidified with formic acid (FA, final 1% vol/vol) before collection on a magnet. The supernatants were transferred to a fresh Eppendorf tube before removing trace beads using a magnet for 5 min. The tryptic digests were then desalted on home-made C18 StageTips as described^82^. Briefly, peptides were immobilised and washed on a 2-disc C18 StageTip. Samples were then dried using a vacuum concentrator (Eppendorf), and the peptides were taken up in 0.1% formic acid solution (10 μl) and directly used for LC-MS/MS experiments.

#### LC-MS/MS

Experiments were performed on an Orbitrap Fusion Lumos (Thermo) that was coupled to a Vanquish Neo ultra-high-performance liquid chromatography (UHPLC) system (Thermo). The UHPLC was operated in the one-column mode. The analytical column was a fused silica capillary (75 µm × 28 cm) with an integrated frit emitter (CoAnn Technologies) packed in-house with Kinetex C18-XB core shell 1.7 µm resin (Phenomenex). The analytical column was encased by a column oven (Sonation) and attached to a nanospray flex ion source (Thermo). The column oven temperature was adjusted to 50 °C during data acquisition. The UHPLC was equipped with two mobile phases: solvent A (0.2% formic acid, FA, 99.9% H_2_O) and solvent B (0.2% formic acid, FA, 80% Acetonitrile, ACN, 19.8% H_2_O). All solvents were of UHPLC grade (Honeywell). Peptides were directly loaded onto the analytical column with a maximum flow rate that would not exceed the set pressure limit of 950 bar (usually ~0.4–0.6 µl min^−1^). Peptides were subsequently separated on the analytical column by running a 67 min gradient of solvent A and solvent B (start with 8% B; gradient 8% to 40% B for 50 min; gradient 50% to 100% B for 11 min gradient and 100% B for 6 min) at a flow rate of 250 ml min^−1^. The mass spectrometer was operated using Tune v3.5.3881.18. The mass spectrometer was set in the positive ion mode. Precursor ion scanning was performed in the Orbitrap analyser (FTMS; Fourier Transform Mass Spectrometry) in the scan range of m/z 380–1400 and at a resolution of 120,000 (at m/z 200) with the internal lock mass option turned on (lock mass was 445.120025 m/z, polysiloxane)^83^. Product ion spectra were recorded in a data-dependent fashion in the FTMS at resolution 30,000 ((m/z 200). The ionisation potential (spray voltage) was set to 2.2 kV. Peptides were analysed using a repeating cycle consisting of a full precursor ion scan (AGC standard; max acquisition time Auto) followed by a variable number of product ion scans (AGC 200% and acquisition time Auto) where peptides are isolated based on their intensity in the full survey scan (threshold of 50,000 counts) for tandem mass spectrum (MS2) generation that permits peptide sequencing and identification. Cycle time between MS1 scans was 3 s. Fragmentation was achieved by assisted Higher Energy Collision Dissociation (aHCD) (NCE 25, 30). During MS2 data acquisition, dynamic ion exclusion was set to 30 s and a repeat count of one. Ion injection time prediction, preview mode for the FTMS, monoisotopic precursor selection and charge state screening were enabled. Only charge states between +3 and +8 were considered for fragmentation.

#### Peptide and protein identification using MetaMorpheus

The raw data files for DSSO and BPA cross-linking experiments were directly processed in MetaMorpheus^84^ (v1.0.3.) using the CALIBRATION and the XL-SEARCH^85^ tasks. Briefly, the MS1 and MS2 spectra were recalibrated with CALIBRATION-task with the default settings. For DSSO experiments, the recalibrated mzML files were then searched in the XL-SEARCH task using DSSO_KSTY as crosslink type (specificity on one site is kept as K and the second site has KSTY). For experiments with B-017-BPA (photoreactive amino acid BPA added to C-term of peptide), BPA was added as a custom amino acid (Name: BPA; OneLetterAbbr.: x; MonoisotopicMass: 251.09462; ChemicalFormula: C16H13NO2) and BPA was set up as a crosslinker (Name: BPA; CrosslinkAminoAcid: x; Crosslinker Amino Acid 2: ACDEFGHIKLMNPQRSTVWYx; Crosslinker Total Mass: 0). The detailed settings for the CALIBRATION and XLSEARCH task can be found in Supplementary Data 1. All MS/MS spectra were searched against the sequences of interest concatenated to a contaminant database (249 entries for DSSO; 250 entries for BPA). FDR calculation is based on a target-Decoy approach, and the acceptance threshold is set to 0.01. The output files from MetaMorpheus were then converted to the ProXL Server^86^ format using the supplied converter and uploaded to ProXL for initial inspection of cross-links.

#### Overlay assays and immunoblotting

For protein/peptide overlay assays, 300 ng mBNIP3-His or mBAX-His was spotted on a nitrocellulose membrane. After 5 min of incubation followed by 5 min of washing with TBS-T, the membranes were incubated for 1.5 h with 50 μg B-017 labelled with Lys(5(6))-Fam. After washing two times for 5 min with TBS-T, fluorescence was detected using the ImageQuant system (Amersham).

Recombinant hBNIP3-GST (1 µg) and recombinant mBAX-GST (1 µg) were incubated with Cy5.5-labelled B-017 for 1 h. Western blot analyses using the iBLOT System (Invitrogen) were performed on nitrocellulose membranes after blocking in 1× Rotiblock (Carl Roth) in TBS-T using the fluorescence-labelled anti-BNIP3 antibody 1C8 (200 ng ml^−1^) and anti-BAX antibody (2D2, 1:1000, Cell Signaling Technologies) overnight. The membranes were incubated for 1 h at room temperature with goat anti-rabbit secondary antibody (CW800, 1:200,000; LI-COR) and exposed to Chameleon Duo Pre-stained Protein Ladder (LI-COR) as instructed by the manufacturer. The membranes were visualised on an Odyssey Imaging System (LI-COR).

#### Cellular uptake

For cellular uptake, wild-type MCF-7 (10,000 cells) were incubated with Cy5.5-labelled B-017 (4.4 µM) or Cy5.5-labelled negative control peptide for 1 h and visualised using the EVOS FL microscope (ThermoFisher Scientific).

#### Co-Immunoprecipitation

Co-immunoprecipitation was performed using Invitrogen^TM^ Dynabeads^TM^ M-270 Epoxy beads (ThermoFisher Scientific) bound to anti-BNIP3-antibody 1C8 or anti-BAX antibody (2D2, Cell Signaling Technologies) (according to the manufacturer’s instructions (Invitrogen^TM^ Dynabeads^TM^ Co-Immunoprecipitation Kit, ThermoFisher Scientific).

Recombinant mBAX-GST/hBAX-GST and recombinant mBNIP3-His/hBNIP3-untagged were incubated for 4 h at room temperature, then subjected to coupled Dynabead-antibodies for 1 h at 4 °C with rotation and processed following the manufacturer’s instructions. Proteins were eluted by boiling in Elution buffer at 95 °C for 10 min. Proteins were then analysed by Western blot on nitrocellulose membranes using anti-BNIP3 antibody 1C8 (200 ng ml^−1^), fluorescence-labelled anti-BNIP3 antibody 1C8 (200 ng ml^−1^) and fluorescence-labelled anti-BAX antibody (2D2 Alexa Fluor-790, 1:1000) (4 h at room temperature). Normal rabbit IgG (polyclonal antibody, 2729, Cell Signaling Technologies) served as control. The membranes were visualised on an Odyssey Imaging System (LI-COR).

Heart tissues isolated from FLAG-BNIP3 mice were initially washed with PBS, dissected, and homogenised in immunoprecipitation (IP) lysis buffer composed of 1× Cell Lysis Buffer (Cell Signaling), 100 mM PMSF (Roche), and 1× protease and phosphatase inhibitor cocktail (ThermoFisher). Homogenised lysates were incubated at 4 °C under constant rotation for 2 h. Following centrifugation, the clarified lysates were incubated with pre-washed streptavidin-conjugated paramagnetic beads (DYNAL™ Dynabeads™ M-280 Streptavidin; Invitrogen) for 3 h at 4 °C for preclearing. After a second centrifugation step, the resulting supernatant was incubated with mouse monoclonal anti-FLAG antibody (clone M2; Sigma-Aldrich) for 2 h at 4 °C on a rotary shaker. To capture BNIP3-BAX complexes, pre-washed streptavidin-conjugated beads were added to the mixture and incubated overnight at 4 °C with continuous rotation. Beads were then separated from the lysate using a magnetic stand (Invitrogen DynaMag 2 Magnet) and washed three times with ice-cold IP lysis buffer. The bound proteins were eluted by resuspending the beads in NuPAGE™ LDS sample buffer (Invitrogen) supplemented with 10 mM DTT and incubated at 95 °C for 15 min.

Eluted proteins were analysed by SDS-PAGE, using 11% SDS-polyacrylamide gels, and transferred onto PVDF membranes (ThermoFisher). Membranes were blocked with 5% BSA in PBS-Tween-20 for 1 h at room temperature and incubated overnight at 4 °C with one of the following primary antibodies: rabbit polyclonal anti-BAX (Cell Signaling Technology), rabbit polyclonal anti-BNIP3 (Cell Signaling Technology), or mouse monoclonal anti-FLAG (clone M2). After washing, membranes were incubated with either HRP-conjugated anti-rabbit IgG (Jackson Immuno Research) or anti-mouse IgG (R&D Systems) secondary antibodies. Protein detection was performed by chemiluminescence using the SuperSignal™ West Femto substrate (ThermoFisher) and visualised with an Odyssey® Fc Imager (LI-COR).

#### Detection of early and late events in BAX conformational activation

To assess the effect of BNIP3-Peptide_1-49_ and/or B-017 on the first activation event, recombinant hBAX-10xHis-tagged (400 nM) was incubated with staurosporine (2 µM), BNIP3-Peptide_1-49_ (5 µM), recombinant hPUMA (500 nM), or recombinant hBIM (500 nM) and B-017 (30 µM) or the negative control peptide, or recombinant hBCL_XL_ (500 nM), or BSA (5 µM) and Pierce Protein A/G magnetic beads-conjugated BAX 6A7 antibody for 1 h. Pierce Protein A/G magnetic beads-conjugated mouse IgG was used as control. After recovery of the immunoprecipitated proteins from beads, Western blot was performed using fluorescence-labelled anti-BAX antibody (2D2 Alexfluor-790, 1:1,000, Cell Signaling Technologies), an antibody that recognises total BAX and band intensity visualised on an Odyssey Imaging System (LI-COR).

To assess the effect of B-017 on the second activation event, the insertion of BAX into the mitochondrial outer membrane as a marker of BAX α-helix 9 exposure, recombinant hBAX-GST (200 nM) or recombinant hBAX, 10xHis-tagged was incubated with isolated C57BL/6 J mouse heart mitochondria (150 µg), staurosporine (2 µM), BNIP3-Peptide_1-49_ (5 µM), recombinant hPUMA (500 nM), recombinant hBIM (500 nM), BIM-SAHB_wt_ (1 µM), BIM-SAHB_a_ (1 µM) or BIM-SAHB_b_ (1 µM), and B-017 (4, 8, 12, 30 µg) or negative control peptide (30 µM) for 2 h at room temperature. In a second approach, MCF-7 cells (3 × 10^6^ cells) and wild-type MEFs (3 × 10^6^ cells) were treated with 2 µM staurosporine and 146 µM/160 µM B-017 or a negative control peptide for 4 h. BAX insertion was assessed using alkaline treatment of isolated mitochondria that separate loosely attached BAX^38^. Isolated mitochondria were incubated with 0.1 M Na_2_CO_3_ (pH 10.5) for 20 min on ice or with an equal volume of water as a negative control. Supernatant and lysed mitochondria were separated by centrifugation at 20,000 × *g* for 45 min. These fractions were analysed by Western blot for BAX using anti-BAX antibody (2D2, 1:1000) and HRP-conjugated secondary antibody (1:20,000). The membranes were visualised on an Odyssey Imaging System (LI-COR).

### Cell viability and mitochondrial health

#### Cell viability test using the xCELLigence real-time cell analyser

To determine the cellular response to B-017 treatment in different cell lines exposed to stimulation of cell death signalling, the cellular impedance was measured in 96-well plates equipped with gold microelectrodes using the xCELLigence Real Time Cell Analyser Cardio system (Agilent Technologies, USA, OLS, Bremen, Germany) placed in an incubator at 37 °C with 5% CO_2_. 100 µl of medium was added to each well of the E-plate 96 (OLS, Bremen, Germany) to measure the background impedance of the cell culture medium. HEK293 cells (8000 cells per well), HCF (2000 cells per well), HepG2 cells (12,000 cells per well), MCF-7 cells (8000 cells per well), *Bax*^*−/−*^, *Bak*^*−/−*^ and *Bcl-2*^*−/−*^ MEFs (10,000 cells per well) were then seeded in the 96-well E-plates. After 30 min, during which the adhesive forces can develop, the E-plate was returned to the incubator. After reaching a cell index of 1, indicating a stable adhesion to the E-plate, the cells were simultaneously treated with staurosporine (HEK293 and HCF: 10 nM; HepG2, *Bax*^*−/−*^, *Bak*^*−/−*^ and *Bcl-2*^*−/−*^ MEFs: 500 nM; MCF-7: 100 nM) and B-017 (52 µM). The impedance signal was recorded every 5 min for up to 6 h, and the normalised cell index was calculated using the RTCA Cardio Software (Agilent Technologies, USA)^87^. The time point chosen to assess the differences was 1 h after treatment for HEK293 and HepG2 cells, 2 h for *Bax*^*−/−*^, *Bak*^*−/−*^ and *Bcl-2*^*−/−*^ MEFs and 4 h for HCF to exclude a proliferation effect.

#### Necrotic and apoptotic signalling

Different cell lines were treated simultaneously with staurosporine (STS) or doxorubicin (DOX) and B-017. After incubation, caspase 3/7 activity was measured in MCF-7 (15,000 cells per well, 2 µM STS, 52 µM B-017/negative control peptide, 4 h), mouse cardiac fibroblast (mCF; 15,000 cells per well, 2 µM STS, 52 µM B-017/negative control peptide, 4 h), wild-type MEFs (10,000 cells per well, 2 µM STS, 26 and 52 µM B-017/negative control peptide, 4 h), *Bax*^*−/−*^ MEFs (10,000 cells per well, 2 µM STS, 26 and 52 µM B-017/negative control peptide, 4 h), *Bcl-2*^*−/−*^ MEFs (10,000 cells per well, 2 µM STS, 26 and 52 µM B-017/negative control peptide, 4 h), and *Bak*^*−/−*^ MEFs (10,000 cells per well, 2 µM STS, 26 and 52 µM B-017/negative control peptide, 4 h) using the Caspase-Glo® M3/7Assay (Promega, G8090), according to the manufacturer’s instructions using the BMG FLUOstar Omega Microplate Reader.

Reactive oxygen species, primarily H_2_O_2,_ generation, was measured in HCM (7,500 cells per well, 10 µM DOX, 4.4 µM, B-017/negative control peptide, 4 h), HCF (80,000 cells per well, 500 nM STS, 52 µM B-017, 1 h), HEK293 (80,000 cells/ per well, 500 nM STS, 52 µM B-017, 1 h) and MCF-7 cells (80,000 cells per well, 500 nM STS, 52 µM B-017, 1 h) using the DCFDA/H_2_DCFDA–Cellular ROS Assay Kit (Abcam, ab113851), according to the manufacturer’s instructions. Fluorescence intensity of H_2_DCFDA was measured with the BMG FLUOstar Omega Microplate Reader or the EVOS FL microscope (Thermo Fisher Scientific) and processed in ImageJ2 software (NIH). H_2_O_2_ generation was further determined in MCF-7 cells (15,000 cells per well, 2 µM STS, 52 µM B-017, 1 h) using the Invitrogen CellROX Deep Red Reagent (ThermoFisher Scientific, C10422), according to the manufacturer’s instructions, using the BMG FLUOstar Omega Microplate Reader.

To analyse the mitochondrial inner membrane potential in human iPSC-derived cardiomyocytes (Axol 2505), the cells were incubated in HEPES buffer (113 mM NaCl, 4.7 mM KCl, 12 mM HEPES, 1.2 mM MgSO_4_, 30 mM taurine, and 1.3 mM CaCl_2_, pH 7.4) under 1% O_2_ at 37 °C for 2 h. For reoxygenation, the cells were incubated in HEPES buffer supplemented with 5.5 mM glucose under 21% O_2_ at 37 °C for 2 h. During hypoxia/reoxygenation the cardiomyocytes were treated with B-017 (4.4 µM) or the negative control peptide (4.4 µM). To determine the effect of B-017 on STS-induced changes in mitochondrial inner membrane potential, MCF-7 cells (15,000 cells per well) were incubated with 500 nM STS and 52 µM B-017 for 1 h. To analyse the effect of B-017 on doxorubicin-induced changes in mitochondrial inner membrane potential, human cardiomyocytes (7,500 cells per well HCM, PromoCell) were incubated with 10 µM DOX and 4.4 µM B-017 for 4 h. The iPSC-derived cardiomyocytes and MCF-7 cells were then incubated with 6 µM JC-1 dye in medium at 37 °C for 30 min and HCM with TMRM dye and processed according to the manufacturer’s instructions (JC-1 Kit, ThermoFisher Scientific, TMRM Kit, Abcam). The cells were analysed using an EVOS FL microscope (ThermoFisher Scientific) and processed in ImageJ2 software (NIH) and the BMG FLUOstar Omega Microplate Reader.

Transient opening of the mPTP, a pore in the mitochondrial inner membrane, was induced in HCM (7,500 cells per well) with 10 µM DOX. 4.4 µM B-017 was applied simultaneously. After 1 h incubation, the cells were processed with the Calcein Kit (Abcam) according to the manufacturer’s instructions using the BMG FLUOstar Omega Microplate Reader.

To assess basal mitochondrial calcium levels, HCM (50,000 cells per well) were incubated with 10 µM DOX and 4.4 µM B-017/negative control peptide for 1 h. The cell culture medium was removed, and the cells were washed three times with warm medium. Subsequently, the cultures were incubated with 1 µM Rhod-2 AM in medium for 30 min at 37 °C and 5% CO_2_. After three additional washes with medium, the coverslips were mounted onto slides using Immuno mount (Sigma-Aldrich). The Rhod-2 AM signal was then recorded using a fluorescence microscope (Leica DMi 8) or the BMG FLUOstar Omega Microplate Reader and processed in ImageJ2 software (NIH).

### In vivo and ex vivo ischaemia/reperfusion (I/R) models

#### In vivo mouse myocardial I/R

For in vivo myocardial I/R, the open-chest model was used^59,88^. Briefly, C57BL/6 J wild-type and *Bnip3*^*−/−*^ mice were anaesthetised with ketamine (100 mg kg^−1^ intraperitoneally, i.p.) and xylazine (10 mg kg^−1^ i.p.), intubated and ventilated. Deep anaesthesia was maintained with 1.2–2 Vol% isoflurane in conjunction with a gas mixture of 0.2 l min^−1^ O_2_ and 0.8 l min^−1^ compressed air. A lateral thoracotomy was performed, and the LCA was occluded. To determine the infarct size and the apoptotic and necrotic signalling pathway markers, the LCA was reopened after 30 min of occlusion for the desired reperfusion times. Buprenorphine (0.1 mg kg^−1^ s.c.) was injected for analgesia immediately after surgery and then every 8 h. To determine LV function, the LCA was occluded for 50 min. For analgesia, Buprenorphine (0.1 mg kg^−1^, s.c.) was injected 30 min before surgery and then every 4 h during the day and administered overnight (8 p.m. to 8 a.m.) with drinking water (0.009 mg ml^−1^) for a period of 3 days. Infarct size was assessed after 30 min of ischaemia followed by 24 h of reperfusion using Evans blue staining (1%) for delineation of the ischaemic area at risk (AAR) from the non-ischaemic zone (remote area) and 1% 2,3,5-triphenyl tetrazolium chloride (TTC) staining for demarcation of the viable and non-viable myocardium within the AAR, as described^19^. The infarct area, AAR, and non-ischaemic left ventricle were assessed with computer-assisted planimetry by an observer blinded to sample identity. The size of the myocardial infarction is expressed as a percentage of the AAR.

To assess the in vivo efficacy of B-017 on infarct size, mice were injected with sodium chloride (0.9% NaCl), TAT-ß-Gal, the negative control peptide (TAT-WVELAASN; 0.2 mg/kg in 50 µl 0.9% NaCl), or B-017 (TAT-WVELHFFN, 0.2 mg kg^−1^ BW, 0.4 mg kg^−1^ BW, 0.8 mg kg^−1^ BW, or 1.6 mg kg^−1^ BW in 50 µl 0.9% NaCl), respectively, into the LV cavity 5 min before reperfusion. To evaluate the inhibitory effects of B-017 on downstream signalling, mice were injected with B-017 (0.8 mg kg^−1^ BW in 50 µl 0.9% NaCl) or the negative control peptide (0.8 mg kg^−1^ BW in 50 µl 0.9% NaCl) into the LV cavity 5 min before reperfusion. Sham-operated mice and baseline values served as controls.

#### In vivo porcine myocardial I/R

For in vivo myocardial I/R, the closed-chest model was used. Naïve Domestic Yorkshire crossbred swine (farm pigs, male, body weight ranging from 37.5 to 40.5 kg) were placed in a dorsal recumbency, and the surgical sites were prepared with alternating wipes of chlorhexidine scrub and solution. The animals were connected to a defibrillator and an EKG machine and monitored thoroughly throughout the procedure. Following the induction of anaesthesia, a small incision was made over the femoral artery and vein; the vessels were isolated. A small opening was made in the artery, and a sheath was introduced. In addition, a sheath was placed in the femoral vein to allow for emergency drug administration, if necessary. An appropriate guide catheter was advanced into the ostium of the left anterior descending artery (LAD) using visual guidance, followed by fluoroscopy. Non-ionic contrast was used for all procedures. A balloon catheter was introduced by advancing it through the guide catheter to the LAD coronary artery. The balloon was advanced into the coronary arteries through a guide catheter to a suitable place above the first diagonal branch of the LAD. The balloon was then inflated to a pressure sufficient to ensure complete occlusion of the artery, and verified via fluoroscopy. Once verified, the balloon was left inflated in the artery for 60 min. Following the 60-min occlusion, TAT-WVELHFFN (0.075 mg kg^−1^) or sodium chloride (0.9% NaCl) was administered via intravenous bolus injection through the femoral vein, 5 min prior to reperfusion. At the end of the ischaemic period, the balloon was deflated, and the ischaemic area was allowed to reperfuse for 4 h. Complete balloon deflation was confirmed via fluoroscopy. At the end of the procedure, all catheters were removed, the artery and vein were ligated, and the incision was closed in a standard fashion. For infarct size assessment, the hearts were removed and flushed with heparinised lactated Ringer's solution until clear of blood. The LAD was tied off at the location of the balloon occlusion during the myocardial infarction procedure. Once tied off, the LAD, LCX, and RCA were cannulated. Evans blue dye was injected. The hearts were cut from base to apex into approximately 1 cm serial sections. Each section was weighed and photographed. The sections of the heart were stained with TTC for 30 min at 37 °C and photographed a second time. The infarct size was calculated as a percent of the AAR. The images (including a ruler) were analysed, and the ratios of infarct/AAR, infarct/left ventricle area (LV), and AAR/LV were calculated.

Logbook v5.3, NextDocs v.6.1, Provantis v9.4, SAS v9.4 were used for data collection and analyses.

#### In vivo mouse brain I/R

Focal cerebral ischaemia was induced by a 30-min tMCAO^89^. C57BL/6 wild-type mice were anaesthetised with 2% isoflurane in O_2_. A servo-controlled heating blanket was used to maintain a core body temperature close to 37 °C throughout surgery. After a midline neck incision, a standardised silicon rubber-coated No. 6.0 nylon monofilament (6023910PK10; Doccol) was inserted into the right common carotid artery and advanced via the internal carotid artery to occlude the origin of the MCA. After 30 min, the mice were re-anaesthetised, and the occluding filament was removed to allow reperfusion. Stroke volumes were assessed 24 h after tMCAO, based on TTC staining. The mice were then randomly assigned to the operation by an independent researcher who was not involved in the data analysis. Investigators involved in the surgery and evaluation of all readout parameters were blinded to the experimental groups. The mice were injected intravenously with sodium chloride or TAT-WVELHFFN (0.8 mg kg^−1^ in 50 µl 0.9% NaCl) immediately before ischaemia. Sham-operated mice served as controls.

#### Isolated liver perfusion

Livers were retrieved from male Wistar rats, 20 min after cardiac arrest induced by intracardial injection of potassium chloride during deep anaesthesia of the animal. After flush-out with and 18 h of cold storage in histidine tryptophan ketoglutarate (HTK) solution, livers were rinsed with 20 ml of saline solution at room temperature with or without the addition of B-017. 20 min of poikilothermic warming up on a petri dish simulated the period of surgical implantation. Eventually, the grafts were put on an established recirculating perfusion circuit to evaluate graft recovery^68^. Freshly prepared Williams E solution, supplemented with 3 g 100 ml^−1^ of bovine serum albumin, was pumped at 3 ml g^−1^ liver min^−1^ through the portal vein by means of a roller pump. The perfusate was oxygenated with a 95% O_2_–5% CO_2_ gas mixture and its temperature was kept at 37 °C by means of a circulating thermostat.

### Functional assessment in mice

#### Transthoracic echocardiography in mice

Echocardiography was used to measure LV systolic function in C57BL/6 J wild-type mice in response to B-017 treatment. Echocardiography was performed before, directly after and 5 h after intracardiac injection of B-017 (0.8 mg kg^−1^ BW in 50 µl 0.9% NaCl) using a Vevo 2100 Imaging System (FUJIFILM VisualSonics, Inc.). To evaluate LV function in C57BL/6 J wild-type mice in the setting of myocardial I/R injury, mice were exposed to 50 min of ischaemia, followed by reperfusion up to 28 days. The mice were injected with B-017 (0.8 mg kg^−1^ BW in 50 µl NaCl) or the negative control peptide (0.8 mg kg^−1^ BW in 50 µl NaCl) intracardially 5 min before reperfusion and intraperitoneally (1.6 mg kg^−1^ BW in 50 µl NaCl) on day 1, day 3, day 5 and day 7 post infarction. A second cohort were injected with B-017 (1.6 mg kg^−1^ BW in 50 µl NaCl) or the negative control peptide intraperitoneally (1.6 mg kg^−1^ BW in 50 µl NaCl) on day 1, day 3, day 5, and day 7 post infarction. Echocardiography was performed at baseline and before treatment on day 1, day 3, day 5, day 7, day 14, and day 28 post infarction using the Vevo 3100 Imaging System (FUJIFILM VisualSonics). The investigators were blinded with respect to the untreated and treated groups. After sedation with 2 Vol% isoflurane, the mice were placed on a heated plate with constant heart rate, respiratory rate and body temperature monitoring via a rectal probe. The LV ejection fraction was calculated using Simpson’s method. Speckle tracking-based strain analyses were performed using VevoLAB 5.9.0 software (FUJIFILM VisualSonics, Inc.). Sham-operated mice served as controls.

### Functional assessment in rats

#### Body weights

Each animal was weighed on days 7, 5, and 1 before the dosing phase started, and on days 4, 7, 11, and 14 during the dosing phase and the recovery phase.

#### Haematology, serum chemistry and urine analysis

At termination, blood samples for haematology and serum chemistry were obtained from the abdominal aorta at necropsy as a terminal procedure after isoflurane anaesthesia. Urine was collected overnight from animals housed in metabolism cages.

#### Toxicokinetics

On day 1 and day 14, blood samples were collected at 0 (predose), 0.083 (5 min), 0.5 (30 min), 1, 2, 6 and 24 h postdose. Approximately 0.2 mL of blood was collected from animals via the jugular. Blood was collected into appropriately labelled tubes containing K_2_EDTA as the anticoagulant. The tubes were gently inverted several times to ensure mixing and immediately placed on wet ice. Whole blood was centrifuged within 0.5 h of collection by centrifugation at 3200 × g and 4 °C for 10 min and plasma was obtained. Plasma was transferred into uniquely labelled clear polypropylene tubes with stabiliser (3.27% orthophosphoric acid in de-ionised water), mixed well. The volume ratio of plasma to stabiliser was 1:1, and the volume was adjusted according to the volume ratio. The plasma with stabiliser was frozen in the upright position immediately over dry ice and stored in a freezer set to maintain <−60 °C. Maximum plasma concentration (C_max_) and time to reach *C*_max_ (*T*_max_) were taken directly from the mean plasma concentration versus time profiles. The area under the curve (AUC_0–24h_) was calculated using the linear up/log down trapezoidal role. The initial plasma concentration (C_0_) and *T*_1/2_ were also calculated. Plasma concentrations that are below the lower limit of quantification (BLQ) were set to zero in the calculations of the mean concentration. However, when more than half ( > 50%) of the values at a single time point are BLQ, mean values were reported as BLQ. Mean plasma concentration below LLOQ (BLQ) was set to zero. AUC_0-24h_, C_max_, and C_0_ values were reported to 3 significant digits. *T*_max_ and *T*_1/2_ values were reported to one decimal place. AUC_0-24h_ and C_0_ ratios were reported to 2 significant digits and used to evaluate sex difference, dose proportionality and accumulation index. Generally, differences of more than 0.5-fold and less than 2-fold in toxicokinetic parameters per group were not considered to indicate a significant difference.

#### Histopathology

Tissues were trimmed and fixed in 10% neutral buffered formalin (NBF) except for the eyes with optic nerves, testes and epididymides, which were fixed in modified Davidson’s solution for 24–72 h. After fixation, these tissues were transferred to 10% NBF.

#### Haemodynamic monitoring in dogs

All analyses were performed by Labcorp Early Development Laboratories Ltd. (UK) in the Labcorp study No. 8453010. For assessing the effects of B-017 peptide on blood pressure and heart rate, 4 conscious telemetered male dogs were examined. The dogs used were surgically implanted intermuscularly with a telemetry device at Labcorp under an enabling Study plan (Labcorp Study No. YV97GG). The dogs received the treatments in 4 sessions with escalating B-017 peptide doses from 0 (0.9% sodium chloride), 1, 3 and 6 mg kg^−1^ intravenously, with appropriate intervals between the sessions. The dogs were acclimatised to the dosing and recording procedures. Then the telemetric recordings were taken whilst the dogs were in recording pens, to which they had been habituated in advance. Cardiovascular recordings were collected via DSI transceivers (TRX). A DSI™ digital data capture system linked with a DSI™ Ponemah data processing and analysis system was used. Telemetric recordings started ~2 h before the administration of the appropriate dose and continued for at least 24 h after the dosing procedure on each test session.

#### Assessment of neurological function

Global neurological deficits were quantified using the Bederson score (Supplementary Table 10). Neurological outcomes were assessed 24 h after the stroke.

### Necrotic and apoptotic signalling in mouse myocardial I/R injury

#### Troponin in serum

To examine necrotic cell death, blood was collected from the abdominal aorta after 24 h of reperfusion following 30 min of ischaemia. After 2 h at room temperature, the blood was centrifuged at 1000 × *g* for 20 min, and serum was collected. Troponin I levels were measured using a Troponin I Kit (Life Diagnostics, CTNI-1-US), according to the manufacturer’s instructions.

#### Mitochondrial ultrastructure and swelling

For transmission electron microscopy analysis of LV tissue, heart samples (1 mm^3^) were taken from the AAR at 10 min reperfusion^70^. Imaging of ultrathin heart sections (55 nm) was performed using a JEM 1400Plus equipped with LaB6 cathodes (JEOL, Ltd., Tokyo Akishima, Japan). Post-processing of TIFF image files acquired with a CMOS camera (TemCam-F416, TVIPS) was conducted with Fiji version 1.52p. The swelling of mitochondria isolated from the AAR at 10 min of reperfusion was measured by changes in light scattering at 540 nm. The AAR was excised from the heart and disrupted in 2 ml of ice-cold isolation buffer (250 mM sucrose, 10 mM HEPES, and 1 mM EGTA, pH 7.4) using a TissueRuptor^22^. The homogenate was centrifuged (700 × *g* for 10 min at 4 °C) to remove debris. Subsequently, the supernatant was centrifuged (15,000 × *g* for 10 min at 4 °C) to precipitate mitochondria. The mitochondria were washed twice with isolation buffer and centrifuged at 12,700 × *g* for 5 min at 4 °C. Finally, the mitochondria were resuspended in isolation buffer. Total protein concentration was measured using the DC Protein Assay (Bio-Rad Laboratories, Inc., Hercules, CA, USA) according to the manufacturer’s instructions. For the determination of mitochondrial swelling, 100 µg isolated mitochondria in 200 µl isolation buffer were used.

### Apoptosis

To detect apoptotic nuclei, TUNEL staining was performed using an ApopTag Red In situ Apoptosis Detection Kit (Millipore, S7165, Darmstadt, Germany) according to the manufacturer’s instructions. The total numbers of TUNEL-positive signal events per left ventricle (LV) were counted in four plane sections of the heart with three slices each (Ctrl. and B-017 group) and one each (Sham group), and calculated per 1 mm^2^ of LV tissue. Images were captured under an inverted epifluorescence microscope (Zeiss Axio Observer Z1, Oberkochen, Germany) and processed in ImageJ software 1.52a (NIH).

#### Mitochondrial BAX and cytochrome *c*

To determine the level of mitochondrial BAX and cytochrome *c*, isolated mitochondria were lysed in Mito-lysis buffer (200 mM sucrose, 10 mM HEPES, 1 mM EGTA, 1% Triton X-100, protease and phosphatase inhibitor, pH 7.4) and lysates were cleared by centrifugation at 20,000 × *g* for 15 min at 4 °C. Protein concentrations in the supernatants and the cytoplasmic fraction to determine the level of BAX cytochrome *c* were measured using a DC Protein Assay (Bio-Rad Laboratories Inc.). Then, samples were diluted in 4× LDS sample buffer and 10× reducing agent (Invitrogen) and boiled at 95 °C for 5 min and loaded onto Bolt 4–12% Bis-Tris Plus Gels (Invitrogen). Western blot analyses were performed on nitrocellulose membranes after blocking in TBS-T containing 5% milk, using the anti-BAX antibody (clone 2D2, Cell Signaling Technologies, 1:1000) and the anti-cytochrome *c* antibody (clone 7H8.2C12, Abcam, 1:1000 dilution, ab13575) and incubating overnight at 4 °C. The membrane was incubated for 1 h at room temperature with HRP-conjugated secondary antibodies from the appropriate species and exposed to SuperSignal West Pico Plus Substrate (ThermoFisher Scientific) as instructed by the manufacturer, with subsequent detection.

#### Caspase-3 activity

Caspase-3 activity was measured in the AAR after 1 h of reperfusion using a Caspase-3 Assay Kit (Abcam), according to the manufacturer’s instructions.

#### Adenosine triphosphate (ATP)

ATP levels were measured in the AAR after 1 h of reperfusion using the ATP Assay Kit ab 83355 – Fluorimetric (Abcam), according to the manufacturer’s instructions.

#### Metabolomics

For global metabolomic analysis by liquid chromatography-tandem mass spectrometry (LCMS/MS), heart fragments weighing 30–70 mg were homogenised using an electronic tissue disruptor (Qiagen) in ice-cold methanol. Metabolites were extracted using a two-step liquid method similar to Sellik et al.^90^. Internal standards (13C6-L-Arginine, 13C5-L-Valine, 13C2-Citric acid, 2H4-Succinic acid and 13C6-Fructose-6-phosphate) were added, followed by homogenisation, sonication, and centrifugation. Supernatants were collected, dried, and resuspended.

The chromatographic separation was performed using an Agilent 1290 Infinity II Bio LC system equipped with an AdvanceBio MS Spent Media column (150 × 2.1 mm, 2.7 μm). A gradient elution was implemented at a flow rate of 450 μl min^−1^, where solvent A was water containing 10 mM ammonium acetate (pH 9), and solvent B was a mixture of acetonitrile and water (95:5 v/v) with the same ammonium acetate concentration. The linear gradient profile was as follows: 0 min, 100% B; 0.5 min, 100% B; 6.5 min, 50% B; 7 min, 50% B; 7.01 min, 100% with 1.1 minutes equilibration between the runs. The column temperature was consistently maintained at 70 °C, with a sample injection volume of 1 μl.

Mass spectrometric detection was achieved by coupling the liquid chromatography system with a Thermo Orbitrap Q Exactive Plus mass spectrometer. The HESI II ion source was utilised for ionisation in both positive and negative modes. MS parameters were meticulously optimised, covering scan ranges of 70–1050 m z^−1^ with a resolution of 35,000 for full scans, and 17,500 resolution for data-dependent MS2 acquisitions.

LC-MS data were processed with MS-Dial 4.9.2, which facilitated compound identification through accurate mass measurements and MS2 spectra. For identification purposes, an in-house metabolite retention time library was utilised. The Compounds were subjected to statistical evaluation with MetaboAnalyst. This involved the application of Pareto scaling, alongside both univariate and multivariate statistical techniques, including principal component analysis (PCA) and partial least squares discriminant analysis (PLS-DA).

### Peptide uptake and distribution

For in vivo uptake and distribution, mice were injected intracardially with B-017-Lys(5(6)-FAM) 5 minutes before reperfusion. After 5 minutes of reperfusion, the mice were reperfused with 0.9% NaCl, then the organs were excised and homogenated. Fluorescence in plasma and mouse heart, liver, and spleen homogenates was detected using the BMG FLUOstar Omega Microplate Reader. For examination of the uptake in the brain, B-017-Lys(5(6)-FAM) was administered at the onset of ischaemia. After 30 min of ischaemia, the mice were reperfused, the brain excised and sectioned into 2 mm thick coronal slices using a brain matrix. These slices were fixed overnight in 4% PFA in TissueTek at −20 °C. Using a cryostat, 10 µm thick sections were cut. After repeated washings with PBS to remove the TissueTek, the sections were covered with Mowiol/DAPCO and examined microscopically.

### Light sheet fluorescence microscopy

For three-dimensional distribution analysis in mouse hearts, mice were injected with B-017-Cy5.5 or the negative control peptide-Cy.5.5 (1.6 mg kg^−1^ in 50 µl 0.9 NaCl) into the LV cavity 5 min before reperfusion. Tissue clearing: hearts were fixed for 4 h in 4% paraformaldehyde (w/v PFA) at 4 °C. Samples need to be protected from light in all the following steps. Next, hearts were dehydrated using an ascending ethanol series (50%, 70%, 100% (v/v) EtOH in ddH_2_O) while shaking: overnight in 50% EtOH at 4 °C, 30 min in 100% EtOH at room temperature, 30 min in 70% EtOH at room temperature. In order to enhance sample clarity, samples were bleached for 4 h at 4 °C using freshly prepared bleaching solution (5% (v/v) hydrogen peroxide, 5% (v/v) dimethyl sulfoxide in 100% ethanol). Afterwards, samples were washed three times in 100% ethanol for 30 min at 4 °C while shaking. For the final clearing step, hearts were warmed to room temperature for 5 min and subsequently transferred to a glass vial containing pure ethyl cinnamate (ECi, Sigma Aldrich). Samples were cleared for at least 4 h before imaging and were kept at room temperature and protected from light until and after imaging.

#### Light fluorescence microscopy and image processing

Samples were imaged using the Ultramicroscope II and ImSpector software (both LaVision BioTec). Cleared hearts were immersed in pure ECi in a quartz cuvette. Since B-017 and the negative control peptide were coupled with Cy5.5 fluorophores, the excitation wavelengths of the light sheets were set to 639 nm, and the corresponding 680/30 band-pass emission filter was used. Additionally, autofluorescence was imaged in the FITC channel (488 nm excitation, 525/50 band-pass emission filter). Samples were clamped in a sample holder, apex and aorta horizontally aligned, between two blocks of ECi-cleared phytagel (1% phytagel in H_2_O) to prevent deformation. The knot of the ligation, which is used to induce the myocardial I/R, remains in situ and needs to face downwards during imaging to reduce blockage of excitation or emission light. Data sets of whole hearts were obtained with ×1.2 total magnification with 10 μm z spacing between optical planes. The sheet width was set to 4200 and the numeric aperture to 0.148. The longest wavelength was imaged first, to prevent photobleaching. 16 bit OME.TIF stacks were converted (ImarisFileConverterx64, BitPlane) into Imaris files (.ims). 3D reconstruction and subsequent analysis were done using Imaris software (BitPlane).

### Statistical analyses

GraphPad Prism 9 software was used to perform statistical tests and to generate graphs. Data are presented as the means ± SD, and *P* values were calculated as detailed in the corresponding legends. Sample sizes were determined on the basis of previous experimental experience or based on general practices in the field. All replicates constitute biological replicates or independent experiments. For the in vivo studies, mice were randomly allocated to groups. In case of differences in fluorescence intensity within independent experiments due to different experimental time points on different days and cell passages used, the results were normalised to the vehicle/control group. Comparisons between characteristics of subject groups were analysed with a two-tailed Student’s *t* test. For comparisons between more than two groups, a one-way, repeated-measures ANOVA or two-way ANOVA with Tukey’s post hoc test, Bonferroni correction, or Sidák’s correction, respectively, was performed.

### Reporting summary

Further information on research design is available in the Nature Portfolio Reporting Summary linked to this article.

## Supplementary information

Supplementary Information

Description of Additional Supplementary Files

Supplementary Data 1

Supplementary Data 2

Supplementary Data 3

Supplementary Data 4

Supplementary Data 5

Supplementary Data 6

Reporting Summary

Transparent Peer Review file

## Source data

Source Data

## Data Availability

The mass spectrometry proteomics data for the crosslinking experiments have been deposited to the ProteomeXchange Consortium via the PRIDE partner repository with the dataset identifier PXD056758. All other data are available in the main text. The mass spectrometry metabolomics data have been deposited in the MetaboLights repository (https://www.ebi.ac.uk/metabolights/editor/MTBLS14417/overview). All other data are available in the main text. B-017 can be obtained from the corresponding authors under a Material Transfer Agreement. Source data are provided with this paper.
