# Supplementary material for: Reverse engineering of BNIP3 identifies a mitochondrial protective peptide
Source: Nat Commun. 2026 Jun 17;17:5359. doi: 10.1038/s41467-026-73993-2 (PMC13275919; doi:10.1038/s41467-026-73993-2)

Supplementary Information

Reverse engineering of BNIP3 identifies a mitochondrial protective peptide

Ulrike B. Hendgen-Cotta[1*#], Anna Roth[1], Christine Beuck[2], Daniel Messiha[1], Stephan Settelmeier[1], Shah Bahrullah Shah[1], Sebastian Korste[1], Kenny Bravo-Rodriguez[3], Mike Blueggel[2], Feyza Cansiz[4], Luiza Martins Nascentes Melo[4], Jonas Roesler[4], Sven W. Meckelmann[5], Oliver J. Schmitz[5], Farnusch Kaschani[6], Markus Kaiser[7], Sonja Esfeld[1], Omar El Bounkari[8], Jürgen Bernhagen[8,9,10], Sophie Brameyer[11], Kirsten Jung[11], Linda-Isabell Schmitt[12], Markus Leo[12], Tim Hagenacker[12], Matthias Totzeck[1], Thomas Minor[13], Michael Ehrmann[14], Alpaslan Tasdogan[4], Peter Bayer[2] & Tienush Rassaf[1*#]

[1]Department of Cardiology and Vascular Medicine, West German Heart and Vascular Center, Medical Faculty, University Duisburg-Essen, Hufelandstraße 55, 45147 Essen, Germany.

[2]Center of Medical Biotechnology, Research Group Structural and Medicinal Biochemistry, Faculty of Biology, University of Duisburg-Essen, Universitätsstraße 2, 45141, Essen, Germany.

[3]Department of Mechanistic Cell Biology, Max Planck Institute of Molecular Physiology, Otto-Hahn-Straße 11, 44227 Dortmund, Germany.

[4]Department of Dermatology, Medical Faculty, University Duisburg-Essen & German Cancer Consortium (DKTK), Partner Site, Hufelandstraße 55, 45147 Essen, Germany.

[5]Applied Analytical Chemistry, University of Duisburg-Essen, Universitätsstraße 2, 45141 Essen, Germany.

[6]Center of Medical Biotechnology, Analytics Core Facility Essen, Faculty of Biology, University of Duisburg-Essen, Universitätsstraße 2, 45141 Essen, Germany.

[7]Center od Medical Biotechnology, Chemical Biology, Faculty of Biology, University of Duisburg-Essen, Universitätsstraße 2, 45141 Essen, Germany.

[8]Division of Vascular Biology, Institute for Stroke and Dementia Research (ISD), LMU Klinikum, Ludwig Maximilian University (LMU) Munich, Munich, Feodor-Lynen-Str. 17, 81377 München, Germany.

[9]German Center for Cardiovascular Research (DZHK), partner site Munich Heart Alliance, Biedersteiner Str. 29, 80802 Munich, Germany.

[10]Munich Cluster for Systems Neurology (SyNergy), Feodor-Lynen-Str. 17, 81377 Munich, Germany.

[11]Faculty of Biology, Microbiology, Ludwig-Maximilians-University (LMU) Munich, Großhaderner Str. 2-4, 82152 Martinsried, Germany.

[12]Department auf Neurology and Center for Translational Neuro and Behavioral Science, Medical Faculty, University Hospital Essen, Hufelandstraße 55, 45147 Essen, Germany.

[13]Surgical Research Department, Medical Faculty, University Hospital Essen, Hufelandstraße 55, 45147 Essen, Germany.

[14]Center of Medical Biotechnology, Department of Microbiology, Faculty of Biology, University of Duisburg-Essen, Universitätsstraße 2, 45141 Essen, Germany.

*Corresponding authors. Email: ulrike.hendgen-cotta@uk-essen.de, tienush.rassaf@uk-essen.de.
#Both authors contributed equally to this work.

Table of Contents

Supplementary Tables

Supplementary Table 1
Supplementary Table 2
Supplementary Table 3
Supplementary Table 4
Supplementary Table 5
Supplementary Table 6
Supplementary Table 7
Supplementary Table 8
Supplementary Table 9
Supplementary Table 10

Supplementary Figures

Supplementary Figure 1
Supplementary Figure 2
Supplementary Figure 3
Supplementary Figure 4
Supplementary Figure 5
Supplementary Figure 6
Supplementary Figure 7
Supplementary Figure 8
Supplementary Figure 9
Supplementary Figure 10
Supplementary Figure 11
Supplementary Figure 12
Supplementary Figure 13
Supplementary Figure 14
Supplementary Figure 15
Supplementary Figure 16
Supplementary Figure 17
Supplementary Figure 18
Supplementary Figure 19
Supplementary Figure 20
Supplementary Figure 21
Supplementary Figure 22
Supplementary Figure 23
Supplementary Figure 24
Supplementary Figure 25

Source Data

Supplementary Figure 14a
Supplementary Figure 23c

Supplementary Table 1. BAX/BNIP3 Interface

Amino acids BAX	Amino acids BNIP3
74A	12P
74A	13P
74A	14P
75A	12P
75A	13P
75A	14P
75A	15P
78A	14P
78A	15P
78A	16P
78A	17P
78A	145P
79A	15P
82A	15P
82A	16P
106A	172P
106A	176P
159A	181P
159A	182P
160A	181P
163A	179P
163A	180P
163A	181P
163A	182P
163A	183P
164A	172P
164A	175P
164A	176P
164A	179P
164A	181P
165A	175P
165A	179P
166A	175P
166A	179P
167A	175P
168A	175P
169A	175P
172A	168P
173A	172P
173A	175P
175A	168P
176A	169P
176A	172P
186A	154P
189A	147P
189A	150P
190A	150P
191A	105P
191A	109P
191A	147P
191A	148P
191A	150P

Supplementary Table 2. BAX/BNIP3-Peptide$_{1-49}$ Interface

Amino acids BAX	Amino acids BNIP3 Peptide$_{1-49}$
34A	20A
35A	20A
36A	22A
37A	18A
37A	20A
37A	21A
37A	22A
38A	18A
38A	20A
38A	22A
38A	31A
38A	32A
38A	33A
39A	22A
40A	29A
77A	17A
78A	11A
78A	12A
78A	13A
78A	15A
78A	47A
81A	15A
81A	17A
82A	15A
119A	18A
122A	18A
122A	20A
123A	18A
123A	35A
123A	36A
123A	37A
125A	33A
126A	18A
126A	19A
126A	20A
126A	32A
126A	33A
126A	34A
126A	35A
127A	33A
127A	35A
128A	32A

Supplementary Table 3. BNIP3 peptides truncated

	Peptide sequences	
MSQSGEENLQGSWVELHFSN	N	M
QG	SN	MS
LQGS	FSN	MSQ
NLQGSW	HFSN	MSQS
ENLQGSWV	LHFSN	MSQSG
EENLQGSWVE	ELHFSN	MSQSGE
GEENLQGSWVEL	VELHFSN	MSQSGEE
SGEENLQGSWVELH	WVELHFSN	MSQSGEEN
QSGEENLQGSWVELHF	SWVELHFSN	MSQSGEENL
SQSGEENLQGSWVELHFS	GSWVELHFSN	MSQSGEENLQ
	QGSWVELHFSN	MSQSGEENLQG
	LQGSWVELHFSN	MSQSGEENLQGS
	NLQGSWVELHFSN	MSQSGEENLQGSW
	ENLQGSWVELHFSN	MSQSGEENLQGSWV
	EENLQGSWVELHFSN	MSQSGEENLQGSWVE
	GEENLQGSWVELHFSN	MSQSGEENLQGSWVEL
	SGEENLQGSWVELHFSN	MSQSGEENLQGSWVELH
	QSGEENLQGSWVELHFSN	MSQSGEENLQGSWVELHF
	SQSGEENLQGSWVELHFSN	MSQSGEENLQGSWVELHFS

Supplementary Table 4. BNIP3 peptides (20mer) substituted

Peptide sequences

MSQSGEENLQGSWVALHFSN	MSQSGERNLQGSWVELHFSN	MSQSKEENLQGSWVELHFSN	MSQSGEENCQGSWVELHFSN	MSQSGEENLQGSWVELHFSS
MSQSGEENLQGSWVCLHFSN	MSQSGESNLQGSWVELHFSN	MSQSLEENLQGSWVELHFSN	MSQSGEENDQGSWVELHFSN	MSQSGEENLQGSWVELHFST
MSQSGEENLQGSWVDLHFSN	MSQSGETNLQGSWVELHFSN	MSQSMEENLQGSWVELHFSN	MSQSGEENEQGSWVELHFSN	MSQSGEENLQGSWVELHFSV
MSQSGEENLQGSWVFLHFSN	MSQSGEVNLQGSWVELHFSN	MSQSNEENLQGSWVELHFSN	MSQSGEENFQGSWVELHFSN	MSQSGEENLQGSWVELHFSW
MSQSGEENLQGSWVGLHFSN	MSQSGEWNLQGSWVELHFSN	MSQSPEENLQGSWVELHFSN	MSQSGEENGQGSWVELHFSN	MSQSGEENLQGSWVELHFSY
MSQSGEENLQGSWVHLHFSN	MSQSGEYNLQGSWVELHFSN	MSQSQEENLQGSWVELHFSN	MSQSGEENHQGSWVELHFSN	MSQSGEEALQGSWVELHFSN
MSQSGEENLQGSWVILHFSN	MSQSGEENLQGSWVELHASN	MSQSREENLQGSWVELHFSN	MSQSGEENIQGSWVELHFSN	MSQSGEECLQGSWVELHFSN
MSQSGEENLQGSWVKLHFSN	MSQSGEENLQGSWVELHCSN	MSQSSEENLQGSWVELHFSN	MSQSGEENKQGSWVELHFSN	MSQSGEEDLQGSWVELHFSN
MSQSGEENLQGSWVLLHFSN	MSQSGEENLQGSWVELHDSN	MSQSTEENLQGSWVELHFSN	MSQSGEENMQGSWVELHFSN	MSQSGEEELQGSWVELHFSN
MSQSGEENLQGSWVMLHFSN	MSQSGEENLQGSWVELHESN	MSQSVEENLQGSWVELHFSN	MSQSGEENNQGSWVELHFSN	MSQSGEEFLQGSWVELHFSN
MSQSGEENLQGSWVNLHFSN	MSQSGEENLQGSWVELHGSN	MSQSWEENLQGSWVELHFSN	MSQSGEENPQGSWVELHFSN	MSQSGEEGLQGSWVELHFSN
MSQSGEENLQGSWVPLHFSN	MSQSGEENLQGSWVELHHSN	MSQSYEENLQGSWVELHFSN	MSQSGEENQQGSWVELHFSN	MSQSGEEHLQGSWVELHFSN
MSQSGEENLQGSWVQLHFSN	MSQSGEENLQGSWVELHISN	MSQSGEENLQGSWVELAFSN	MSQSGEENRQGSWVELHFSN	MSQSGEEILQGSWVELHFSN
MSQSGEENLQGSWVRLHFSN	MSQSGEENLQGSWVELHKSN	MSQSGEENLQGSWVELCFSN	MSQSGEENSQGSWVELHFSN	MSQSGEEKLQGSWVELHFSN
MSQSGEENLQGSWVSLHFSN	MSQSGEENLQGSWVELHLSN	MSQSGEENLQGSWVELDFSN	MSQSGEENTQGSWVELHFSN	MSQSGEELLQGSWVELHFSN
MSQSGEENLQGSWVTLHFSN	MSQSGEENLQGSWVELHMSN	MSQSGEENLQGSWVELEFSN	MSQSGEENVQGSWVELHFSN	MSQSGEEMLQGSWVELHFSN
MSQSGEENLQGSWVVLHFSN	MSQSGEENLQGSWVELHNSN	MSQSGEENLQGSWVELFFSN	MSQSGEENWQGSWVELHFSN	MSQSGEEPLQGSWVELHFSN
MSQSGEENLQGSWVWLHFSN	MSQSGEENLQGSWVELHPSN	MSQSGEENLQGSWVELGFSN	MSQSGEENYQGSWVELHFSN	MSQSGEEQLQGSWVELHFSN
MSQSGEENLQGSWVYLHFSN	MSQSGEENLQGSWVELHQSN	MSQSGEENLQGSWVELIFSN	ASQSGEENLQGSWVELHFSN	MSQSGEERLQGSWVELHFSN
MSQSGAENLQGSWVELHFSN	MSQSGEENLQGSWVELHRSN	MSQSGEENLQGSWVELKFSN	CSQSGEENLQGSWVELHFSN	MSQSGEESLQGSWVELHFSN
MSQSGCENLQGSWVELHFSN	MSQSGEENLQGSWVELHSSN	MSQSGEENLQGSWVELLFSN	DSQSGEENLQGSWVELHFSN	MSQSGEETLQGSWVELHFSN
MSQSGDENLQGSWVELHFSN	MSQSGEENLQGSWVELHTSN	MSQSGEENLQGSWVELMFSN	ESQSGEENLQGSWVELHFSN	MSQSGEEVLQGSWVELHFSN
MSQSGFENLQGSWVELHFSN	MSQSGEENLQGSWVELHVSN	MSQSGEENLQGSWVELNFSN	FSQSGEENLQGSWVELHFSN	MSQSGEEWLQGSWVELHFSN
MSQSGGENLQGSWVELHFSN	MSQSGEENLQGSWVELHWSN	MSQSGEENLQGSWVELPFSN	GSQSGEENLQGSWVELHFSN	MSQSGEEYLQGSWVELHFSN
MSQSGHENLQGSWVELHFSN	MSQSGEENLQGSWVELHYSN	MSQSGEENLQGSWVELQFSN	HSQSGEENLQGSWVELHFSN	MSQSGEENLAGSWVELHFSN
MSQSGIENLQGSWVELHFSN	MSQSGEENLQASWVELHFSN	MSQSGEENLQGSWVELRFSN	ISQSGEENLQGSWVELHFSN	MSQSGEENLCGSWVELHFSN
MSQSGKENLQGSWVELHFSN	MSQSGEENLQCSWVELHFSN	MSQSGEENLQGSWVELSFSN	KSQSGEENLQGSWVELHFSN	MSQSGEENLDGSWVELHFSN
MSQSGLENLQGSWVELHFSN	MSQSGEENLQDSWVELHFSN	MSQSGEENLQGSWVELTFSN	LSQSGEENLQGSWVELHFSN	MSQSGEENLEGSWVELHFSN
MSQSGMENLQGSWVELHFSN	MSQSGEENLQESWVELHFSN	MSQSGEENLQGSWVELVFSN	NSQSGEENLQGSWVELHFSN	MSQSGEENLFGSWVELHFSN
MSQSGNENLQGSWVELHFSN	MSQSGEENLQFSWVELHFSN	MSQSGEENLQGSWVELWFSN	PSQSGEENLQGSWVELHFSN	MSQSGEENLGGSWVELHFSN
MSQSGPENLQGSWVELHFSN	MSQSGEENLQHSWVELHFSN	MSQSGEENLQGSWVELYFSN	QSQSGEENLQGSWVELHFSN	MSQSGEENLHGSWVELHFSN
MSQSGQENLQGSWVELHFSN	MSQSGEENLQISWVELHFSN	MSQSGEENLQGSWVEAHFSN	RSQSGEENLQGSWVELHFSN	MSQSGEENLIGSWVELHFSN
MSQSGRENLQGSWVELHFSN	MSQSGEENLQKSWVELHFSN	MSQSGEENLQGSWVECHFSN	SSQSGEENLQGSWVELHFSN	MSQSGEENLKGSWVELHFSN
MSQSGSENLQGSWVELHFSN	MSQSGEENLQLSWVELHFSN	MSQSGEENLQGSWVEDHFSN	TSQSGEENLQGSWVELHFSN	MSQSGEENLLGSWVELHFSN
MSQSGTENLQGSWVELHFSN	MSQSGEENLQMSWVELHFSN	MSQSGEENLQGSWVEEHFSN	VSQSGEENLQGSWVELHFSN	MSQSGEENLMGSWVELHFSN
MSQSGVENLQGSWVELHFSN	MSQSGEENLQNSWVELHFSN	MSQSGEENLQGSWVEFHFSN	WSQSGEENLQGSWVELHFSN	MSQSGEENLNGSWVELHFSN
MSQSGWENLQGSWVELHFSN	MSQSGEENLQPSWVELHFSN	MSQSGEENLQGSWVEGHFSN	YSQSGEENLQGSWVELHFSN	MSQSGEENLPGSWVELHFSN
MSQSGYENLQGSWVELHFSN	MSQSGEENLQQSWVELHFSN	MSQSGEENLQGSWVEHHFSN	MSQSGEENLQGSWVELHFSA	MSQSGEENLRGSWVELHFSN
MSQSGEANLQGSWVELHFSN	MSQSGEENLQRSWVELHFSN	MSQSGEENLQGSWVEIHFSN	MSQSGEENLQGSWVELHFSC	MSQSGEENLSGSWVELHFSN
MSQSGECNLQGSWVELHFSN	MSQSGEENLQSSWVELHFSN	MSQSGEENLQGSWVEKHFSN	MSQSGEENLQGSWVELHFSD	MSQSGEENLTGSWVELHFSN
MSQSGEDNLQGSWVELHFSN	MSQSGEENLQTSWVELHFSN	MSQSGEENLQGSWVEMHFSN	MSQSGEENLQGSWVELHFSE	MSQSGEENLVGSWVELHFSN
MSQSGEFNLQGSWVELHFSN	MSQSGEENLQVSWVELHFSN	MSQSGEENLQGSWVENHFSN	MSQSGEENLQGSWVELHFSF	MSQSGEENLWGSWVELHFSN
MSQSGEGNLQGSWVELHFSN	MSQSGEENLQWSWVELHFSN	MSQSGEENLQGSWVEPHFSN	MSQSGEENLQGSWVELHFSG	MSQSGEENLYGSWVELHFSN
MSQSGEHNLQGSWVELHFSN	MSQSGEENLQYSWVELHFSN	MSQSGEENLQGSWVEQHFSN	MSQSGEENLQGSWVELHFSH	MSASGEENLQGSWVELHFSN
MSQSGEINLQGSWVELHFSN	MSQSAEENLQGSWVELHFSN	MSQSGEENLQGSWVERHFSN	MSQSGEENLQGSWVELHFSI	MSCSGEENLQGSWVELHFSN
MSQSGEKNLQGSWVELHFSN	MSQSCEENLQGSWVELHFSN	MSQSGEENLQGSWVESHFSN	MSQSGEENLQGSWVELHFSK	MSDSGEENLQGSWVELHFSN
MSQSGELNLQGSWVELHFSN	MSQSDEENLQGSWVELHFSN	MSQSGEENLQGSWVETHFSN	MSQSGEENLQGSWVELHFSL	MSESGEENLQGSWVELHFSN
MSQSGEMNLQGSWVELHFSN	MSQSEEENLQGSWVELHFSN	MSQSGEENLQGSWVEVHFSN	MSQSGEENLQGSWVELHFSM	MSFSGEENLQGSWVELHFSN
MSQSGENNLQGSWVELHFSN	MSQSFEENLQGSWVELHFSN	MSQSGEENLQGSWVEWHFSN	MSQSGEENLQGSWVELHFSP	MSGSGEENLQGSWVELHFSN
MSQSGEPNLQGSWVELHFSN	MSQSHEENLQGSWVELHFSN	MSQSGEENLQGSWVEYHFSN	MSQSGEENLQGSWVELHFSQ	MSHSGEENLQGSWVELHFSN
MSQSGEQNLQGSWVELHFSN	MSQSIEENLQGSWVELHFSN	MSQSGEENAQGSWVELHFSN	MSQSGEENLQGSWVELHFSR	MSISGEENLQGSWVELHFSN
MSKSGEENLQGSWVELHFSN	MSQSGEENLQGTWVELHFSN	MDQSGEENLQGSWVELHFSN	MSQKGEENLQGSWVELHFSN	MSQSGEENLQGSWRELHFSN
MSLSGEENLQGSWVELHFSN	MSQSGEENLQGVWVELHFSN	MEQSGEENLQGSWVELHFSN	MSQLGEENLQGSWVELHFSN	MSQSGEENLQGSWSELHFSN
MSMSGEENLQGSWVELHFSN	MSQSGEENLQGWWVELHFSN	MFQSGEENLQGSWVELHFSN	MSQMGEENLQGSWVELHFSN	MSQSGEENLQGSWTELHFSN
MSNSGEENLQGSWVELHFSN	MSQSGEENLQGYWVELHFSN	MGQSGEENLQGSWVELHFSN	MSQNGEENLQGSWVELHFSN	MSQSGEENLQGSWWELHFSN
MSPSGEENLQGSWVELHFSN	MSQSGEENLQGSWVELHFCN	MHQSGEENLQGSWVELHFSN	MSQPGEENLQGSWVELHFSN	MSQSGEENLQGSWYELHFSN
MSRSGEENLQGSWVELHFSN	MSQSGEENLQGSWVELHFDN	MIQSGEENLQGSWVELHFSN	MSQQGEENLQGSWVELHFSN	MSQSGEENLQGSAVELHFSN
MSSSGEENLQGSWVELHFSN	MSQSGEENLQGSWVELHFEN	MKQSGEENLQGSWVELHFSN	MSQRGEENLQGSWVELHFSN	MSQSGEENLQGSCVELHFSN
MSVSGEENLQGSWVELHFSN	MSQSGEENLQGSWVELHFFN	MLQSGEENLQGSWVELHFSN	MSQTGEENLQGSWVELHFSN	MSQSGEENLQGSDVELHFSN
MSWSGEENLQGSWVELHFSN	MSQSGEENLQGSWVELHFGN	MMQSGEENLQGSWVELHFSN	MSQVGEENLQGSWVELHFSN	MSQSGEENLQGSEVELHFSN
MSYSGEENLQGSWVELHFSN	MSQSGEENLQGSWVELHFHN	MNQSGEENLQGSWVELHFSN	MSQWGEENLQGSWVELHFSN	MSQSGEENLQGSFVELHFSN
MSQSGEENLQGAWVELHFSN	MSQSGEENLQGSWVELHFIN	MPQSGEENLQGSWVELHFSN	MSQYGEENLQGSWVELHFSN	MSQSGEENLQGSGVELHFSN
MSQSGEENLQGCWVELHFSN	MSQSGEENLQGSWVELHFKN	MQQSGEENLQGSWVELHFSN	MSQSGEENLQGSWAELHFSN	MSQSGEENLQGSHVELHFSN
MSQSGEENLQGDWVELHFSN	MSQSGEENLQGSWVELHFLN	MRQSGEENLQGSWVELHFSN	MSQSGEENLQGSWCELHFSN	MSQSGEENLQGSIVELHFSN
MSQSGEENLQGEWVELHFSN	MSQSGEENLQGSWVELHFMN	MTQSGEENLQGSWVELHFSN	MSQSGEENLQGSWDELHFSN	MSQSGEENLQGSKVELHFSN
MSQSGEENLQGFWVELHFSN	MSQSGEENLQGSWVELHFNN	MVQSGEENLQGSWVELHFSN	MSQSGEENLQGSWEELHFSN	MSQSGEENLQGSLVELHFSN
MSQSGEENLQGGWVELHFSN	MSQSGEENLQGSWVELHFPN	MWQSGEENLQGSWVELHFSN	MSQSGEENLQGSWFELHFSN	MSQSGEENLQGSMVELHFSN
MSQSGEENLQGHWVELHFSN	MSQSGEENLQGSWVELHFQN	MYQSGEENLQGSWVELHFSN	MSQSGEENLQGSWGELHFSN	MSQSGEENLQGSNVELHFSN
MSQSGEENLQGIWVELHFSN	MSQSGEENLQGSWVELHFRN	MSQAGEENLQGSWVELHFSN	MSQSGEENLQGSWHELHFSN	MSQSGEENLQGSPVELHFSN
MSQSGEENLQGKWVELHFSN	MSQSGEENLQGSWVELHFTN	MSQCGEENLQGSWVELHFSN	MSQSGEENLQGSWIELHFSN	MSQSGEENLQGSQVELHFSN
MSQSGEENLQGLWVELHFSN	MSQSGEENLQGSWVELHFVN	MSQDGEENLQGSWVELHFSN	MSQSGEENLQGSWKELHFSN	MSQSGEENLQGSRVELHFSN
MSQSGEENLQGMWVELHFSN	MSQSGEENLQGSWVELHFWN	MSQEGEENLQGSWVELHFSN	MSQSGEENLQGSWLELHFSN	MSQSGEENLQGSSVELHFSN
MSQSGEENLQGNWVELHFSN	MSQSGEENLQGSWVELHFYN	MSQFGEENLQGSWVELHFSN	MSQSGEENLQGSWMELHFSN	
MSQSGEENLQGPWVELHFSN	MSQSGEENLQGSWVELHFAN	MSQGGEENLQGSWVELHFSN	MSQSGEENLQGSWNELHFSN	
MSQSGEENLQGQWVELHFSN	MAQSGEENLQGSWVELHFSN	MSQHGEENLQGSWVELHFSN	MSQSGEENLQGSWPELHFSN	
MSQSGEENLQGRWVELHFSN	MCQSGEENLQGSWVELHFSN	MSQIGEENLQGSWVELHFSN	MSQSGEENLQGSWQELHFSN	

Supplementary Table 5. BNIP3 peptides (8mer) substituted.

Peptide sequences		
WVELHFSN	WVPLHFSN	WVELHHSN
AVELHFSN	WVQLHFSN	WVELHISN
CVELHFSN	WVRLHFSN	WVELHKSN
DVELHFSN	WVSLHFSN	WVELHLSN
EVELHFSN	WVTLHFSN	WVELHMSN
FVELHFSN	WVVLHFSN	WVELHNSN
GVELHFSN	WVWLHFSN	WVELHPSN
HVELHFSN	WVYLHFSN	WVELHQSN
IVELHFSN	WVEAHFSN	WVELHRSN
KVELHFSN	WVECHFSN	WVELHSSN
LVELHFSN	WVEDHFSN	WVELHTSN
MVELHFSN	WVEEHFSN	WVELHVSN
NVELHFSN	WVEFHFSN	WVELHWSN
PVELHFSN	WVEGHFSN	WVELHYSN
QVELHFSN	WVEHHFSN	WVELHFAN
RVELHFSN	WVEIHFSN	WVELHFCN
SVELHFSN	WVEKHFSN	WVELHFEN
TVELHFSN	WVEMHFSN	WVELHFFN
VVELHFSN	WVENHFSN	WVELHFGN
YVELHFSN	WVEPHFSN	WVELHFHN
WAELHFSN	WVEQHFSN	WVELHFIN
WCELHFSN	WVERHFSN	WVELHFKN
WDELHFSN	WVESHFSN	WVELHFLN
WEELHFSN	WVETHFSN	WVELHFMN
WFELHFSN	WVEVHFSN	WVELHFNN
WGELHFSN	WVEWHFSN	WVELHFPN
WHELHFSN	WVEYHFSN	WVELHFQN
WIELHFSN	WVELAFSN	WVELHFRN
WKELHFSN	WVELCFSN	WVELHFTN
WLELHFSN	WVELDFSN	WVELHFVN
WMELHFSN	WVELEFSN	WVELHFWN
WNELHFSN	WVELFFSN	WVELHFYN
WPELHFSN	WVELGFSN	WVELHFSA
WQELHFSN	WVELIFSN	WVELHFSC
WRELHFSN	WVELKFSN	WVELHFSD
WSELHFSN	WVELLFSN	WVELHFSE
WTELHFSN	WVELMFSN	WVELHFSF
WWELHFSN	WVELNFSN	WVELHFSG
WYELHFSN	WVELPFSN	WVELHFSH
WVELHFSN	WVELQFSN	WVELHFSI
WVALHFSN	WVELRFSN	WVELHFSK
WVCLHFSN	WVELSFSN	WVELHFSL
WVDLHFSN	WVELTFSN	WVELHFSM
WVFLHFSN	WVELVFSN	WVELHFSP
WVGLHFSN	WVELWFSN	WVELHFSQ
WVHLHFSN	WVELYFSN	WVELHFSR
WVILHFSN	WVELHASN	WVELHFSS
WVKLHFSN	WVELHCSN	WVELHFST
WVLLHFSN	WVELHDSN	WVELHFSV
WVMLHFSN	WVELHESN	WVELHFSW
WVNLHFSN	WVELHGSN	WVELHFSY

Supplementary Table 6. Serum chemistry in rats – Revocery phase

Sex: Male Day 29 relative to Start Date

Group 1, 0 mg/kg/day	ALT	AST	TP	ALB	BIL-T	ALP	GGT	sGLU	UREA	CRE	Ca	P	TCHO	TG	K	Na	Cl	GLB	A/G	CK
	(U/L)	(U/L)	(g/L)	(g/L)	(µmol/L)	(U/L)	(U/L)	(mmol/L)	(mmol/L)	(µmol/L)	(mmol/L)	(mmol/L)	(mmol/L)	(mmol/L)	(mmol/L)	(mmol/L)	(mmol/L)	(g/L)		(U/L)
1011	32	151	57.0	33.1	1.09	125	-1 E[a]	7.81	6.84	28	2.37	1.81	1.51	1.61	4.6	142	100	23.9	1.38	937
1012	21	130	56.3	33.4	1.24	120	-1 E[a]	5.28	5.42	26	2.35	2.16	1.61	0.64	4.4	144	101	22.9	1.46	1091
1013	29	108	57.0	33.0	1.20	92	-1 E[a]	8.99	6.87	22	2.28	2.18	2.07	1.10	4.3	141	99	24.0	1.38	652
1014	24	104	54.4	32.7	0.96	96	-1 E[a]	7.58	8.06	30	2.40	2.22	1.29	0.87	4.4	142	101	21.7	1.51	718
1015	23	133	58.1	34.0	1.08	96	-1 E[a]	8.36	8.44	28	2.24	1.69	1.55	0.48	4.4	142	103	24.1	1.41	765

Group 4, 12 mg/kg/day	ALT	AST	TP	ALB	BIL-T	ALP	GGT	sGLU	UREA	CRE	Ca	P	TCHO	TG	K	Na	Cl	GLB	A/G	CK
	(U/L)	(U/L)	(g/L)	(g/L)	(µmol/L)	(U/L)	(U/L)	(mmol/L)	(mmol/L)	(µmol/L)	(mmol/L)	(mmol/L)	(mmol/L)	(mmol/L)	(mmol/L)	(mmol/L)	(mmol/L)	(g/L)		(U/L)
4011	24	101	61.3	34.4	1.01	101	0 E[a]	7.52	7.96	28	2.39	1.79	2.41	1.37	4.6	142	99	26.9	1.28	687
4012	33	127	58.6	34.2	1.38	103	-1 E[a]	5.98	5.55	25	2.41	2.24	1.74	0.56	4.5	142	100	24.4	1.40	824
4013	24	191	59.5	32.1	1.31	139	-1 E[a]	6.16	7.30	30	2.37	2.08	1.80	0.42	4.7	140	100	27.4	1.17	1377
4014	30	113	58.6	33.8	1.13	97	-1 E[a]	8.24	8.71	29	2.31	1.96	1.60	0.90	4.8	140	100	24.8	1.36	758
4015	39	157	54.3	31.4	0.97	158	-1 E[a]	10.14	10.29	27	2.21	1.54	1.40	0.55	4.8	140	103	22.9	1.37	1016

Sex: Female Day 29 relative to Start Date

Group 1, 0 mg/kg/day	ALT	AST	TP	ALB	BIL-T	ALP	GGT	sGLU	UREA	CRE	Ca	P	TCHO	TG	K	Na	Cl	GLB	A/G	CK
	(U/L)	(U/L)	(g/L)	(g/L)	(µmol/L)	(U/L)	(U/L)	(mmol/L)	(mmol/L)	(µmol/L)	(mmol/L)	(mmol/L)	(mmol/L)	(mmol/L)	(mmol/L)	(mmol/L)	(mmol/L)	(g/L)		(U/L)
1511	25	156	59.3	35.5	1.46	54	0 E[a]	6.23	8.13	31	2.29	1.92	0.99	0.38	4.1	139	97	23.8	1.49	1129
1512	17	115	57.8	36.0	1.06	67	0 E[a]	8.34	7.45	27	2.40	2.17	0.84	0.30	4.2	142	102	21.8	1.65	735
1513	18	99	62.5	38.5	1.02	63	0 E[a]	8.47	6.20	28	2.35	1.72	1.96	0.50	4.0	141	102	24.0	1.60	737
1514	21	96	64.1	38.9	1.23	76	0 E[a]	5.92	8.47	31	2.44	2.60	2.39	0.34	3.7	144	99	25.2	1.54	782
1515	19	97	60.0	36.1	1.45	68	0 E[a]	11.29	9.02	31	2.37	1.94	1.43	0.32	4.3	140	102	23.9	1.51	515

Group 4, 12 mg/kg/day	ALT	AST	TP	ALB	BIL-T	ALP	GGT	sGLU	UREA	CRE	Ca	P	TCHO	TG	K	Na	Cl	GLB	A/G	CK
	(U/L)	(U/L)	(g/L)	(g/L)	(µmol/L)	(U/L)	(U/L)	(mmol/L)	(mmol/L)	(µmol/L)	(mmol/L)	(mmol/L)	(mmol/L)	(mmol/L)	(mmol/L)	(mmol/L)	(mmol/L)	(g/L)		(U/L)
4511	16	82	63.2	37.6	1.52	74	0 E[a]	5.86	7.34	32	2.52	2.04	2.12	0.44	4.1	142	100	25.6	1.47	508
4512	20	120	63.7	39.5	1.75	81	1 E[a]	7.71	7.05	27	2.40	1.86	0.91	0.35	4.0	141	100	24.2	1.63	858
4513	19	113	62.9	37.9	1.57	44	0 E[a]	6.68	6.17	30	2.43	2.38	2.06	0.44	3.9	139	99	25.0	1.52	549
4514	21	121	59.5	36.6	1.72	53	0 E[a]	8.24	9.35	26	2.48	2.20	1.66	0.57	4.4	139	99	22.9	1.60	589
4515	18	106	59.0	36.5	1.62	48	0 E[a]	5.87	7.93	36	2.43	2.16	1.13	0.39	4.0	146	107	22.5	1.62	660

E = Exclude
[a] *[RC:Below the Limit of Quantification]*

Abbreviations

ALT Alanine Aminotransferase
AST Aspartate Aminotransferase
TP Total Protein
ALB Albumin
BIL-T Total Bilirubin(Diasys)
ALP Alkaline Phosphatase
GGT Gamma-Glutamyltransferase
sGLU Glucose
UREA Urea
CRE Creatinine
Ca Calcium
P Inorganic Phosphorus
TCHO Total Cholesterol
TG Triglyceride
K Potassium
Na Sodium
Cl Chloride
GLB Globulin
A/G A/G Ratio
CK Creatine Kinase

Supplementary Table 7. Urine analysis parameters in rats – Recovery phase

Sex: Male Day 29 relative to Start Date

Group 1, 0 mg/kg/day	LEU	GLU	BIL	KET	BLD	PRO	URO	Color	Clarity	pH	SG	Volume (mL)
1011	-	-	-	-	-	-	-	L-YE	-	7.5	1.009	27
1012	-	-	-	-	-	-	-	L-YE	-	7.0	1.006	37
1013	-	-	-	-	-	+-	-	ST	-	7.0	1.020	12
1014	-	-	-	-	-	+-	-	ST	-	6.5	1.013	17
1015	-	-	-	-	-	+-	-	ST	-	8.0	1.014	22

Group 4, 12 mg/kg/day	LEU	GLU	BIL	KET	BLD	PRO	URO	Color	Clarity	pH	SG	Volume (mL)
4011	-	-	-	-	-	-	-	ST	-	7.5	1.011	27
4012	-	-	-	-	-	+-	-	ST	-	7.5	1.014	20
4013	-	-	-	-	+-	+-	-	ST	-	6.0	1.014	22
4014	-	-	-	-	-	+-	-	ST	-	7.5	1.020	15
4015	+	-	-	-	-	+-	-	ST	-	7.0	1.027	10

Sex: Female Day 29 relative to Start Date

Group 1, 0 mg/kg/day	LEU	GLU	BIL	KET	BLD	PRO	URO	Color	Clarity	pH	SG	Volume (mL)
1511	-	-	-	-	-	-	-	ST	-	5.5	1.012	17
1512	-	-	-	-	-	-	-	L-YE	-	6.5	1.009	3
1513	-	-	-	-	-	-	-	L-YE	-	6.0	1.009	25
1514	-	-	-	-	-	-	-	ST	-	6.5	1.015	15
1515	-	-	-	-	-	-	-	ST	-	7.0	1.017	12

Group 4, 12 mg/kg/day	LEU	GLU	BIL	KET	BLD	PRO	URO	Color	Clarity	pH	SG	Volume (mL)
4511	-	-	-	-	-	-	-	L-YE	-	6.0	1.009	25
4512	-	-	-	-	-	-	-	L-YE	-	6.0	1.007	25
4513	-	-	-	-	-	-	-	ST	-	6.5	1.014	12
4514	-	-	-	-	-	-	-	ST	-	6.5	1.019	12
4515	-	-	-	-	-	-	-	L-YE	-	6.0	1.005	35

Abbreviations

LEU Urine Leucocyte (Semi-quantitative)
GLU Urine Glucose (Semi-quantitative)
BIL Urine Bilirubin (Semi-quantitative)
KET Urine Ketones (Semi-quantitative)
BLD Urine Occult Blood (Semi-quantitative)
PRO Urine Protein (Semi-quantitative)
URO Urine Urobilinogen (Semi-quantitative)
Color Urine Color
Clarity Clarity
pH Urine pH
SG Urine Specific Gravity

Supplementary Table 8. Haematological values in rats – Recovery phase

Sex: Male Day 29 relative to Start Date

Group 1, 0 mg/kg/day	WBC (10^3/µL)	RBC (10^6/µL)	HGB (g/dL)	HCT (%)	MCV (fL)	MCH (pg)	MCHC (g/dL)	RDW (%)	#RET (10^9/L.)	%RET (%)	#NEUT (10^3/µL)	%NEUT (%)	#LYMP (10^3/µL)	%LYMP (%)	#MONO (10^3/µL)	%MONO (%)	#EOS (10^3/µL)	%EOS (%)	#BASO (10^3/µL)	%BASO (%)	PLT (10^3/µL)	MPV (fL)
1011	4.54	8.31	14.4	42.6	51.2	17.3	33.8	13.4	175.2	2.11	0.86	18.9	3.43	75.6	0.16	3.5	0.07	1.6	0.01	0.2	813	10.1
1012	4.44	7.76	14.3	41.2	53.1	18.5	34.9	13.1	191.7	2.47	0.63	14.2	3.58	80.7	0.14	3.2	0.05	1.2	0.01	0.1	905	10.4
1013	3.85	6.99	14.0	40.9	58.5	20.0	34.2	17.2	285.1	4.08	0.87	22.7	2.77	72.0	0.14	3.6	0.04	1.1	0.01	0.1	862	10.4
1014	3.00	7.37	14.2	40.8	55.5	19.2	34.7	12.5	193.1	2.62	0.56	18.6	2.26	75.2	0.13	4.3	0.04	1.5	0.00	0.0	719	10.4
1015	6.23	8.00	13.8	42.6	53.3	17.3	32.5	12.2	179.5	2.24	0.89	14.2	5.04	80.9	0.14	2.2	0.12	2.0	0.01	0.2	898	10.0

Group 4, 12 mg/kg/day	WBC (10^3/µL)	RBC (10^6/µL)	HGB (g/dL)	HCT (%)	MCV (fL)	MCH (pg)	MCHC (g/dL)	RDW (%)	#RET (10^9/L.)	%RET (%)	#NEUT (10^3/µL)	%NEUT (%)	#LYMP (10^3/µL)	%LYMP (%)	#MONO (10^3/µL)	%MONO (%)	#EOS (10^3/µL)	%EOS (%)	#BASO (10^3/µL)	%BASO (%)	PLT (10^3/µL)	MPV (fL)
4011	3.77	7.66	14.0	41.3	54.0	18.3	34.0	11.6	165.5	2.16	0.60	16.0	3.04	80.7	0.06	1.7	0.05	1.3	0.00	0.1	829	10.3
4012	3.95	7.55	14.1	41.1	54.4	18.6	34.2	12.9	215.7	2.86	0.83	21.1	3.01	76.3	0.04	1.0	0.05	1.3	0.01	0.1	752	10.7
4013	5.58	8.25	14.3	43.9	53.2	17.3	32.6	13.9	295.3	3.58	1.43	25.7	3.86	69.2	0.22	3.9	0.03	0.6	0.00	0.1	1040	9.8
4014	5.40	8.25	13.9	42.5	51.5	16.8	32.6	13.1	223.4	2.71	1.09	20.2	4.02	74.6	0.19	3.5	0.07	1.4	0.01	0.2	844	10.7
4015	4.33	7.38	13.3	38.7	52.4	18.1	34.5	13.7	191.7	2.60	0.93	21.5	3.23	74.7	0.06	1.5	0.08	1.9	0.01	0.2	831	10.1

Sex: Female Day 29 relative to Start Date

Group 1, 0 mg/kg/day	WBC (10^3/µL)	RBC (10^6/µL)	HGB (g/dL)	HCT (%)	MCV (fL)	MCH (pg)	MCHC (g/dL)	RDW (%)	#RET (10^9/L.)	%RET (%)	#NEUT (10^3/µL)	%NEUT (%)	#LYMP (10^3/µL)	%LYMP (%)	#MONO (10^3/µL)	%MONO (%)	#EOS (10^3/µL)	%EOS (%)	#BASO (10^3/µL)	%BASO (%)	PLT (10^3/µL)	MPV (fL)
1511	1.82	7.79	13.5	41.1	52.7	17.4	32.9	12.1	295.6	3.79	0.29	15.7	1.46	80.4	0.03	1.8	0.03	1.8	0.00	0.0	953	10.7
1512	1.68	6.47	12.8	37.2	57.4	19.8	34.5	23.4	209.7	3.24	0.27	16.1	1.32	78.2	0.04	2.6	0.05	2.8	0.00	0.0	914	10.5
1513	1.13	6.91	13.0	38.2	55.3	18.8	34.0	15.1	231.6	3.35	0.52	45.6	0.46	40.5	0.08	7.5	0.07	6.2	0.00	0.0	962	10.3
1514	4.13	7.04	13.4	40.6	57.6	19.1	33.1	11.9	242.5	3.45	0.59	14.3	3.34	80.9	0.08	2.0	0.10	2.3	0.01	0.2	952	10.1
1515	2.97	6.93	13.7	40.2	58.1	19.8	34.1	11.8	260.1	3.76	0.66	22.2	2.21	74.4	0.05	1.6	0.04	1.3	0.00	0.1	982	10.0

Group 4, 12 mg/kg/day	WBC (10^3/µL)	RBC (10^6/µL)	HGB (g/dL)	HCT (%)	MCV (fL)	MCH (pg)	MCHC (g/dL)	RDW (%)	#RET (10^9/L.)	%RET (%)	#NEUT (10^3/µL)	%NEUT (%)	#LYMP (10^3/µL)	%LYMP (%)	#MONO (10^3/µL)	%MONO (%)	#EOS (10^3/µL)	%EOS (%)	#BASO (10^3/µL)	%BASO (%)	PLT (10^3/µL)	MPV (fL)
4511	2.78	7.19	13.8	40.2	56.0	19.2	34.4	13.0	254.0	3.53	0.43	15.3	2.24	80.7	0.05	1.8	0.05	1.7	0.00	0.1	929	10.0
4512	2.22	6.88	13.4	39.5	57.5	19.5	33.8	12.1	238.8	3.47	0.44	19.6	1.64	73.6	0.07	2.9	0.07	3.4	0.00	0.1	936	10.2
4513	4.27	7.46	14.1	41.7	56.0	18.9	33.8	11.0	270.8	3.63	0.56	13.2	3.55	83.2	0.10	2.4	0.04	0.8	0.01	0.1	827	9.8
4514	4.17	7.74	14.3	41.5	53.6	18.4	34.4	12.0	239.1	3.09	0.62	14.9	3.38	81.2	0.10	2.4	0.05	1.2	0.00	0.1	885	9.6
4515	4.48	7.75	14.0	43.0	55.5	18.1	32.5	11.8	203.1	2.62	0.47	10.6	3.81	85.0	0.11	2.5	0.07	1.5	0.00	0.1	765	10.7

Supplementary Table 9. Toxicokinetic parameters of B-017 following once daily i.v. injection of B-017 to male and female rats for 14 days

Dose (mg/kg/day)	Study Day	Sex	C_0 (ng/mL)	C_{max} (ng/mL)	T_{max} (h)	$T_{1/2}$ (h)	$AUC_{0\text{-}24h}$ (h*ng/mL)
3	1	Male	1360	1010	0.1	0.3	479
		Female	1020	863	0.1	0.4	510
	14	Male	1790	1460	0.1	0.3	812
		Female	1800	1510	0.1	0.4	917
6	1	Male	1840	1430	0.1	0.4	821
		Female	1890	1570	0.1	0.3	964
	14	Male	5290	4050	0.1	0.4	2220
		Female	3680	2990	0.1	0.3	1750
12	1	Male	7890	5750	0.1	0.6	2900
		Female	6750	4990	0.1	0.6	2780
	14	Male	9890	7550	0.1	0.5	4210
		Female	10700	8290	0.1	0.5	4950

Supplementary Table 10. The modified Bederson's neurological deficit scale

Score	*signs*
0	Normal
1	Flexion of the left front paw
2	Flexion of the left front paw and decreased resistance to lateral push
3	Circling to one side
4	Circling and spinning around the cranial-caudal axis, loss of walking or righting reflex
5	Comatose or moribund

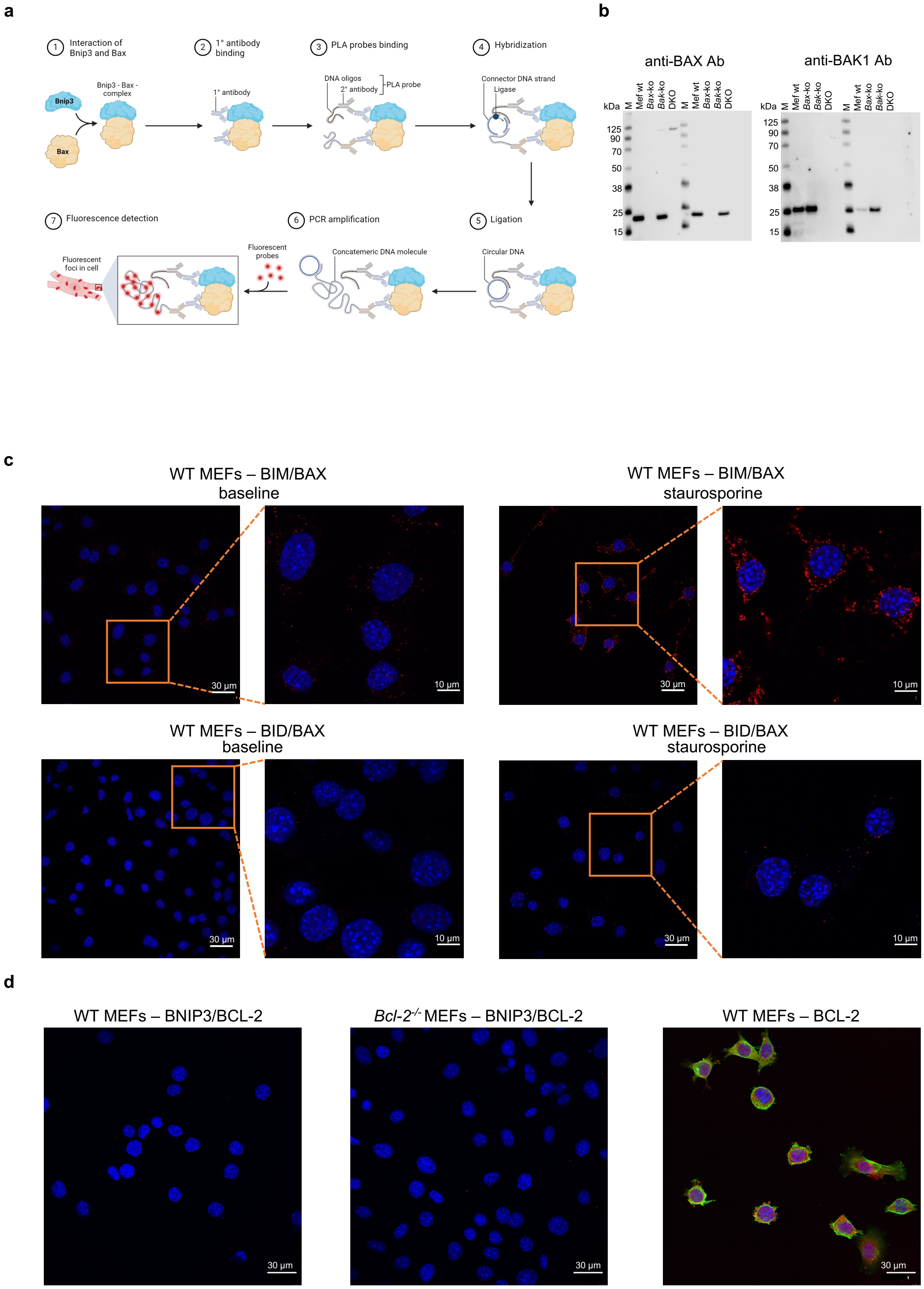
a
1 Interaction of Bnip3 and Bax
2 1° antibody binding
3 PLA probes binding
4 Hybridization
Bnip3
Bax
Bnip3 - Bax - complex
1° antibody
DNA oligos
2° antibody
PLA probe
Connector DNA strand
Ligase
7 Fluorescence detection
6 PCR amplification
5 Ligation
Fluorescent foci in cell
Fluorescent probes
Concatemeric DNA molecule
Circular DNA
b
anti-BAX Ab
anti-BAK1 Ab
kDa
M
Mef wt
Bax-ko
Bak-ko
DKO
125
90
70
50
38
25
15
c
WT MEFs – BIM/BAX baseline
WT MEFs – BIM/BAX staurosporine
WT MEFs – BID/BAX baseline
WT MEFs – BID/BAX staurosporine
30 µm
10 µm
d
WT MEFs – BNIP3/BCL-2
Bcl-2-/- MEFs – BNIP3/BCL-2
WT MEFs – BCL-2
30 µm

Supplementary Fig. 1. Additional data on the identification of BNIP3 interactions with BCL-2 family members.

a, Workflow for the proximity ligation assay (PLA, Created in BioRender. Settelmeier, S. (2024). BioRender.com/x56a488). **b**, Immunoblot analysis of BAX expression in *Bak* knockout (*Bak*$^{-/-}$ mouse embryonic fibroblasts (MEFs) and of BAK in Bax knockout (*Bax*$^{-/-}$) MEFs, using the anti-BAX antibody (1:1,000, C2772, Cell Signaling; anti-rabbit HRP, 1:20.000) and the anti-BAK1 antibody (1:1,000, 12105, Cell Signaling). (n = 2 independent experiments). **c**, PLA in wild-type (WT) MEFs revealed that BIM and BAX are not localised in close proximity under baseline conditions (left, top). The interaction requires a stimulus, as demonstrated by staurosporine (STS) treatment (right, top). The same applies to the interaction of BID with BAX (bottom). A pair of validated primary antibodies against BIM, BAX, and BID, as well as a pair of secondary species-specific antibodies conjugated to complementary oligonucleotides, were used. Generation of proximity ligation puncta, the specific PLA signal, occurs when targets are in close proximity ($\leq$40 nm). Scale bars, 30 µm, 10 µm. (n = 3 independent experiments). **d**, PLA in WT MEFs showed that BNIP3 and BCL-2 are not localised in close proximity (left). A pair of validated primary antibodies against BNIP3 and BCL-2 and a pair of secondary species-specific antibodies conjugated to complementary oligonucleotides were used. Scale bars, 30 µm. No signal was observed in *Bcl-2*$^{-/-}$ MEFs (middle). To validate that the antibody binds to its specific target, BCL-2 was detected *via* immunofluorescence staining in WT MEFs (red, right). (Nuclei stained with DAPI, blue, cytoskeleton stained with phalloidin, green), (n = 3 independent experiments).

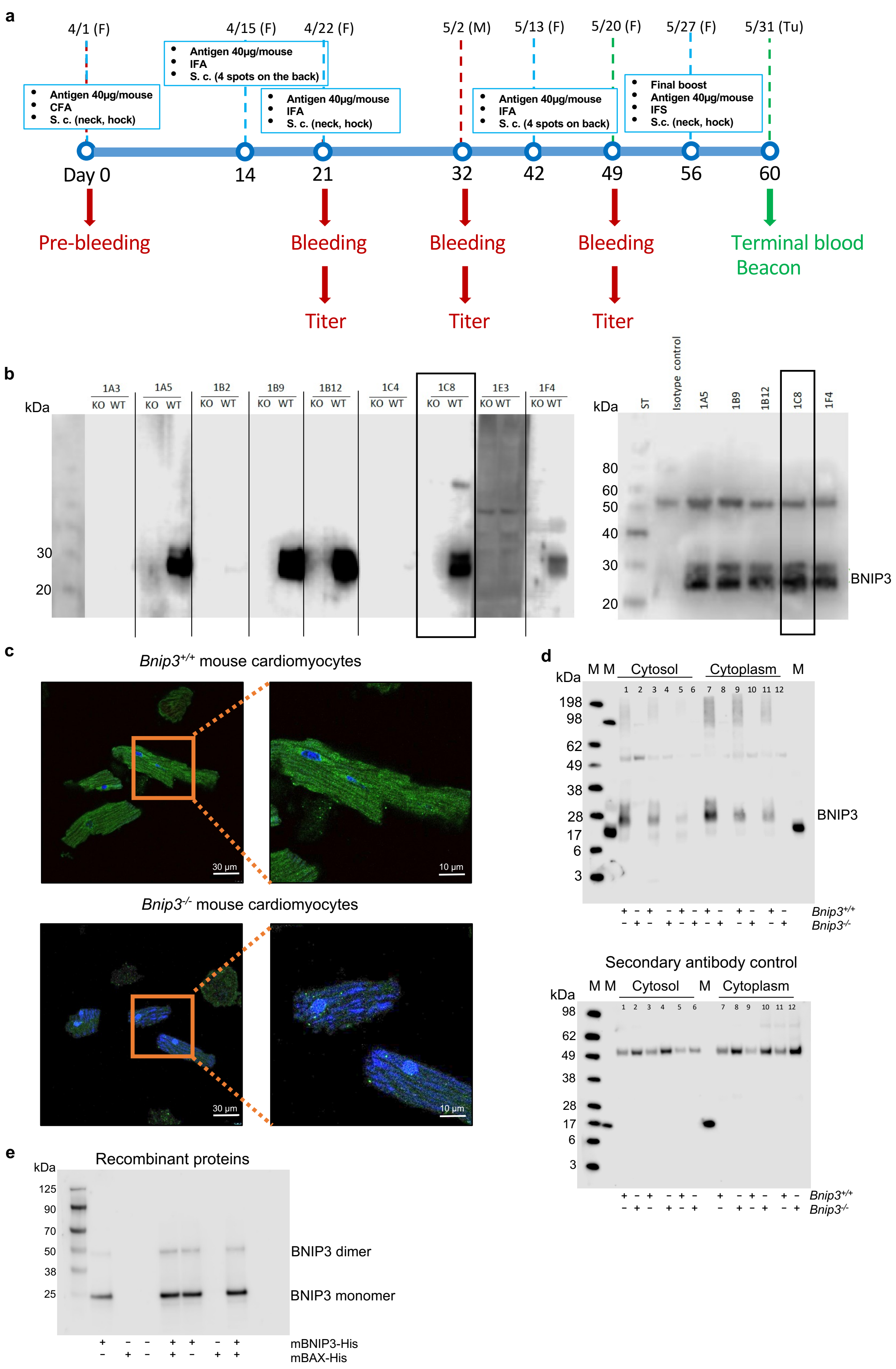
a
4/1 (F)
4/15 (F)
4/22 (F)
5/2 (M)
5/13 (F)
5/20 (F)
5/27 (F)
5/31 (Tu)
Antigen 40µg/mouse
CFA
S. c. (neck, hock)
Antigen 40µg/mouse
IFA
S. c. (4 spots on the back)
Antigen 40µg/mouse
IFA
S. c. (neck, hock)
Antigen 40µg/mouse
IFA
S. c. (4 spots on back)
Final boost
Antigen 40µg/mouse
IFS
S.c. (neck, hock)
Day 0
14
21
32
42
49
56
60
Pre-bleeding
Bleeding
Bleeding
Bleeding
Terminal blood
Beacon
Titer
Titer
Titer
b
kDa
1A3
1A5
1B2
1B9
1B12
1C4
1C8
1E3
1F4
KO WT
30
20
ST
Isotype control
1A5
1B9
1B12
1C8
1F4
80
60
50
40
30
20
BNIP3
c
Bnip3+/+ mouse cardiomyocytes
30 µm
10 µm
Bnip3-/- mouse cardiomyocytes
30 µm
10 µm
d
kDa
M M Cytosol Cytoplasm M
1 2 3 4 5 6 7 8 9 10 11 12
198
98
62
49
38
28
17
6
3
BNIP3
Bnip3+/+
Bnip3-/-
Secondary antibody control
M M Cytosol M Cytoplasm
98
62
49
38
28
17
6
3
Bnip3+/+
Bnip3-/-
e
Recombinant proteins
kDa
125
90
70
50
38
25
BNIP3 dimer
BNIP3 monomer
mBNIP3-His
mBAX-His

Supplementary Fig. 2. Generation of a mouse monoclonal antibody against mouse BNIP3 by single B cell cloning technology.

a, Immunisation regimen. Five female SJL-Elite mice were immunised with 40 µg of recombinant mouse BNIP3. **b,** Immunoblot analysis of nine clones. *Bnip3*$^{+/+}$ and *Bnip3*$^{-/-}$ mouse heart lysates were loaded at 20 µg per lane for SDS-PAGE. Purified antibodies were diluted to a final concentration of 500 ng ml^{-1} (left). Immunoprecipitation using individual antibodies was followed by immunoblot analysis probed with clone 1B9 (right). The 1C8 antibody was selected for all subsequent experiments. **c, d,** Knockout validation to confirm the anti-BNIP3 antibody 1C8 specificity for BNIP3 in isolated *Bnip3*$^{-/-}$ mouse cardiomyocytes (BNIP3 green, nuclei blue, n = 2 biological replicates) and mouse heart cytosolic and cytoplasmic fractions (n = 3 biological replicates). The antibody yielded no signal in *Bnip3*$^{-/-}$ mouse cardiomyocytes (**c**) or in mouse heart cytosolic and cytoplasmic fractions (**d**). In isolated *Bnip3*$^{+/+}$ mouse cardiomyocytes (**c**) and mouse heart cytosolic and cytoplasmic fractions (**d**) the antibody detected the BNIP3 signal. For immunoblot analysis 20 µg (lanes 1, 2, 7, 8), 15 µg (lanes 3, 4 9, 10) and 10 µg of protein (lanes 5, 6, 11, 12) were used. **e**, Immunoblot analysis using recombinant mouse (m) BNIP3-His (200 nM) and recombinant mBAX-His (200 nM). The anti-BNIP3 antibody 1C8 specifically detected recombinant mBNIP3. (n = 2 independent experiments).

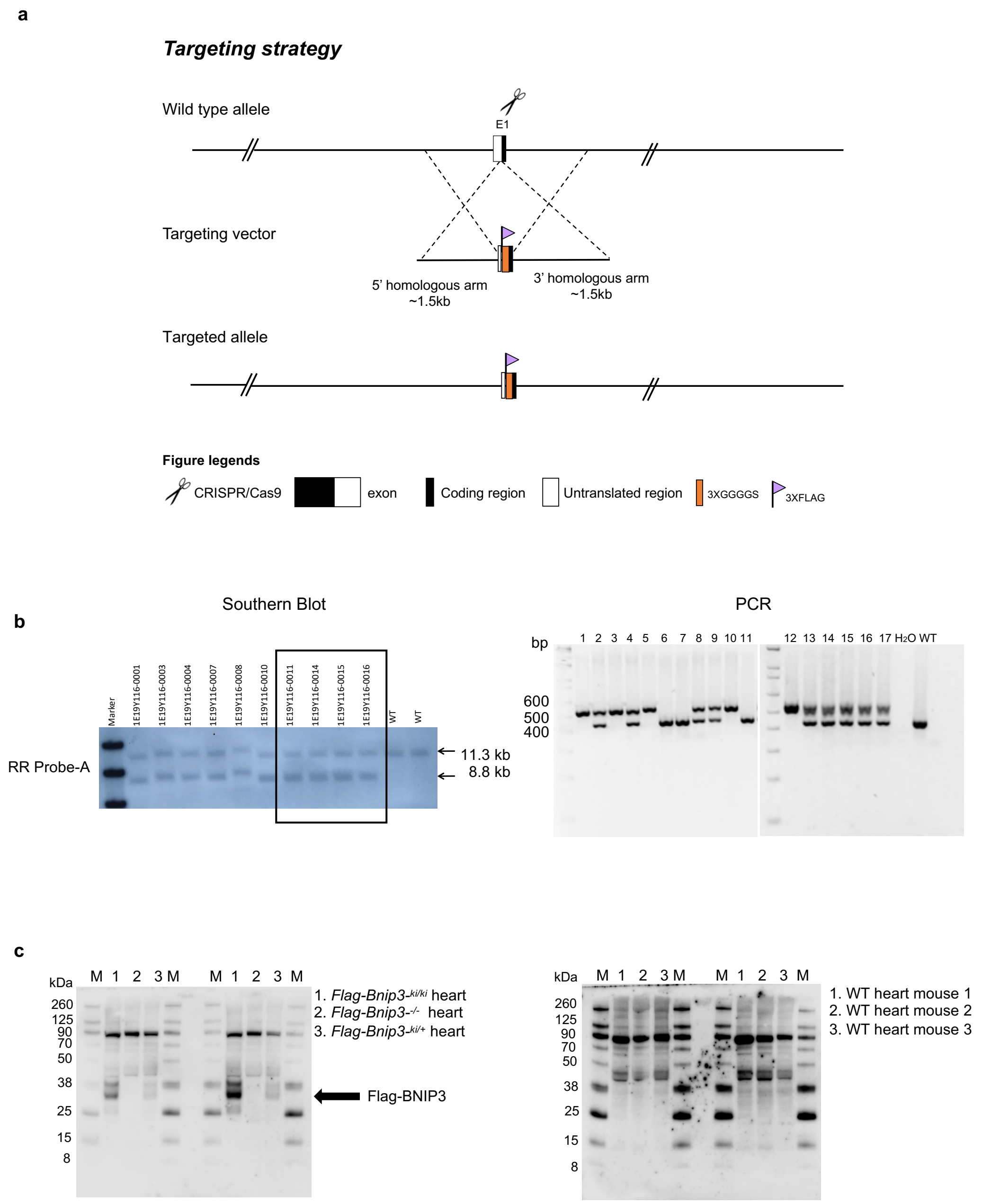

Supplementary Fig. 3. Generation of an EGE-LY2-0116-A-3xFlag knockin (ki) mouse model by EGE® system, a CRISPR/Cas9 based technology.

a, Targeting strategy (provided and assigned by Biocytogen Co., Ltd). The design was based on transcript-201 (NM_009760.4, NP_033890.1). EGE® is a trademark of Biocytogen Co., Ltd, registered in 2015. **b**, Southern blot using the restriction enzyme BspHI (wild-type (WT; 11.3 kb; targeted 8.8) (left). The marked mice (F1 generation) were used for breeding. PCR screening was performed on *Flag-Bnip3*$^{ki/ki}$, *Flag-Bnip3*$^{ki/+}$, *Flag- Bnip3* $^{-/-}$ mice from heterozygous crosses (434-bp wild-type allele product and 545-bp Bnip3-3xFlag allele product) (right). Female and male mice of the F5 generation were used for experiments. **c,** Immunoblot analysis using an anti-Flag antibody. The antibody yielded a signal in *Flag-Bnip3*$^{ki/ki}$ and *Flag-Bnip3*$^{ki/+}$ mouse hearts (left, top), whereas no signal was observed in *Flag-Bnip3* $^{-/-}$ (left, top) and C57BL/6J wild-type mouse hearts (left, bottom). Western blot analysis using the fluorescence-labelled anti-BNIP3 antibody 1C8 (200 ng ml^{-1}) following co-immunoprecipitation with the anti-Flag antibody verified its immunoprecipitation in *Flag-Bnip3*$^{ki/+}$ mouse hearts. *Flag-Bnip3* $^{-/-}$ mouse hearts served as controls (right).

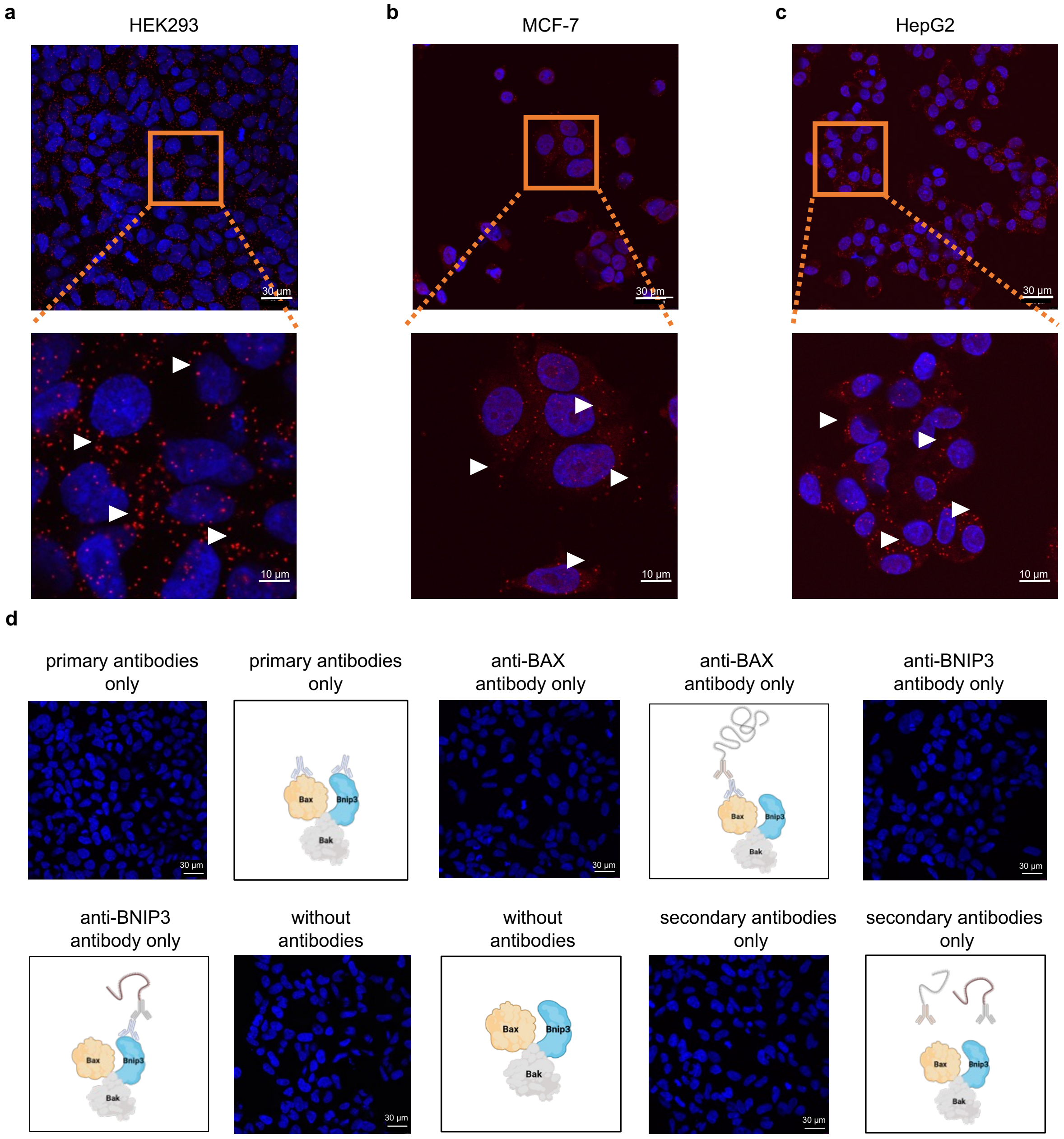

Created in BioRender. Settelmeier, S. (2024). BioRender.com/a79v328 and BioRender.com/p11n850.

Supplementary Fig. 4. Identification of BNIP3/BAX in different cell lines.

a-c, *In situ* proximity ligation assay (PLA) showed BNIP3 and BAX interactions in HEK293, MCF-7, and HepG2 cell lines, as indicated by the red dots with white arrows. A pair of validated primary antibodies, a monoclonal antibody against BNIP3 (1C8, 1:200) and polyclonal antibody against BAX (1:100) and a pair of secondary species-specific antibodies conjugated to complementary oligonucleotides were used. Proximity ligation puncta, the specific PLA signal, are generated when targets are in close proximity ($\leq$40 nm). Scale bars, 30 µm, 10 µm. (n = 2 independent experiments). **d,** PLA controls. Representative micrographs of control staining in HEK293 cells showing no signal. (Nuclei stained with DAPI, blue). Graphics created in BioRender. BioRender.com/a79v328 and BioRender.com/p11n850.

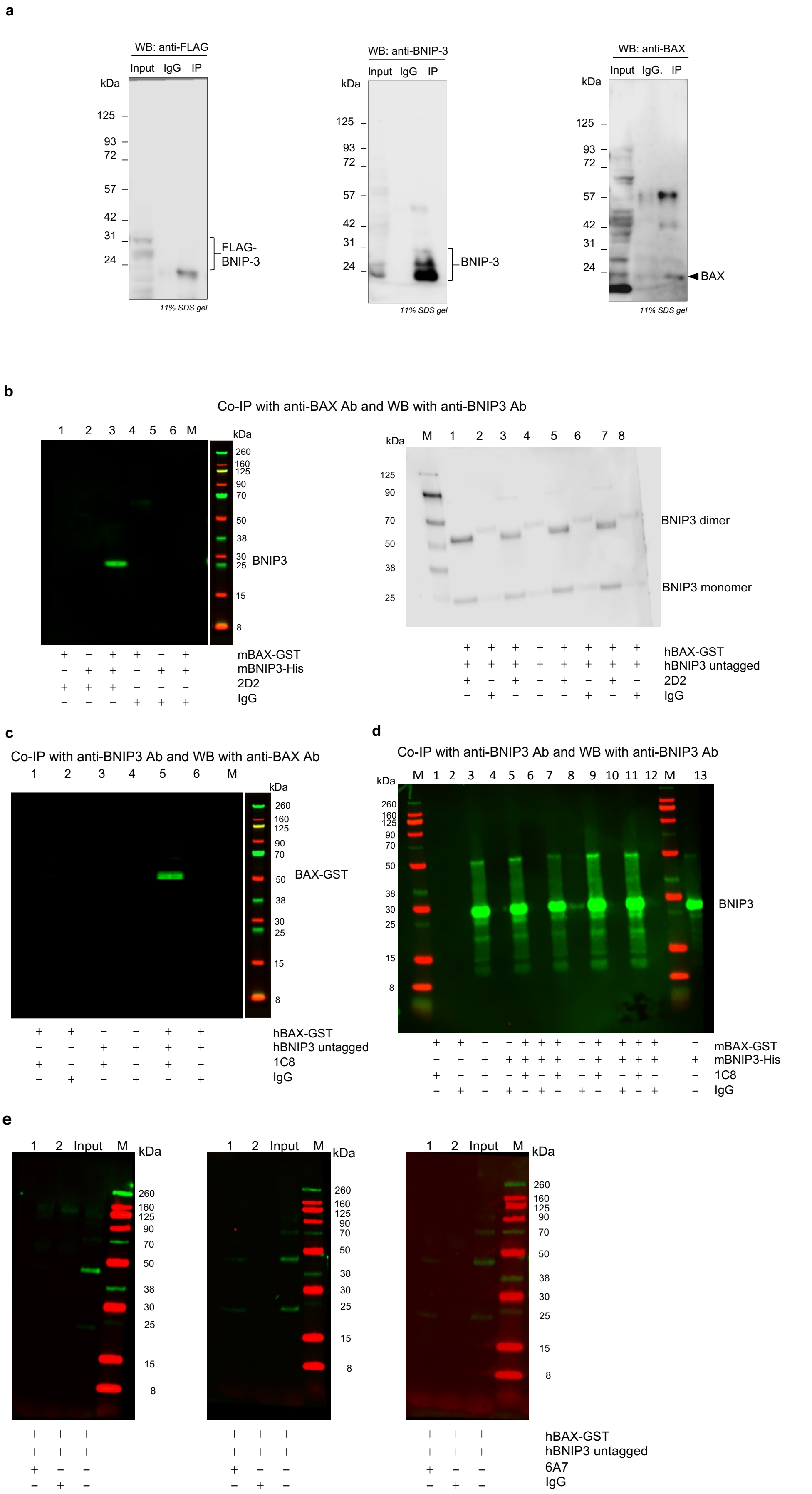

a
WB: anti-FLAG
Input IgG IP
kDa
125
93
72
57
42
31
24
FLAG-
BNIP-3
11% SDS gel
WB: anti-BNIP-3
Input IgG IP
kDa
125
93
72
57
42
31
24
BNIP-3
11% SDS gel
WB: anti-BAX
Input IgG. IP
kDa
125
93
72
57
42
31
24
◄BAX
11% SDS gel
b
Co-IP with anti-BAX Ab and WB with anti-BNIP3 Ab
1 2 3 4 5 6 M
kDa
260
160
125
90
70
50
38
30
25
BNIP3
15
8
mBAX-GST
mBNIP3-His
2D2
IgG
kDa
M 1 2 3 4 5 6 7 8
125
90
70
50
38
25
BNIP3 dimer
BNIP3 monomer
hBAX-GST
hBNIP3 untagged
2D2
IgG
c
Co-IP with anti-BNIP3 Ab and WB with anti-BAX Ab
1 2 3 4 5 6 M
kDa
260
160
125
90
70
50
BAX-GST
38
30
25
15
8
hBAX-GST
hBNIP3 untagged
1C8
IgG
d
Co-IP with anti-BNIP3 Ab and WB with anti-BNIP3 Ab
kDa
M 1 2 3 4 5 6 7 8 9 10 11 12 M 13
260
160
125
90
70
50
38
30
25
15
8
BNIP3
mBAX-GST
mBNIP3-His
1C8
IgG
e
1 2 Input M
kDa
260
160
125
90
70
50
38
30
25
15
8
hBAX-GST
hBNIP3 untagged
6A7
IgG

Supplementary Fig. 5. Identification of BNIP3/BAX interaction in mouse heart lysates and *in vitro*.

a, Co-immunoprecipitation of Flag-BNIP3 using *Flag-Bnip3*$^{ki/ki}$ mouse heart lysates. Western blot analysis of Flag-BNIP3 conjugates using the anti-BAX antibody 2D2 showed that endogenous BAX was co-isolated. Western blot analysis using an anti-Flag antibody and the anti-BNIP3 antibody 1C8 following co-immunoprecipitation verified immunoprecipitation of Flag-BNIP3. (n = 3 independent experiments). **b, c,** Co-immunoprecipitation of recombinant mouse (m)/human (h)BAX-GST and mBNIP3-His/hBNIP3 untagged. Western blot analysis following SDS-PAGE showed that BNIP3 was co-isolated with BAX and vice versa, supporting a BNIP3/BAX interaction. **d,** Western blot analysis using the fluorescence-labelled anti-BNIP3 antibody 1C8 following co-immunoprecipitation with the unlabelled anti-BNIP3 antibody 1C8 verified its immunoprecipitation. **e**, Western blot analysis of the activation status of BAX within the BNIP3/BAX complex. Recombinant untagged human BNIP3 (hBNIP3) and human BAX-GST (hBAX-GST) were incubated simultaneously with 6A7 antibody and IgG control. Western blot analysis following SDS-PAGE using the anti-BAX antibody 2D2 showed that BAX is in its inactive state within the BNIP3/BAX complex. (n = 3 independent experiments).

a

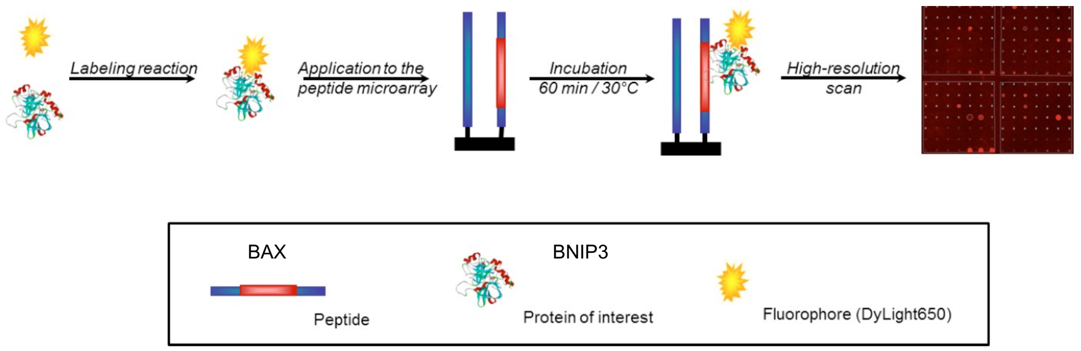

b

PEPTIDE 1: MDGSGEQLGSGGPTS**SEQIM** (α**1**)

PEPTIDE 2: **SEQIMKTGAFLLQGFIQDRA** (α**1**)

PEPTIDE 3: **A**GRMAGETPELTLEQPPQD**A**

PEPTIDE 4: D**ASTKKLSECLRRIGDELD**S (α**2**)

PEPTIDE 5: ELDSN**MELQRMIA**DVDTDSP (α**3**)

PEPTIDE 6: TDSP**REVFFRVAADM**FADGN (α**4**)

PEPTIDE 7: W**GRVVALFYFASKLVLKALC** (α**5**)

PEPTIDE 8: V**PELIRTIMGWTLDFLRE**RL (α**6**)

PEPTIDE 9: RLLV**WIQD**QGG**WEGLLSY**FG (α**7**, α**8**)

PEPTIDE 10: T**WQTVTIFVAGVLTASLTIW** (α**9**)

PEPTIDE 11: *MFADGNFNWGRVVALFYFAS* (*BH1 DOMAIN*)

PEPTIDE 12: LV*WIQDQGGWEGLLSYF*GTP (*BH2 DOMAIN*)

PEPTIDE 13: ASTKK*LSECLRRIGDELDSN* (*BH3 DOMAIN*)

Supplementary Fig. 6. Additional data on BNIP3/BAX peptide microarray.

a, Workflow for protein-peptide microarray (provided and assigned by JPT Peptide Technologies). **b**, Library of 13 synthesised BAX peptides. The peptides were designed to each comprise one or two of the BAX alpha-helices, or one of the three BH domains.

a

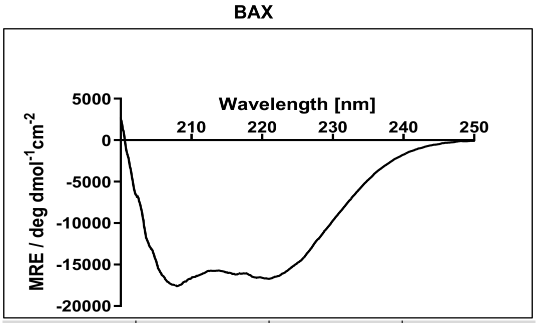

α-Helix	β-Sheet	Random coil	Total
0.54	0.15	0.32	1

b

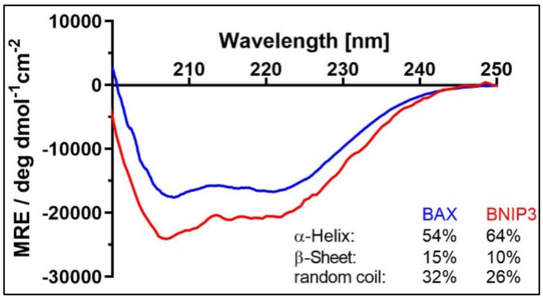

c

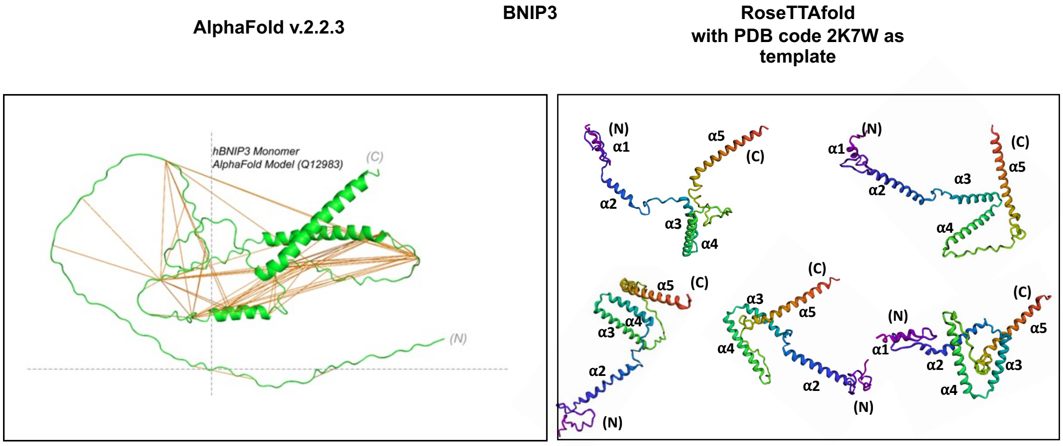

Supplementary Fig. 7. BNIP3 3D structure models.

a, The secondary structure composition of recombinant hBAX, as determined by circular dichroism (CD) spectroscopy, is in good agreement with its nuclear magnetic resonance structure (PDB code 2K7W, model 1, alpha-helices: 63%). **b**, Overlay of the CD spectra of BAX and BNIP3. **c**, Computational approach to predict the structure of BNIP3 using the neural network-based models AlphaFold v.2.2.3 (left) and RoseTTAFold (template structure: PDB code 2K7W) (right). Both, the AlphaFold model and the RoseTTAFold models, which consist of four to five helices without a defined tertiary structure, did not agree with the CD data obtained with recombinant hBNIP3. The DSSO cross-links identified in recombinant hBNIP3 by mass spectrometry mapped on the AlphaFold model (highlighted in orange) also do not confirm this model.

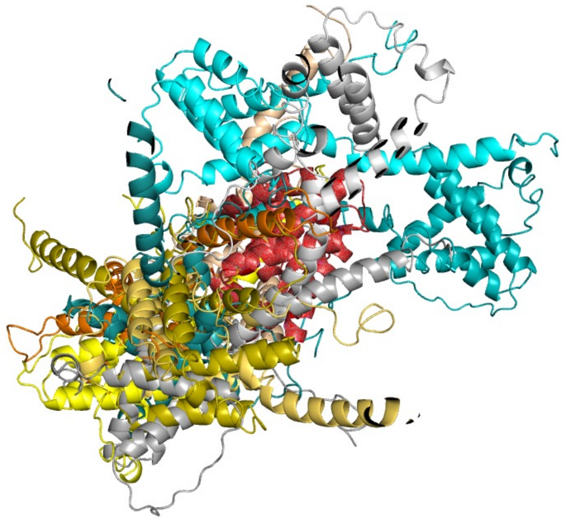

Supplementary Fig. 8. Additional data on the interaction of BNIP3 and BAK1

HDOCK docking simulation of BNIP3 and BAK1 (red; pdb template code 2JCN) did not yield a favourable docking profile and did not converge on a defined binding interface. The 10 best models were aligned according to BAK1 (shown in red, BNIP3 molecules coloured by number).

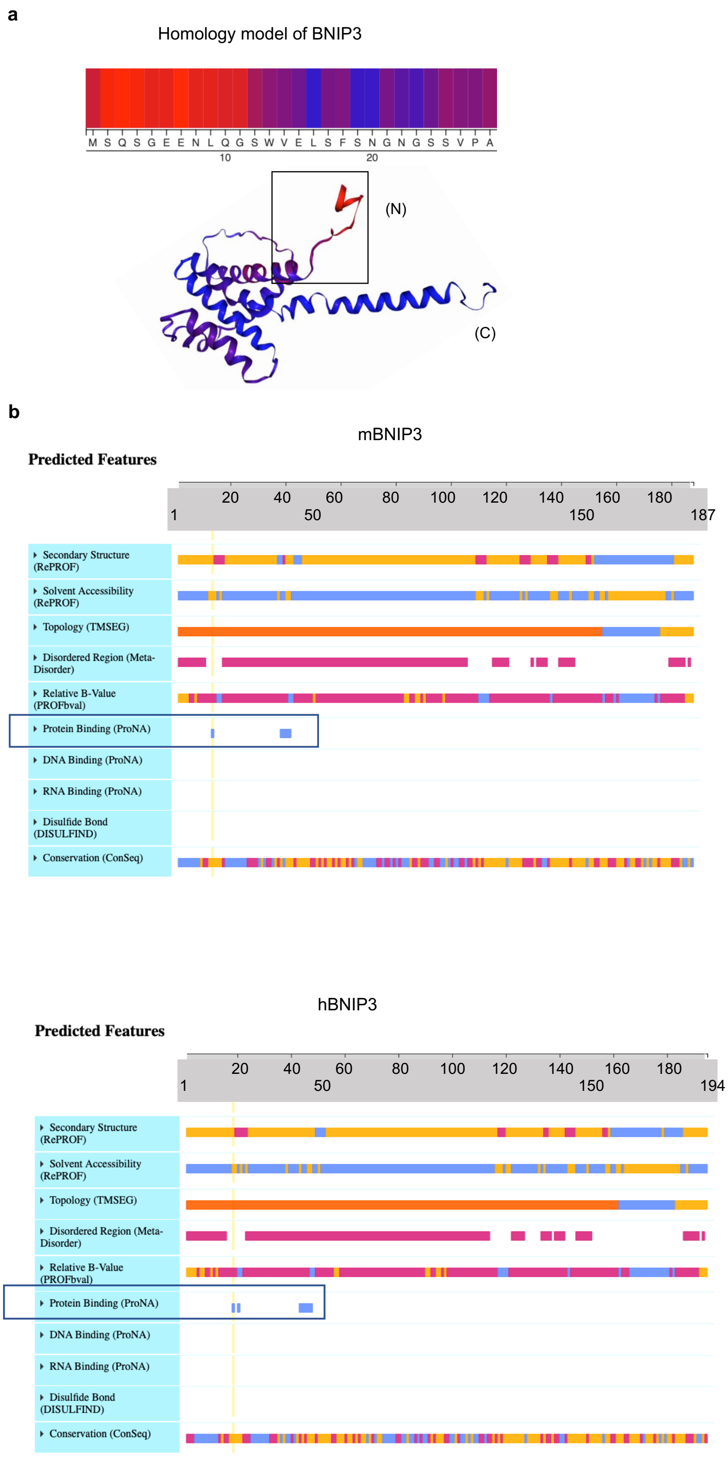

Supplementary Fig. 9. Additional data on the functional domain of BNIP3.

a, Prediction of a functional binding domain in BNIP3 using the DeepFRI server (https://beta.deepfri.flatironinstitute.org/workspace/3HDE54/predictions/QYTKE5). The predicted N-terminal region as a functional binding domain supports our hypothesis that the N-terminus of BNIP3 may be involved in BAX activation, which also implies the proximity of the N-terminus to the activation site as determined by cross-linking mass spectrometry. **b,** Prediction of a functional binding domain in recombinant human and mouse BNIP3 using the PredictProtein web server (https://predictprotein.org/). The predicted N-terminal region as a functional binding domain in recombinant mouse (top) and human BNIP3 (bottom) agreed with the results obtained with the DeepFRI algorithm.

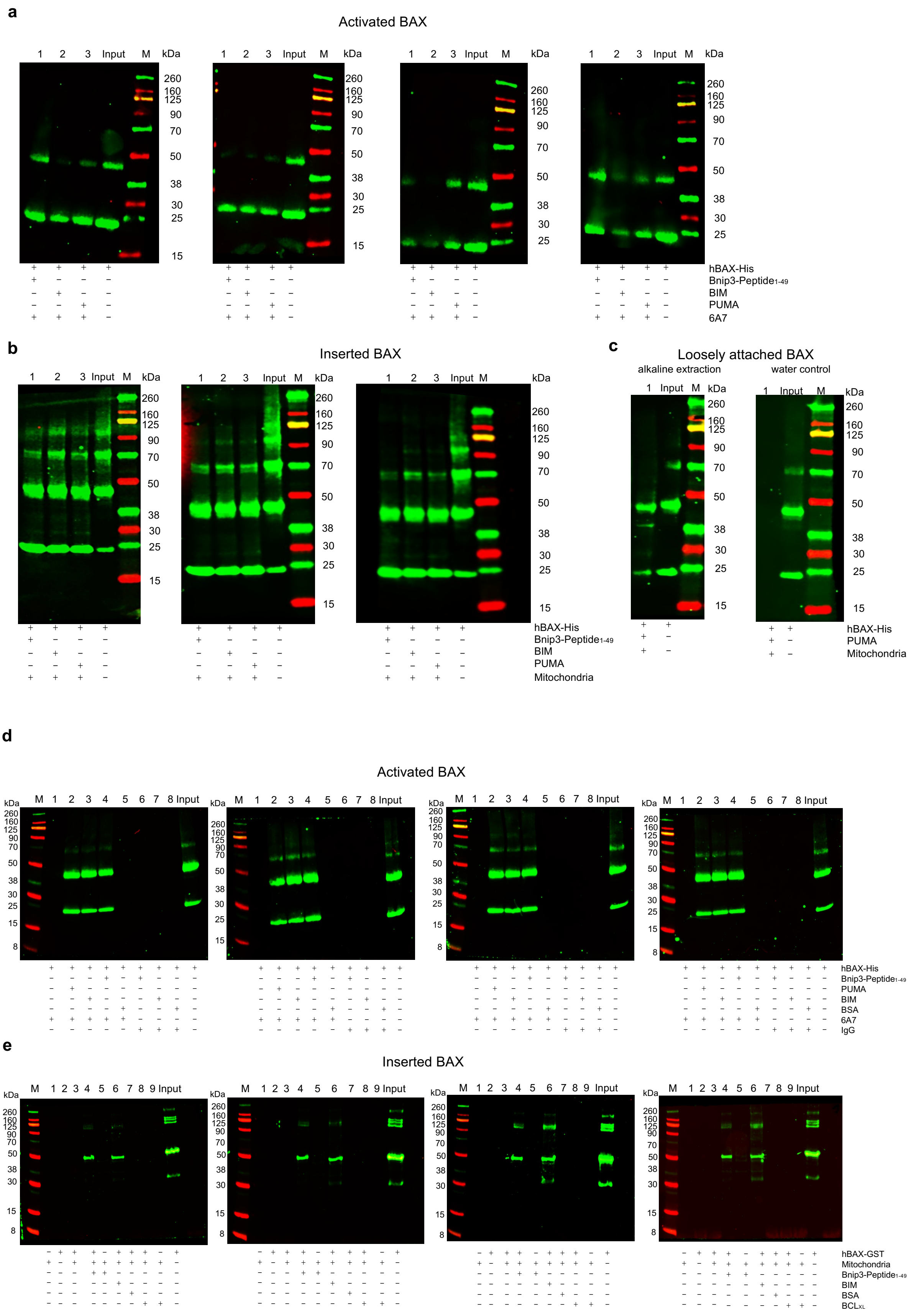
a
Activated BAX
1 2 3 Input M kDa
260 160 125 90 70 50 38 30 25 15
hBAX-His
Bnip3-Peptide1-49
BIM
PUMA
6A7
b
Inserted BAX
1 2 3 Input M kDa
hBAX-His
Bnip3-Peptide1-49
BIM
PUMA
Mitochondria
c
Loosely attached BAX
alkaline extraction
water control
1 Input M kDa
hBAX-His
PUMA
Mitochondria
d
Activated BAX
kDa M 1 2 3 4 5 6 7 8 Input
260 160 125 90 70 50 38 30 25 15 8
hBAX-His
Bnip3-Peptide1-49
PUMA
BIM
BSA
6A7
IgG
e
Inserted BAX
kDa M 1 2 3 4 5 6 7 8 9 Input
260 160 125 90 70 50 38 30 15 8
hBAX-GST
Mitochondria
Bnip3-Peptide1-49
BIM
BSA
BCLXL

Supplementary Fig. 10. Additional data on the activation capacity of BNIP3-Peptide$_{1-49}$. **a, b,** Immunoblot analyses of activated and inserted BAX, related to Fig. 2b. c, Immunoblot analysis showing successful alkaline extraction, evidenced by the removal of loosely attached BAX (left) and a negative control using water (right). **d, e,** Impact of bovine serum albumin (BSA, 5 µM) and the antiapoptotic protein BCL$_{XL}$ (500 nM) on the 1st and 2nd activation steps of BAX. Staurosporine (STS), BNIP3-Peptide$_{1-49}$ (Peptide$_{1-49}$), PUMA and BIM were used as stimulation agents. Immunoblot analyses of activated (**d**) and inserted BAX (**e**) showed no activation of BAX upon treatment with BSA and BCL$_{XL}$.

a

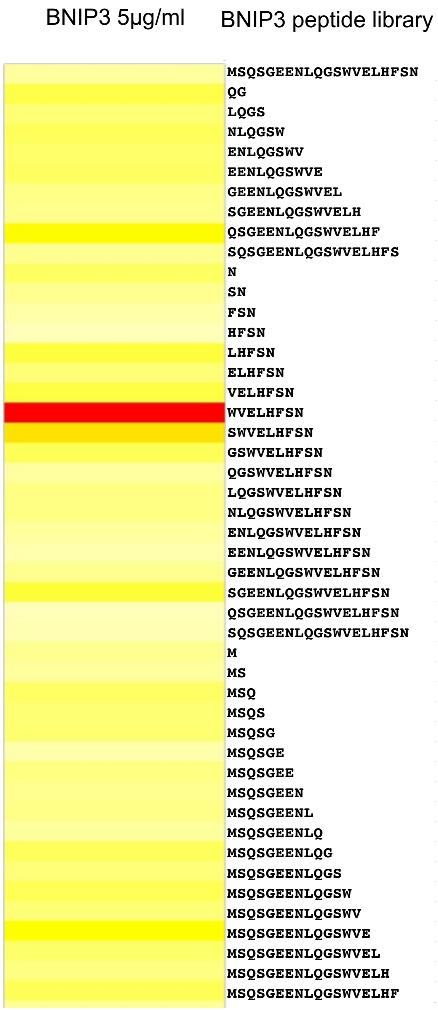
BNIP3 5µg/ml
BNIP3 peptide library
MSQSGEENLQGSWVELHFSN
QG
LQGS
NLQGSW
ENLQGSWV
EENLQGSWVE
GEENLQGSWVEL
SGEENLQGSWVELH
QSGEENLQGSWVELHF
SQSGEENLQGSWVELHFS
N
SN
FSN
HFSN
LHFSN
ELHFSN
VELHFSN
WVELHFSN
SWVELHFSN
GSWVELHFSN
QGSWVELHFSN
LQGSWVELHFSN
NLQGSWVELHFSN
ENLQGSWVELHFSN
EENLQGSWVELHFSN
GEENLQGSWVELHFSN
SGEENLQGSWVELHFSN
QSGEENLQGSWVELHFSN
SQSGEENLQGSWVELHFSN
M
MS
MSQ
MSQS
MSQSG
MSQSGE
MSQSGEE
MSQSGEEN
MSQSGEENL
MSQSGEENLQ
MSQSGEENLQG
MSQSGEENLQGS
MSQSGEENLQGSW
MSQSGEENLQGSWV
MSQSGEENLQGSWVE
MSQSGEENLQGSWVEL
MSQSGEENLQGSWVELH
MSQSGEENLQGSWVELHF

b

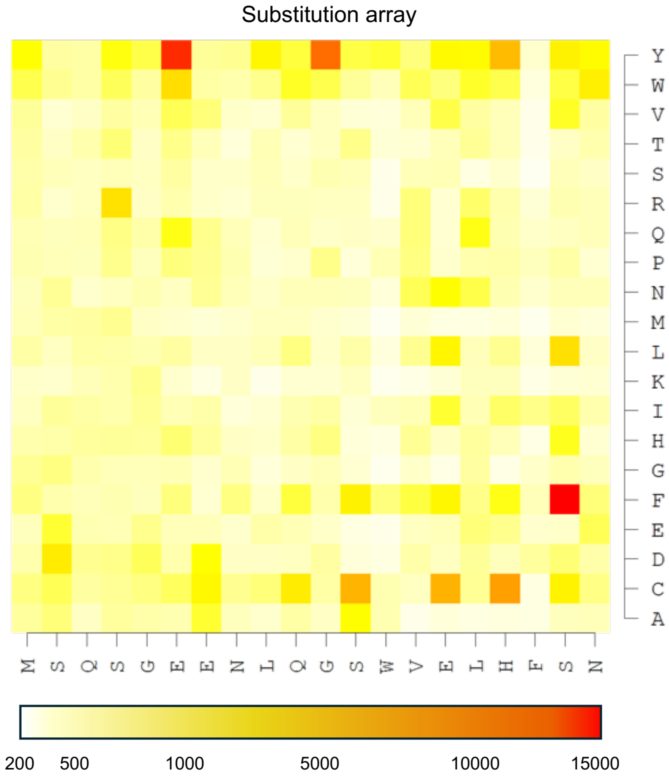
Substitution array
Y
W
V
T
S
R
Q
P
N
M
L
K
I
H
G
F
E
D
C
A
M S Q S G E E N L Q G S W V E L H F S N
200
500
1000
5000
10000
15000

Supplementary Fig. 11. Additional data on BNIP3/BAX interaction.

a, Incubation of fluorescence-labelled recombinant mouse BNIP3 with a library of 65 synthesised peptides representing N-terminal truncations of the BNIP3 sequence MSQSGEENLQGSWVELHFSN (amino acid residues 1-20) immobilised on microarrays revealed that the WVELHFSN sequence resulted in the strongest binding signal, with a 14-fold increase compared to the non-truncated peptide, as shown in the heatmap. (n = 3 identical subarrays with mouse IgG protein controls). **b,** Substitution analysis was performed using fluorescence-labelled recombinant BNIP3 and a library of 379 synthesised peptides immobilised on microarrays. Single residues of the BNIP3 sequence MSQSGEENLQGSWVELHFSN (amino acid residues 1-20) were exchanged for 20 natural amino acids (Ala, Cys, Asp, Glu, Phe, Gly, His, Ile, Lys, Leu, Met, Asn, Pro, Gln, Arg, Ser, Thr, Val, Trp, Tyr). Exchanging Trp-13 and Phe-18 with each of the canonical amino acids impaired BNIP3 binding. The most intense signal was observed when Ser-19 was exchanged for phenylalanine as shown in the heatmap. (n = 3 identical subarrays with mouse IgG protein controls).

a

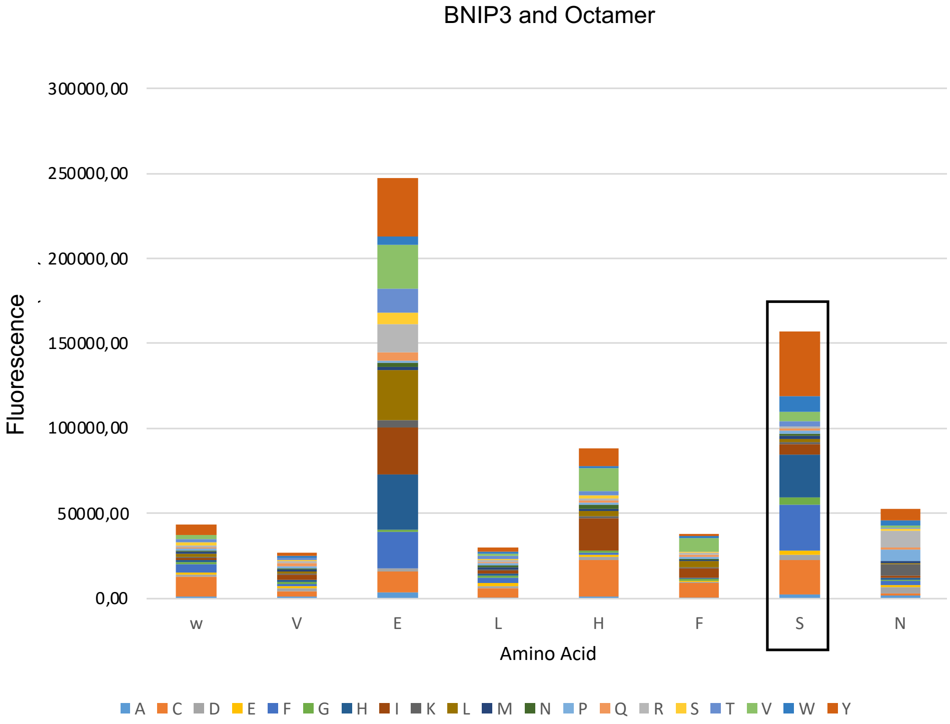

b

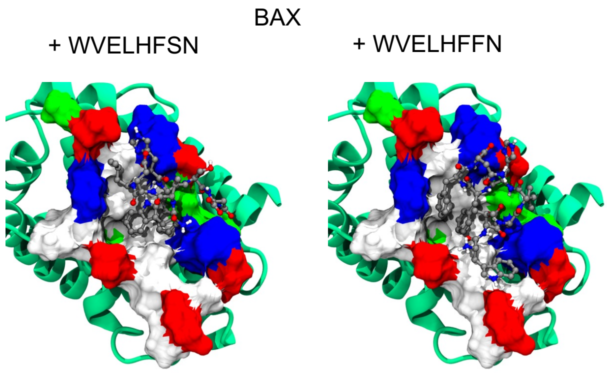

Supplementary Fig. 12. Additional data on peptide binding to BNIP3 and BAX.

a, Substitution analysis was performed using fluorescence-labelled recombinant BNIP3 and also BAX and a library of 156 synthesised peptides immobilised on microarrays. Single residues of the BNIP3 sequence WVELHFSN (amino acid residues 1-20) were exchanged with 20 natural amino acids (Ala, Cys, Asp, Glu, Phe, Gly, His, Ile, Lys, Leu, Met, Asn, Pro, Gln, Arg, Ser, Thr, Val, Trp, Tyr). Exchanging Glu-14 and Ser-19 for the canonical amino acids strengthened the binding affinity to BNIP3. (*n* = 3 identical subarrays with mouse IgG protein controls). **b,** Docking experiments on BAX (PDB code 4S0O) with TAT-WVELHFSN and TAT-WVELHFFN were performed using HADDOCK. Addition of a third aromatic residue results improves intrapeptide aromatic interactions.

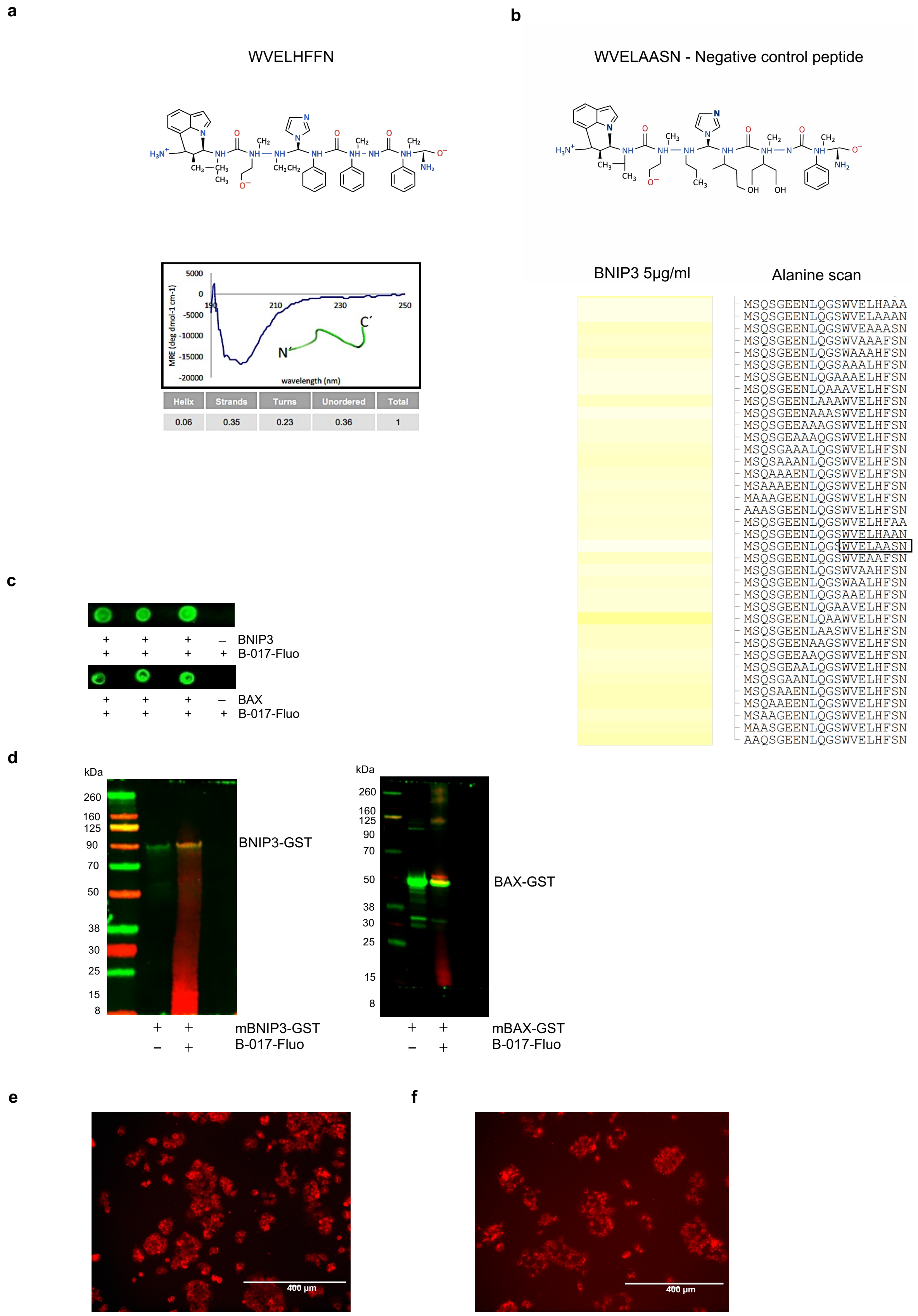

a
WVELHFFN
5000
0
190
210
230
250
-5000
-10000
-15000
-20000
MRE (deg dmol-1 cm-1)
wavelength (nm)
N
C´
Helix
Strands
Turns
Unordered
Total
0.06
0.35
0.23
0.36
1
b
WVELAASN - Negative control peptide
BNIP3 5µg/ml
Alanine scan
MSQSGEENLQGSWVELHAAA
MSQSGEENLQGSWVELAAAN
MSQSGEENLQGSWVEAAASN
MSQSGEENLQGSWVAAAFSN
MSQSGEENLQGSWAAAHFSN
MSQSGEENLQGSAAALHFSN
MSQSGEENLQGAAAELHFSN
MSQSGEENLQAAAVELHFSN
MSQSGEENLAAAWVELHFSN
MSQSGEENAAASWVELHFSN
MSQSGEEAAAGSWVELHFSN
MSQSGEAAAQGSWVELHFSN
MSQSGAAALQGSWVELHFSN
MSQSAAANLQGSWVELHFSN
MSQAAAENLQGSWVELHFSN
MSAAAEENLQGSWVELHFSN
MAAAGEENLQGSWVELHFSN
AAASGEENLQGSWVELHFSN
MSQSGEENLQGSWVELHFAA
MSQSGEENLQGSWVELHAAN
MSQSGEENLQGSWVELAASN
MSQSGEENLQGSWVEAAFSN
MSQSGEENLQGSWVAAHFSN
MSQSGEENLQGSWAALHFSN
MSQSGEENLQGSAAELHFSN
MSQSGEENLQGAAVELHFSN
MSQSGEENLQAAWVELHFSN
MSQSGEENLAASWVELHFSN
MSQSGEENAAGSWVELHFSN
MSQSGEEAAQGSWVELHFSN
MSQSGEAALQGSWVELHFSN
MSQSGAANLQGSWVELHFSN
MSQSAAENLQGSWVELHFSN
MSQAAEENLQGSWVELHFSN
MSAAGEENLQGSWVELHFSN
MAASGEENLQGSWVELHFSN
AAQSGEENLQGSWVELHFSN
c
+ + + – BNIP3
+ + + + B-017-Fluo
+ + + – BAX
+ + + + B-017-Fluo
d
kDa
260
160
125
90
70
50
38
30
25
15
8
BNIP3-GST
+ + mBNIP3-GST
– + B-017-Fluo
kDa
260
160
125
90
70
50
38
30
25
15
8
BAX-GST
+ + mBAX-GST
– + B-017-Fluo
e
400 µm
f
400 µm

Supplementary Fig. 13. B-017 structure and binding to BNIP3 and BAX.

a, Structure of the truncated and substituted sequence WVELHFFN. Since this octamer showed stronger binding to BNIP3 than the non-truncated, non-substituted peptide consisting of the first 20 amino acid residues of BNIP3, we assigned the octamer to a potential functional antagonist of BNIP3 due to its ability to form critical hydrophobic interactions for binding affinity between biomolecules and drugs (top). To enable cellular uptake, we covalently attached the HIV-1 TAT protein transduction domain$_{48\text{-}59}$ (GRKKRRQRRRPQ) as a delivery sequence to the WVELHFFN octamer. Circular dichroism spectroscopy showed a random coil conformation (bottom). **b,** Structure of the designed negative control peptide (top). Alanine scan was performed using fluorescence-labelled recombinant mouse BNIP3 and a library of 19 double-substituted and 18 triple-substituted alanine peptides immobilised on microarrays. No peptide with double or triple alanine substitution demonstrated stronger binding to the protein than the BNIP3 sequence MSQSGEENLQGSWVELHFSN (amino acid residues 1-20), as shown by the heatmap (bottom), which was part of the truncation array. **c,** Images of membranes spotted with recombinant mouse BNIP3-His (top) and recombinant mouse BAX-His (bottom) and incubated with fluorescently labelled B-017 (B-017-Fluo). **d,** Immunoblot analysis showing interaction of recombinant mouse (m) BNIP3-GST (1 µg) (left) and recombinant mBAX-GST (1 µg) (right) with B-017-Fluo (10 ng) after 1 h incubation; representative immunoblot of n = 3 independent experiments. **e,** Representative image of MCF-7 cells showing uptake of Cy5.5-conjugated B-017. Scale bar 200 µm. **f,** Representative image of MCF-7 cells showing uptake of Cy5.5-conjugated negative control peptide. Scale bar, 200 µm.

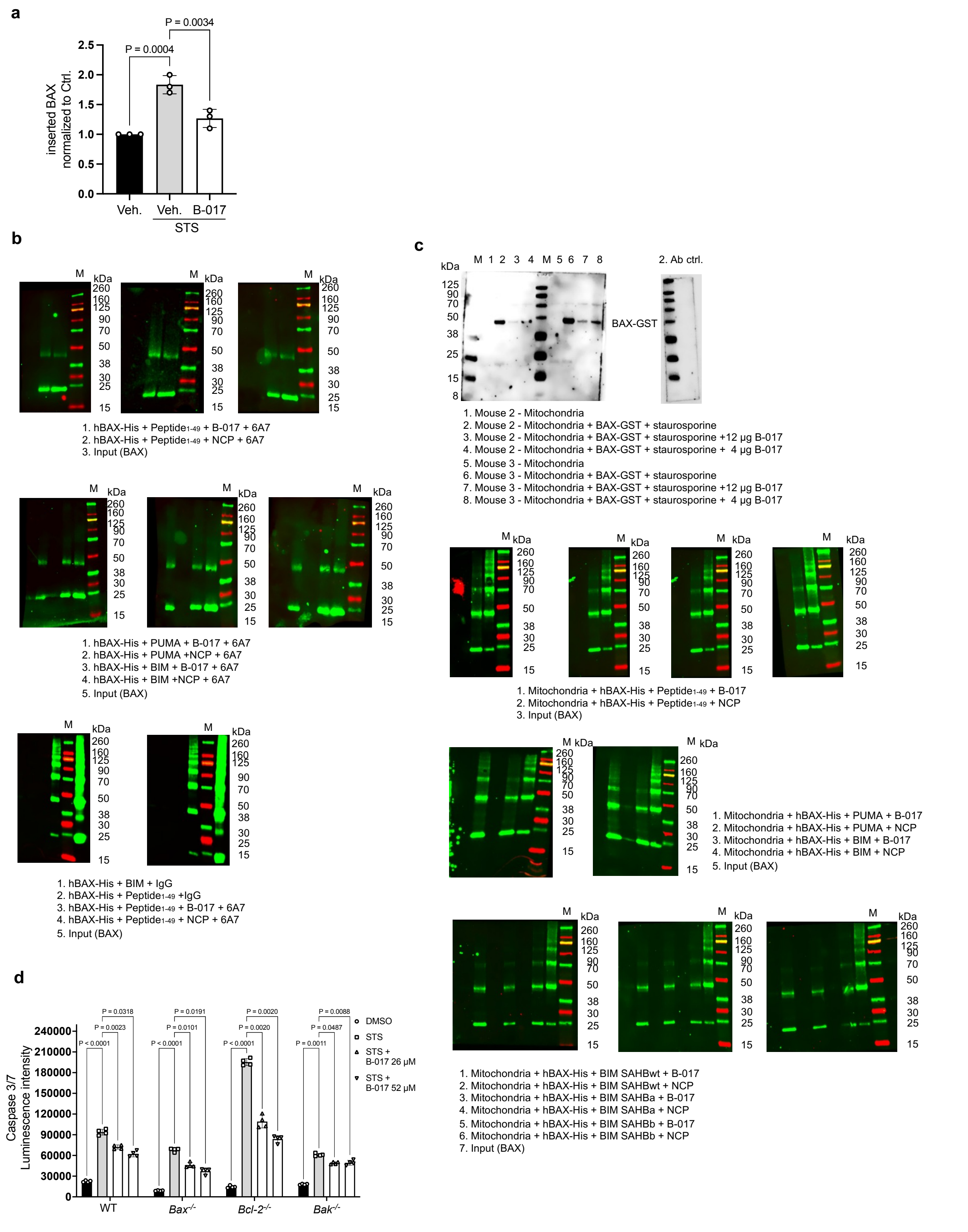
a
P = 0.0034
P = 0.0004
inserted BAX normalized to Ctrl.
Veh.
Veh.
B-017
STS
b
1. hBAX-His + Peptide1-49 + B-017 + 6A7
2. hBAX-His + Peptide1-49 + NCP + 6A7
3. Input (BAX)
1. hBAX-His + PUMA + B-017 + 6A7
2. hBAX-His + PUMA +NCP + 6A7
3. hBAX-His + BIM + B-017 + 6A7
4. hBAX-His + BIM +NCP + 6A7
5. Input (BAX)
1. hBAX-His + BIM + IgG
2. hBAX-His + Peptide1-49 +IgG
3. hBAX-His + Peptide1-49 + B-017 + 6A7
4. hBAX-His + Peptide1-49 + NCP + 6A7
5. Input (BAX)
c
BAX-GST
2. Ab ctrl.
1. Mouse 2 - Mitochondria
2. Mouse 2 - Mitochondria + BAX-GST + staurosporine
3. Mouse 2 - Mitochondria + BAX-GST + staurosporine +12 µg B-017
4. Mouse 2 - Mitochondria + BAX-GST + staurosporine + 4 µg B-017
5. Mouse 3 - Mitochondria
6. Mouse 3 - Mitochondria + BAX-GST + staurosporine
7. Mouse 3 - Mitochondria + BAX-GST + staurosporine +12 µg B-017
8. Mouse 3 - Mitochondria + BAX-GST + staurosporine + 4 µg B-017
1. Mitochondria + hBAX-His + Peptide1-49 + B-017
2. Mitochondria + hBAX-His + Peptide1-49 + NCP
3. Input (BAX)
1. Mitochondria + hBAX-His + PUMA + B-017
2. Mitochondria + hBAX-His + PUMA + NCP
3. Mitochondria + hBAX-His + BIM + B-017
4. Mitochondria + hBAX-His + BIM + NCP
5. Input (BAX)
1. Mitochondria + hBAX-His + BIM SAHBwt + B-017
2. Mitochondria + hBAX-His + BIM SAHBwt + NCP
3. Mitochondria + hBAX-His + BIM SAHBa + B-017
4. Mitochondria + hBAX-His + BIM SAHBa + NCP
5. Mitochondria + hBAX-His + BIM SAHBb + B-017
6. Mitochondria + hBAX-His + BIM SAHBb + NCP
7. Input (BAX)
d
Caspase 3/7 Luminescence intensity
DMSO
STS
STS + B-017 26 µM
STS + B-017 52 µM
WT
Bax-/-
Bcl-2-/-
Bak-/-

Supplementary Fig. 14. Additional data on the impact of B-017 on BAX activation.

a, MCF-7 cells (3x10^6 cells) were exposed to staurosporine (STS) (2 μM) and B-017 (160 μM), and vehicle (Veh.). To assess BAX insertion, mitochondria were isolated after 2 h of incubation and subjected to alkaline extraction to remove loosely attached BAX. Immunoblot analyses showed that B-017 prevents BAX insertion. (n = 3 independent experiments, one-way ANOVA). **b, c,** Immunoblots of activated and inserted BAX related to Fig. 3b and c. **d**, Response to B-017 in staurosporine-treated wild-type mouse embryonic fibroblasts (WT MEFs) and MEFs lacking BCL-2 family members in terms of late-stage cell death signalling. Deletion of *Bax* resulted in decreased caspase activity compared to WT MEFs when exposed to STS (500 nM) for 4 h, an effect that was mitigated by B-017. MEFs lacking the anti-apoptotic protein BCL-2 exhibited a substantial increase in caspase activity, which was predominately alleviated by co-treatment with B-017. Knockout of *Bak* in MEFs also led to the expected reduction in caspase activity, which was not significantly attenuated by B-017. (Experiment with 4 technical replicates related to Fig. 3d; two-way ANOVA). Source data are provided as a Source Data file.

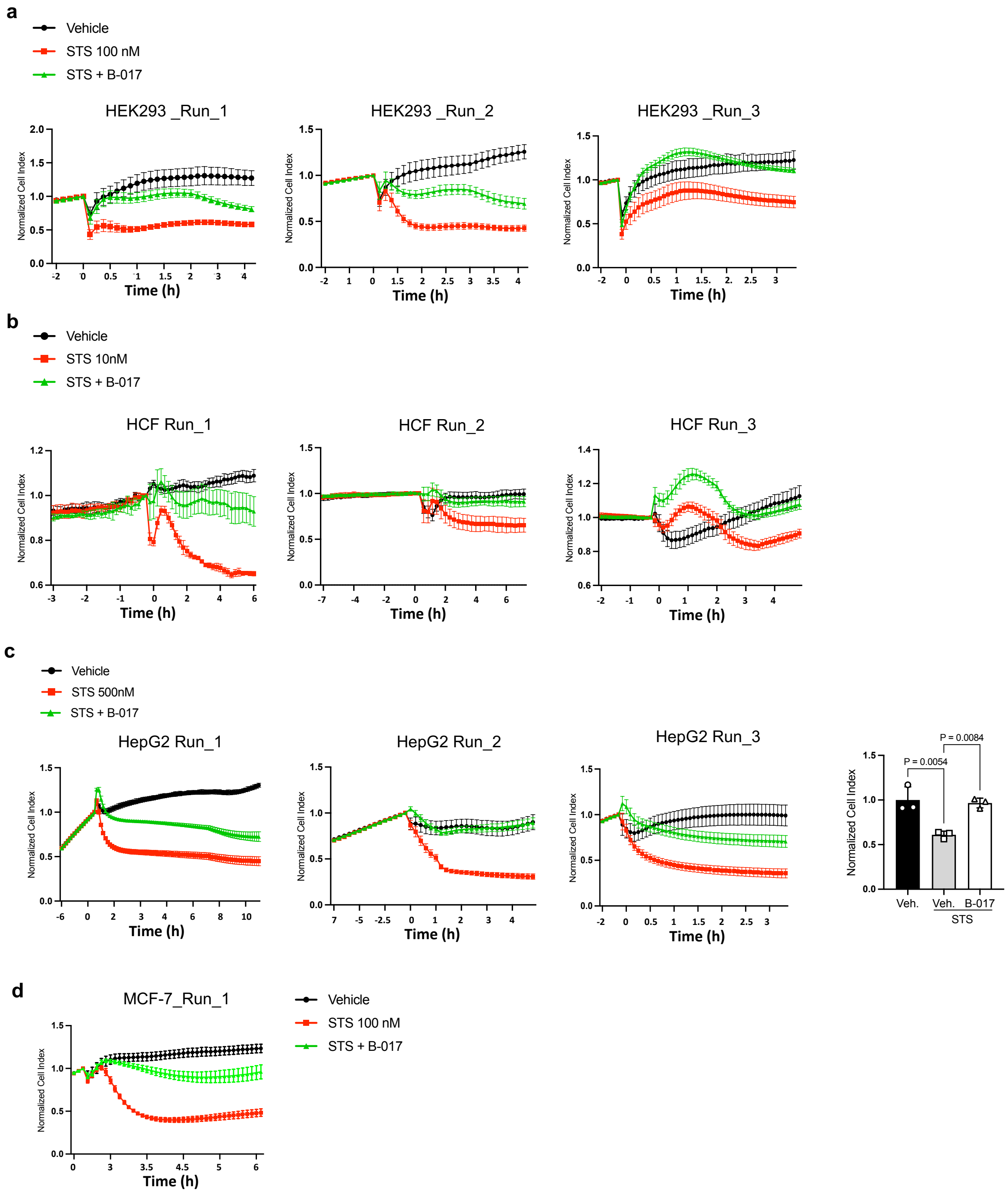

Supplementary Fig. 15. Additional data on B-017 protects cell viability.

a, b, Additional data to cell adhesion experiments in human cardiac fibroblasts (HCF) and HEK293 cells. **c, d,** HepG2 (12,000 cells per well) and MCF-7 cells (8,000 cells per well) were simultaneously treated with 500 nM or 100 nM staurosporine (STS) and B-017 (160 µM) or vehicle (Veh.) after reaching a cell index of 1. Live cell impedance measurements showed that B-017 rescued the viability of HepG2 cells (**c**) and MCF-7 cells (**d**), which is impaired by STS treatment, as cell adhesion rates over time displayed a similar pattern to cells treated with vehicle alone. The time point chosen to assess the differences was 1 h after treatment for HepG2 cells to exclude a proliferation effect. (n = 3 independent experiments in HCF, HEK293 and HepG2 cells, one-way ANOVA, n = 1 experiment in MCF-7).

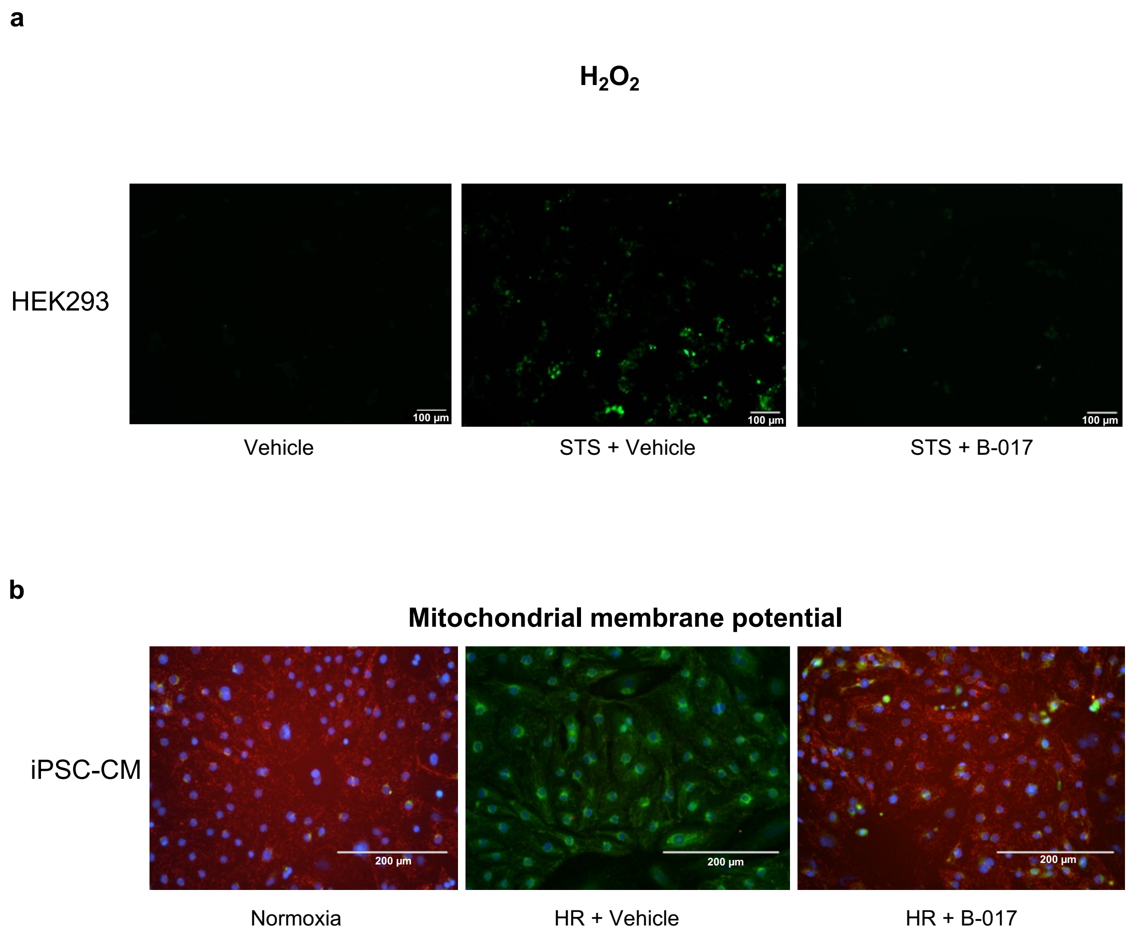

Supplementary Fig. 16. Additional data on B-017 protects cell viability.

a, Representative fluorescence micrographs for assessing hydrogen peroxide in HEK293 cells exposed to staurosporine (STS) with vehicle or B-017 treatment, related to Fig. 4c. **b,** Representative fluorescence micrographs for assessing mitochondrial membrane potential in induced pluripotent stem cell (iPSC)-derived cardiomyocytes exposed to normoxia or hypoxia/reoxygenation (HR) and treated with vehicle or B-017, related to Fig. 4f right.

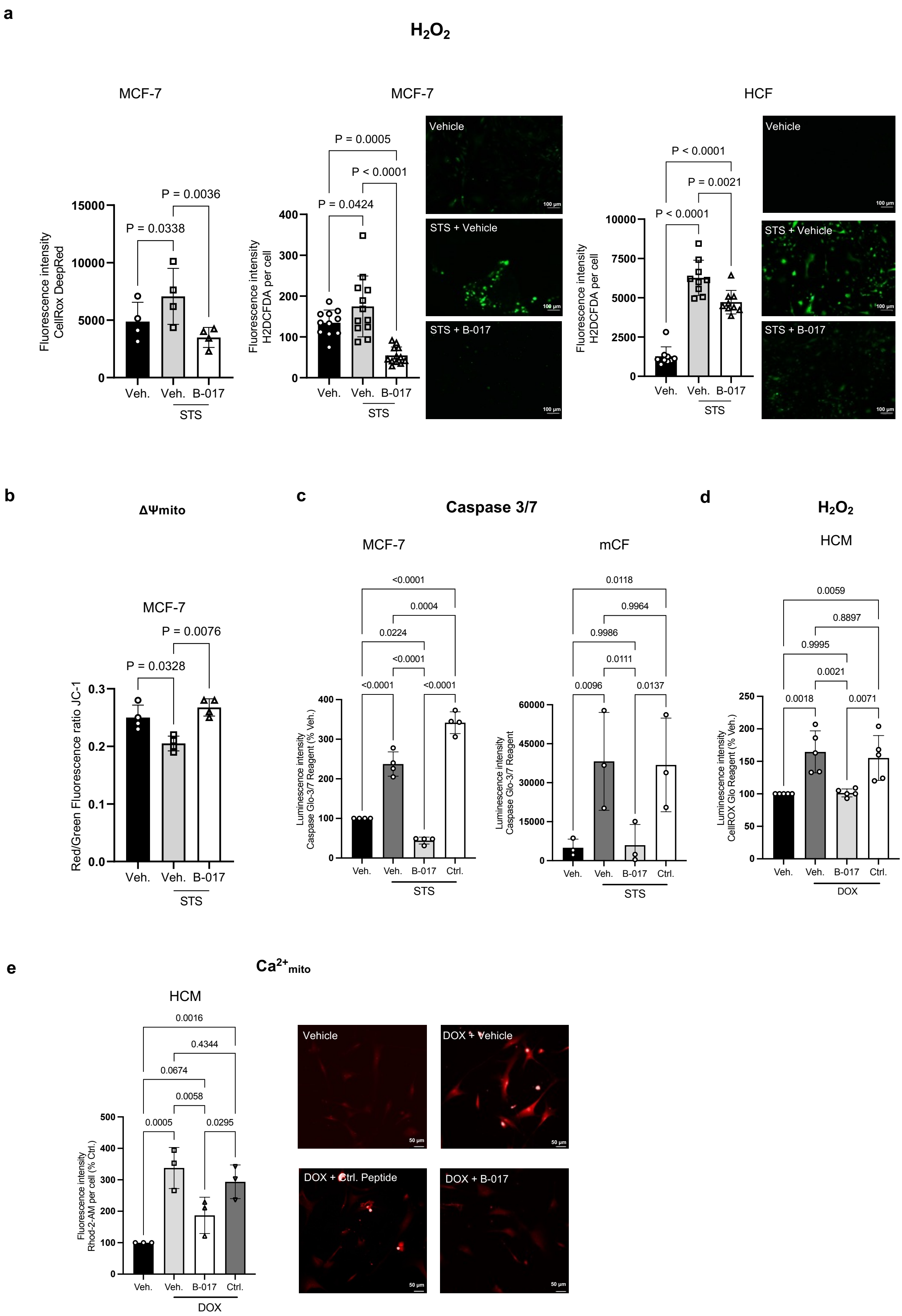

a
H2O2
MCF-7
MCF-7
HCF
Fluorescence intensity CellRox DeepRed
P = 0.0036
P = 0.0338
Veh.
Veh.
B-017
STS
Fluorescence intensity H2DCFDA per cell
P = 0.0005
P < 0.0001
P = 0.0424
Vehicle
STS + Vehicle
STS + B-017
Fluorescence intensity H2DCFDA per cell
P < 0.0001
P = 0.0021
P < 0.0001
b
ΔΨmito
MCF-7
P = 0.0076
P = 0.0328
Red/Green Fluorescence ratio JC-1
c
Caspase 3/7
MCF-7
mCF
Luminescence intensity Caspase Glo-3/7 Reagent (% Veh.)
Luminescence intensity Caspase Glo-3/7 Reagent
Ctrl.
d
H2O2
HCM
Luminescence intensity CellROX Glo Reagent (% Veh.)
DOX
e
Ca2+mito
HCM
Fluorescence intensity Rhod-2-AM per cell (% Ctrl.)
DOX + Vehicle
DOX + Ctrl. Peptide
DOX + B-017

Supplementary Fig. 17. Additional data on B-017 protects mitochondrial health.

a, b, Additional data on cellular consequences of B-017 treatment in MCF-7 cells and human cardiac fibroblasts (HCF) in detail. Staurosporine (STS) was chosen as cell death signalling inducing-agent (500 nM, 2 µM). B-017 (52 µM) or vehicle (Veh.) was administered concomitantly with the stimulus. Cell death signalling characterised by reactive oxygen species generation (ROS) and mitochondrial membrane depolarisation ($\Delta\Psi_{mito}$) were determined. The fluorescence intensity of CellROX DeepRed and JC-1 ($\Delta\Psi_{mito}$) was assessed using a fluorescence microplate reader and H_2DCFDA (H_2O_2) by fluorescence microscopy. B-017 significantly prevented STS-induced cell death signalling as evidenced by decreased generation of ROS in MCF-7 (**a**, left and middle) and HCF (**a**, right) and mitochondrial membrane depolarisation in MCF-7 (**b**). (n = 1 experiment with 4 and 12 technical replicates in MCF-7 and 9 technical replicates in HCF, one-way ANOVA). **c-e**, Additional data on cellular consequences of B-017 treatment in MCF-7 and mouse cardiac fibroblasts (mCF) related to caspase 3/7 activity upon treatment with staurosporine (**c**), and in human cardiomyocytes related to ROS (H_2O_2) generation (**d**) and mitochondrial calcium level upon treatment with doxorubicin (**e**). The negative control peptide was used as control to evaluate a potential impact of the TAT sequence and a peptide itself (n = 3 independent experiments, two-way ANOVA). Source data are provided as a Source Data file.

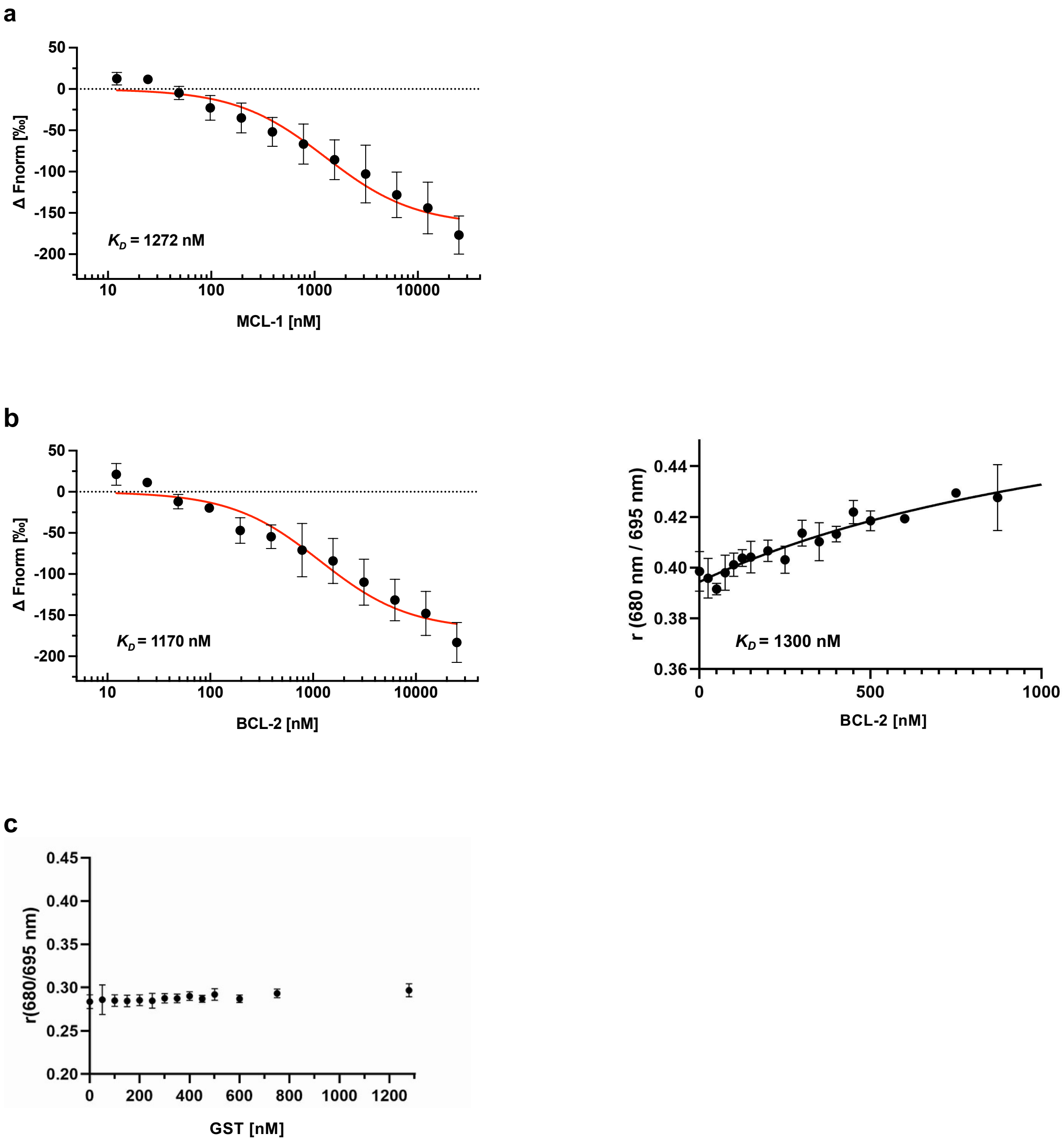

Supplementary Fig. 18. Dose-response curves of the binding of the proteins MCL-1, BCL-2, and glutathione-S-transferase to Cy5.5-labelled peptide.

Microscale thermophoresis dose-response curves for the binding of Cy5.5-labelled B-017 to the human proteins (**a**) MCL-1 and (**b, left**) BCL-2. A constant concentration of 200 nM fluorescence-labelled B-017 was incubated with serial dilutions of each protein (12.2–25,000 nM). Normalised fluorescence changes were plotted as a function of protein concentration. To determine binding affinities (K_D), nonlinear regression analysis was performed using a one-site binding model. Data points represent the mean±SD from three biological replicates (n = 3), and solid red lines show the fitted binding curves. **b, c,** Fluorescence anisotropy measurement (680/695 nm) with titration of increasing human BCL-2 (**b, right**) or glutathione-S-transferase (GST) concentrations (**c**) to 100 nM Cy5.5-labelled B-017. All titrations were performed in n = 3 technical replicates and data points are shown as means ± SD based on the three independent experiments (n = 3). Binding curves of the averaged data were fitted with GraphPad Prism 10.0 (GraphPad) using the quadratic binding equation for a one-site specific binding model and K_D values are given as fit of the averaged data points ± standard deviation of the fit.

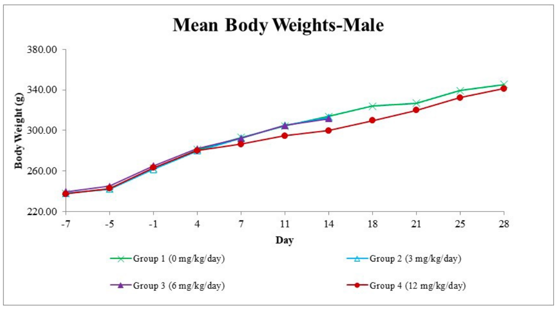

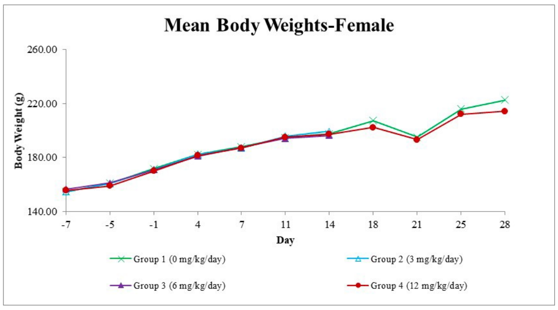

Supplementary Fig. 19. Impact of B-017 on body weight in male and female rats.

The animals received four different doses of B-017 (0, 3, 6 or 12 mg kg^{-1} body weight) for 14 days, followed by a 14-day recovery phase. Neither sex showed any loss of body weight during the dosing or recovery phases of the B-017 treatment.

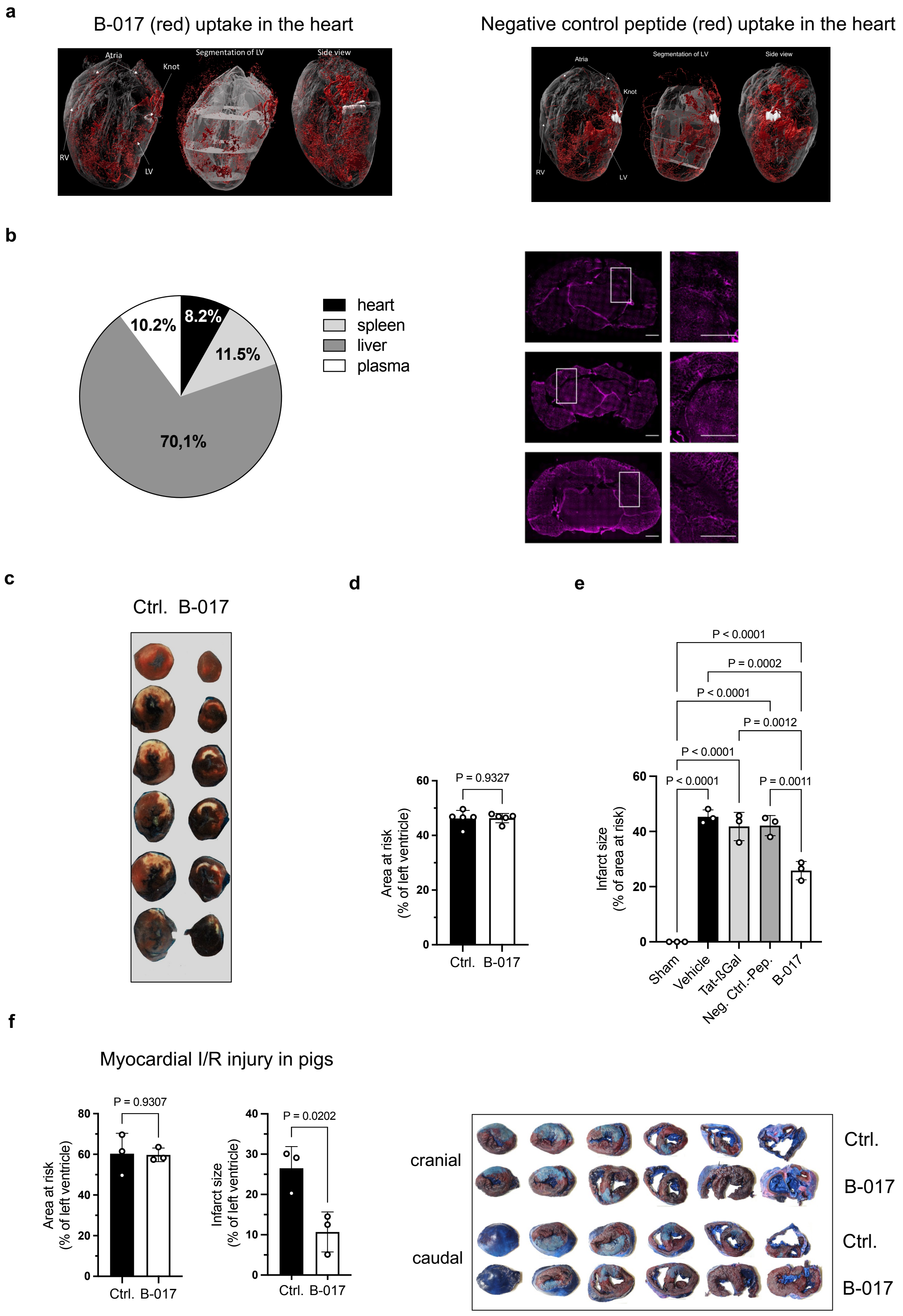
a
B-017 (red) uptake in the heart
Atria
Knot
Segmentation of LV
Side view
RV
LV
Negative control peptide (red) uptake in the heart
Atria
Knot
Segmentation of LV
Side view
RV
LV
b
8.2%
10.2%
11.5%
70,1%
heart
spleen
liver
plasma
c
Ctrl. B-017
d
P = 0.9327
Area at risk (% of left ventricle)
60
40
20
0
Ctrl. B-017
e
P < 0.0001
P = 0.0002
P < 0.0001
P = 0.0012
P < 0.0001
P < 0.0001
P = 0.0011
Infarct size (% of area at risk)
60
40
20
0
Sham
Vehicle
Tat-ßGal
Neg. Ctrl.-Pep.
B-017
f
Myocardial I/R injury in pigs
P = 0.9307
Area at risk (% of left ventricle)
80
60
40
20
0
Ctrl. B-017
P = 0.0202
Infarct size (% of left ventricle)
40
30
20
10
0
Ctrl. B-017
cranial
caudal
Ctrl.
B-017
Ctrl.
B-017

Supplementary Fig. 20. Additional data on B-017 induced protective biological responses in ischaemia/reperfusion injury.

a, Representative 3D visualisation of B-017-Cy5.5 (red, left) and negative control peptide-Cy5.5 (red, right) distribution across a whole mouse heart after 30 min of ischaemia with 5 min of reperfusion with endogenous autofluorescence (green). Ischaemic wild-type male mice were initially injected with fluorescence-labelled B-017 or negative control peptide-Cy5.5 5 min prior to reperfusion. As in the light sheet microscopic analysis of the whole heart, the distribution of B-017 and the negative control peptide was remote and within the region, which was blocked from blood flow through ligation of the left coronary artery (knot), the so-called area at risk (AAR). LV, left ventricle; RV, right ventricle. (n = 2 C57BL/6J male mice). **b,** Uptake of Lys(5(6)-FAM-labelled B-017 in different mouse organs at 5 min reperfusion following 30 min of myocardial ischaemia. The peptide was administered into the LV cavity 5 min before reperfusion (**left**) (n = 3 C57BL/6J male mice). Representative micrographs showing uptake of Lys(5(6)-FAM-labelled B-017 in the brain at 30 min ischaemia. The peptide was administered intravenously at the onset of ischaemia (**right**) (n = 3 C57BL/6J male mice). (Scale bars 1,000 µm). **c,** Representative tetrazolium chloride (TTC)-stained slices of male mouse hearts subjected to 30 min of ischaemia followed by 24 h of reperfusion with vehicle (Ctrl.) or B-017 (0.8 mg kg^{-1} BW) administered. (Blue (remote area) and red (AAR), live tissue; white, infarcted tissue). **d**, Experimental occlusion of the left coronary artery induces ischaemia downstream of the ligation, namely the AAR. For comparison of dead tissue per AAR, it is a prerequisite that the risk zone is similar in all animals. Measurement of the AAR in the vehicle (Ctrl.) and B-017 treated group are similar (left) (n = 5 C57BL/6J mice per group, two-tailed Student's t-test). **e,** Therapeutic response to various control treatments and B-017 in mice exposed to 30 min of ischaemia followed by 24 h of reperfusion. Mice were injected with vehicle (0.9% sodium chloride, NaCl), TAT-ßGal, and the negative control peptide TAT-WVELAASN or B-017 administered into the LV cavity 5 min prior to reperfusion. TAT-ßGal served as a control for the TAT-sequence in B-017, and TAT-WVELAASN for the peptide drug class. Sham mice served as control. Infarct size measurement (infarcted tissue per AAR) showed no difference between vehicle, TAT-ßGal, and negative control peptide treatments and no protective effects. B-017 significantly reduced infarct size by 40% relative to the control treatments (n = 3 C57BL/6J mice per group, one-way ANOVA). **f,** Therapeutic response to B-017 in pigs exposed to 60 min of ischaemia and 4 h of reperfusion. Pigs were injected with vehicle (0.9% sodium chloride, Ctrl.) or B-017 (0.075 mg kg^{-1} BW), administered *via* intravenous bolus injection 5 min prior to reperfusion. No difference in the AAR was observed (left). Infarct size measurement (infarcted tissue per AAR) showed that B-017 significantly reduced infarct size per left ventricle (LV; right) compared to vehicle treatment (middle). Representative tetrazolium chloride (TTC)-stained slices of pig hearts subjected to 60 min of ischaemia followed by 4 h of reperfusion with vehicle or B-017 (0.075 mg kg^{-1} BW) administered. (n = 3 pigs per group, two-tailed Student's t-test). Source data are provided as a Source Data file.

a

Impact of B-017 on basal LV-function in mice

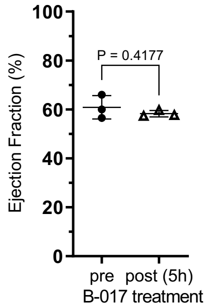

b

Long-term blood pressure measurements in dogs

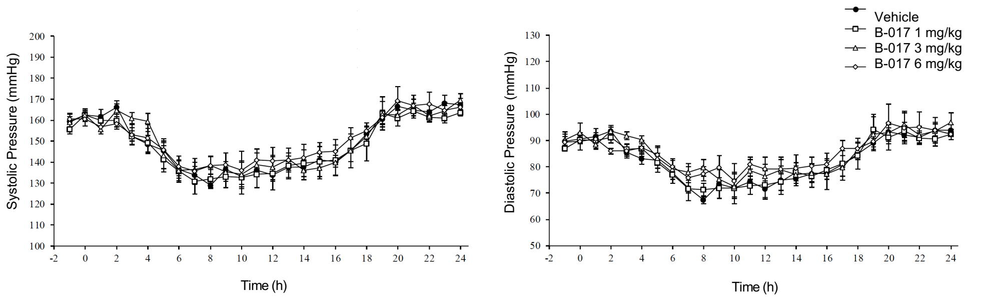

Long-term measurements of mean arterial pressure and heart rate in dogs

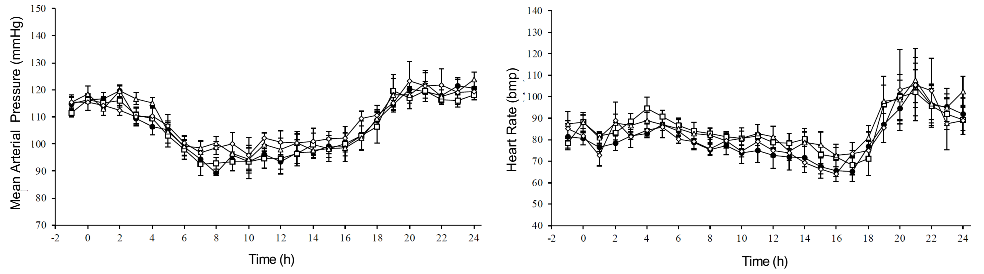

c

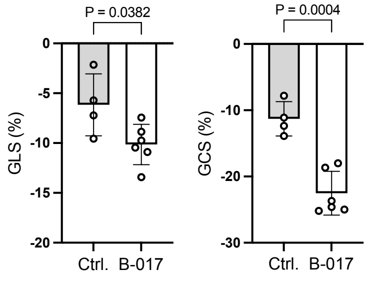

Supplementary Fig. 21. Additional data on impact of B-017 on left ventricular function and haemodynamics.

a, Therapeutic response of basal cardiac function to B-017 in mice. Mice were injected B-017 (0.8 mg kg^{-1} BW). Changes in cardiac function were monitored by echocardiography baseline and 5 h post treatment. At the end of 5 h, B-017 showed no impact on cardiac function, as indicated by left ventricular ejection fraction (LVEF) calculated by Simpson's method (n = 3 C57BL/6J male mice per group, two-tailed Student's t-test). **b,** Therapeutic response of blood pressure and heart rate to 3 doses of B-017 (1, 3 and 6 mg kg^{-1} BW) in male dogs. Parameters were examined in 4 conscious telemetered dogs prior to and following intravenous administration of vehicle (0.9% sodium chloride, Ctrl.) or B-017 and showed no effect of B-017 on systolic, diastolic, or mean arterial pressure and heart rate compared to respective pre-dose baseline values. Group mean values followed a similar pattern to the control group (n = 3 male dogs per group). **c,** Therapeutic response of left ventricular function to B-017. Mice were injected with either vehicle (Ctrl.) or B-017 (0.8 mg kg^{-1} BW) 5 min before reperfusion and on d1, d3, d5, and d7 after ischaemia. At the end of the 28-days follow-up, discrete changes in myocardial contraction speckle-tracking-based strain analysis were assessed. The global longitudinal strain and global circumferential strain (GRS, GCS), measured midventricular, were impaired by ischaemia/reperfusion injury and protected by B-017 treatment. (n = 4 Ctrl. and n = 6 B-017-treated C57BL/6J male mice per group, two-tailed Student's t-test).

a

Stroke in mice

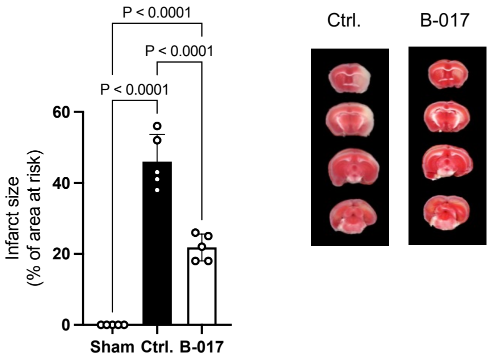

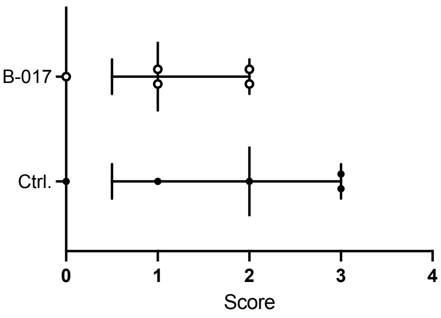

Score signs

0 Normal
1 Flexion of the left front paw
2 Flexion of the left front paw and decreased resistance to lateral push
3 Circling to one side
4 Circling and spinning around the cranial-caudal axis, loss of walking or righting reflex
5 Comatose or moribund

b

Liver I/R injury in rats

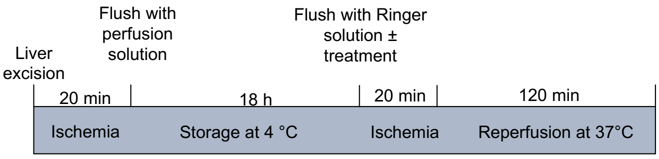

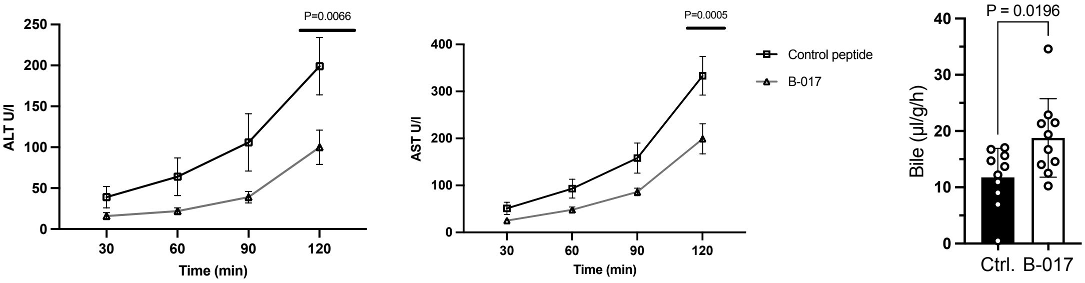

Supplementary Fig. 22. Additional data on B-017 induces protective biological responses in ischaemia/reperfusion injury.

a, Therapeutic response to B-017 in mice exposed to brain ischaemia/reperfusion. Mice were subjected to 30 min of transient middle cerebral artery occlusion (tMCAO) followed by reperfusion and treated with B-017 (0.8 mg kg^{-1}) or vehicle (Ctrl.) immediately prior to ischaemia. Sham operated mice served as controls. Stroke volumes assessed by TTC staining 24 h after tMCAO (right) showed that B-017 markedly reduced infarct size by 52% compared to controls (n = 5 C57BL/6 male mice per group, one-way ANOVA). The Bederson Score assessment 24 hours postoperative in control group and B-017 treatment group tended to show fewer neurological deficits under B-017 treatment. (n = 5 C57BL/6J male mice per group, Student's t-test, P = 0.4112). **b,** Therapeutic effect of B-017 on ischaemia/reperfusion in male rat liver explanted for organ transplant. Timeline of the *ex vivo* liver ischaemia/reperfusion model (top). After excision during the 20 min of ischaemia after cardiac arrest, rat livers were cold stored for 18 h, flushed with 20 ml of saline with or without B-017, kept at room temperature for 20 min (simulating surgical implantation), and then reperfused at 37 °C for 120 min**.** Measurement of aspartate aminotransferase (AST) and alanine transaminase (ALT) levels showed that B-017 reduced tissue injury at 120 min reperfusion, as evidenced by significantly lower AST (left) and ALT (right) levels in the circulating perfusion solution compared to negative control peptide treatment (Ctrl.). B-017 improved also functional recovery as shown by higher hepatic bile production during reperfusion compared to control. Total bile secretion was collected by inserting a 27-gauge polyethylene tubing into the common bile duct. Hepatic bile production was calculated as µl g^{-1} h^{-1}. (n = 6 male rats per group, two-way ANOVA; n = 9 male rats per group, two-tailed Student's t-test). Source data are provided as a Source Data file.

a

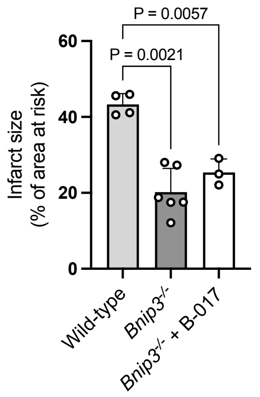
P = 0.0057
P = 0.0021
60
40
20
0
Infarct size
(% of area at risk)
Wild-type
Bnip3-/-
Bnip3-/- + B-017

b

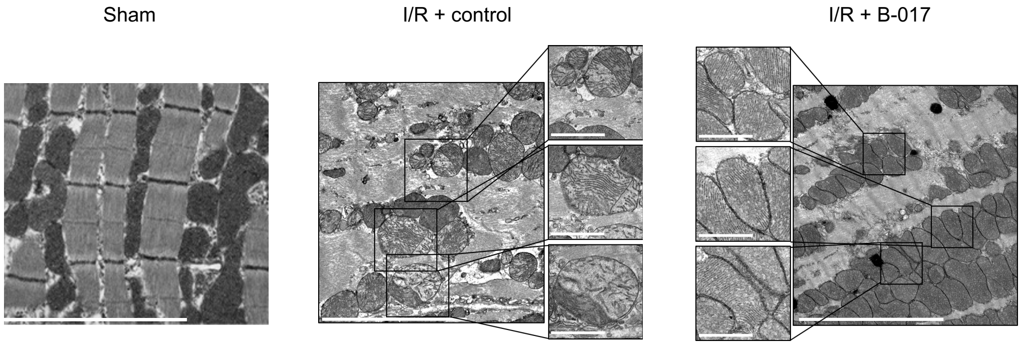
Sham
I/R + control
I/R + B-017

c

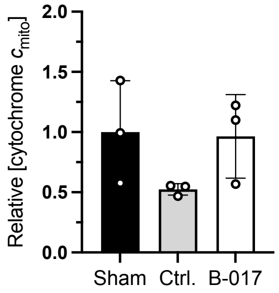
2.0
1.5
1.0
0.5
0.0
Relative [cytochrome c mito]
Sham
Ctrl.
B-017

Supplementary Fig. 23. Additional data on impact of B-017 on cell death signalling in ischaemia/reperfusion injury.

a, Therapeutic response to the intracardiac injection of B-017 in *Bnip3* knockout (*Bnip3*$^{-/-}$) mice, compared to untreated wild-type and *Bnip3*$^{-/-}$ mice, in ischaemia/reperfusion (I/R). Mice were exposed to 30 min of ischaemia, followed by 24 h of reperfusion. Infarct size measurement (infarcted tissue per AAR) showed that *Bnip3* deletion generally protected cardiac tissue from I/R injury, as evidenced by a 51% reduction in infarct size compared to wild-type mice. B-017 treatment provided no additional protection (*n* = 4 C57BL/6J untreated male mice, *n* = 6 untreated *Bnip3*$^{-/-}$ male mice, *n* = B-017-treated *Bnip3*$^{-/-}$ male mice, one-way ANOVA). **b,** Representative electron micrographs depicting swollen mitochondria in the area at risk in negative control peptide-treated mice related to Fig. 6a. Scale bars, 2 µm, 1 µm, n = 3 C57BL/6 male mice. **c,** Immunoblot analysis of mitochondrial cytochrome *c* levels in the AAR showed a tendency towards higher levels under B-017 treatment compared to those treated with the negative control peptide. (*n* = 3 C57BL/6J male mice, one-way ANOVA). Source data are provided as a Source Data file.

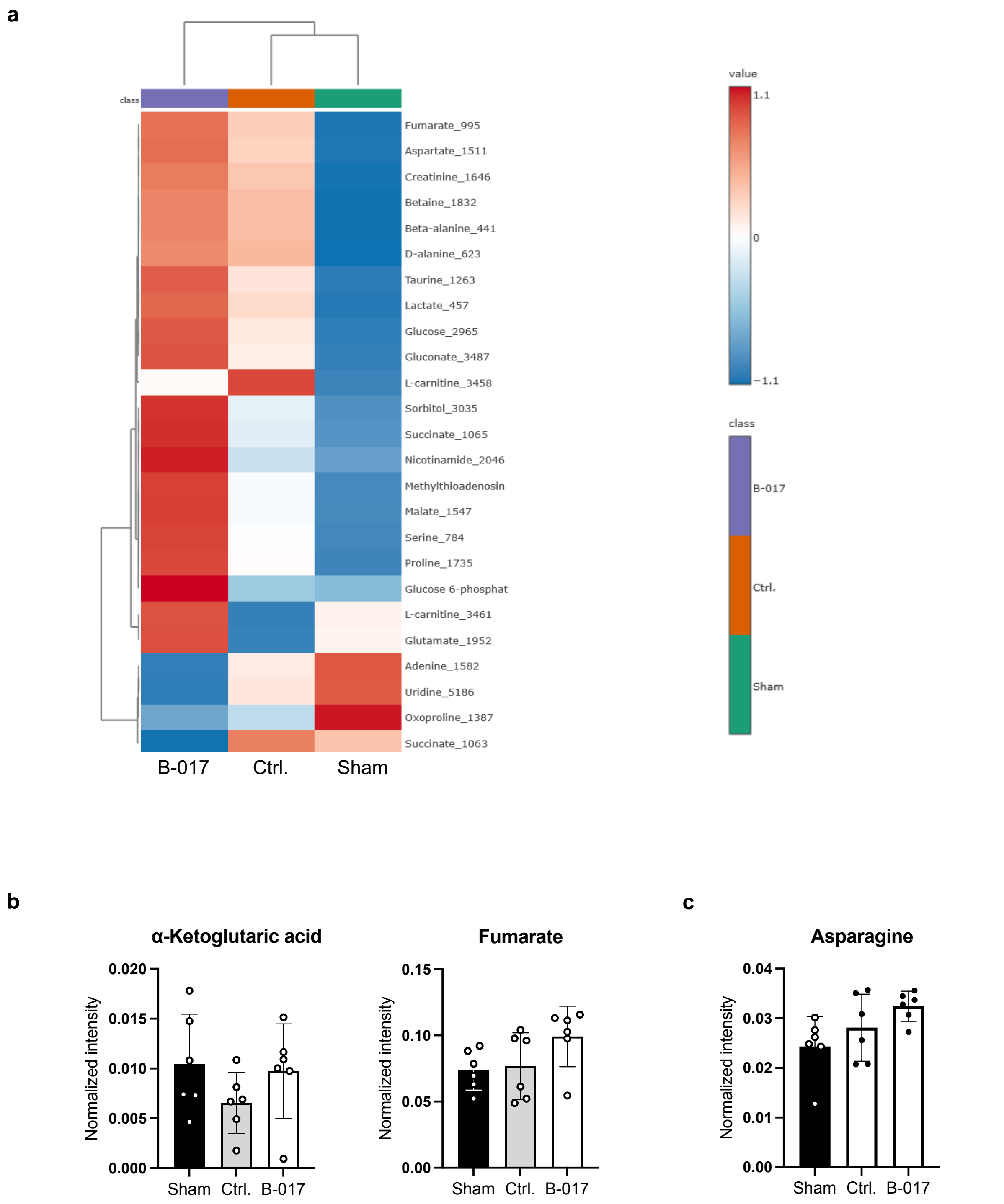

Supplementary Fig. 24. Additional data on impact of B-017 on cardiac metabolism.

a-c, Altered cardiac metabolomics after 60 min of reperfusion following 30 min of ischaemia in the B-017-treated group compared with control. **a,** Heatmap of cardiac metabolomics based on a principal component analysis showing the top 25 metabolites that are different between B-017-treated, control and sham group in the setting of ischaemia/reperfusion injury. **b,** Levels of altered TCA intermediates α-ketoglutarate and fumarate and **c,** levels of the amino acid asparagine altered by B-017 treatment compared to control and sham group. (*n* = 6 C57BL/6J male mice per group).

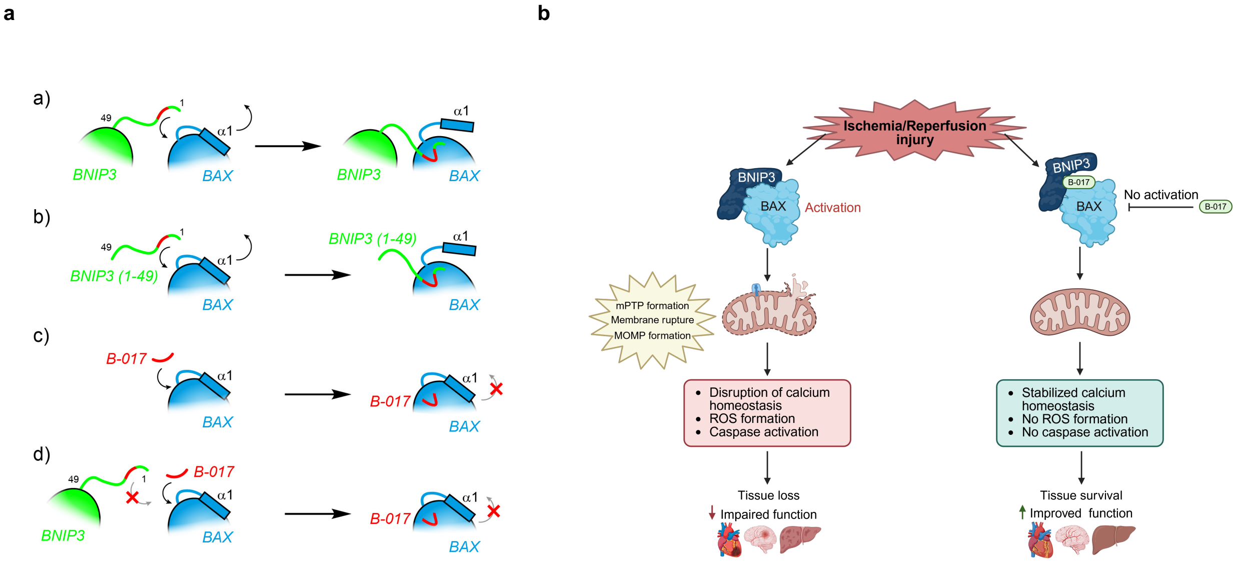

Supplementary Fig. 25. Mode of action of B-017.

a, Schematic diagram showing the proposed mechanism of B-017 illustrating (**a**) the functional domain of full-length BNIP3 activating BAX, exposing the transmembrane domain for insertion into the mitochondrial outer membrane (MOM), created with Adobe Illustrator. (**b**) binding of the functional domain of BNIP3 as BNIP3-peptide$_{1-49}$ activating BAX, exposing the transmembrane for insertion into the MOM, (**c**) binding of B-017 to BAX with no activation of BAX, and (**d**) competition of B-017 with the BNIP3 activating domain, preventing the activation of BAX. **b**, Schematic diagram showing the impact of B-017 on the mitochondrial protection pathway. BNIP3 plays a crucial role in BAX-induced mitochondrial damage. Direct activation of BAX by BNIP3 leads to the perturbation of the mitochondrial membranes, resulting in mitochondrial outer membrane rupture or pore formation in the mitochondrial outer membrane. These culminates in the initiation of apoptotic and necrotic cell death, which has a dramatic effect on cardiac function. (mPTP, mitochondrial permeability transition pore; MOMP, mitochondrial outer membrane pore). Created in BioRender. Roth, A. (2026). BioRender.com/XE29MK0Y32

Source Data

Supplementary Fig. 14a

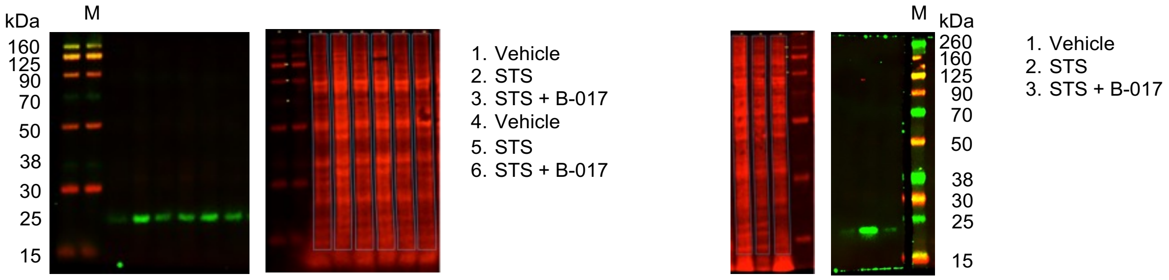

one-way ANOVA		Groups
P value	0.0004	Vehicle vs. Vehicle + STS
P value	0.0154	Vehicle + STS vs. B-017 + STS

Supplementary Fig. 23c

Negative control peptide	n = 3
B-017	n = 3

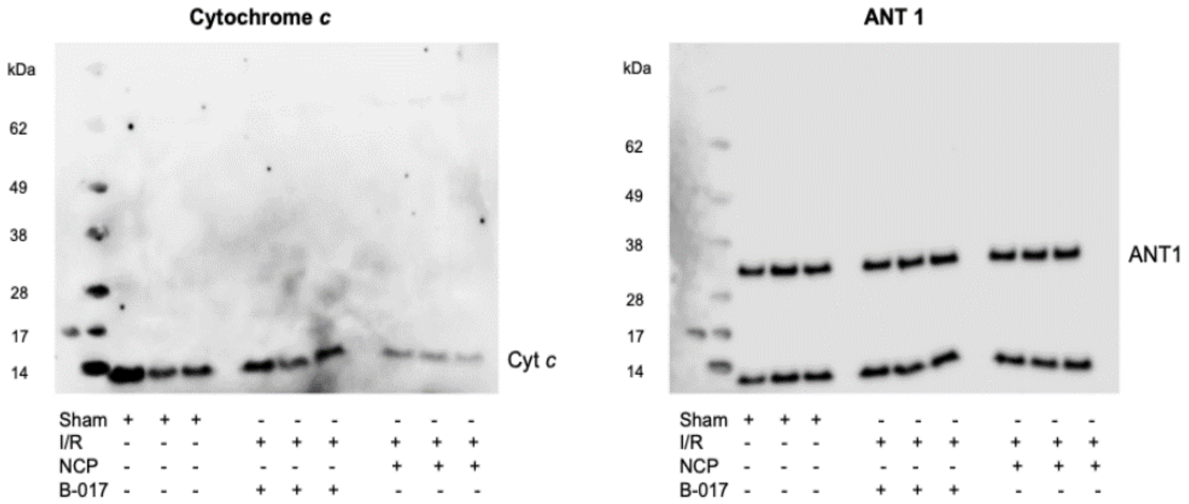

Supplement: Supplementary file 1 — Supplementary Information [file 41467_2026_73993_MOESM1_ESM.pdf]
