# Supplementary material for: Reverse engineering of BNIP3 identifies a mitochondrial protective peptide
Source: Nat Commun. 2026 Jun 17;17:5359. doi: 10.1038/s41467-026-73993-2 (PMC13275919; doi:10.1038/s41467-026-73993-2)
Supplement: Supplementary file 2 — Description of Additional Supplementary Files [file 41467_2026_73993_MOESM2_ESM.pdf]

Description of Additional Supplementary Files

Supplementary Data 1:

Detailed settings for the CALIBRATION and XLSEARCH related to peptide and protein identification using MetaMorpheus.

Supplementary Data 2:

Serum chemistry in rats – Dosing phase

Supplementary Data 3:

Urine analysis parameters in rats – Dosing phase

Supplementary Data 4:

Histopathology Finding in rats – Dosing phase

Supplementary Data 5:

Histopathological Findings in rats – Recovery Phase

Supplementary Data 6:

Haematological values in rats – Dosing phase
