# Supplementary material for: Reverse engineering of BNIP3 identifies a mitochondrial protective peptide
Source: Nat Commun. 2026 Jun 17;17:5359. doi: 10.1038/s41467-026-73993-2 (PMC13275919; doi:10.1038/s41467-026-73993-2)
Supplement: Supplementary file 3 — Supplementary Data 1 [file 41467_2026_73993_MOESM3_ESM.pdf]

Overview

ACE project	Title
ACE_0836	Chemical Crosslinking of the BAX:BNIP3 complex and the BAX and BNIP3 homodimers with DSSO
ACE_0880	Photo-Crosslinking of the cell-protective Peptide B (with BpA) to BAX and BNIP3

ACE_0836

File legend

ACE ID	Organism	Organ/ cell line	Treatment/ experimental setup
ACE_0836_CB02	*H. sapiens*	Purified recomb. Protein (expressed in E. coli)	25 µM BAX + 10 µM BNIP3 + 50x DSSO
ACE_0836_CB03	H. sapiens	dito	25 µM BAX + 10 µM BNIP3 + 50x DSSO

LC_Settings

MS device	Orbitrap Fusion Lumos
LC device	Thermo Vanquish Neo
ion source	Thermo Nanospray Flex
**Analytical column**	Self-packed fused silica capillary with an integrated sintered frit; CoAnn Technologies ICT36007515F-50-5
column diameter	Length (L_C) = 28 cm; ID = 75µm; OD = 360 µm; emitter 15 µm
stationary phase	Phenomenex Kinetex C18-XB core shell
particle diameter (d_p)	1.7 µm
Pore size	120 Å
Column ID	AC151
Column oven	Sonation column oven PRSO-V2
Column oven temp.	50°C
**solvents**	A: 0.2% FA, 2% ACN, 98% H_2O<br>B: 0.2% FA, 80% ACN, 20 % H_2O
gradient	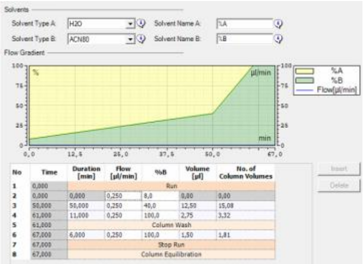

MS_Settings

Project	MS	general	MS1	MS2	MS2	MS3	Comments; special settings
ACE_0836	Lumos	Tune v3.5.3881.18<br>Xcalibur v4.5.445.18<br>Gradient: 67 min	Analyzer: FT<br>Res.: 120000<br>SR: 380 - 1400<br>AGC: Standard<br>AcT: Auto<br>RF: 30<br>SF: --<br>DDM: CT/3sec	Analyzer: FT<br>Res./ScR: 30000/-<br>SR: Auto<br>AGC: 200%<br>AcT: 70 ms<br>CS: +3 to +8<br>IsM: Q<br>IsW: 1.6<br>Frag.: aHCD<br>NCE: 25, 30			classic orbitrap experiment: MS1 in Orbitrap at high resolution and data dependent MS2 also in Orbitrap high resolution. Dynamic exclusion enabled (exclude after n times=1; Exclusion duration (s)= 30; mass tolerance= ± 10ppm)<br><br>Intensity Threshold: 50000<br>Ion transfer Tube Temp: 270 °C<br>Ion Source Voltage: 2200 V

Note: **FT**= Fourier Transform (Orbitrap); **IT**= Iontrap; **Q**= Quadrupol; **Res.**= max. Resolution at 200 m/z (Lumos) or 400 m/z (Elite) [FWHM (full width at half maximum)]; **ScR**= scan rate for measurements in the IT; **SR**= scan range [m/z]; **AGC**= automatic gain control, max number of acquired ions per measurement; **AcT**= max. Ion acquisition time [ms]; **CS**= charge states used for fragmentation; **IsM**= Isolation mode (Q or IT), MS2 isolation and further is only done in IT; **IsW**= Isolation window [m/z], value followed by scan mode the isolation is based on (MS1, MS2 …) **Frag.**= Fragmentation method; **HCD**= Higher-energy collisional dissociation; **CID**= Collision-induced dissociation; **ETD**= Electron-transfer dissociation**; EThcD=** Electron-Transfer/Higher-Energy Collision Dissociation; **sHCD**= stepped HCD**; NCE**= normalized collision energy; **cycles**: number of MSn recorded or max cycle time; RF= RF Lens [%]; **SF**= Source Fragmentation [V]; **DDM**: Data dependent Mode (cycle time in seconds, CT/[s] or number of scans, NS); **NS**= Number of data dependent scans

Search Settings

Program & version	MetaMorpheus v1.0.3.
Search engine	MetaMorpheusXL
settings	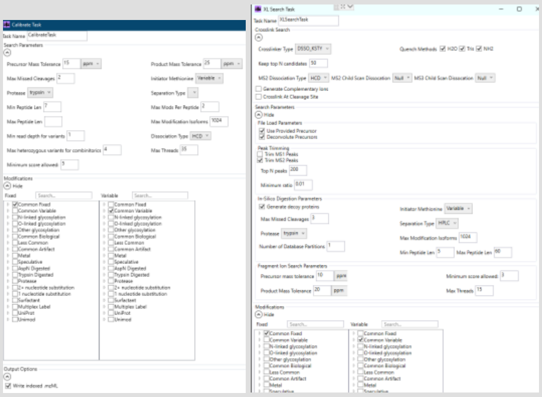
Static modification	Carbamidomethyl (C)
Digestion mode	Trypsin/P (specific), 3 missed cleavages
CL and specificity	DSSO; Site A: **K**; Site B: **KSTY**
Dynamic modification	Oxidation (M)
Custom amino acid defined	
Program & version	1. ACE_0836_SOI_v01.fasta
Annotation	

Note: Database includes contaminants

ACE_0880

File legend

ACE ID	Organism	Organ/ cell line	Treatment/ experimental setup
ACE_0880_CB01	*H. sapiens*	BAX purified recomb. Protein (expressed in E. coli), peptide is synthetic	25 µM hBAX + 25µM Tat-PeptideB(B-017)-BpA
ACE_0880_CB02	*H. sapiens*	dito	25 µM hBAX + 25µM Tat-PeptideB(B-017)-BpA
ACE_0880_CB03	*H. sapiens*	dito	25 µM hBAX + 25µM Tat-PeptideB(B-017)-BpA

LC_Settings

MS device	Orbitrap Fusion Lumos
LC device	Thermo Vanquish Neo
ion source	Thermo Nanospray Flex
**Analytical column**	Self-packed fused silica capillary with an integrated sintered frit; CoAnn Technologies ICT36007515F-50-5
column diameter	Length (L_C) = 28 cm; ID = 75µm; OD = 360 µm; emitter 15 µm
stationary phase	Phenomenex Kinetex C18-XB core shell
particle diameter (d_p)	1.7 µm
Pore size	120 Å
Column ID	AC155
Column oven	Sonation column oven PRSO-V2
Column oven temp.	50°C
**solvents**	A: 0.2% FA, 2% ACN, 98% H_2O<br>B: 0.2% FA, 80% ACN, 20 % H_2O
gradient	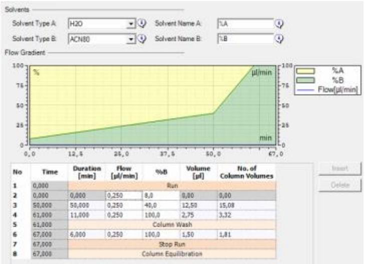

MS_Settings

Project	MS	general	MS1	MS2	MS2	MS3	Comments; special settings
ACE_0880	Lumos	Tune v4.1.4244<br>Xcalibur v4.7.69.37<br>SII: 1.7.0.468<br>Gradient: 67	Analyzer: FT<br>Res.: 60000<br>SR: 380 - 1400<br>AGC: Standard<br>AGC abs.: 400000<br>AcT: Auto<br>RF: 30<br>SF: --<br>DDM: CT/3sec	Analyzer: FT<br>Res./ScR: 30000/-<br>SR: Auto<br>AGC: 200%<br>AGC abs.: 100000<br>AcT: 70 ms<br>CS: +3 to +8<br>IsM: Q<br>IsW: 1.6<br>Frag.: aHCD<br>NCE: 25, 30			classic orbitrap experiment: MS1 in Orbitrap at high resolution and data dependent MS2 also in Orbitrap high resolution. Dynamic exclusion enabled (exclude after n times=1; Exclusion duration (s)= 30; mass tolerance= ± 10ppm)<br>Intensity Threshold: 50000<br>Ion transfer Tube Temp: 270 °C<br>Ion Source Voltage: 2200 V

Note: **FT**= Fourier Transform (Orbitrap); **IT**= Iontrap; **Q**= Quadrupol; **Res.**= max. Resolution at 200 m/z (Lumos) or 400 m/z (Elite) [FWHM (full width at half maximum)]; **ScR**= scan rate for measurements in the IT; **SR**= scan range [m/z]; **AGC**= automatic gain control, max number of acquired ions per measurement; **AcT**= max. Ion acquisition time [ms]; **CS**= charge states used for fragmentation; **IsM**= Isolation mode (Q or IT), MS2 isolation and further is only done in IT; **IsW**= Isolation window [m/z], value followed by scan mode the isolation is based on (MS1, MS2 …) **Frag.**= Fragmentation method; **HCD**= Higher-energy collisional dissociation; **CID**= Collision-induced dissociation; **ETD**= Electron-transfer dissociation**; EThcD=** Electron-Transfer/Higher-Energy Collision Dissociation; **sHCD**= stepped HCD**; NCE**= normalized collision energy; **cycles**: number of MSn recorded or max cycle time; RF= RF Lens [%]; **SF**= Source Fragmentation [V]; **DDM**: Data dependent Mode (cycle time in seconds, CT/[s] or number of scans, NS); **NS**= Number of data dependent scans

Search Settings

Program & version	MetaMorpheus v1.0.3.
Search engine	MetaMorpheusXL
settings	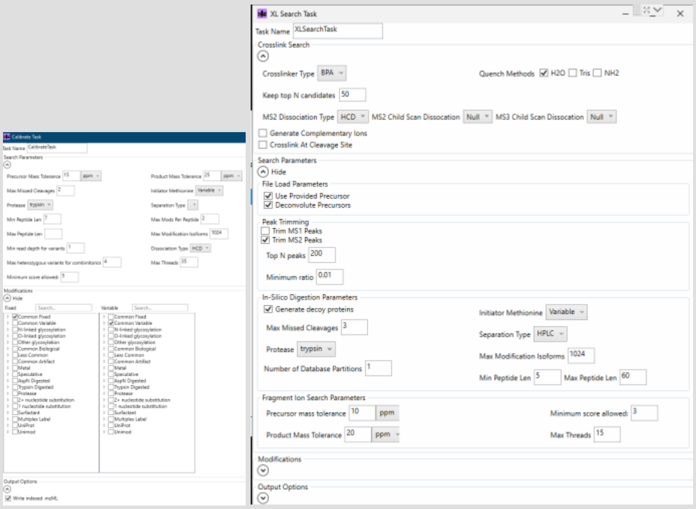
Static modification	Carbamidomethyl (C)
Digestion mode	Trypsin/P (specific), 3 missed cleavages
CL and specificity	DSS; Site A: **x**; Site B: **ABCDEFGHIKLMPQRSTVWY**
Dynamic modification	Oxidation (M)
Custom amino acid defined	BPA x 251.09462859 C16H13NO2
Program & version	1. ACE_0880_SOI_plus_con_MM_v01.fasta
Annotation	

Note: BPA setup as new aminoacid with code “x” (case sensitive).
