# Supplementary material for: Reverse engineering of BNIP3 identifies a mitochondrial protective peptide
Source: Nat Commun. 2026 Jun 17;17:5359. doi: 10.1038/s41467-026-73993-2 (PMC13275919; doi:10.1038/s41467-026-73993-2)
Supplement: Supplementary file 4 — Supplementary Data 2 [file 41467_2026_73993_MOESM4_ESM.pdf]

Supplementary Data 2. Serum chemistry in rats – Dosing phase

Sex: Male Day 15 relative to Start Date

Group 1, 0 mg/kg/day	ALT (U/L)	AST (U/L)	TP (g/L)	ALB (g/L)	BIL-T (µmol/L)	ALP (U/L)	GGT (U/L)	sGLU (mmol/L)	UREA (mmol/L)	CRE (µmol/L)	Ca (mmol/L)	P (mmol/L)	TCHO (mmol/L)	TG (mmol/L)	K (mmol/L)	Na (mmol/L)	Cl (mmol/L)	GLB (g/L)	A/G	CK (U/L)
1001	35	168	61.4	35.7	1.32	129	-1 E[a]	7.31	6.88	25	2.41	2.11	1.44	1.03	4.7	142	102	25.7	1.39	1014
1002	35	145	57.2	33.8	1.12	181	-1 E[a]	7.36	6.89	27	2.32	2.05	1.64	0.63	4.2	142	103	23.4	1.44	965
1003	22	131	59.9	34.3	1.30	132	-1 E[a]	7.27	8.58	23	2.47	2.34	1.70	0.45	5.1	141	101	25.6	1.34	938
1004	28	97	55.3	31.8	1.00	110	-1 E[a]	9.26	6.93	25	2.44	2.29	2.09	1.13	4.3	142	101	23.5	1.35	515
1005	26	120	54.7	32.4	1.38	101	-1 E[a]	10.31	8.20	24	2.41	1.66	1.29	0.76	4.7	142	104	22.3	1.45	815
1006	30	140	59.4	34.6	1.34	116	-1 E[a]	7.55	6.99	25	2.38	2.03	1.33	0.49	4.5	144	104	24.8	1.40	887
1007	25	105	59.0	33.6	1.76	171	-1 E[a]	9.42	9.99	28	2.45	2.45	1.46	0.37	4.8	142	104	25.4	1.32	735
1008	34	106	52.9	31.1	1.14	109	-1 E[a]	9.73	6.70	22	2.38	2.81	1.33	0.31	5.0	143	107	21.8	1.43	480
1009	23	136	61.4	35.8	1.12	139	-1 E[a]	8.86	7.24	19	2.53	2.34	1.83	0.78	4.9	144	104	25.6	1.40	1098
1010	30	74	58.5	33.4	1.39	110	-1 E[a]	9.29	8.96	21	2.39	2.41	1.87	0.54	4.7	141	105	25.1	1.33	364

Group 2, 3 mg/kg/day	ALT (U/L)	AST (U/L)	TP (g/L)	ALB (g/L)	BIL-T (µmol/L)	ALP (U/L)	GGT (U/L)	sGLU (mmol/L)	UREA (mmol/L)	CRE (µmol/L)	Ca (mmol/L)	P (mmol/L)	TCHO (mmol/L)	TG (mmol/L)	K (mmol/L)	Na (mmol/L)	Cl (mmol/L)	GLB (g/L)	A/G	CK (U/L)
2001	26	138	58.2	33.6	1.08	94	-1 E[a]	8.05	7.03	29	2.34	2.10	1.75	0.50	4.5	141	102	24.6	1.37	1002
2002	23	114	57.4	33.5	1.36	105	-1 E[a]	7.34	7.55	26	2.41	2.00	1.87	0.45	4.7	143	104	23.9	1.40	642
2003	24	133	58.5	34.5	1.17	126	0 E[a]	8.31	8.09	24	2.40	2.17	1.70	0.74	5.0	141	102	24.0	1.44	811
2004	25	111	59.6	34.9	1.17	102	0 E[a]	8.55	9.23	27	2.38	1.73	1.82	0.97	4.7	141	103	24.7	1.41	517
2005	25	124	58.0	32.9	1.69	151	-1 E[a]	11.76	8.04	21	2.38	2.28	1.84	0.77	4.7	142	105	25.1	1.31	1115
2006	27	90	58.5	33.2	1.24	164	-1 E[a]	7.91	6.72	22	2.46	2.30	1.87	0.62	4.6	143	105	25.3	1.31	470
2007	29	116	56.7	33.2	1.68	122	-1 E[a]	7.86	10.43	28	2.48	2.34	1.58	0.28	4.6	142	107	23.5	1.41	710
2008	29	105	57.7	33.6	1.34	120	-1 E[a]	10.69	7.21	20	2.49	2.59	1.53	0.35	4.8	143	106	24.1	1.39	538
2009	21	122	54.8	33.3	1.48	155	-1 E[a]	9.18	9.16	26	2.34	2.36	1.73	0.20	5.1	143	106	21.5	1.55	771
2010	32	157	56.0	33.2	1.76	164	-1 E[a]	8.86	6.75	20	2.41	2.41	1.24	0.88	4.9	143	106	22.8	1.46	1037

Group 3, 6 mg/kg/day	ALT (U/L)	AST (U/L)	TP (g/L)	ALB (g/L)	BIL-T (µmol/L)	ALP (U/L)	GGT (U/L)	sGLU (mmol/L)	UREA (mmol/L)	CRE (µmol/L)	Ca (mmol/L)	P (mmol/L)	TCHO (mmol/L)	TG (mmol/L)	K (mmol/L)	Na (mmol/L)	Cl (mmol/L)	GLB (g/L)	A/G	CK (U/L)
3001	25	95	59.3	34.4	1.63	152	-1 E[a]	8.08	7.75	26	2.43	2.39	2.12	1.10	4.3	142	100	24.9	1.38	583
3002	26	102	57.4	33.0	1.48	159	-1 E[a]	7.38	7.19	27	2.33	2.10	1.73	0.42	4.9	143	104	24.4	1.35	601
3003	20	113	56.8	33.5	1.30	108	0 E[a]	8.84	7.55	24	2.41	1.99	1.57	0.67	4.9	142	104	23.3	1.44	696
3004	30	141	57.3	34.5	1.22	162	-1 E[a]	7.08	7.55	27	2.38	1.87	1.56	1.04	4.7	141	104	22.8	1.51	822
3005	32	113	59.8	34.5	1.16	141	-1 E[a]	6.78	8.85	26	2.40	1.90	2.03	0.30	4.3	142	103	25.3	1.36	464
3006	28	129	60.2	34.7	1.76	116	-1 E[a]	6.09	7.31	29	2.39	2.00	1.91	0.48	4.5	140	102	25.5	1.36	793
3007	27	151	58.5	34.2	1.59	149	-1 E[a]	10.07	8.73	23	2.39	1.99	1.65	0.33	4.8	143	106	24.3	1.41	794
3008	30	83	58.1	34.0	1.82	134	-1 E[a]	11.34	8.38	21	2.56	2.81	1.49	0.29	5.5	142	106	24.1	1.41	225
3009	29	110	57.5	33.0	1.66	93	-1 E[a]	13.17	7.61	21	2.40	2.35	1.33	0.26	5.2	142	108	24.5	1.35	628
3010	35	82	61.1	35.3	1.00	129	-1 E[a]	13.74	9.62	23	2.53	2.77	1.90	0.42	5.1	142	104	25.8	1.37	258

Group 4, 12 mg/kg/day	ALT (U/L)	AST (U/L)	TP (g/L)	ALB (g/L)	BIL-T (µmol/L)	ALP (U/L)	GGT (U/L)	sGLU (mmol/L)	UREA (mmol/L)	CRE (µmol/L)	Ca (mmol/L)	P (mmol/L)	TCHO (mmol/L)	TG (mmol/L)	K (mmol/L)	Na (mmol/L)	Cl (mmol/L)	GLB (g/L)	A/G	CK (U/L)
4001	25	152	59.1	34.3	1.29	111	-1 E[a]	7.79	8.95	29	2.38	1.93	1.74	0.68	4.8	138	100	24.8	1.38	997
4002	29	163	53.6	29.0	0.99	112	-1 E[a]	7.63	8.47	32	2.30	2.15	1.35	0.50	5.1	141	103	24.6	1.18	1110
4003	27	130	55.0	28.7	1.54	111	-1 E[a]	7.72	8.17	28	2.31	2.21	1.68	0.48	4.9	142	103	26.3	1.09	957
4004	29	106	62.1	35.2	1.17	136	-1 E[a]	7.76	8.45	24	2.43	2.08	2.23	1.27	5.0	141	103	26.9	1.31	564
4005	26	114	56.8	31.4	1.28	142	-1 E[a]	8.18	8.23	27	2.33	1.89	1.70	0.66	4.6	142	103	25.4	1.24	590
4006	27	166	57.1	31.0	1.79	109	0 E[a]	7.83	10.08	30	2.38	2.01	1.70	0.33	5.3	144	109	26.1	1.19	1182
4007	31	108	58.9	32.1	1.17	113	-1 E[a]	8.23	10.58	28	2.46	2.21	1.51	0.50	4.9	142	107	26.8	1.20	745
4008	29	114	55.6	30.6	1.40	140	-2 E[a]	10.24	8.62	24	2.45	2.94	1.57	0.24	5.5	143	108	25.0	1.22	633
4009	32	118	56.8	33.6	1.92	178	-1 E[a]	10.01	5.86	21	2.50	3.07	1.61	0.51	5.1	143	105	23.2	1.45	600
4010	28	119	57.1	32.2	1.45	111	-1 E[a]	11.46	8.02	26	2.34	2.17	1.78	0.53	4.9	142	103	24.9	1.29	964

E = Exclude

[a] *[RC:Below the Limit of Quantification]*

Supplementary Data 2. Serum chemistry in rats – Dosing phase (continued)

Sex: Female Day 15 relative to Start Date

Group 1, 0 mg/kg/day	ALT (U/L)	AST (U/L)	TP (g/L)	ALB (g/L)	BIL-T (μmol/L)	ALP (U/L)	GGT (U/L)	sGLU (mmol/L)	UREA (mmol/L)	CRE (μmol/L)	Ca (mmol/L)	P (mmol/L)	TCHO (mmol/L)	TG (mmol/L)	K (mmol/L)	Na (mmol/L)	Cl (mmol/L)	GLB (g/L)	A/G	CK (U/L)
1501	23	124	58.8	36.5	1.85	56	-1 E[a]	7.67	9.92	29	2.43	1.53	1.51	0.39	4.5	140	104	22.3	1.64	766
1502	23	119	55.2	33.5	1.23	73	-1 E[a]	7.26	11.32	29	2.35	2.21	1.23	0.26	4.8	139	102	21.7	1.54	771
1503	21	112	61.6	37.4	1.97	97	-1 E[a]	8.83	9.15	30	2.51	2.05	1.51	0.26	4.2	140	103	24.2	1.55	594
1504	21	106	61.5	37.4	1.78	63	-1 E[a]	4.83	5.84	27	2.32	2.48	1.05	0.26	4.2	141	106	24.1	1.55	646
1505	28	115	61.2	36.7	1.36	64	-1 E[a]	7.02	7.64	23	2.50	2.22	1.82	0.37	4.2	141	104	24.5	1.50	594
1506	25	111	61.2	36.4	1.89	82	-1 E[a]	8.43	6.18	23	2.54	2.87	1.72	0.23	4.5	143	108	24.8	1.47	888
1507	36	119	61.3	37.8	2.01	61	-1 E[a]	8.25	8.03	26	2.50	2.16	1.14	0.20	4.4	142	109	23.5	1.61	570
1508	31	99	59.6	35.8	1.72	117	-1 E[a]	9.00	7.56	27	2.48	2.25	1.41	0.29	4.5	142	108	23.8	1.50	509
1509	31	149	58.6	36.1	1.51	37	-1 E[a]	6.34	6.46	26	2.47	2.36	1.08	0.36	4.5	141	104	22.5	1.60	784
1510	25	110	58.1	35.8	1.25	78	-1 E[a]	9.43	9.27	25	2.53	2.47	1.27	0.27	4.4	141	105	22.3	1.61	768

Group 2, 3 mg/kg/day	ALT (U/L)	AST (U/L)	TP (g/L)	ALB (g/L)	BIL-T (μmol/L)	ALP (U/L)	GGT (U/L)	sGLU (mmol/L)	UREA (mmol/L)	CRE (μmol/L)	Ca (mmol/L)	P (mmol/L)	TCHO (mmol/L)	TG (mmol/L)	K (mmol/L)	Na (mmol/L)	Cl (mmol/L)	GLB (g/L)	A/G	CK (U/L)
2501	23	115	58.5	36.0	1.23	75	-1 E[a]	9.22	8.42	29	2.34	2.03	1.10	0.35	4.4	140	103	22.5	1.60	833
2502	26	120	57.5	35.3	1.23	58	-1 E[a]	7.47	9.09	30	2.33	1.99	1.28	0.32	4.5	140	103	22.2	1.59	698
2503	25	118	63.3	39.1	1.60	58	-1 E[a]	7.39	8.68	29	2.44	1.47	1.35	0.31	4.1	140	105	24.2	1.62	685
2504	50	201	58.5	36.0	2.10	64	-1 E[a]	6.93	8.22	27	2.44	2.04	1.21	0.30	4.7	140	105	22.5	1.60	790
2505	22	105	60.8	37.9	1.52	73	-1 E[a]	8.88	9.26	27	2.42	2.27	1.57	0.26	3.8	141	106	22.9	1.66	594
2506	22	129	57.5	35.2	1.47	96	-1 E[a]	6.91	7.68	24	2.42	2.02	1.13	0.40	4.7	141	106	22.3	1.58	722
2507	17	80	63.0	37.6	1.90	76	-1 E[a]	8.13	6.18	25	2.51	2.36	1.25	0.33	4.0	142	108	25.4	1.48	250
2508	22	104	58.5	34.7	1.52	132	-1 E[a]	7.93	6.59	23	2.57	2.51	1.36	0.32	4.6	141	106	23.8	1.46	632
2509	22	86	60.0	37.2	1.58	91	-1 E[a]	8.57	8.17	25	2.47	2.33	1.11	0.19	4.2	143	107	22.8	1.63	437
2510	20	75	57.6	35.9	1.74	95	0 E[a]	8.04	6.67	26	2.45	2.14	1.44	0.24	4.3	143	106	21.7	1.65	390

Group 3, 6 mg/kg/day	ALT (U/L)	AST (U/L)	TP (g/L)	ALB (g/L)	BIL-T (μmol/L)	ALP (U/L)	GGT (U/L)	sGLU (mmol/L)	UREA (mmol/L)	CRE (μmol/L)	Ca (mmol/L)	P (mmol/L)	TCHO (mmol/L)	TG (mmol/L)	K (mmol/L)	Na (mmol/L)	Cl (mmol/L)	GLB (g/L)	A/G	CK (U/L)
3501	16	111	63.0	37.8	1.51	52	0 E[b]	5.27	6.73	30	2.42	2.11	1.89	0.48	3.9	140	103	25.2	1.50	659
3502	22	98	64.2	39.8	1.90	94	-1 E[b]	7.15	9.87	30	2.46	1.99	1.42	0.26	4.1	141	103	24.4	1.63	550
3503	20	106	57.3	34.3	1.63	65	-1 E[b]	7.68	8.80	24	2.40	1.99	1.31	0.35	4.4	140	103	23.0	1.49	614
3504	28	113	62.6	38.2	1.85	73	-1 E[b]	7.04	9.19	27	2.46	1.90	1.10	0.28	4.2	142	106	24.4	1.57	619
3505	28 [a]	132 [a]	59.4 [a]	35.8 [a]	1.42 [a]	91 [a]	-3 E[c]	8.05 [a]	6.74 [a]	19 [a]	2.73 [a]	2.96 [a]	1.17 [a]	0.38 [a]	5.1 [a]	142 [a]	108 [a]	23.6	1.52	1618 [a]
3506	24	110	61.3	37.1	1.72	66	-1 E[b]	8.29	7.24	28	2.57	1.86	1.66	0.31	4.4	140	104	24.2	1.53	488
3507	25	71	57.9	35.1	1.28	70	0 E[b]	7.28	6.67	27	2.48	2.26	1.49	0.30	4.0	141	105	22.8	1.54	280
3508	29	113	58.0	35.4	1.84	97	-1 E[b]	8.36	8.89	31	2.44	1.75	0.84	0.24	4.7	141	105	22.6	1.57	646
3509	28	118	60.2	36.8	1.85	40	-1 E[b]	8.10	6.97	25	2.47	1.94	1.96	0.39	4.2	143	106	23.4	1.57	704
3510	24	86	59.7	36.4	1.38	64	-1 E[b]	7.06	7.31	30	2.45	2.07	1.54	0.24	4.3	141	104	23.3	1.56	427

Group 4, 12 mg/kg/day	ALT (U/L)	AST (U/L)	TP (g/L)	ALB (g/L)	BIL-T (μmol/L)	ALP (U/L)	GGT (U/L)	sGLU (mmol/L)	UREA (mmol/L)	CRE (μmol/L)	Ca (mmol/L)	P (mmol/L)	TCHO (mmol/L)	TG (mmol/L)	K (mmol/L)	Na (mmol/L)	Cl (mmol/L)	GLB (g/L)	A/G	CK (U/L)
4501	21	134	60.7	36.7	1.69	99	-1 E[b]	5.90	8.50	35	2.35	1.82	1.36	0.56	3.7	141	104	24.0	1.53	768
4502	29	87	59.4	36.2	1.58	112	0 E[b]	8.21	8.07	29	2.49	2.36	1.39	0.27	4.6	139	105	23.2	1.56	464
4503	29	111	62.7	37.9	1.78	82	-1 E[b]	6.26	7.14	28	2.49	2.07	1.35	0.29	4.2	141	103	24.8	1.53	613
4504	26	122	62.8	37.6	2.32	90	-1 E[b]	8.44	8.47	31	2.47	1.86	1.50	0.28	4.2	140	104	25.2	1.49	619
4505	22	107	58.3	35.1	1.50	75	-1 E[b]	8.90	8.95	26	2.29	1.93	1.23	0.35	4.1	140	105	23.2	1.51	674
4506	23	110	60.6	36.8	1.95	74	-1 E[b]	8.01	8.60	28	2.48	2.25	1.98	0.46	4.4	141	103	23.8	1.55	521
4507	28 [a]	175 [a]	61.6 [a]	37.0 [a]	1.92 [a]	59 [a]	-3 E[c]	6.67 [a]	5.27 [a]	25 [a]	2.47 [a]	2.37 [a]	1.58 [a]	0.39 [a]	4.9 [a]	140 [a]	104 [a]	24.6	1.50	5580 [a]
4508	28	109	61.8	36.6	1.78	74	-1 E[b]	9.18	11.96	24	2.51	2.04	1.59	0.56	4.7	143	107	25.2	1.45	610
4509	24	85	64.8	40.4	2.27	78	-1 E[b]	9.28	5.58	23	2.62	2.31	1.11	0.74	3.9	142	105	24.4	1.66	358
4510	36	147	57.6	31.5	1.88	58	-1 E[b]	7.86	5.92	25	2.43	2.51	1.08	0.23	4.9	142	103	26.1	1.21	700

E = Exclude
[a] [RC:Below the Limit of Quantification]

Abbreviations

ALT	Alanine Aminotransferase
AST	Aspartate Aminotransferase
TP	Total Protein
ALB	Albumin
BIL-T	Total Bilirubin(Diasys)
ALP	Alkaline Phosphatase
GGT	Gamma-Glutamyltransferase
sGLU	Glucose
UREA	Urea
CRE	Creatinine
Ca	Calcium
P	Inorganic Phosphorus
TCHO	Total Cholesterol
TG	Triglyceride
K	Potassium
Na	Sodium
Cl	Chloride
GLB	Globulin
A/G	A/G Ratio
CK	Creatine Kinase
