# Supplementary material for: Reverse engineering of BNIP3 identifies a mitochondrial protective peptide
Source: Nat Commun. 2026 Jun 17;17:5359. doi: 10.1038/s41467-026-73993-2 (PMC13275919; doi:10.1038/s41467-026-73993-2)
Supplement: Supplementary file 5 — Supplementary Data 3 [file 41467_2026_73993_MOESM5_ESM.pdf]

Supplementary Data 3. Urine analysis parameters in rats – Dosing phase

Sex: Male Day 15 relative to Start Date

Group 1, 0 mg/kg/day	LEU	GLU	BIL	KET	BLD	PRO	URO	Color	Clarity	pH	SG	Volume (mL)
1001	-	-	-	-	-	+-	-	ST	-	8.5	1.012	29
1002	-	-	-	-	-	+-	-	ST	-	7.0	1.017	12
1003	-	-	-	-	-	+-	-	ST	-	7.5	1.017	16
1004	-	-	-	-	-	-	-	L-YE	-	7.5	1.007	7
1005	-	-	-	-	-	-	-	ST	-	8.0	1.011	22
1006	-	-	-	-	-	+-	-	ST	-	7.0	1.014	22
1007	+	-	-	-	-	+	-	ST	+	7.5	1.029	8
1008	-	-	-	-	-	-	-	ST	-	7.5	1.011	24
1009	-	-	-	-	-	+-	-	ST	-	7.5	1.010	25
1010	-	-	-	-	-	+-	-	ST	-	7.5	1.011	30

Group 2, 3 mg/kg/day	LEU	GLU	BIL	KET	BLD	PRO	URO	Color	Clarity	pH	SG	Volume (mL)
2001	-	-	-	-	-	+-	-	ST	-	7.0	1.012	25
2002	-	-	-	-	-	+-	-	ST	-	7.5	1.010	30
2003	-	-	-	-	-	+-	-	ST	-	7.0	1.015	20
2004	-	-	-	-	-	+-	-	ST	-	7.0	1.015	17
2005	-	-	-	-	-	+-	-	ST	+	8.0	1.019	13
2006	-	-	-	-	-	+-	-	ST	+	8.0	1.020	14
2007	-	-	-	-	-	+	-	ST	-	7.5	1.025	10
2008	-	-	-	-	-	-	-	L-YE	-	7.0	1.009	32
2009	-	-	-	-	-	-	-	L-YE	-	7.5	1.008	29
2010	-	-	-	-	-	+-	-	L-YE	-	7.5	1.011	26

Group 3, 6 mg/kg/day	LEU	GLU	BIL	KET	BLD	PRO	URO	Color	Clarity	pH	SG	Volume (mL)
3001	-	-	-	-	-	+-	-	ST	-	7.5	1.013	27
3002	-	-	-	-	-	-	-	ST	-	7.5	1.014	22
3003	-	-	-	-	-	-	-	L-YE	-	7.0	1.010	34
3004	-	-	-	-	-	+	-	ST	-	7.5	1.022	12
3005	+	-	-	-	-	+	-	ST	-	7.0	1.035	8
3006	-	-	-	-	-	+-	-	ST	+	7.5	1.020	16
3007	-	-	-	-	-	+-	-	ST	-	7.5	1.021	12
3008	-	-	-	-	-	+-	-	ST	-	8.5	1.013	25
3009	-	-	-	-	-	+-	-	ST	-	7.0	1.013	22
3010	-	-	-	-	-	+-	-	ST	-	7.5	1.012	27

Group 4, 12 mg/kg/day	LEU	GLU	BIL	KET	BLD	PRO	URO	Color	Clarity	pH	SG	Volume (mL)
4001	-	-	-	-	-	+-	-	ST	-	7.0	1.017	17
4002	-	-	-	-	+-	-	-	L-YE	-	7.0	1.008	47
4003	-	-	-	-	+	+	-	ST	-	7.5	1.027	9
4004	-	-	-	-	-	+-	-	ST	-	7.0	1.015	24
4005	-	-	-	-	-	+-	-	ST	-	7.5	1.015	22
4006	-	-	-	-	+	-	-	L-YE	-	6.5	1.010	29
4007	-	-	-	-	+-	+	-	ST	-	7.0	1.026	11
4008	-	-	-	-	+-	+-	-	ST	-	7.5	1.011	29
4009	-	-	-	-	-	+-	-	ST	-	8.0	1.012	25
4010	-	-	-	-	+	+	-	ST	-	6.5	1.026	11

Supplementary Data 3. Urine analysis parameters in rats – Dosing phase (continued)

Sex: Female Day 15 relative to Start Date

Group 1, 0 mg/kg/day	LEU	GLU	BIL	KET	BLD	PRO	URO	Color	Clarity	pH	SG	Volume (mL)
1501	-	-	-	-	-	-	-	ST	-	6.0	1.016	14
1502	-	-	-	-	-	-	-	ST	-	6.5	1.014	15
1503	-	-	-	-	-	-	-	ST	-	7.5	1.017	10
1504	-	-	-	-	-	+-	-	ST	-	6.0	1.032	4
1505	-	-	-	-	-	-	-	ST	-	5.0	1.020	6
1506	-	-	-	-	-	+-	-	ST	-	5.5	1.029	2
1507	-	-	-	-	-	-	-	ST	-	6.0	1.015	12
1508	-	-	-	-	-	-	-	ST	-	6.0	1.025	9
1509	-	-	-	-	+-	+-	-	ST	-	6.5	1.038	3
1510	-	-	-	-	-	-	-	ST	-	5.0	1.033	6
Group 2, 3 mg/kg/day	LEU	GLU	BIL	KET	BLD	PRO	URO	Color	Clarity	pH	SG	Volume (mL)
2501	-	-	-	-	-	-	-	L-YE	-	6.5	1.010	24
2502	-	-	-	-	-	-	-	ST	-	6.5	1.014	12
2503	-	-	-	-	-	-	-	ST	-	6.0	1.022	8
2504	-	-	-	-	-	+	-	AM	-	5.5	1.049	3
2505	-	-	-	-	-	-	-	ST	-	6.5	1.019	9
2506	-	-	-	-	-	-	-	ST	-	6.0	1.025	10
2507	-	-	-	-	-	-	-	ST	-	7.0	1.015	12
2508	-	-	-	-	-	-	-	ST	-	6.0	1.017	12
2509	-	-	-	-	-	-	-	ST	-	6.5	1.012	17
2510	-	-	-	-	-	-	-	L-YE	-	6.5	1.012	14
Group 3, 6 mg/kg/day	LEU	GLU	BIL	KET	BLD	PRO	URO	Color	Clarity	pH	SG	Volume (mL)
3501	-	-	-	-	-	-	-	L-YE	-	7.0	1.012	17
3502	-	-	-	-	-	-	-	L-YE	-	7.0	1.009	24
3503	-	-	-	-	-	-	-	L-YE	-	6.5	1.008	35
3504	-	-	-	-	-	-	-	L-YE	-	5.5	1.010	22
3505	NR [a]	NR [a]	NR [a]	NR [a]	NR [a]	NR [a]	NR [a]	NR [a]	NR [a]	NR [a]	NR [a]	1 [b]
3506	-	-	-	-	-	-	-	ST	-	6.0	1.020	9
3507	-	-	-	-	-	-	-	ST	-	6.0	1.015	15
3508	-	-	-	-	-	-	-	ST	-	6.0	1.018	10
3509	-	-	-	-	-	-	-	L-YE	-	6.5	1.014	17
3510	-	-	-	-	-	-	-	ST	-	6.5	1.014	11
Group 4, 12 mg/kg/day	LEU	GLU	BIL	KET	BLD	PRO	URO	Color	Clarity	pH	SG	Volume (mL)
4501	-	-	-	-	-	-	-	ST	-	6.5	1.020	9
4502	-	-	-	-	-	-	-	ST	-	6.5	1.022	10
4503	-	-	-	-	-	-	-	ST	-	6.5	1.014	17
4504	-	-	-	-	-	-	-	L-YE	-	5.5	1.007	31
4505	-	-	-	-	-	-	-	ST	-	6.0	1.014	17
4506	-	-	-	-	-	-	-	ST	-	5.0	1.027	5
4507	-	-	-	-	-	-	-	ST	-	6.5	1.019	9
4508	-	-	-	-	-	-	-	ST	-	5.5	1.020	10
4509	-	-	-	-	-	-	-	L-YE	-	6.5	1.011	22
4510	-	-	-	-	+	-	-	ST	-	7.0	1.017	10

Abbreviations

LEU Urine Leucocyte (Semi-quantitative)
GLU Urine Glucose (Semi-quantitative)
BIL Urine Bilirubin (Semi-quantitative)
KET Urine Ketones (Semi-quantitative)
BLD Urine Occult Blood (Semi-quantitative)
PRO Urine Protein (Semi-quantitative)
URO Urine Urobilinogen (Semi-quantitative)
Color Urine Color
Clarity Clarity
pH Urine pH
SG Urine Specific Gravity
Volume Volume
