# Supplementary material for: Reverse engineering of BNIP3 identifies a mitochondrial protective peptide
Source: Nat Commun. 2026 Jun 17;17:5359. doi: 10.1038/s41467-026-73993-2 (PMC13275919; doi:10.1038/s41467-026-73993-2)
Supplement: Supplementary file 6 — Supplementary Data 4 [file 41467_2026_73993_MOESM6_ESM.pdf]

**Supplementary Data 4**. Histopathology Findings in rats – Dosing Phase

| Removal Reason(s): Scheduled Euthanasia-Termination(Dosing Phase)<br>Summary: Incidence | Male | | | | Female | | | |
|---|---|---|---|---|---|---|---|---|
| | Group 1, 0 mg/kg/day | Group 2, 3 mg/kg/day | Group 3, 6 mg/kg/day | Group 4, 12 mg/kg/day | Group 1, 0 mg/kg/day | Group 2, 3 mg/kg/day | Group 3, 6 mg/kg/day | Group 4, 12 mg/kg/day |
| Number of Animals: | 10 | 10 | 10 | 10 | 10 | 10 | 10 | 10 |
| Number of Completed Animals: | 10 | 10 | 10 | 10 | 10 | 10 | 10 | 10 |
| **Adrenal Glands** | | | | | | | | |
| Examined | 10 | 0 | 0 | 10 | 10 | 0 | 0 | 10 |
| No Visible Lesions | 10 | . | . | 10 | 10 | . | . | 10 |
| **Aorta** | | | | | | | | |
| Examined | 10 | 0 | 0 | 10 | 10 | 0 | 0 | 10 |
| No Visible Lesions | 10 | . | . | 10 | 10 | . | . | 10 |
| **Bone Marrow, Sternum** | | | | | | | | |
| Examined | 10 | 0 | 0 | 10 | 10 | 0 | 0 | 10 |
| No Visible Lesions | 10 | . | . | 10 | 10 | . | . | 10 |
| **Bone, Sternum** | | | | | | | | |
| Examined | 10 | 0 | 0 | 10 | 10 | 0 | 0 | 10 |
| No Visible Lesions | 10 | . | . | 10 | 10 | . | . | 10 |
| **Bone, Femur, Including Stifle Joint** | | | | | | | | |
| Examined | 10 | 0 | 0 | 10 | 10 | 0 | 0 | 10 |
| No Visible Lesions | 10 | . | . | 10 | 10 | . | . | 10 |
| **Brain** | | | | | | | | |
| Examined | 10 | 0 | 0 | 10 | 10 | 0 | 0 | 10 |
| No Visible Lesions | 10 | . | . | 10 | 10 | . | . | 10 |
| **Epididymides** | | | | | | | | |
| Examined | 10 | 0 | 0 | 10 | . | . | . | . |
| No Visible Lesions | 10 | . | . | 9 | . | . | . | . |
| Infiltrate; Mononuclear Cell, Interstitium; Multifocal | 0 | . | . | 1 | . | . | . | . |
| .... Minimal | 0 | . | . | 1 | . | . | . | . |
| **Esophagus** | | | | | | | | |
| Examined | 10 | 0 | 0 | 10 | 10 | 0 | 0 | 10 |
| No Visible Lesions | 10 | . | . | 10 | 10 | . | . | 10 |
| **Eyes** | | | | | | | | |
| Examined | 10 | 0 | 0 | 10 | 10 | 0 | 0 | 10 |
| No Visible Lesions | 10 | . | . | 10 | 10 | . | . | 10 |
| **Nerve(S), Optic** | | | | | | | | |
| Examined | 10 | 0 | 0 | 10 | 10 | 0 | 0 | 10 |
| No Visible Lesions | 10 | . | . | 10 | 10 | . | . | 10 |
| **Fallopian Tubes** | | | | | | | | |
| Examined | . | . | . | . | 10 | 0 | 0 | 10 |
| No Visible Lesions | . | . | . | . | 10 | . | . | 10 |
| **Harderian Glands** | | | | | | | | |
| Examined | 10 | 0 | 0 | 10 | 10 | 0 | 0 | 10 |
| No Visible Lesions | 10 | . | . | 10 | 9 | . | . | 10 |
| Infiltration; Mononuclear Cell, Multifocal, Unilateral | 0 | . | . | 0 | 1 | . | . | 0 |
| .... Mild | 0 | . | . | 0 | 1 | . | . | 0 |
| **Heart** | | | | | | | | |
| Examined | 10 | 0 | 0 | 10 | 10 | 0 | 0 | 10 |
| No Visible Lesions | 9 | . | . | 10 | 10 | . | . | 10 |
| Infiltration; Mononuclear Cell, Myocardium; Focal | 1 | . | . | 0 | 0 | . | . | 0 |
| .... Minimal | 1 | . | . | 0 | 0 | . | . | 0 |
| **Kidneys** | | | | | | | | |
| Examined | 10 | 0 | 0 | 10 | 10 | 0 | 0 | 10 |

**Supplementary Data 4**. Histopathology Findings in rats – Dosing Phase (continued)

| Removal Reason(s): Scheduled Euthanasia-Termination(Dosing Phase)<br>Summary: Incidence | Male | | | | Female | | | |
|---|---|---|---|---|---|---|---|---|
| | Group 1, 0 mg/kg/day | Group 2, 3 mg/kg/day | Group 3, 6 mg/kg/day | Group 4, 12 mg/kg/day | Group 1, 0 mg/kg/day | Group 2, 3 mg/kg/day | Group 3, 6 mg/kg/day | Group 4, 12 mg/kg/day |
| Number of Animals: | 10 | 10 | 10 | 10 | 10 | 10 | 10 | 10 |
| Number of Completed Animals: | 10 | 10 | 10 | 10 | 10 | 10 | 10 | 10 |
| **Kidneys (Continued...)** | | | | | | | | |
| No Visible Lesions | 7 | . | . | 5 | 7 | . | . | 6 |
| Nephroblastematosis; Benign, Cortex; Incidental | 0 | . | . | 0 | 0 | . | . | 1 |
| Infarct; Chronic, Cortex; Medulla; Focal, Unilateral | 1 | . | . | 0 | 0 | . | . | 0 |
| .... Minimal | 1 | . | . | 0 | 0 | . | . | 0 |
| Mineralization; Medulla; Multifocal, Bilateral | 0 | . | . | 0 | 1 | . | . | 0 |
| .... Minimal | 0 | . | . | 0 | 1 | . | . | 0 |
| Mineralization; Medulla; Focal | 0 | . | . | 3 | 0 | . | . | 1 |
| .... Minimal | 0 | . | . | 3 | 0 | . | . | 1 |
| Basophilia; Tubule; Focal, Unilateral | 1 | . | . | 0 | 0 | . | . | 0 |
| .... Minimal | 1 | . | . | 0 | 0 | . | . | 0 |
| Basophilia; Tubule; Multifocal, Unilateral | 1 | . | . | 0 | 0 | . | . | 0 |
| .... Minimal | 1 | . | . | 0 | 0 | . | . | 0 |
| Cyst; Medulla; Focal, Unilateral | 0 | . | . | 0 | 1 | . | . | 1 |
| .... Present | 0 | . | . | 0 | 1 | . | . | 1 |
| Infiltration; Mononuclear Cell, Interstitium; Focal, Bilateral | 1 | . | . | 0 | 0 | . | . | 0 |
| .... Minimal | 1 | . | . | 0 | 0 | . | . | 0 |
| Infiltration; Mononuclear Cell, Interstitium; Focal, Unilateral | 1 | . | . | 3 | 2 | . | . | 2 |
| .... Minimal | 1 | . | . | 3 | 2 | . | . | 2 |
| Infiltration; Mononuclear Cell, Interstitium; Multifocal, Unilateral | 0 | . | . | 1 | 0 | . | . | 1 |
| .... Minimal | 0 | . | . | 1 | 0 | . | . | 1 |
| **Large Intestine, Cecum** | | | | | | | | |
| Examined | 10 | 0 | 0 | 10 | 10 | 0 | 0 | 10 |
| No Visible Lesions | 10 | . | . | 10 | 10 | . | . | 10 |
| **Large Intestine, Colon** | | | | | | | | |
| Examined | 10 | 0 | 0 | 10 | 10 | 0 | 0 | 10 |
| No Visible Lesions | 10 | . | . | 10 | 10 | . | . | 10 |
| **Large Intestine, Rectum** | | | | | | | | |
| Examined | 10 | 0 | 0 | 10 | 10 | 0 | 0 | 10 |
| No Visible Lesions | 10 | . | . | 10 | 10 | . | . | 10 |
| **Liver** | | | | | | | | |
| Examined | 10 | 0 | 0 | 10 | 10 | 0 | 0 | 10 |
| No Visible Lesions | 9 | . | . | 8 | 9 | . | . | 9 |
| Infiltration; Inflammatory Cell, Portal; Parenchyma; Multifocal | 0 | . | . | 1 | 0 | . | . | 0 |
| .... Minimal | 0 | . | . | 1 | 0 | . | . | 0 |
| Infiltration; Mononuclear Cell, Portal; Parenchyma; Multifocal | 0 | . | . | 0 | 0 | . | . | 1 |
| .... Minimal | 0 | . | . | 0 | 0 | . | . | 1 |
| Infiltration; Mononuclear Cell, Portal; Perivascular; Multifocal | 1 | . | . | 1 | 1 | . | . | 0 |
| .... Minimal | 1 | . | . | 1 | 1 | . | . | 0 |

**Supplementary Data 4**. Histopathology Findings in rats – Dosing Phase (continued)

| Removal Reason(s): Scheduled Euthanasia-Termination(Dosing Phase)<br>Summary: Incidence | Male | | | | Female | | | |
|---|---|---|---|---|---|---|---|---|
| | Group 1, 0 mg/kg/day | Group 2, 3 mg/kg/day | Group 3, 6 mg/kg/day | Group 4, 12 mg/kg/day | Group 1, 0 mg/kg/day | Group 2, 3 mg/kg/day | Group 3, 6 mg/kg/day | Group 4, 12 mg/kg/day |
| Number of Animals: | 10 | 10 | 10 | 10 | 10 | 10 | 10 | 10 |
| Number of Completed Animals: | 10 | 10 | 10 | 10 | 10 | 10 | 10 | 10 |
| **Liver (Continued...)** | | | | | | | | |
| Necrosis/Infiltration; Hepatocyte; Subcapsular; Multifocal | 0 | . | . | 1 | 0 | . | . | 0 |
| .... Minimal | 0 | . | . | 1 | 0 | . | . | 0 |
| **Lungs With Mainstem Bronchi** | | | | | | | | |
| Examined | 10 | 0 | 0 | 10 | 10 | 0 | 0 | 10 |
| No Visible Lesions | 9 | . | . | 9 | 7 | . | . | 5 |
| Increased; Alveolus; Macrophage; Focal | 0 | . | . | 1 | 0 | . | . | 0 |
| .... Minimal | 0 | . | . | 1 | 0 | . | . | 0 |
| Increased; Alveolus; Macrophage; Multifocal | 1 | . | . | 0 | 0 | . | . | 0 |
| .... Minimal | 1 | . | . | 0 | 0 | . | . | 0 |
| Hemorrhage; Alveolus; Subcapsular; Focal | 0 | . | . | 1 | 0 | . | . | 0 |
| .... Minimal | 0 | . | . | 1 | 0 | . | . | 0 |
| Mineralization; Vascular Wall; Focal | 0 | . | . | 1 | 0 | . | . | 0 |
| .... Minimal | 0 | . | . | 1 | 0 | . | . | 0 |
| Inflammation; Mixed Cell, Bronchiolo-Alveolar; Focal | 0 | . | . | 0 | 2 | . | . | 1 |
| .... Minimal | 0 | . | . | 0 | 1 | . | . | 1 |
| .... Mild | 0 | . | . | 0 | 1 | . | . | 0 |
| Thrombus; Intravenous; Focal | 0 | . | . | 0 | 1 | . | . | 0 |
| .... Minimal | 0 | . | . | 0 | 1 | . | . | 0 |
| Metaplasia; Osseous, Alveolus; Focal | 0 | . | . | 0 | 1 | . | . | 1 |
| .... Present | 0 | . | . | 0 | 1 | . | . | 1 |
| **Lungs With Mainstem Bronchi (Continued...)** | | | | | | | | |
| Degeneration/Regeneration; Bronchiole; Epithelium; Focal | 0 | . | . | 0 | 0 | . | . | 1 |
| .... Minimal | 0 | . | . | 0 | 0 | . | . | 1 |
| Granuloma; Alveolus; Focal | 0 | . | . | 0 | 0 | . | . | 1 |
| .... Minimal | 0 | . | . | 0 | 0 | . | . | 1 |
| Granuloma; Alveolus; Multifocal | 0 | . | . | 0 | 0 | . | . | 1 |
| .... Minimal | 0 | . | . | 0 | 0 | . | . | 1 |
| **Lymph Node, Mandibular** | | | | | | | | |
| Examined | 10 | 0 | 0 | 10 | 10 | 0 | 0 | 10 |
| No Visible Lesions | 10 | . | . | 10 | 8 | . | . | 10 |
| Erythrocyte; Sinusoid; Multifocal | 0 | . | . | 0 | 2 | . | . | 0 |
| .... Minimal | 0 | . | . | 0 | 2 | . | . | 0 |
| **Lymph Node, Mesenteric** | | | | | | | | |
| Examined | 10 | 0 | 0 | 10 | 10 | 0 | 0 | 10 |
| No Visible Lesions | 10 | . | . | 9 | 10 | . | . | 10 |
| Erythrophagocytosis; Sinusoid; Multifocal | 0 | . | . | 1 | 0 | . | . | 0 |
| .... Minimal | 0 | . | . | 1 | 0 | . | . | 0 |
| **Mammary Gland, Inguinal** | | | | | | | | |
| Examined | 10 | 0 | 0 | 10 | 10 | 0 | 0 | 10 |
| No Visible Lesions | 10 | . | . | 10 | 10 | . | . | 10 |
| **Nerves, Sciatic** | | | | | | | | |
| Examined | 10 | 0 | 0 | 10 | 10 | 0 | 0 | 10 |
| No Visible Lesions | 10 | . | . | 10 | 10 | . | . | 10 |

**Supplementary Data 4**. Histopathology Findings in rats – Dosing Phase (continued)

| Removal Reason(s): Scheduled Euthanasia-Termination(Dosing Phase) Summary: Incidence | Male | | | | Female | | | |
|---|---|---|---|---|---|---|---|---|
| | Group 1, 0 mg/kg/day | Group 2, 3 mg/kg/day | Group 3, 6 mg/kg/day | Group 4, 12 mg/kg/day | Group 1, 0 mg/kg/day | Group 2, 3 mg/kg/day | Group 3, 6 mg/kg/day | Group 4, 12 mg/kg/day |
| Number of Animals: | 10 | 10 | 10 | 10 | 10 | 10 | 10 | 10 |
| Number of Completed Animals: | 10 | 10 | 10 | 10 | 10 | 10 | 10 | 10 |
| **Ovaries** | | | | | | | | |
| Examined | . | . | . | . | 10 | 0 | 1 | 10 |
| No Visible Lesions | . | . | . | . | 10 | . | 0 | 10 |
| Cyst; Follicle; Single, Unilateral | . | . | . | . | 0 | . | 1 | 0 |
| .... Present | . | . | . | . | 0 | . | 1 | 0 |
| **Pancreas** | | | | | | | | |
| Examined | 10 | 0 | 0 | 10 | 10 | 0 | 0 | 10 |
| No Visible Lesions | 10 | . | . | 10 | 10 | . | . | 10 |
| **Pituitary Gland** | | | | | | | | |
| Examined | 10 | 0 | 0 | 10 | 10 | 0 | 0 | 10 |
| No Visible Lesions | 9 | . | . | 10 | 10 | . | . | 10 |
| Cyst; Pars Distalis; Multifocal | 1 | . | . | 0 | 0 | . | . | 0 |
| .... Present | 1 | . | . | 0 | 0 | . | . | 0 |
| **Prostate Gland** | | | | | | | | |
| Examined | 10 | 0 | 0 | 10 | . | . | . | . |
| No Visible Lesions | 8 | . | . | 10 | . | . | . | . |
| Infiltration; Inflammatory Cell, Interstitium; Multifocal | 2 | . | . | 0 | . | . | . | . |
| .... Minimal | 2 | . | . | 0 | . | . | . | . |
| **Salivary Gland, Mandibular** | | | | | | | | |
| Examined | 10 | 0 | 0 | 10 | 10 | 0 | 0 | 10 |
| No Visible Lesions | 10 | . | . | 10 | 10 | . | . | 10 |
| **Seminal Vesicles** | | | | | | | | |
| Examined | 10 | 0 | 0 | 10 | . | . | . | . |
| No Visible Lesions | 10 | . | . | 10 | . | . | . | . |
| **Skeletal Muscle, Biceps Femoris** | | | | | | | | |
| Examined | 10 | 0 | 0 | 10 | 10 | 0 | 0 | 10 |
| No Visible Lesions | 10 | . | . | 10 | 10 | . | . | 10 |
| **Skin, Inguinal** | | | | | | | | |
| Examined | 10 | 0 | 0 | 10 | 10 | 0 | 0 | 10 |
| No Visible Lesions | 10 | . | . | 10 | 10 | . | . | 10 |
| **Small Intestine, Duodenum** | | | | | | | | |
| Examined | 10 | 0 | 0 | 10 | 10 | 0 | 0 | 10 |
| No Visible Lesions | 10 | . | . | 10 | 10 | . | . | 10 |
| **Small Intestine, Ileum** | | | | | | | | |
| Examined | 10 | 0 | 0 | 10 | 10 | 0 | 0 | 10 |
| No Visible Lesions | 10 | . | . | 10 | 10 | . | . | 10 |
| **Small Intestine, Jejunum** | | | | | | | | |
| Examined | 10 | 0 | 0 | 10 | 10 | 0 | 0 | 10 |
| No Visible Lesions | 10 | . | . | 10 | 10 | . | . | 10 |
| **Spinal Cord, Cervical, Thoracic, Lumbar** | | | | | | | | |
| Examined | 10 | 0 | 0 | 10 | 10 | 0 | 0 | 10 |
| No Visible Lesions | 10 | . | . | 10 | 10 | . | . | 10 |
| **Spleen** | | | | | | | | |
| Examined | 10 | 0 | 0 | 10 | 10 | 0 | 0 | 10 |
| No Visible Lesions | 9 | . | . | 9 | 10 | . | . | 10 |
| Extramedullary Hematopoiesis; Increased, Red Pulp; Multifocal | 1 | . | . | 0 | 0 | . | . | 0 |
| .... Minimal | 1 | . | . | 0 | 0 | . | . | 0 |

**Supplementary Data 4**. Histopathology Findings in rats – Dosing Phase (continued)

| Removal Reason(s): Scheduled Euthanasia-Termination(Dosing Phase)<br>Summary: Incidence | Male | | | | Female | | | |
|---|---|---|---|---|---|---|---|---|
| | Group 1, 0 mg/kg/day | Group 2, 3 mg/kg/day | Group 3, 6 mg/kg/day | Group 4, 12 mg/kg/day | Group 1, 0 mg/kg/day | Group 2, 3 mg/kg/day | Group 3, 6 mg/kg/day | Group 4, 12 mg/kg/day |
| Number of Animals: | 10 | 10 | 10 | 10 | 10 | 10 | 10 | 10 |
| Number of Completed Animals: | 10 | 10 | 10 | 10 | 10 | 10 | 10 | 10 |
| **Spleen (Continued...)** | | | | | | | | |
| Decreased Cellularity; Lymphocytic, Marginal Zone; White Pulp; Diffuse | 0 | . | . | 1 | 0 | . | . | 0 |
| .... Minimal | 0 | . | . | 1 | 0 | . | . | 0 |
| **Stomach** | | | | | | | | |
| Examined | 10 | 0 | 0 | 10 | 10 | 0 | 0 | 10 |
| No Visible Lesions | 9 | . | . | 10 | 10 | . | . | 10 |
| Vacuolation; Cytoplasmic, Epithelium; Multifocal | 1 | . | . | 0 | 0 | . | . | 0 |
| .... Minimal | 1 | . | . | 0 | 0 | . | . | 0 |
| **Testes** | | | | | | | | |
| Examined | 10 | 0 | 0 | 10 | . | . | . | . |
| No Visible Lesions | 9 | . | . | 10 | . | . | . | . |
| Degeneration/Atrophy; Seminiferous Tubule; Multifocal, Bilateral | 1 | . | . | 0 | . | . | . | . |
| .... Mild | 1 | . | . | 0 | . | . | . | . |
| **Thymus** | | | | | | | | |
| Examined | 10 | 0 | 0 | 10 | 10 | 0 | 0 | 10 |
| No Visible Lesions | 10 | . | . | 10 | 10 | . | . | 10 |
| **Thyroid Glands** | | | | | | | | |
| Examined | 10 | 0 | 0 | 10 | 10 | 0 | 0 | 10 |
| No Visible Lesions | 10 | . | . | 10 | 9 | . | . | 10 |
| Lymphoid Tissue; Interstitium; Focal, Unilateral | 0 | . | . | 0 | 1 | . | . | 0 |
| .... Minimal | 0 | . | . | 0 | 1 | . | . | 0 |
| **[illegible]** | | | | | | | | |
| Examined | 10 | 0 | 0 | 10 | 10 | 0 | 0 | 10 |
| No Visible Lesions | 10 | . | . | 10 | 10 | . | . | 10 |
| **Trachea** | | | | | | | | |
| Examined | 10 | 0 | 0 | 10 | 10 | 0 | 0 | 10 |
| No Visible Lesions | 10 | . | . | 10 | 10 | . | . | 10 |
| **Urinary Bladder** | | | | | | | | |
| Examined | 10 | 0 | 0 | 10 | 10 | 0 | 0 | 10 |
| No Visible Lesions | 10 | . | . | 10 | 10 | . | . | 10 |
| **Uterus** | | | | | | | | |
| Examined | . | . | . | . | 10 | 0 | 0 | 10 |
| No Visible Lesions | . | . | . | . | 8 | . | . | 7 |
| Dilation; Bilateral | . | . | . | . | 1 | . | . | 3 |
| .... Mild | . | . | . | . | 1 | . | . | 3 |
| Dilation; Unilateral | . | . | . | . | 1 | . | . | 0 |
| .... Mild | . | . | . | . | 1 | . | . | 0 |
| **Cervix** | | | | | | | | |
| Examined | . | . | . | . | 10 | 0 | 0 | 10 |
| No Visible Lesions | . | . | . | . | 8 | . | . | 7 |
| Dilation | . | . | . | . | 2 | . | . | 3 |
| .... Mild | . | . | . | . | 2 | . | . | 3 |
| **Vagina** | | | | | | | | |
| Examined | . | . | . | . | 10 | 0 | 0 | 10 |
| No Visible Lesions | . | . | . | . | 10 | . | . | 9 |

**Supplementary Data 4**. Histopathology Findings in rats – Dosing Phase (continued)

| Removal Reason(s): Scheduled Euthanasia-Termination(Dosing Phase)<br>Summary: Incidence | Male | | | | Female | | | |
|---|---|---|---|---|---|---|---|---|
| | Group 1, 0 mg/kg/day | Group 2, 3 mg/kg/day | Group 3, 6 mg/kg/day | Group 4, 12 mg/kg/day | Group 1, 0 mg/kg/day | Group 2, 3 mg/kg/day | Group 3, 6 mg/kg/day | Group 4, 12 mg/kg/day |
| Number of Animals: | 10 | 10 | 10 | 10 | 10 | 10 | 10 | 10 |
| Number of Completed Animals: | 10 | 10 | 10 | 10 | 10 | 10 | 10 | 10 |
| **Vagina (Continued...)** | | | | | | | | |
| Mucification; Mucosa; Epithelium; Diffuse | . | . | . | . | 0 | . | . | 1 |
| .... Minimal | . | . | . | . | 0 | . | . | 1 |
| **Injection Site(S)** | | | | | | | | |
| Examined | 10 | 10 | 10 | 10 | 10 | 10 | 10 | 10 |
| No Visible Lesions | 10 | 6 | 5 | 0 | 6 | 6 | 5 | 3 |
| Edema; Subcutaneous, Perivascular | 0 | 0 | 0 | 1 | 0 | 0 | 0 | 0 |
| .... Minimal | 0 | 0 | 0 | 1 | 0 | 0 | 0 | 0 |
| Hemorrhage; Subcutaneous; Perivascular | 0 | 0 | 1 | 2 | 0 | 0 | 0 | 3 |
| .... Minimal | 0 | 0 | 1 | 1 | 0 | 0 | 0 | 3 |
| .... Mild | 0 | 0 | 0 | 1 | 0 | 0 | 0 | 0 |
| Inflammation; Mixed Cell, Subcutaneous; Perivascular | 0 | 4 | 5 | 5 | 4 | 4 | 5 | 6 |
| .... Minimal | 0 | 4 | 4 | 1 | 4 | 4 | 4 | 3 |
| .... Mild | 0 | 0 | 1 | 3 | 0 | 0 | 1 | 3 |
| .... Moderate | 0 | 0 | 0 | 1 | 0 | 0 | 0 | 0 |
| Inflammation; Mixed Cell And Necrotizing, Cutaneous/Subcutaneous; Cutaneous Muscle | 0 | 0 | 0 | 2 | 0 | 0 | 0 | 1 |
| .... Moderate | 0 | 0 | 0 | 1 | 0 | 0 | 0 | 1 |
| .... Marked | 0 | 0 | 0 | 1 | 0 | 0 | 0 | 0 |
| Necrosis; Cutaneous/Subcutaneous; Cutaneous Muscle | 0 | 0 | 0 | 2 | 0 | 0 | 0 | 0 |
| .... Mild | 0 | 0 | 0 | 2 | 0 | 0 | 0 | 0 |
| Necrosis; Cutaneous/Subcutaneous; Vein | 0 | 0 | 0 | 1 | 0 | 0 | 0 | 0 |
| .... Mild | 0 | 0 | 0 | 1 | 0 | 0 | 0 | 0 |
| **Injection Site(S) (Continued...)** | | | | | | | | |
| Thrombosis; Intravenous; Focal | 0 | 0 | 0 | 0 | 0 | 0 | 0 | 2 |
| .... Mild | 0 | 0 | 0 | 0 | 0 | 0 | 0 | 2 |
| Congestion; Cutaneous/Subcutaneous; Multifocal | 0 | 0 | 0 | 1 | 0 | 0 | 0 | 0 |
| .... Mild | 0 | 0 | 0 | 1 | 0 | 0 | 0 | 0 |
| Bacterial Colonies; Epidermis; Multifocal | 0 | 0 | 0 | 1 | 0 | 0 | 0 | 0 |
| .... Present | 0 | 0 | 0 | 1 | 0 | 0 | 0 | 0 |
