# Supplementary material for: Reverse engineering of BNIP3 identifies a mitochondrial protective peptide
Source: Nat Commun. 2026 Jun 17;17:5359. doi: 10.1038/s41467-026-73993-2 (PMC13275919; doi:10.1038/s41467-026-73993-2)
Supplement: Supplementary file 7 — Supplementary Data 5 [file 41467_2026_73993_MOESM7_ESM.pdf]

Supplementary Data 5. Histopathological Findings in rats – Recovery Phase

Removal Reason(s): Scheduled Euthanasia-Termination(Recovery Phase)<br>Summary: Incidence	Male		Female	
	Group 1, 0 mg/kg/day	Group 4, 12 mg/kg/day	Group 1, 0 mg/kg/day	Group 4, 12 mg/kg/day
Number of Animals:	5	5	5	5
Number of Completed Animals:	5	5	5	5
**Adrenal Glands**				
Examined	5	5	5	5
No Visible Lesions	5	5	5	5
**Aorta**				
Examined	5	5	5	5
No Visible Lesions	5	5	5	5
**Bone Marrow, Sternum**				
Examined	5	5	5	5
No Visible Lesions	5	5	5	5
**Bone, Sternum**				
Examined	5	5	5	5
No Visible Lesions	5	5	5	5
**Bone, Femur, Including Stifle Joint**				
Examined	5	5	5	5
No Visible Lesions	5	4	4	5
Increased Thickness; Growth Plate; Segmental	0	1	1	0
.... Minimal	0	1	1	0
**Brain**				
Examined	5	5	5	5
No Visible Lesions	5	5	5	5
**Epididymides**				
Examined	5	5	.	.
No Visible Lesions	5	4	.	.
Cell Debris; Duct; Diffuse, Bilateral	0	1	.	.
.... Mild	0	1	.	.
Hypospermia; Luminal, Duct; Diffuse, Bilateral	0	1	.	.
.... Moderate	0	1	.	.
**Esophagus**				
Examined	5	5	5	5
No Visible Lesions	5	5	5	5
**Eyes**				
Examined	5	5	5	5
No Visible Lesions	5	5	5	5
**Nerve(S), Optic**				
Examined	5	5	5	5
No Visible Lesions	5	5	5	5
**Fallopian Tubes**				
Examined	.	.	5	5
No Visible Lesions	.	.	5	5
**Harderian Glands**				
Examined	5	5	5	5
No Visible Lesions	5	3	5	5
Infiltration; Mononuclear Cell, Focal, Bilateral	0	1	0	0
.... Minimal	0	1	0	0
Infiltration; Mononuclear Cell, Multifocal, Unilateral	0	1	0	0
.... Minimal	0	1	0	0
**Heart**				
Examined	5	5	5	5
No Visible Lesions	5	4	5	4

Supplementary Data 5. Histopathological Findings in rats– Recovery Phase (continued)

Removal Reason(s): Scheduled Euthanasia-Termination(Recovery Phase)<br>Summary: Incidence	Male		Female	
	Group 1, 0 mg/kg/day	Group 4, 12 mg/kg/day	Group 1, 0 mg/kg/day	Group 4, 12 mg/kg/day
Number of Animals:	5	5	5	5
Number of Completed Animals:	5	5	5	5
**Heart (Continued...)**				
Infiltration; Mononuclear Cell, Myocardium; Focal	0	1	0	1
.... Minimal	0	1	0	1
**Kidneys**				
Examined	5	5	5	5
No Visible Lesions	3	3	4	5
Infarct; Chronic, Cortex; Medulla; Focal, Unilateral	0	0	1	0
.... Minimal	0	0	1	0
Inflammation; Lymphohistiocytic, Medulla; Focal, Unilateral	1	0	0	0
.... Minimal	1	0	0	0
Mineralization; Medulla; Focal	0	1	0	0
.... Minimal	0	1	0	0
Cyst; Medulla; Multifocal, Unilateral	0	1	0	0
.... Present	0	1	0	0
Dilation; Tubule; Focal, Unilateral	0	1	0	0
.... Minimal	0	1	0	0
Infiltration; Mononuclear Cell, Interstitium; Focal, Bilateral	0	1	0	0
.... Minimal	0	1	0	0
Infiltration; Mononuclear Cell, Interstitium; Focal, Unilateral	1	0	0	0
.... Minimal	1	0	0	0
**Large Intestine, Cecum**				
Examined	5	5	5	5
No Visible Lesions	5	5	5	5
**Large Intestine, Colon**				
Examined	5	5	5	5
No Visible Lesions	5	5	5	5
**Large Intestine, Rectum**				
Examined	5	5	5	5
No Visible Lesions	5	5	5	5
**Liver**				
Examined	5	5	5	5
No Visible Lesions	4	5	4	4
Infiltration; Mononuclear Cell, Portal; Perivascular; Multifocal	1	0	1	1
.... Minimal	1	0	1	1
**Lungs With Mainstem Bronchi**				
Examined	5	5	5	5
No Visible Lesions	3	5	5	4
Mineralization; Vascular Wall; Focal	0	0	0	1
.... Minimal	0	0	0	1
Metaplasia; Osseous, Alveolus; Focal	1	0	0	0
.... Present	1	0	0	0
Granuloma; Alveolus; Focal	1	0	0	0
.... Minimal	1	0	0	0
**Lymph Node, Mandibular**				
Examined	5	5	5	5
No Visible Lesions	5	5	5	5

Supplementary Data 5. Histopathological Findings in rats– Recovery Phase (continued)

Removal Reason(s): Scheduled Euthanasia-Termination(Recovery Phase)<br>Summary: Incidence	Male		Female	
	Group 1, 0 mg/kg/day	Group 4, 12 mg/kg/day	Group 1, 0 mg/kg/day	Group 4, 12 mg/kg/day
Number of Animals:	5	5	5	5
Number of Completed Animals:	5	5	5	5
**Lymph Node, Mesenteric**				
Examined	5	5	5	5
No Visible Lesions	5	5	4	5
Erythrophagocytosis; Sinusoid; Multifocal	0	0	1	0
.... Mild	0	0	1	0
**Mammary Gland, Inguinal**				
Examined	5	5	5	5
No Visible Lesions	5	5	5	5
**Nerves, Sciatic**				
Examined	5	5	5	5
No Visible Lesions	5	5	4	5
Infiltration; Inflammatory Cell, Peripheral; Multifocal	0	0	1	0
.... Minimal	0	0	1	0
**Ovaries**				
Examined	.	.	5	5
No Visible Lesions	.	.	5	5
**Pancreas**				
Examined	5	5	5	5
No Visible Lesions	5	5	5	5
**Pituitary Gland**				
Examined	5	5	5	5
No Visible Lesions	4	4	5	4
Atrophy; Pars Distalis; Diffuse	0	1	0	0
.... Minimal	0	1	0	0
**Pituitary Gland (Continued...)**				
Cyst; Pars Distalis; Focal	1	0	0	1
.... Present	1	0	0	1
**Prostate Gland**				
Examined	5	5	.	.
No Visible Lesions	1	1	.	.
Atrophy; Acinus; Diffuse	0	1	.	.
.... Minimal	0	1	.	.
Infiltration; Inflammatory Cell, Interstitium; Multifocal	4	3	.	.
.... Minimal	3	3	.	.
.... Mild	1	0	.	.
**Salivary Gland, Mandibular**				
Examined	5	5	5	5
No Visible Lesions	5	5	5	5
**Seminal Vesicles**				
Examined	5	5	.	.
No Visible Lesions	5	4	.	.
Atrophy; Acinus; Diffuse, Bilateral	0	1	.	.
.... Minimal	0	1	.	.
**Skeletal Muscle, Biceps Femoris**				
Examined	5	5	5	5
No Visible Lesions	5	5	5	5
**Skin, Inguinal**				
Examined	5	5	5	5

Supplementary Data 5. Histopathological Findings in rats– Recovery Phase (continued)

Removal Reason(s): Scheduled Euthanasia-Termination(Recovery Phase)<br>Summary: Incidence	Male		Female	
	Group 1, 0 mg/kg/day	Group 4, 12 mg/kg/day	Group 1, 0 mg/kg/day	Group 4, 12 mg/kg/day
Number of Animals:	5	5	5	5
Number of Completed Animals:	5	5	5	5
**Skin, Inguinal (Continued...)**				
No Visible Lesions	5	5	5	5
**Small Intestine, Duodenum**				
Examined	5	5	5	5
No Visible Lesions	5	5	5	5
**Small Intestine, Ileum**				
Examined	5	5	5	5
No Visible Lesions	5	5	5	5
**Small Intestine, Jejunum**				
Examined	5	5	5	5
No Visible Lesions	5	5	5	5
**Spinal Cord, Cervical, Thoracic, Lumbar**				
Examined	5	5	5	5
No Visible Lesions	5	5	5	5
**Spleen**				
Examined	5	5	5	5
No Visible Lesions	4	5	4	4
Extramedullary Hematopoiesis; Increased, Red Pulp; Multifocal	1	0	1	1
.... Minimal	1	0	0	1
.... Mild	0	0	1	0
Decreased Cellularity; Lymphocytic, Marginal Zone; White Pulp; Diffuse	0	0	1	0
.... Mild	0	0	1	0
**Stomach**				
Examined	5	5	5	5
No Visible Lesions	3	2	3	4
Infiltration; Inflammatory Cell, Submucosa; Focal	1	0	0	1
.... Minimal	1	0	0	1
Vacuolation; Epithelial, Non-Glandular; Mucosa; Multifocal	1	3	2	1
.... Minimal	1	3	2	1
Hyperkeratosis; Mucosa; Epithelium; Segmental	1	3	1	0
.... Minimal	1	3	1	0
**Testes**				
Examined	5	5	.	.
No Visible Lesions	5	4	.	.
Degeneration/Atrophy; Seminiferous Tubule; Multifocal, Bilateral	0	1	.	.
.... Mild	0	1	.	.
**Thymus**				
Examined	5	5	5	5
No Visible Lesions	5	5	5	5
**Thyroid Glands**				
Examined	5	5	5	5
No Visible Lesions	5	4	4	5
Lymphoid Tissue; Interstitium; Focal, Unilateral	0	1	0	0
.... Minimal	0	1	0	0
Ectopia-Thymus	0	0	1	0
**Parathyroid Gland(S)**				
Examined	5	5	5	5
No Visible Lesions	5	5	5	5

Supplementary Data 5. Histopathological Findings in rats– Recovery Phase (continued)

Removal Reason(s): Scheduled Euthanasia-Termination(Recovery Phase)<br>Summary: Incidence	Male		Female	
	Group 1, 0 mg/kg/day	Group 4, 12 mg/kg/day	Group 1, 0 mg/kg/day	Group 4, 12 mg/kg/day
Number of Animals:	5	5	5	5
Number of Completed Animals:	5	5	5	5
**Trachea**				
Examined	5	5	5	5
No Visible Lesions	5	5	5	5
**Urinary Bladder**				
Examined	5	5	5	5
No Visible Lesions	4	5	5	4
Infiltration; Inflammatory Cell, Serosa; Muscularis; Focal	1	0	0	0
.... Mild	1	0	0	0
Infiltration; Mononuclear Cell, Submucosa; Muscularis; Multifocal	0	0	0	1
.... Mild	0	0	0	1
**Uterus**				
Examined	.	.	5	5
No Visible Lesions	.	.	1	2
Dilation; Bilateral	.	.	4	3
.... Minimal	.	.	1	0
.... Mild	.	.	3	3
**Cervix**				
Examined	.	.	5	5
No Visible Lesions	.	.	1	2
Dilation	.	.	4	3
.... Mild	.	.	4	3
**Vagina**				
Examined	.	.	5	5
No Visible Lesions	.	.	4	5
Mucification; Mucosa; Epithelium; Diffuse	.	.	1	0
.... Minimal	.	.	1	0
**Injection Site(S)**				
Examined	5	5	5	5
No Visible Lesions	5	4	5	5
Hemorrhage; Subcutaneous; Perivascular	0	1	0	0
.... Minimal	0	1	0	0
Inflammation; Mixed Cell And Necrotizing, Cutaneous/Subcutaneous; Cutaneous Muscle; Multifocal	0	1	0	0
.... Moderate	0	1	0	0
Bacterial Colonies; Epidermis; Multifocal	0	1	0	0
.... Present	0	1	0	0
