# Supplementary material for: Reverse engineering of BNIP3 identifies a mitochondrial protective peptide
Source: Nat Commun. 2026 Jun 17;17:5359. doi: 10.1038/s41467-026-73993-2 (PMC13275919; doi:10.1038/s41467-026-73993-2)
Supplement: Supplementary file 8 — Supplementary Data 6 [file 41467_2026_73993_MOESM8_ESM.pdf]

Supplementary Data 6. Haematological values in rats – Dosing phase

Sex: Male Day 15 relative to Start Date

Group 1, 0 mg/kg/day	WBC	RBC	HGB	HCT	MCV	MCH	MCHC	RDW	#RET	%RET	#NEUT	%NEUT	#LYMP	%LYMP	#MONO	%MONO	#EOS	%EOS	#BASO	%BASO	PLT	MPV
	(10^3/µL)	(10^6/µL)	(g/dL)	(%)	(fL)	(pg)	(g/dL)	(%)	(10^9/L.)	(%)	(10^3/µL)	(%)	(10^3/µL)	(%)	(10^3/µL)	(%)	(10^3/µL)	(%)	(10^3/µL)	(%)	(10^3/µL)	(fL)
1001	3.31	7.23	14.2	40.2	55.6	19.6	35.3	12.5	216.0	2.99	0.45	13.5	2.76	83.5	0.04	1.1	0.04	1.2	0.00	0.0	939	9.6
1002	2.87	7.78	14.7	41.9	53.8	18.9	35.0	11.8	192.7	2.48	0.68	23.6	2.07	72.2	0.08	2.7	0.03	1.1	0.00	0.0	699	10.4
1003	2.67	7.80	14.6	40.6	52.0	18.7	36.0	12.4	218.7	2.80	0.34	12.9	2.25	84.2	0.04	1.6	0.02	0.9	0.00	0.1	1108	9.7
1004	4.48	7.90	14.7	41.9	53.0	18.6	35.0	11.9	214.8	2.72	0.73	16.2	3.58	80.0	0.11	2.4	0.04	1.0	0.00	0.1	917	10.6
1005	2.89	6.96	13.9	38.6	55.5	19.9	35.9	14.2	206.6	2.97	0.48	16.7	2.26	78.4	0.09	3.1	0.04	1.4	0.00	0.0	795	10.0
1006	3.79	7.45	14.7	41.5	55.7	19.7	35.3	12.0	217.8	2.92	0.69	18.1	2.92	77.1	0.08	2.2	0.09	2.3	0.00	0.1	1005	9.7
1007	5.48	7.06	14.0	39.2	55.6	19.8	35.7	15.7	270.6	3.83	0.97	17.7	4.35	79.3	0.08	1.5	0.05	0.9	0.00	0.0	879	10.6
1008	4.31	7.66	14.0	40.8	53.3	18.3	34.4	12.7	264.8	3.46	0.92	21.3	3.21	74.4	0.07	1.7	0.09	2.0	0.01	0.1	913	9.4
1009	3.03	8.10	15.3	43.9	54.2	18.9	34.8	11.9	196.7	2.43	0.34	11.2	2.54	83.8	0.11	3.5	0.03	0.8	0.01	0.3	957	9.9
1010	5.02	8.02	14.8	42.6	53.1	18.5	34.9	12.0	243.5	3.04	0.40	7.9	4.44	88.4	0.11	2.3	0.05	1.0	0.01	0.1	904	9.1

Group 2, 3 mg/kg/day	WBC	RBC	HGB	HCT	MCV	MCH	MCHC	RDW	#RET	%RET	#NEUT	%NEUT	#LYMP	%LYMP	#MONO	%MONO	#EOS	%EOS	#BASO	%BASO	PLT	MPV
	(10^3/µL)	(10^6/µL)	(g/dL)	(%)	(fL)	(pg)	(g/dL)	(%)	(10^9/L.)	(%)	(10^3/µL)	(%)	(10^3/µL)	(%)	(10^3/µL)	(%)	(10^3/µL)	(%)	(10^3/µL)	(%)	(10^3/µL)	(fL)
2001	3.45	7.19	13.4	38.4	53.5	18.6	34.8	12.6	217.1	3.02	0.36	10.4	3.01	87.3	0.06	1.7	0.02	0.6	0.00	0.0	956	9.7
2002	2.32	7.84	14.8	42.7	54.5	18.8	34.6	11.5	226.0	2.88	0.51	21.9	1.70	73.2	0.06	2.6	0.04	1.9	0.00	0.0	867	10.3
2003	3.81	7.78	14.7	41.2	52.9	18.8	35.6	12.5	250.1	3.21	0.99	25.9	2.65	69.5	0.09	2.3	0.08	2.1	0.00	0.1	1061	10.1
2004	2.97	7.39	13.9	39.3	53.2	18.9	35.4	12.2	214.2	2.90	0.44	14.9	2.38	80.0	0.09	2.9	0.06	2.0	0.00	0.0	755	10.3
2005	5.96	8.14	15.2	44.1	54.2	18.7	34.4	11.1	207.9	2.56	0.75	12.7	4.95	83.0	0.18	3.0	0.06	1.0	0.01	0.1	872	10.3
2006	2.97	8.07	14.7	42.9	53.2	18.2	34.2	12.3	245.6	3.04	0.65	21.9	2.19	73.5	0.08	2.8	0.05	1.6	0.00	0.0	830	10.2
2007	4.72	7.99	14.7	42.4	53.1	18.4	34.6	12.2	255.8	3.20	0.51	10.8	4.09	86.6	0.09	2.0	0.02	0.4	0.00	0.1	834	9.3
2008	6.51	7.61	15.0	41.9	55.1	19.7	35.8	14.0	245.4	3.22	0.80	12.3	5.43	83.4	0.12	1.8	0.12	1.9	0.00	0.1	873	9.4
2009	5.88	7.51	14.4	40.8	54.3	19.2	35.3	11.6	238.0	3.17	0.59	10.1	5.09	86.4	0.14	2.4	0.03	0.5	0.00	0.1	895	9.0
2010	7.28	7.82	15.5	43.9	56.1	19.8	35.3	11.3	177.0	2.26	1.11	15.2	5.91	81.2	0.11	1.5	0.11	1.5	0.01	0.1	888	9.7

Group 3, 6 mg/kg/day	WBC	RBC	HGB	HCT	MCV	MCH	MCHC	RDW	#RET	%RET	#NEUT	%NEUT	#LYMP	%LYMP	#MONO	%MONO	#EOS	%EOS	#BASO	%BASO	PLT	MPV
	(10^3/µL)	(10^6/µL)	(g/dL)	(%)	(fL)	(pg)	(g/dL)	(%)	(10^9/L.)	(%)	(10^3/µL)	(%)	(10^3/µL)	(%)	(10^3/µL)	(%)	(10^3/µL)	(%)	(10^3/µL)	(%)	(10^3/µL)	(fL)
3001	3.92	7.52	14.2	41.4	55.0	18.9	34.4	11.4	209.8	2.79	0.50	12.9	3.14	80.1	0.20	5.0	0.05	1.3	0.00	0.1	837	10.1
3002	4.45	7.10	13.5	38.0	53.5	19.0	35.6	13.6	243.4	3.43	0.74	16.7	3.58	80.4	0.07	1.5	0.04	1.0	0.00	0.0	941	10.2
3003	4.02	7.47	14.4	39.9	53.4	19.2	36.0	14.0	222.4	2.98	0.62	15.3	3.29	82.0	0.04	1.0	0.05	1.1	0.00	0.1	817	10.2
3004	3.17	7.51	14.6	42.6	56.7	19.4	34.2	12.0	181.5	2.42	0.48	15.2	2.58	81.3	0.08	2.4	0.03	0.9	0.00	0.1	869	9.7
3005	4.71	7.24	14.5	40.1	55.4	20.1	36.2	14.0	235.6	3.25	0.60	12.8	3.82	81.2	0.18	3.8	0.09	2.0	0.00	0.0	812	10.2
3006	4.59	7.73	13.8	39.6	51.3	17.8	34.8	13.2	235.9	3.05	0.89	19.3	3.49	76.0	0.13	2.7	0.08	1.6	0.00	0.0	903	9.8
3007	6.38	8.04	15.3	43.9	54.6	19.0	34.9	11.8	253.1	3.15	0.77	12.0	5.32	83.4	0.14	2.2	0.11	1.7	0.01	0.1	873	9.7
3008	6.07	7.65	14.7	42.8	55.9	19.2	34.4	11.6	235.2	3.07	0.73	12.0	5.05	83.1	0.20	3.2	0.06	1.0	0.00	0.1	777	9.5
3009	4.70	7.93	14.9	43.6	55.0	18.9	34.3	11.3	211.1	2.66	0.37	7.9	4.19	89.1	0.09	1.9	0.03	0.6	0.00	0.1	902	9.3
3010	4.36	8.50	15.3	44.7	52.6	18.0	34.2	12.8	269.9	3.17	0.52	11.9	3.70	84.9	0.07	1.6	0.05	1.1	0.00	0.1	819	9.9

Group 4, 12 mg/kg/day	WBC	RBC	HGB	HCT	MCV	MCH	MCHC	RDW	#RET	%RET	#NEUT	%NEUT	#LYMP	%LYMP	#MONO	%MONO	#EOS	%EOS	#BASO	%BASO	PLT	MPV
	(10^3/µL)	(10^6/µL)	(g/dL)	(%)	(fL)	(pg)	(g/dL)	(%)	(10^9/L.)	(%)	(10^3/µL)	(%)	(10^3/µL)	(%)	(10^3/µL)	(%)	(10^3/µL)	(%)	(10^3/µL)	(%)	(10^3/µL)	(fL)
4001	4.23	7.54	14.2	40.2	53.3	18.8	35.3	12.2	187.2	2.48	0.49	11.6	3.62	85.8	0.08	1.8	0.02	0.4	0.00	0.1	792	9.7
4002	5.68	7.20	13.4	38.4	53.3	18.6	34.8	11.1	182.7	2.54	1.98	34.9	3.46	60.9	0.17	3.0	0.04	0.8	0.00	0.0	1116	10.8
4003	8.80	6.91	12.9	36.7	53.1	18.7	35.2	13.0	206.1	2.98	4.13	47.0	4.32	49.1	0.27	3.1	0.05	0.6	0.00	0.1	894	10.5
4004	3.82	7.78	14.5	41.3	53.1	18.7	35.2	12.5	229.2	2.95	0.73	19.1	2.93	76.7	0.08	2.0	0.06	1.7	0.00	0.1	917	10.3
4005	5.62	7.50	13.7	39.7	53.0	18.3	34.5	12.6	301.9	4.02	2.55	45.4	2.66	47.3	0.32	5.7	0.06	1.1	0.00	0.0	927	10.8
4006	9.06	7.57	13.8	39.1	51.6	18.2	35.2	12.9	231.9	3.06	3.16	34.9	5.64	62.3	0.15	1.6	0.05	0.6	0.01	0.1	1250	9.7
4007	7.08	7.82	14.0	40.4	51.7	17.9	34.5	12.2	225.8	2.89	0.91	12.9	5.93	83.7	0.12	1.7	0.06	0.9	0.00	0.1	963	9.4
4008	9.36	7.60	14.3	42.0	55.2	18.8	34.1	11.5	267.9	3.52	2.39	25.5	6.62	70.7	0.25	2.7	0.04	0.5	0.01	0.1	993	10.2
4009	6.81	7.87	15.1	43.8	55.7	19.2	34.4	11.4	207.3	2.63	0.60	8.8	5.92	87.0	0.19	2.8	0.05	0.8	0.01	0.1	886	10.2
4010	6.80	7.62	14.2	41.2	54.0	18.7	34.6	12.0	244.0	3.20	1.58	23.2	4.96	72.9	0.19	2.8	0.06	0.8	0.00	0.0	1004	10.6

Supplementary Data 6. Haematological values in rats – Dosing phase (continued)

Sex: Female Day 15 relative to Start Date

Group 1, 0 mg/kg/day	WBC	RBC	HGB	HCT	MCV	MCH	MCHC	RDW	#RET	%RET	#NEUT	%NEUT	#LYMP	%LYMP	#MONO	%MONO	#EOS	%EOS	#BASO	%BASO	PLT	MPV
	(10^3/µL)	(10^6/µL)	(g/dL)	(%)	(fL)	(pg)	(g/dL)	(%)	(10^9/L.)	(%)	(10^3/µL)	(%)	(10^3/µL)	(%)	(10^3/µL)	(%)	(10^3/µL)	(%)	(10^3/µL)	(%)	(10^3/µL)	(fL)
1501	3.13	7.49	13.7	38.7	51.7	18.2	35.3	10.8	210.9	2.81	0.40	12.7	2.61	83.5	0.07	2.2	0.04	1.2	0.00	0.1	912	10.0
1502	2.96	7.90	14.7	41.5	52.5	18.6	35.5	11.4	184.9	2.34	0.60	20.3	2.23	75.2	0.10	3.3	0.03	0.9	0.00	0.1	882	10.1
1503	3.41	7.03	13.6	38.8	55.2	19.4	35.1	10.5	192.3	2.74	0.37	10.7	2.92	85.4	0.08	2.3	0.04	1.2	0.01	0.2	823	9.9
1504	2.96	7.71	14.0	39.7	51.5	18.1	35.2	11.4	263.3	3.42	0.68	22.8	2.16	73.0	0.07	2.5	0.04	1.3	0.00	0.0	880	10.0
1505	2.74	7.09	13.9	39.2	55.3	19.6	35.5	11.7	279.8	3.95	0.38	13.9	2.25	82.1	0.06	2.3	0.03	1.2	0.00	0.0	1165	9.9
1506	2.42	7.92	15.0	43.6	55.1	19.0	34.4	12.0	261.7	3.30	0.28	11.6	2.00	82.6	0.10	4.2	0.03	1.2	0.00	0.0	950	10.1
1507	3.29	7.83	14.3	41.4	52.9	18.3	34.5	12.0	233.7	2.98	0.41	12.3	2.67	81.1	0.11	3.4	0.09	2.8	0.00	0.0	841	10.0
1508	3.11	7.64	14.8	42.6	55.8	19.4	34.7	11.3	256.2	3.35	0.46	14.8	2.53	81.4	0.08	2.6	0.03	0.9	0.00	0.1	1042	9.2
1509	2.46	7.59	14.4	40.1	52.9	19.0	35.8	10.8	164.8	2.17	0.25	10.3	2.09	85.0	0.06	2.4	0.04	1.6	0.00	0.0	862	10.3
1510	2.69	7.51	14.6	40.9	54.5	19.4	35.6	10.8	191.3	2.55	0.27	10.2	2.30	85.5	0.07	2.5	0.04	1.6	0.00	0.1	996	9.1

Group 2, 3 mg/kg/day	WBC	RBC	HGB	HCT	MCV	MCH	MCHC	RDW	#RET	%RET	#NEUT	%NEUT	#LYMP	%LYMP	#MONO	%MONO	#EOS	%EOS	#BASO	%BASO	PLT	MPV
	(10^3/µL)	(10^6/µL)	(g/dL)	(%)	(fL)	(pg)	(g/dL)	(%)	(10^9/L.)	(%)	(10^3/µL)	(%)	(10^3/µL)	(%)	(10^3/µL)	(%)	(10^3/µL)	(%)	(10^3/µL)	(%)	(10^3/µL)	(fL)
2501	2.30	6.89	13.0	36.7	53.2	18.9	35.5	12.0	191.3	2.78	0.29	12.6	1.86	80.7	0.10	4.3	0.04	1.9	0.00	0.1	840	10.0
2502	2.62	6.82	13.3	37.8	55.4	19.5	35.1	10.8	296.9	4.35	0.34	12.8	2.19	83.6	0.06	2.4	0.03	1.0	0.00	0.0	953	10.7
2503	2.09	7.11	14.1	39.1	55.1	19.9	36.1	12.4	288.6	4.06	0.31	14.9	1.66	79.6	0.07	3.6	0.03	1.4	0.00	0.1	1091	10.3
2504	3.23	7.04	13.6	38.7	55.0	19.3	35.1	12.1	247.2	3.51	0.45	14.1	2.65	81.9	0.08	2.5	0.04	1.2	0.00	0.0	981	10.5
2505	3.08	6.85	13.4	37.6	54.9	19.5	35.6	12.6	258.2	3.77	0.58	18.9	2.38	77.2	0.05	1.5	0.06	1.8	0.00	0.0	823	10.6
2506	4.47	7.44	13.9	39.3	52.9	18.7	35.4	11.5	211.2	2.84	0.42	9.5	3.84	86.1	0.11	2.4	0.06	1.4	0.00	0.1	854	10.5
2507	3.47	7.58	14.8	42.6	56.1	19.5	34.8	10.7	300.0	3.96	0.36	10.4	2.99	86.3	0.05	1.5	0.05	1.3	0.00	0.1	1009	10.0
2508	5.69	7.50	14.6	42.0	56.0	19.5	34.9	10.6	234.7	3.13	0.59	10.4	4.88	85.7	0.12	2.1	0.06	1.1	0.01	0.1	1064	10.3
2509	2.10	7.37	13.8	39.2	53.2	18.7	35.2	10.8	180.6	2.45	0.16	7.6	1.87	89.0	0.04	1.9	0.02	0.9	0.00	0.1	869	9.5
2510	3.02	7.57	14.0	40.5	53.5	18.4	34.5	10.8	236.3	3.12	0.31	10.4	2.60	86.3	0.04	1.2	0.06	1.8	0.00	0.0	1124	9.1

Group 3, 6 mg/kg/day	WBC	RBC	HGB	HCT	MCV	MCH	MCHC	RDW	#RET	%RET	#NEUT	%NEUT	#LYMP	%LYMP	#MONO	%MONO	#EOS	%EOS	#BASO	%BASO	PLT	MPV
	(10^3/µL)	(10^6/µL)	(g/dL)	(%)	(fL)	(pg)	(g/dL)	(%)	(10^9/L.)	(%)	(10^3/µL)	(%)	(10^3/µL)	(%)	(10^3/µL)	(%)	(10^3/µL)	(%)	(10^3/µL)	(%)	(10^3/µL)	(fL)
3501	3.02	6.97	14.0	39.4	56.6	20.0	35.4	11.8	264.1	3.79	0.37	12.1	2.57	85.0	0.03	1.0	0.04	1.2	0.00	0.0	857	10.3
3502	2.92	7.19	13.8	38.8	54.0	19.1	35.4	11.4	192.5	2.68	0.35	12.0	2.43	83.1	0.10	3.3	0.04	1.3	0.00	0.0	1047	10.6
3503	2.14	7.54	13.4	38.6	51.2	17.7	34.6	12.3	256.1	3.40	0.34	16.0	1.69	79.0	0.06	2.8	0.04	1.9	0.00	0.1	963	10.0
3504	2.36	7.42	14.5	40.4	54.3	19.5	35.9	10.5	192.2	2.59	0.48	20.3	1.73	73.1	0.10	4.2	0.04	1.8	0.00	0.1	876	10.5
3505	3.33	7.69	14.2	41.1	53.4	18.4	34.5	10.6	251.7	3.27	0.44	13.1	2.72	81.6	0.10	3.0	0.06	1.9	0.00	0.1	1064	9.5
3506	3.69	7.40	14.3	39.8	53.8	19.3	35.9	11.3	200.9	2.71	0.23	6.3	3.34	90.6	0.05	1.4	0.04	1.0	0.01	0.2	866	9.5
3507	4.14	7.19	14.0	39.0	54.3	19.4	35.7	11.4	240.7	3.35	0.38	9.1	3.66	88.4	0.05	1.1	0.03	0.8	0.00	0.1	1088	9.4
3508	3.39	7.31	13.9	39.7	54.3	19.1	35.1	11.3	215.1	2.94	0.28	8.3	3.01	88.9	0.05	1.6	0.03	0.9	0.00	0.1	694	10.3
3509	2.67	7.18	13.6	39.4	54.9	18.9	34.5	11.1	255.4	3.56	0.33	12.4	2.24	83.9	0.07	2.5	0.02	0.9	0.00	0.1	945	9.8
3510	2.59	7.30	14.2	39.7	54.4	19.4	35.7	12.1	287.5	3.94	0.40	15.5	2.08	80.2	0.06	2.3	0.04	1.6	0.00	0.0	921	10.0

Group 4, 12 mg/kg/day	WBC	RBC	HGB	HCT	MCV	MCH	MCHC	RDW	#RET	%RET	#NEUT	%NEUT	#LYMP	%LYMP	#MONO	%MONO	#EOS	%EOS	#BASO	%BASO	PLT	MPV
	(10^3/µL)	(10^6/µL)	(g/dL)	(%)	(fL)	(pg)	(g/dL)	(%)	(10^9/L.)	(%)	(10^3/µL)	(%)	(10^3/µL)	(%)	(10^3/µL)	(%)	(10^3/µL)	(%)	(10^3/µL)	(%)	(10^3/µL)	(fL)
4501	2.96	6.85	13.7	38.2	55.8	20.1	36.0	12.6	325.9	4.76	0.78	26.5	2.06	69.5	0.08	2.7	0.03	1.1	0.00	0.0	888	10.4
4502	3.74	7.23	13.1	38.5	53.2	18.2	34.1	11.5	346.2	4.79	0.67	18.0	2.94	78.7	0.06	1.5	0.04	1.2	0.00	0.0	933	10.3
4503	3.13	7.45	14.4	40.4	54.2	19.3	35.6	10.6	184.9	2.48	0.50	16.0	2.50	79.8	0.07	2.4	0.04	1.2	0.00	0.0	978	10.7
4504	2.41	7.37	14.1	39.8	54.0	19.2	35.5	10.7	234.0	3.17	0.30	12.5	2.02	83.8	0.04	1.6	0.04	1.9	0.00	0.1	771	10.8
4505	3.14	6.74	13.5	37.8	56.1	20.0	35.7	11.5	229.2	3.40	0.28	9.1	2.77	88.0	0.05	1.6	0.03	0.9	0.00	0.0	877	10.5
4506	2.54	7.51	14.5	41.2	54.9	19.3	35.2	11.1	299.1	3.98	0.39	15.4	2.05	80.7	0.07	2.6	0.03	1.1	0.00	0.0	970	10.3
4507	4.57	7.33	14.2	40.4	55.1	19.3	35.1	11.9	228.3	3.11	0.70	15.3	3.59	78.5	0.21	4.6	0.06	1.3	0.00	0.0	884	10.1
4508	4.06	7.24	13.5	39.6	54.7	18.6	34.0	10.6	254.4	3.52	0.39	9.5	3.56	87.6	0.07	1.7	0.03	0.7	0.00	0.1	1035	9.5
4509	3.89	7.16	13.9	39.4	55.0	19.5	35.4	10.8	258.6	3.61	0.29	7.4	3.47	89.1	0.09	2.4	0.02	0.6	0.00	0.1	1021	9.8
4510	6.62	6.03	11.5	33.4	55.3	19.1	34.5	12.1	309.3	5.13	2.49	37.7	3.64	55.0	0.36	5.4	0.10	1.6	0.01	0.1	1236	10.1

Abbreviations

WBC	Leukocyte count
RBC	Erythrocyte count
HGB	Hemoglobin
HCT	Hematocrit
MCV	Mean Corpuscular Volume
MCH	Mean Corpuscular Hemoglobin
MCHC	Mean Corpuscular Hemoglobin Conc.
RDW	RBC Distribution Width
#RET	Reticulocytes, absolute
%RET	Reticulocytes, percent
#NEUT	Neutrophils, absolute
%NEUT	Neutrophils, percent
#LYMP	Lymphocytes, absolute
%LYMP	Lymphocytes, percent
#MONO	Monocytes, absolute
%MONO	Monocytes, percent
#EOS	Eosinophils, absolute
%EOS	Eosinophils, percent
#BASO	Basophils, absolute
%BASO	Basophils, percent
PLT	Platelet Count
MPV	Mean Platelet Volume
