# Supplementary material for: Reverse engineering of BNIP3 identifies a mitochondrial protective peptide
Source: Nat Commun. 2026 Jun 17;17:5359. doi: 10.1038/s41467-026-73993-2 (PMC13275919; doi:10.1038/s41467-026-73993-2)
Supplement: Supplementary file 9 — Reporting Summary [file 41467_2026_73993_MOESM9_ESM.pdf]

nature portfolio

Corresponding author(s): Tienush Rassaf, Ulrike Hendgen-Cotta

Last updated by author(s): 2026/04/24



# Reporting Summary

Nature Portfolio wishes to improve the reproducibility of the work that we publish. This form provides structure for consistency and transparency in reporting. For further information on Nature Portfolio policies, see our Editorial Policies and the Editorial Policy Checklist.

## Statistics

For all statistical analyses, confirm that the following items are present in the figure legend, table legend, main text, or Methods section.

| n/a | Confirmed | |
|---|---|---|
| ☐ | ☒ | The exact sample size (*n*) for each experimental group/condition, given as a discrete number and unit of measurement |
| ☐ | ☒ | A statement on whether measurements were taken from distinct samples or whether the same sample was measured repeatedly |
| ☐ | ☒ | The statistical test(s) used AND whether they are one- or two-sided<br>*Only common tests should be described solely by name; describe more complex techniques in the Methods section.* |
| ☐ | ☒ | A description of all covariates tested |
| ☐ | ☒ | A description of any assumptions or corrections, such as tests of normality and adjustment for multiple comparisons |
| ☐ | ☒ | A full description of the statistical parameters including central tendency (e.g. means) or other basic estimates (e.g. regression coefficient) AND variation (e.g. standard deviation) or associated estimates of uncertainty (e.g. confidence intervals) |
| ☐ | ☒ | For null hypothesis testing, the test statistic (e.g. *F*, *t*, *r*) with confidence intervals, effect sizes, degrees of freedom and *P* value noted<br>*Give P values as exact values whenever suitable.* |
| ☒ | ☐ | For Bayesian analysis, information on the choice of priors and Markov chain Monte Carlo settings |
| ☒ | ☐ | For hierarchical and complex designs, identification of the appropriate level for tests and full reporting of outcomes |
| ☒ | ☐ | Estimates of effect sizes (e.g. Cohen's *d*, Pearson's *r*), indicating how they were calculated |

*Our web collection on statistics for biologists contains articles on many of the points above.*

## Software and code

Policy information about availability of computer code

| | |
|---|---|
| Data collection | Adobe Photoshop CS6 and LiCOR Odyssey XF, Software Empiria Studio 2.2 for Western blot analyses and presentation, Vevo 2100 and 3100 Imaging Systems (FUJIFILM Visual Sonics) for echocardiography, Zeiss ELYRA PS.1 SIM/PAL-M/STORM/TIRF and LSM710 (confocal laser scanning microscope), AMG EVOS fl digital inverted microscope, Light Sheet Fluorescence Microscope Miltenyi Blaze, Biocytogen Extreme Genome Editing System (EGE; CRISPR/CAS9 based technology, Biocytogen Boston Corp. USA), Charles River Laboratories (Testing facility study no. 2865-001, pig study), Labcorp study No. 8453010 (Cardiovascular effects in dogs), Beacon Optofluidic system (Biocytogen Boston Corp, mouse monoclonal anti-BNIP3 antibody), Leica SP8 MP (confocal laser scanning microscope), Tecan HS 4800 microarray processing station, Jasco J-170 CD spectrometer, Jasco FP-8300 fluorescence spectrometer, BioRad GelDoc Imager, Orbitrap Fusion Lumos coupled to an Vanquish Neo ultra high performance liquid chromatography sytem (Thermo), ImageQuant system (Amersham), xCELLigence Real Time Cell Analyzer Cardio System (Agilent Technologies), BMG FLUOstar Omega Microplate Reader, Leica DMi 8 (fluorescence microscope), JEM 1400PLUS equipped with LaB6 cathodes (JEOL) and CMOS camera (TemCam-F416, TVIPS), Agilent 1290 Infinity II Bio LC system, Thermo Orbitrap Q Exactive Plus mass spectrometer, BMG Labtech CLARIOstar Plus, Leica S6E, Leica M80, Monolith NT.115 (NanoTemper Technologies), Logbook v5.3, NextDocs v.6.1, Provantis v, MS Excel v16.0, Ponemah 5.4x/6.5x |

| | |
|---|---|
| Data analysis | Odyssey Imaging System (LI-COR), Software Empiria Studio 2.2, Charles River, Testing facility study No. 2865-001, SAS v9.4, Labcorp study No. 8453010 Ponemah, SASv9.4, GenePix spot-recognition sofware v7.2 (Molecular Devices), DichroWeb (http://dichroweb.cryst.bbk.ac.uk/html/home.shtml), Modeller 9.15, NAMD2.9, CHARMM36 force field, RosettaFold (https://robetta.bakerlab.org/), AlphaFold2v2.2.3,ColabFold server 1.5.5, DeepFRI server, HDOCK web server(Profacgen), GraphPad Prism 10.0, MetaMorpheus (v1.0.3.), RTCA Cardio Software 1.1.0 (Agilent Technologies) ImageJ2 software 1.54 (NIH), VevoLAB 5.9.0 software, DSI(TM) digital data caputure system linked with a DSI(TM) Ponemah data processing 5.4/6.5x, MS-Dial 4.9.2, Metabo Analyst (web server), ImarisFileConverterx64 Imaris Software (BitPlane 10.01), GraphPad Prism 9, v10.6.1; MO.Affinity Analysis v.2.2.4 (NanoTemper Technologies), Microsoft Excel v16.0. |

For manuscripts utilizing custom algorithms or software that are central to the research but not yet described in published literature, software must be made available to editors and reviewers. We strongly encourage code deposition in a community repository (e.g. GitHub). See the Nature Portfolio guidelines for submitting code & software for further information.

## Data

Policy information about availability of data

All manuscripts must include a data availability statement. This statement should provide the following information, where applicable:

- Accession codes, unique identifiers, or web links for publicly available datasets
- A description of any restrictions on data availability
- For clinical datasets or third party data, please ensure that the statement adheres to our policy

The mass spectrometry proteomics data for the crosslinking experiments have been deposited to the ProteomeXchange Consortium via the PRIDE partner repository
(https://www.ebi.ac.uk/pride/archive/) with the dataset identifier PXD056758. All other data are available in the main text.
The mass spectrometry metabolomics data have been deposited to the MetaboLights repository (https://www.ebi.ac.uk/metabolights/) with the dataset identifier REQ20260326218191. All other data are available in the main text.
Source data are provided with this paper.

## Research involving human participants, their data, or biological material

Policy information about studies with human participants or human data. See also policy information about sex, gender (identity/presentation), and sexual orientation and race, ethnicity and racism.

| | |
|---|---|
| Reporting on sex and gender | *Use the terms sex (biological attribute) and gender (shaped by social and cultural circumstances) carefully in order to avoid confusing both terms. Indicate if findings apply to only one sex or gender; describe whether sex and gender were considered in study design; whether sex and/or gender was determined based on self-reporting or assigned and methods used. Provide in the source data disaggregated sex and gender data, where this information has been collected, and if consent has been obtained for sharing of individual-level data; provide overall numbers in this Reporting Summary. Please state if this information has not been collected. Report sex- and gender-based analyses where performed, justify reasons for lack of sex- and gender-based analysis.* |
| Reporting on race, ethnicity, or other socially relevant groupings | *Please specify the socially constructed or socially relevant categorization variable(s) used in your manuscript and explain why they were used. Please note that such variables should not be used as proxies for other socially constructed/relevant variables (for example, race or ethnicity should not be used as a proxy for socioeconomic status). Provide clear definitions of the relevant terms used, how they were provided (by the participants/respondents, the researchers, or third parties), and the method(s) used to classify people into the different categories (e.g. self-report, census or administrative data, social media data, etc.) Please provide details about how you controlled for confounding variables in your analyses.* |
| Population characteristics | *Describe the covariate-relevant population characteristics of the human research participants (e.g. age, genotypic information, past and current diagnosis and treatment categories). If you filled out the behavioural & social sciences study design questions and have nothing to add here, write "See above."* |
| Recruitment | *Describe how participants were recruited. Outline any potential self-selection bias or other biases that may be present and how these are likely to impact results.* |
| Ethics oversight | *Identify the organization(s) that approved the study protocol.* |

Note that full information on the approval of the study protocol must also be provided in the manuscript.

# Field-specific reporting

Please select the one below that is the best fit for your research. If you are not sure, read the appropriate sections before making your selection.

☒ Life sciences ☐ Behavioural & social sciences ☐ Ecological, evolutionary & environmental sciences

For a reference copy of the document with all sections, see nature.com/documents/nr-reporting-summary-flat.pdf

# Life sciences study design

All studies must disclose on these points even when the disclosure is negative.

| | |
|---|---|
| Sample size | No statistical methods were used to predetermine sample size (n). Number of sample was determined based on experimental approach, availability, feasibility required to obtain definitive results. |
| Data exclusions | Data were excluded only in rare cases due to major technical issues. |
| Replication | The number of independently conducted experiments is indicated in the legends of the figures and supplementary figures throughout the manuscript, with all replications completed successfully. |
| Randomization | The animals were randomly assigned to each experimental/control group. A random allocation of samples/specimens was not relevant in the other parts of the study. |
| Blinding | Investigators were not blinded to group allocation during data collection. For all data analyses, investigators were blinded. |

# Reporting for specific materials, systems and methods

We require information from authors about some types of materials, experimental systems and methods used in many studies. Here, indicate whether each material, system or method listed is relevant to your study. If you are not sure if a list item applies to your research, read the appropriate section before selecting a response.

## Materials & experimental systems

| n/a | Involved in the study | |
|---|---|---|
| ☐ | ☒ | Antibodies |
| ☐ | ☒ | Eukaryotic cell lines |
| ☒ | ☐ | Palaeontology and archaeology |
| ☐ | ☒ | Animals and other organisms |
| ☒ | ☐ | Clinical data |
| ☒ | ☐ | Dual use research of concern |
| ☒ | ☐ | Plants |

## Methods

| n/a | Involved in the study | |
|---|---|---|
| ☒ | ☐ | ChIP-seq |
| ☒ | ☐ | Flow cytometry |
| ☒ | ☐ | MRI-based neuroimaging |

## Antibodies

Antibodies used

HRP-labelled mouse monoclonal anti-DYKDDDDK Tag (Flag) antibody, 1:1,000 (clone 5A8E5, GenScript, A01428-100), anti-BNIP3 antibody, 200 ng/ml / 1:200 (clon 1C8, Biocytogen, custom made), anti-BNIP3 antibody, 1:100 (PA5-11402, Invitrogen), anti-BAX antibody, 1:1,000 (2D2, Cell Signaling, C2772), monoclonal rabbit anti-BAK antibody, 1:100/1:1,000 (clone D4E4, Cell Signaling, 12105), monoclonal mouse anti-BCL2-antibody, 1:100 (clone BCL-2-100, Invitrogen, 13-8800 ), anti-BIM antibody 1:200 (MA5-14848, Inivtrogen), anti-BID antibody 1:100 (MA5-17034, Invitrogen), goat anti-rabbit secondary antibody, 1:200,000 (CW800, LI-COR), anti-BAX antibody, 1:1,000 (2D2 Alexa Fluor-790, Cell Signaling, sc20067), mouse anti rabbit IgG, HRP-conjugated, 1:20,000 (polyclonal antibody, 2729, Cell Signaling), goat anti-mouse IgG2c HRP-conjugated secondary antibody, 1:20,000, 1:100,000 (Jackson Immuno, ab97255), anti-cytochrome c antibody 1:1,000 (clone 7H8.2C12, Abcam, ab13575), anti-activated BAX antibody (6A7, Santa Cruz, sc-23959), anti-ANT1 antibody, 1:2,000 (Abcam, ab 110322), anti-tubulin antibody, 1:4,000 (Abcam, 1b15246), Alexa Fluor 488-conjugated goat-anti-mouse secondary antibody, 1:200 (Invitrogen, A-11001),

Validation

All of antibodies were validated by vendors, other researchers or our group. We based specificity on their provided data sheets.The mouse monoclonal anti-BNIP3 antibody 1C8 was validated by Biocytogen and within the manuscript (Methods section 'Mouse monoclonal anti-BNIP3 antibody', Supplementary Fig. 2).
HRP-labelled mouse monoclonal anti-DYKDDDDK Tag (Flag) antibody (clone 5A8E5, GenScript),
Reactivity: M Application: ELISA/WB
https://www.genscript.com/antibody/A01428-THE_DYKDDDDK_Tag_Antibody_HRP_mAb_Mouse.html#tab_Overview

anti-BNIP3 antibody (clone 1C8, Biocytogen, custom made),
Reactivity: M Application: WB
https://biocytogen.com

anti-BNIP3 antibody (PA5-11402, Invitrogen),
Reactivity: H M Application: ICH/ICC-IF
https://www.thermofisher.com/antibody/product/BNIP3-Antibody-Polyclonal/PA5-11402

anti-BAX antibody (2D2, Cell Signaling, C2772),
Reactivity: H M R Mk Application: WB
https://www.cellsignal.com/products/primary-antibodies/bax-antibody/2772

monoclonal rabbit anti-BAK antibody (clone D4E4, Cell Signaling),
Reactivity: H M R Mk Application: WB/IP/ICH/IF/F

https://www.cellsignal.com/products/primary-antibodies/bak-d4e4-rabbit-monoclonal-antibody/12105?srsltid=AfmBOoqExs-SB6ML6zYzNB8n6n7Z8vNyY_5LwIBColHD6PbSjJ3iy6-0

monoclonal mouse anti-BCL2-antibody (cloneBcl-2-100, Invitrogen),
Reactivity: H M Application: WB/IP/ICH/
https://www.fishersci.com/shop/products/bcl-2-monoclonal-antibody-bcl-2-100/138800

anti-BIM antibody (MA5-14848, Inivtrogen),
Reactivity: H M R Application: F/ICH/IP/WB/IC
https://www.fishersci.com/shop/products/bim-monoclonal-antibody-k-912-7/PIMA514848

anti-BID antibody (MA5-17034, Invitrogen),
Reactivity: H Application: ELISA/F/ICH/WB/IC
https://www.fishersci.de/shop/products/bid-monoclonal-antibody-3c5/15307930?srsltid=AfmBOor2bryLDYN_-HMhzd3LTy-kK4af6RZEyKl_xrerNYdDKvgmEdCT

anti-BAX antibody (2D2 Alexa Fluor-790, Cell Signaling),
Reactivity: M R H Application: WB/IF/FCM
https://www.scbt.com/de/p/bax-antibody-2d2

anti-cytochorme c antibody (clone 7H8.2C12, Abcam),
Reactivity: H M R Rb Eq Application: WB
https://www.thermofisher.com/antibody/product/Cytochrome-C-Antibody-clone-7H8-2C12-Monoclonal/338500?ef_id=EAIaIQobChMIk5fumPGDlAMVwGpBAh2gvws6EAAYAiAAEgLC5_D_BwE:G:s&s_kwcid=AL!3652!3!803202487879!!!g!!!10950825775!201728129904&cid=bid_pca_aup_r01_co_cp1359_pjt0000_bid00000_0se_gaw_dy_pur_con&gad_source=1&gad_campaignid=10950825775&gbraid=0AAAAADxi_GT3ExT187KR-5gXZCkdiHFpr&gclid=EAIaIQobChMIk5fumPGDlAMVwGpBAh2gvws6EAAYAiAAEgLC5_D_BwE

anti-activated BAX antibody (6A7, Santa Cruz),
Reactivity: M R H Application: WB/IP/IF/ICH
https://www.scbt.com/de/p/bax-antibody-6a7?srsltid=AfmBOopJHPoHOanX3g7uGmFD_AK95cUHTtnOWZZB2jMcxh347YLf_fz8

anti-ANT antibody (AbcamAb 110322),
Reactivity: H M R B Applications: ICC-IF/WB
https://www.citeab.com/antibodies/708969-ab110322-anti-adenine-nucleotide-translocase-1-ant-1

anti-tubulin antibody (Abcam, 1b15246),
Reactivity: H Applications:ICH-P/WB
https://www.abcam.com/en-us/products/primary-antibodies/alpha-tubulin-antibody-loading-control-ab15246

## Eukaryotic cell lines

Policy information about cell lines and Sex and Gender in Research

| | |
|---|---|
| Cell line source(s) | human iPSC-derived ventricular cardiomyocytes (Axol, Bioscience, Ax2505) human cardiac fibroblasts (PromoCell, C-12375), mouse embryonic fibroblasts wild-type (SV 40, ATCC-CRL 2907), Bcl-2 KO (SV40, ATCC-CRL-2908), Bax KO (SV40, ATCC-CRL 2910), Bak KO (SV40, ATCC-CRL 2912), Bax Bak KO (SV40, ATCC-CRL-2913), from ATCC, USA, HepG2, HEK293, MCF-7 cell lines were provided by A. Schramm (West German Cancer Center, University Hospital, Essen) and authenticated by the Leibniz-Institute DSMZ, Germany. Adult cardiomyocytes and fibroblast isolated from C57BL/6J wild-type, Bnip3-/-, Flag-Bnip3(ki/ki), Flag-Bnip3-/- mice. |
| Authentication | Human CMs were authenticated by measuring troponin I. All cell lines used were authenticated by the Leibniz-Institute DSZM, Germany. They have carried out DNA profiling using 17 different and highly polymorphic short tandem repeat (STR) loci. They have tested for presence of mitochondrial DNA sequences from rodent cells as mouse, rat, Chinese and Syrian Hamster. In addition, the mouse embryonic fibroblast cell lines have been subjected to the procedure of Cytochrome C Subunit I DNA barcoding for identification of the species. |
| Mycoplasma contamination | All cell lines were testet negative for mycoplasma contamination |
| Commonly misidentified lines (See ICLAC register) | No commonly misidentified lines were used. |

## Animals and other research organisms

Policy information about studies involving animals; ARRIVE guidelines recommended for reporting animal research, and Sex and Gender in Research

| | |
|---|---|
| Laboratory animals | All relevant mice experiments: C57BL/6J wild-type mice from Janvier Lab (France), 12 +/-3 weeks, male<br>All relevant mice experiments: C57BL6/J-TgH (Bnip3-/-) mice were bred and maintained in the local animal house of the University |

Hospital Essen, C57BL/6 background, 12+/-3 weeks, male
All relevant mice experiments: C57BL/6 Bnip3-3xFlag-knockin mice were bred and maintained in the local animal house of the University Hospital Essen, generated with the Biocytogen Extreme Genome Editing System, 12+/-3 weeks, male, C57BL/6J background
All mice were housed on a 12-h light/dark cycle, at a temperature of 20-24°C and humidity of 40% bis 60%. Bevor using, the mice were kept for 1 week in the local animal house for acclimatisation. Mice of similar age and body weight were used for ischemia/reperfusion surgeries.
Myocardial I/R injury:Naive Domestic Yorkshire crossbred swine (farm pigs). The animals were received from Oak Hill Genetics, Ewing, Illinois. The experiments were performed by Charles River, Mattawan, Michigan, USA, 12-13.5 week, housed in stainless steel runs with raised flooring, fluorescence lighting for 12 h per day, temperature of 69°F to 79°F, humidity of 30% - 70%
Liver I/R injury: Wistar Han rats, male, body weight ranging from 250g to 300g, 8-9 weeks, Charles River, Germany
Toxicology Study: Wistar Han rats, female and male, body weight ranging from 154g to 194g (female) and 239g to 284g (male), Study performed by WuXi, China, 8-9 weeks, 12h light/dark cycle, 22.3°C to 24.6°C, humidity of 44,4% to 78,1%. The animals were received from Wistar Han, Beijing Vital River Laboratory Animal Technology Co., Ltd.
Cardiovascular function: Beagle dogs, male, body weight ranging from 9.2 to 10.1 kg, Study performed by Labcorp Early Development Laboratories, UK, 27-28 weeks, 12h light/dark cycle, 13 to 23°C. The dogs were received from Marshall Bioresource, UK.

Wild animals

Study did not involved wild animals.

Reporting on sex

The toxicological and toxicokinetics studies were conducted on female (n=50) and male rats (n=50) . The other experiments were conducted on male animals. Cell lines from female (MCF-7, HEK293) and male donors (HCM, HCF, HepG2) were used.

Field-collected samples

No field collected samples were used in the study.

Ethics oversight

- All mice procedures were performed in accordance with institutional guidelines and approval from the local ethics committee in compliance with the European Convention for the Protection of Vertebrate Animals Used for Experimental and other Scientific Purposes (Directive 2010/63/EU). Approved by the Landesamt für Natur, Umwelt und Verbraucherschutz Nordrhein-Westfalen, Germany, 84-02.04.2014.A144, 81-02.04.2019.A369.
- The swine I/R surgeries were performed in accordance with The Animal Welfare Act (9 CFR Parts 1, 2, and 3) of the US Department of Agriculture (USDA), and the Guide for the Care and Use of Laboratory Animals, Institute of Laboratory Animal Resources, National Academy Press, Washington, DC, 2011. The study was approved by the Testing Facility Institutional Animal Care and Use Committee.
- All studies in dogs were conducted in accordance with GLP regulations 1999, Statutory Instrument 1999 No. 3106 from the United Kingdom GLP Monitoring Authority. Medicines and Healthcare Products Regualtory Agency (MI-IRA) and Principles on Good Laboratory Practice. ENV/MC/CHEM (98) 17 (revised in 1997, issued January 1998) from the Organisation for Economic Co-Operation and Development. The study was approved by the Institutional Animal Care und Use Committee (IACUC).
- The protocol and any amendments or procedures involving the care or use of rats on the toxicological study had been reviewed and approved by WuXi AppTec Institutional Animal Care and Use Committee (IACUC) prior to the initiation of such procedures. A staff veterinarian monitored the study for animal welfare issues. All applicable portions of the study conformed to the AAALAC International guidelines as reported in the Guide for the Care and Use of Laboratory Animals, National Research Council (2011) and The People's Republic of China, Ministry of Science & Technology, "Regulations for the Administration of Affairs Concerning Experimental Animals," 2017.
- The procedure of rat killing with intracardially injection of potassium chloride in deep isoflurane anaesthesia for organ retrieval according to §4 Abs. 3 Tierschutzgesetz (German Legislation on animal protection) has been approved by the animal welfare officers of the University Hospital Essen.

Note that full information on the approval of the study protocol must also be provided in the manuscript.

## Plants

Seed stocks

*Report on the source of all seed stocks or other plant material used. If applicable, state the seed stock centre and catalogue number. If plant specimens were collected from the field, describe the collection location, date and sampling procedures.*

Novel plant genotypes

*Describe the methods by which all novel plant genotypes were produced. This includes those generated by transgenic approaches, gene editing, chemical/radiation-based mutagenesis and hybridization. For transgenic lines, describe the transformation method, the number of independent lines analyzed and the generation upon which experiments were performed. For gene-edited lines, describe the editor used, the endogenous sequence targeted for editing, the targeting guide RNA sequence (if applicable) and how the editor was applied.*

Authentication

*Describe any authentication procedures for each seed stock used or novel genotype generated. Describe any experiments used to assess the effect of a mutation and, where applicable, how potential secondary effects (e.g. second site T-DNA insertions, mosiacism, off-target gene editing) were examined.*
