# Supplementary material for: Reverse engineering of BNIP3 identifies a mitochondrial protective peptide
Source: Nat Commun. 2026 Jun 17;17:5359. doi: 10.1038/s41467-026-73993-2 (PMC13275919; doi:10.1038/s41467-026-73993-2)
Supplement: Supplementary file 10 — Transparent Peer Review file [file 41467_2026_73993_MOESM10_ESM.pdf]

nature portfolio

Peer Review File

Reverse engineering of BNIP3 identifies a mitochondrial protective peptide.

Corresponding Author: Professor Ulrike Hendgen-Cotta

This file contains all reviewer reports in order by version, followed by all author rebuttals in order by version.

Version 0:

Reviewer comments:

Reviewer #1

(Remarks to the Author)
This study employed a systematic experimental design to investigate the binding mechanism between BNIP3 and Bax, leading to the identification of BNIP3 13-20 amino acid peptide. The study then developed B-017 peptide and validated its efficacy and mechanism through cellular experiments (mitochondrial apoptosis assay) and animal models (cardiac, cerebral, and hepatic ischemia). The work is well-conceived and the data is substantial. To further strengthen the manuscript and ensure its conclusions, I have several suggestions aimed at bolstering the mechanistic insights and data robustness.

1. Animal study administration routes: The route of administration for B-017 is unclear. Please specify whether intracardiac or intravenous (e.g., tail vein) injection was used in the myocardial and cerebral ischemia models. While B-017 was administered via intracardiac injection in acute myocardial ischemia models, the subsequent consecutive administration route should be explicitly stated. The administration route for cerebral ischemia models also needs clarification and supplementation.

2. Morphological data completeness: Quantitative analyses should be supplemented with representative qualitative images to enhance evidentiary value.
Representative TTC-stained images for the porcine myocardial ischemia-reperfusion model (Extended Data Fig. 17d) are highly encouraged. This data holds stronger clinical relevance and warrants presentation in the main figure.
Please provide representative fluorescence micrographs for assays like Rho-2 AM, JC-1, and Calcein (Fig. 4), which are crucial for data authenticity and experimental success.
Additionally, TUNEL staining in myocardial ischemia models to confirm apoptosis is recommended.

3. Stroke model details and functional assessment: Please clearly state the administration route used in the tMCAO model (Extended Data Fig. 19a). Furthermore, neurological deficit, a critical parameter for evaluating stroke severity and drug efficacy, could be supplemented in data.

4. Mechanistic validation: To further elucidate the relationship between B-017's mechanism and BNIP3/Bax, it is recommended to evaluate whether the protective effects of B-017 are attenuated or abolished in BNIP3 knockout mice (already established in this study)or Bax knockout mice. This would provide crucial genetic evidence for its mechanism.

5. Extended Fig. 20 mitochondrial apoptosis pathway analysis:
In Extended Data Fig. 20b, the use of ANT1 as a mitochondrial loading control is inappropriate due to its variable expression under stress like ischemia. It is recommended to use more stable loading control like VDAC1 or COX IV.
In Extended Data Fig. 20c, the release of Cyto C from mitochondria into the cytosol is a core event in apoptosis. It is recommended to simultaneously present data for Cyto C levels in both the cytosolic and mitochondrial fractions.
In addition, variability in loading control bands across both panels undermines result reliability.

6. Necrosis pathway indicators: The manuscript states that "B-017 inhibits both necrosis and apoptosis". While apoptosis pathways are assessed via Bax, caspase-3/7, and Cyto c, current data lack specific indicators validating necrosis pathway.

7. Potential off-target effects and toxicity: To better address specificity, could the authors discuss or provide data on whether B-017 interacts with other key BCL-2 family proteins (e.g., anti-apoptotic members like BCL-2)? Furthermore, for the long-

term studies, data on animal body weight and histological analysis (HE staining) of major organs (heart, liver, kidney) could be provided for preliminary safety evaluation.

8. In vivo distribution of the peptide: Current data only show macroscopic cardiac distribution (Extended Data Fig. 17a). It is recommended to provide distribution data for B-017 in other key target organs, particularly the brain and liver. This could include measurements of tissue concentration or visualization of its distribution at the cellular level within tissues using immunofluorescence.

Reviewer #2

(Remarks to the Author)
Comments for Authors

Hendgen-Cotta et al. performed a wide range of experiments to design an inhibitor of BAX/BNIP3 to suppress death signaling in vitro and in vivo, with the goal of attenuating I/R injury. They employed a variety of cellular and animal models to demonstrate the efficacy of their therapeutic peptide, B-017. The notion of reverse engineering is certainly interesting. The authors also made a great effort explaining their logic, as well as the details of the experiments. The potential application areas, including myocardial I/R, cerebral I/R, and liver transplantation, of BNIP3/BAX inhibition are also of clinical interest. However, the current study, as presented, suffers from insufficient data to support their conclusions, as well as concerns regarding the proposed mechanism of action of B-017. Detailed comments are listed below.

Fig. 1a: BNIP3-BAX/BAK PLA experiment in Bak-/- and Bax-/- MEFs.
First of all, validation of the absence of the two proteins in MEFs is lacking. Then, the authors discussed the lack of BNIP3-BAX signal in Bax-/- MEFs as a dependence of BAK1 orientation on BAX (a reference should be cited). While this is certainly possible, the authors did not rule out the possibility that BAX deletion impacts BAK1 expression, and vice versa. A simple experiment would be to use Western blotting to detect the BAX and BAK1 expression in Bak-/- and Bax-/- MEFs, respectively. In addition, the authors did not discuss the reduced BNIP3-BAK PLA signal in Bak-/- MEFs.

The use of circular dichroism spectroscopy is not explained, which is not a standard tool to determine protein tertiary structure. This method evaluates chirality, which is distinct from protein structure. The authors state that "recombinant hBNIP3 exhibited a comparable tertiary structure to recombinant hBAX as determined by CD spectroscopy (Extended Data Fig. 7a)" (Lines 158-160). However, their information was not shown side by side, and could not be compared. Also, is CD sufficient to tell tertiary structure? From the data shown in Extended Data Fig. 7b, it is only a statistical summary of the secondary structures within a protein.

Fig. 2b: The in vitro BAX activity assay, as explained in the Methods, is essentially immunoprecipitation using an anti-BAX (6A7) antibody. If Lane 6 (left blot) is input, then it should be labeled as such. Why are there so many bands for BAX? The IgG is applied to the wrong group. It should be used instead of 6A7, where peptides/proteins are added to activate BAX. A negative control is also lacking (a protein/peptide that is known to not activate BAX). Lanes 3 and 4 are exactly the same. In Lane 2, it is not clear why IgG is added in addition to an anti-BAX (6A7) antibody. The right blot also lacks a negative control, such that we cannot rule out that it is inherently activated.

The main logical flaw of this study is the rationale for designing a BNIP3 antagonist starting with a truncated BNIP3 peptide. The BNIP3 peptide 1-49 was shown to interact with BAX in Fig. 2a and 2b. Then, the authors started to use screen binding of mBNIP3 with mBNIP3 N-terminal peptides (starting Line 288), which has no basis. Why is BNIP3 supposed to interact with BNIP3 in an N-terminal fashion? Preceding data focused on showing BNIP3 interaction with BAX. In addition, the authors stated that "BNIP3 is known to form a stable homodimer via the coiled-coil formation of its C-terminal helix" (Line 189), and not the N-terminus.
In addition, given that the N-terminus of BNIP3 was shown to activate BAX, it would be logical to devise an inhibitor to this protein-protein interaction (either something that covers the 1-49 part of BNIP3 or binding to the corresponding BAX hotsopt without activating it), and not determining the sequence of BNIP3 amino acids that binds to BNIP3, as was done in this study.
Furthermore, peptide 1-49 was shown to activate BAX (Fig. 2b). What is the rationale for using it as a starting material to design an antagonist, which has the opposite function?
For the determination of the antagonist in Fig.2, BAX activity measurements were not shown. (did not appear until Fig.3b). Then, this part of the results section cannot use a title containing 'antagonist'.
It is also not clear why the peptide also derived from a BAX-activating peptide, and then matched the inactive form (Line 307). Does it bind active and inactive BAX equally? Or this there a binding preference?

In Line 327 and 333, the authors mentioned the binding of B-017 to BAX/BNIP3 to form a ternary complex, which was not mentioned earlier, and the data do not support their conclusions. First of all, there is no computational model nor experimental validation of the BAX/BNIP3/B-017 complex.
Secondly, if such a ternary complex existed, how does it work? The authors showed that B-017 binds both BNIP3 and BAX (Line 320). But if BNIP3 binds to BAX using its N-terminus, then how can a N-term-derived BNIP3 peptide bind to them? This is not logically possible.
Furthermore, If BNIP3 does not need stimulus to bind BAX (Fig. 1b), then is BAX constitutively active? Does B-017 compete with BNIP3 for BAX binding? In Fig. 3d, it seems that B-017 competes with BNIP3. Is its affinity higher? All of these questions are not clarified.

Fig.3b. Staurosporine (STS) is used to stimulate cell death. But this experiment was an in vitro (cell-free) assay. What is the rationale for using STS? Even if it were a cellular assay, why use STS if BNIP3-BAX interact at base level (Fig. 1b)? Does STS enhance BNIP3-BAx interaction (not shown in the data)?

Line 448 “It can be inferred that BAX may also be activated indirectly through the inhibition of BCL-2.” This is not evident in the data shown. BCL2 did not appear in any preceding experiment.

Fig. 3d How does B-017 suppress apoptosis in the absences of Bax? Off-target effect? The authors briefly touched upon this point in line 458. But what are the implications? B-017 also lowered the signal in BAX/BAK double-KO cells, suggesting additional mechanisms. From the degree of the B-017 effect, it almost seems that it is a stronger inhibitor of BAK1 than of BAX (3d right, 3e left and middle). Do the authors have any comments regarding the specificity/selectivity of B-017?
On a sidenote, the legend for Fig. 3d right indicates n = 2 in knockout MEFs but the figure shows 3 data points. N=2 is clearly not sufficient for statistical analysis.
Extended data Fig. 14e why is the baseline lower in the BCL2-/- group compared to WT?

Fig. 4. The use of cellular impedance as a measure/surrogate of cell viability/death is not warranted. In addition to cell attachment, impedance can also change with cell size, shape, etc. Common cell viability assays should provide additional information.

Line 638. The pig model is a good model for investigation. But why were very few data gained from this experiment? What happened to the pigs later? Any data?

Line 649. Dogs were used for toxicity measurement at baseline. Why change models so frequently (and then back to mice)?

The authors continued to use a wide variety models to test their peptide, which is good in the sense that it shows various potentially applications, but lack in-depth analysis and consistency. Too many cell, animal and disease models were used in general.

Fig. 5a and 5d. Lack of sham control. Fig. 5d. ‘Reduction in cTnI was accompanied by minor mitochondrial swelling’ It seems the other way around, i.e. reduced swelling. Fig. 5e. Fragementation not visible. This is how it normally looks like in vivo, and also depends on the specific site in the cell. The authors should provide additional images showing larger areas, as well as rigorous quantification.

Finally, on a broader scale, how does B-017 compare to established Bax inhibitors? How does Bax or BNIP3 depletion itself impact I/R?

Minor comments.
1. Overall, the figure legends are severely overloaded. Many of the method details and results descriptions in the legends should go in the Methods.
2. Line 144 mBNIP3-His?
3. Labeling of Extended Data Fig. 5a is not sufficiently clear to the reader. Does IPCr mean IgG control? What do the * in the figure indicate? What antibody was used for IP in each blot?
4. Color scale missing in Extended Data Fig. 11b.
5. Fig. 3a: were proteins normalized to total protein? Repeated-measures ANOVA should be used instead of one-way ANOVA. N=3 (legend) or n=4 (figure)?
6. Figure quality needs to be improved in many cases. For example, Fig. 3c left blot, Fig. 5e TEM images, Fig.5a TTC/Evans Blue stains, etc.
7. Line 613. Rationale for using 12 month-old mice? Normally younger mice are used.

Reviewer #3

(Remarks to the Author)
The manuscript presents a compelling reverse-engineering approach to develop a mitochondrial protective peptide, B-017, derived from BNIP3. This comprehensive study integrates structural modeling with in vitro, cellular, and in vivo models of ischemia/reperfusion injury. The innovative concept of targeting the BNIP3-BAX/BAK1 axis to simultaneously inhibit apoptosis and necrosis holds significant potential. However, to meet the rigorous standards of Nature Communications, substantial revisions are necessary, particularly concerning data presentation, statistical rigor, mechanistic clarity, and depth of evidence.
Major Concerns:
1.Experiments should be conducted to compare the protective effects of B-017 in wild-type versus Bnip3-/- mice subjected to I/R injury. A significant reduction or abolition of protection in Bnip3-/- mice would confirm BNIP3 as the essential target. If feasible, studies in Bnip3 and Bax double-knockout models (cell lines or mice) are recommended. The loss of B-017's efficacy in these models would provide the strongest evidence for its dependence on the BNIP3-BAX axis.
2.All typographical and numerical inconsistencies, especially in figure legends, must be corrected. For instance, the dose of B-017 in Fig. 5a should be reconciled, as it is currently reported variably as 8 mg/kg and 0.8 mg/kg.
3.Sham-operated control groups should be included in all in vivo experiments (Fig. 5 and related supplements) to properly establish baseline conditions and injury-specific effects.

4.A schematic diagram should be included to summarize the proposed mechanism of B-017, illustrating its interactions with BNIP3 and BAX/BAK1 and the resulting mitochondrial protection pathway.
5.The metabolomics analysis in Fig. 5i should be strengthened by directly linking specific metabolic alterations—such as changes in TCA cycle intermediates—to the observed improvements in mitochondrial function and protection, thereby providing deeper biological insight.
6.All figures must be re-exported at high resolution with complete and clear labels. For example, the incomplete y-axis label in Fig. 2e should be corrected, and the clarity of Fig. 2f should be enhanced.
7.The specificity of B-017 should be assessed by evaluating potential off-target interactions with other BCL-2 family proteins (e.g., BCL-2, MCL-1) using binding assays such as SPR or ITC.
8.A comprehensive safety profile is required, including an evaluation of organ toxicity, immune responses, and key pharmacokinetic parameters (e.g., plasma stability, tissue distribution, clearance, and half-life) to robustly support the therapeutic potential of B-017.
9.Testing B-017 in chronic disease models involving BNIP3/BAX dysregulation (e.g., chronic heart failure, neurodegeneration, or NASH) is recommended to explore its potential for long-term efficacy.
10.High-resolution respirometry should be performed to quantitatively assess mitochondrial oxygen consumption rates in cells or tissues treated with B-017 following I/R injury. This direct functional assessment would strongly support the conclusion of mitochondrial protection.

Version 1:

Reviewer comments:

Reviewer #1

(Remarks to the Author)
The authors have addressed my previous concerns thoroughly with substantial new data. I appreciate their efforts. However, a few minor issues remain:

1. Supplementary Fig. 22a (neurological function): The data presentation is unclear. Please specify what the data points represent, add statistical information, and present as a scatter plot with median.

2. Supplementary Fig. 23c (mitochondrial cytochrome c): There is a discrepancy: legend says lanes 3-5 are I/R, but blot labels them as sham. Please clarify. The representative blot images mentioned in the rebuttal are not included in the revised Supplementary Figures. Please provide them alongside the cytosolic data. In addition, the mitochondrial cytochrome c blot lacks a mitochondrial loading control (e.g., VDAC1/COX IV) to verify equal loading across lanes.

3. The ANT1 Western blot images mentioned in the rebuttal are also missing from the Supplementary figures. Please include them.

4. Fig. 6a (mitochondrial swelling): The figure shows that the Ctrl peptide group has lower mitochondrial swelling, while the B-017 group shows higher swelling. This appears to contradict the statement that B-017 prevents swelling. Please clarify this inconsistency.

I have reviewed the authors' response to Reviewer 3.

Overall, the authors have made substantial efforts and added considerable new data. I appreciate their work. However, a few issues remain that should be addressed or acknowledged:

1. Mechanism diagram (Supplementary Fig. 25b) is incomplete. It only depicts the heart, but the study also includes brain ischemia and liver ischemia models. Please expand the diagram accordingly.

2. Sham controls are still missing. Especially key figures including Fig. 5b, 5d/e, and particularly Fig. 5f lack sham-operated controls. Without a sham group, it is difficult to assess baseline function or the degree of recovery.

3. The chronic heart failure study (Fig. 5f) is underpowered. Only LVEF is shown without other cardiac function indices. Besides, standard endpoints such as histological assessment of fibrosis (e.g., Masson's trichrome) and heart weight/tibia length ratio are not provided.

If these data are not available, the authors should acknowledge these as limitations.

Reviewer #2

(Remarks to the Author)

The authors have addressed most of my concerns.The manuscript has much improved in terms of clarity.

Open Access This Peer Review File is licensed under a Creative Commons Attribution 4.0 International License, which permits use, sharing, adaptation, distribution and reproduction in any medium or format, as long as you give appropriate credit to the original author(s) and the source, provide a link to the Creative Commons license, and indicate if changes were made.
In cases where reviewers are anonymous, credit should be given to 'Anonymous Referee' and the source.
The images or other third party material in this Peer Review File are included in the article's Creative Commons license, unless indicated otherwise in a credit line to the material. If material is not included in the article's Creative Commons license and your intended use is not permitted by statutory regulation or exceeds the permitted use, you will need to obtain permission directly from the copyright holder.
To view a copy of this license, visit https://creativecommons.org/licenses/by/4.0/

Point-by-point response to Reviewers relating to NCOMMS-25-61408-T
Manuscript Title: **Reverse engineering of BNIP3 identifies a mitochondrial protective peptide by Hendgen-Cotta et al.**

Reviewer Comments & Author Responses

Reviewers' Comments:

We thank the Reviewers for their thorough examination and constructive remarks, which have significantly strengthened this manuscript. Below, you will find a point-by-point reply to each of the Reviewers' comments, with comments shown in **bold.** All changes and edits in the revised manuscript are highlighted in yellow. Information about line numbers of the revisions refers to the revised manuscript version.

Reviewer #1 (Remarks to the Author):

This study employed a systematic experimental design to investigate the binding mechanism between BNIP3 and Bax, leading to the identification of BNIP3 13-20 amino acid peptide. The study then developed B-017 peptide and validated its efficacy and mechanism through cellular experiments (mitochondrial apoptosis assay) and animal models (cardiac, cerebral, and hepatic ischemia). The work is well-conceived and the data is substantial. To further strengthen the manuscript and ensure its conclusions, I have several suggestions aimed at bolstering the mechanistic insights and data robustness.

We thank the Reviewer for their suggestions, which have greatly helped us to improve the quality of our work.

1. Animal study administration routes: The route of administration for B-017 is unclear. Please specify whether intracardiac or intravenous (e.g., tail vein) injection was used in the myocardial and cerebral ischemia models. While B-017 was administered via intracardiac injection in acute myocardial ischemia models, the subsequent consecutive administration route should be explicitly stated. The administration route for cerebral ischemia models also needs clarification and supplementation.

In the mouse myocardial ischaemia/reperfusion (I/R) injury model, B-017 was indeed administered via intracardiac injection five minutes before reperfusion to evaluate the infarct size and downstream signalling. To investigate left ventricular function, B-017 was consecutively administered intraperitoneally. Pigs were injected with B-017 as an intravenous bolus injection through the femoral vein. For cerebral ischaemia, B-017 was injected intravenously. The administration routes are now specified in required detail in the Results ***(lines 436, 452, 459, 472, 473, 497)*** *and Methods section (****lines 1139-1140, 1142-1143, 1161, 1185, 1201, 1207, 1209****) of the revised manuscript, and in the figure legends accordingly.*

2. Morphological data completeness: Quantitative analyses should be supplemented with representative qualitative images to enhance evidentiary value.
Representative TTC-stained images for the porcine myocardial ischemia-reperfusion model (Extended Data Fig. 17d) are highly encouraged. This data holds stronger clinical relevance and warrants presentation in the main figure.
Please provide representative fluorescence micrographs for assays like Rho-2 AM, JC-1, and Calcein (Fig. 4), which are crucial for data authenticity and experimental success. Additionally, TUNEL staining in myocardial ischemia models to confirm apoptosis is recommended.

*We have now included the infarct size results for the porcine myocardial I/R model in **new Fig. 5c**. Representative TTC-stained images of the porcine hearts (**new Supplementary Fig. 20f**), along with representative fluorescence micrographs for assessing H_2O_2 in HEK293 (Fig. 4c, **new Supplementary Fig. 16a**), MCF-7 cells and HCF (**new Supplementary Fig. 17a**), calcium levels in HCM (**new Supplementary Fig. 17e**), and mitochondrial membrane potential in iPSC-derived cardiomyocytes (Fig. 4f, **new Supplementary Fig. 16b**) are now also included in the revised manuscript. Caspase 3/7 activity in MCF-7 and mCF (Fig. 4b, Supplementary Fig. 17c), H_2O_2 in HCM (Fig. 4c, Supplementary Fig. 17d), mitochondrial calcium levels in HCM (Fig. 4d), mPTP in HCM (Fig. 4e) and mitochondrial membrane potential in HCM (Fig. 4f) and MCF-7 (Supplementary Fig. 17b) were measured with the BMG FLUOstar Omega Microplate Reader.*

*Following the Reviewer's advice, we quantified apoptosis at 4 h reperfusion following 30 min of ischaemia using TUNEL staining. The data reveal a significant reduction of apoptotic signal events in the left ventricle when treated with B-017 (0.8 mg kg^{-1} body weight, administered intracardially) compared to the negative control peptide treatment. These results are presented in **new Figure 6f** of the revised manuscript. The results and the method have been added to the Results (**line 534**) and the Methods section (**lines 1291-1298**).*

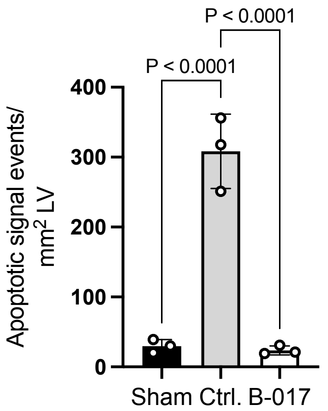

***New Fig. 6f:** Apoptosis at 4 h reperfusion. The total numbers of apoptotic signal events per left ventricle (LV) was counted in four plane sections of the heart with three slices each (Ctrl. and B-017 group) and one each (sham group) and calculated per 1 mm^2 of LV tissue. Images were captured using an inverted epifluorescence microscope and processed in ImageJ software 1.52a (NIH). Ctr., negative control peptide. (n = 3 C57BL/6J mice per group).*

3. Stroke model details and functional assessment: Please clearly state the administration route used in the tMCAO model (Extended Data Fig. 19a). Furthermore, neurological deficit, a critical parameter for evaluating stroke severity and drug efficacy, could be supplemented in data.

*All animals with tMCAO underwent intravenous treatment. We also performed functional behavioural tests on all the animals, now shown in Supplementary Fig. 22a (formerly Extended Data Fig. 19a), 24 h after the stroke. The Bederson Score assessment showed a tendency towards fewer neurological deficits in the B-017-treated group compared to the control group. We have included information on the administration route (**line 496**), a modified Bederson neurological scale (**Supplementary Table 14**), and the results and methods in the Results (**lines 495-496, 498-500**) and Methods sections (**lines 1185, 1264-1266**). The data are now presented as **new Supplementary Fig. 22a** in the revised manuscript.*

Supplementary Table 14. The modified Bederson's neurological deficit scale

Score	*signs*
0	Normal
1	Flexion of the left front paw
2	Flexion of the left front paw and decreased resistance to lateral push
3	Circling to one side
4	Circling and spinning around the cranial-caudal axis, loss of walking or righting reflex
5	Comatose or moribund

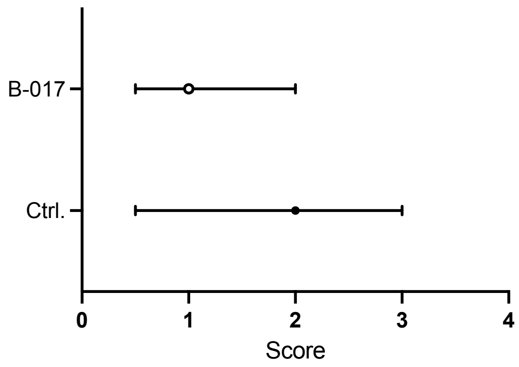

New Supplementary Fig. 22a: Assessment of neurological function 24 h postoperatively in vehicle (Ctr., 0.9% sodium chloride) and B-017-treated mice, using the Bederson score (n = 5 per group).

4. Mechanistic validation: To further elucidate the relationship between B-017's mechanism and BNIP3/Bax, it is recommended to evaluate whether the protective effects of B-017 are attenuated or abolished in BNIP3 knockout mice (already established in this study) or Bax knockout mice. This would provide crucial genetic evidence for its mechanism.

*To further elucidate the relationship between B-017's mechanism and BNIP3/BAX, we have conducted myocardial I/R injury in Bnip3 knockout mice. We identified that Bnip3 deletion generally protected cardiac tissue from I/R injury, as evidenced by a 51% reduction in infarct size compared to wild-type mice. However, intracardiac treatment of Bnip3 knockout mice with B-017 five minutes before the onset of reperfusion provided no additional protection. This finding supports the proposed relationship. We have added these results and the methods to the Results **(lines 516-524)** and Methods section **(line 1118)**. The data are now shown in **new Supplementary Fig. 23a** of the revised manuscript.*

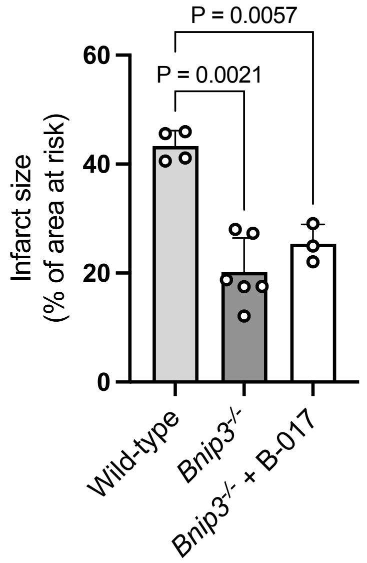

***New Supplementary Fig. 23a:** Therapeutic response to the intracardiac injection of B-017 in Bnip3 knockout ($Bnip3^{-/-}$) mice, compared to untreated wild-type and $Bnip3^{-/-}$ mice, in ischaemia/reperfusion. Mice were exposed to 30 min of ischaemia, followed by 24 h of reperfusion. Infarct size measurement (infarcted tissue per AAR) showed that Bnip3 deletion generally protected cardiac tissue from I/R injury, as evidenced by a 51% reduction in infarct size compared to wild-type mice. B-017 treatment provided no additional protection (n = 4 C57Bl/6J mice, n = 6 $Bnip3^{-/-}$ mice, n = $Bnip3^{-/-}$ mice treated with B-017, one-way ANOVA).*

5. Extended Fig. 20 mitochondrial apoptosis pathway analysis:
In Extended Data Fig. 20b, the use of ANT1 as a mitochondrial loading control is inappropriate due to its variable expression under stress like ischemia. It is recommended to use more stable loading control like VDAC1 or COX IV. In Extended Data Fig. 20c, the release of Cyto C from mitochondria into the cytosol is a core event in apoptosis. It is recommended to simultaneously present data for Cyto C levels in both the cytosolic and mitochondrial fractions. In addition, variability in loading control bands across both panels undermines result reliability.

We agree with the Reviewer that ANT1 shows variable expression under high-stress conditions. However, in the specific context of acute myocardial I/R with short ischaemia and reperfusion times, total ANT1 abundance has been found to remain stable (Dörner et al., 2021, Membranes). This aligns with our observation of only minimal variance that appears attributable to loading rather than biological regulation. The Western blot images depicting ANT1 as a loading control and included in the formerly Extended Data Fig. 20b were incorrectly cropped and not used for analysis, and were therefore presented in error. We apologise for this oversight. For the analysis, we used images that had been exposed for a shorter time. The correct images have now been included in the Source Data file. While we agree with the Reviewer that the loading control bands exhibit a certain degree of variability, we are certain that this is offset by normalisation and does not influence the results achieved. Accordingly, we would kindly request to keep ANT1 as loading control in this particular setting.

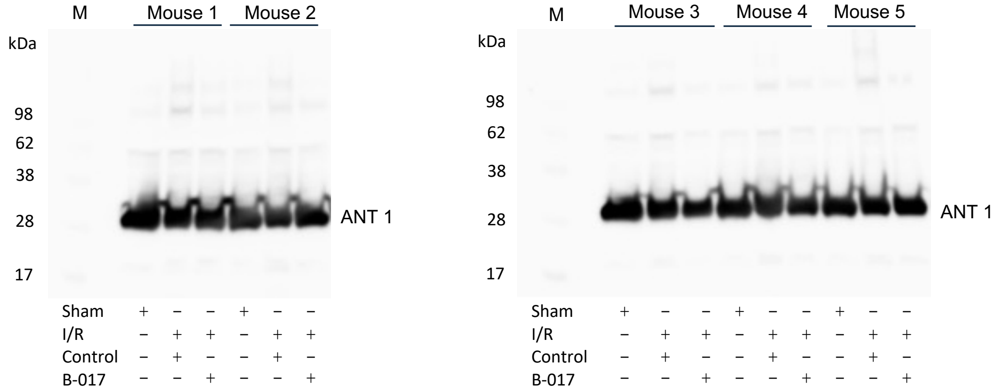

*Following the Reviewer's recommendation, we assessed cytochrome c levels in the mitochondrial fractions of 3 mice per group. These mice were subjected to myocardial I/R (lanes 3-5) or to I/R with 30 min of ischaemia followed by 30 min of reperfusion and treated intracardially with either B-017 (lanes 7-9) or the negative control peptide (lanes 11-13). The mitochondrial fractions analysed correspond to the cytosolic fractions shown in formerly Extended Data Fig. 20c (mice 1-3). Western blot analysis revealed comparable levels of mitochondrial cytochrome c in hearts treated with B-017 compared to sham-operated controls, with a tendency towards higher levels in B-017-treated hearts compared to those treated with the negative control peptide. These results further support the protective effect of B-017 in limiting cytochrome c release, as evidenced by the reduced cytosolic cytochrome c levels observed in the corresponding samples. We have added the results and the methods to the Results (**line 533**) and Methods section (**lines 1299-1300**) in the revised manuscript. The data are now shown in **new Supplementary Fig. 23c**.*

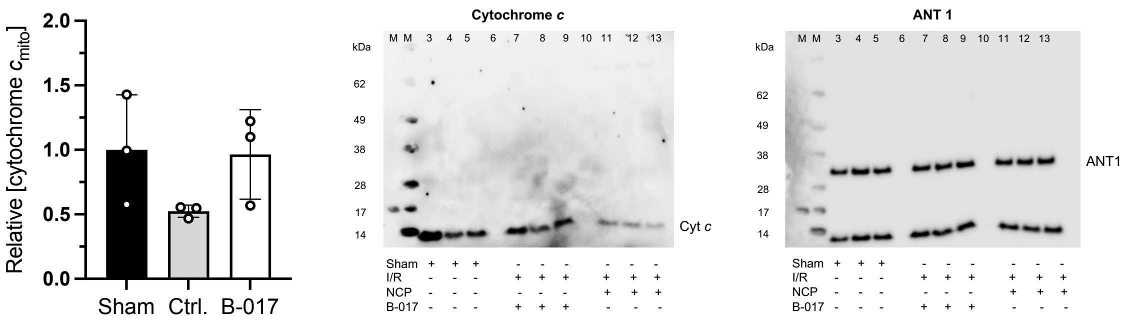

New Supplementary Fig. 23c: *Immunoblot analysis of mitochondrial cytochrome c levels in the AAR of mouse hearts after 30 min of reperfusion showed a tendency towards higher levels under B-017 treatment compared to those treated with the negative control peptide (NCP, Ctrl.).*

6. Necrosis pathway indicators: The manuscript states that "B-017 inhibits both necrosis and apoptosis". While apoptosis pathways are assessed via Bax, caspase-3/7, and Cyto c, current data lack specific indicators validating necrosis pathway.

*We have probably not sufficiently elaborated on this point. Mitochondrial swelling, resulting from the opening of the mitochondrial permeability transition pore (mPTP), precipitates rupture of the mitochondrial membranes, collapse of bioenergetic homeostasis, and subsequent loss of plasma-membrane integrity, thereby committing the cell to necrotic death. Cardiac troponin I is a highly sensitive and necrosis-specific biomarker of cardiomyocyte injury, released only upon irreversible disruption of the sarcolemmal membrane. As shown in Fig. 6, B-017 treatment significantly reduced these specific indicators of necrosis pathway (mitochondrial swelling, Fig. 6a, and plasma troponin I level, Fig. 6b). We have now described this more clearly in the Results section of the revised manuscript **(lines 528-529)**.*

7. Potential off-target effects and toxicity: To better address specificity, could the authors discuss or provide data on whether B-017 interacts with other key BCL-2 family proteins (e.g., anti-apoptotic members like BCL-2)? Furthermore, for the long-term studies, data on animal body weight and histological analysis (HE staining) of major organs (heart, liver, kidney) could be provided for preliminary safety evaluation.

*We thank the Reviewer for these important points. To further address specificity, we performed off-target analyses using microscale thermophoresis and fluorescence anisotropy. First, we titrated fluorescence-labelled B-017 with increasing concentrations of the anti-apoptotic BCL-2 family members human (h) MCL-1 and hBCL-2, which inhibit the oligomerisation required for the formation of the MOMP by activated and already inserted BAX. With K_D values of approximately 1.3 µM for hMCL-1 and hBCL-2, B-017 bound these proteins with a 4-fold lower affinity than it bound hBAX (K_D = 303 nM). This residual binding to BCL-2 and MCL-1 is likely attributable to the general propensity of peptides to interact with proteins that share relatively high structural similarity, which is the case for members within the BCL-2 protein family (London et al., 2010, Structure). For example, BCL-2 and BAX display a root mean square deviation (RMSD) of approximately 1.9 Å to 2.2 Å (Petros et al., 2001, PNAS), indicating that they have similar structures. On the other hand and in support of this interpretation, no binding of B-017 was detected to the structurally unrelated protein glutathione-S-transferase. Together, this points to an unlikely occurrence of side effects due to off-target responses. We have added these new results and the respective methods to the Results (**lines 387-401**) and Methods section (**lines 853-854, 866-881**), and a text to the Abstract (**lines 56, 57, 59**). The data are now shown in **new Supplementary Fig. 18** of the revised manuscript. Since all MST and fluorescence anisotropy measurements were performed with tagged recombinant proteins, except the anisotropic evaluation of B-017 binding to BAX, we have replaced Fig 2f with the results achieved with tagged BAX for unification*

*(**new Figure 2f, right panel**). The comparability of the two methods used is demonstrated by the identical K_D values obtained for BCL-2 (**new Supplementary Fig. 18b**).*

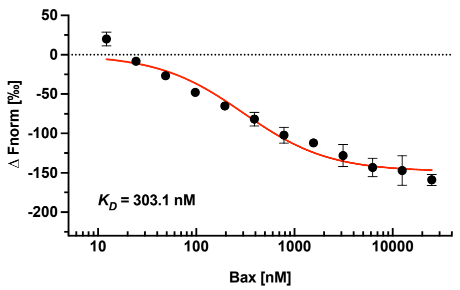

***New Fig. 2f:** Microscale thermophoresis dose-response curve of the binding of the protein BAX to Cy.5.5-labelled peptide. A constant concentration of 200 nM fluorescence-labeled peptide was incubated with serial dilutions of BAX (12.2–25,000 nM). Normalised fluorescence changes were plotted as a function of protein concentration. To determine binding affinities (K_D), nonlinear regression analysis was performed using a one-site binding model. Data points represent the mean ± SD from three biological replicates (n = 3), and solid red lines show the fitted binding curves.*

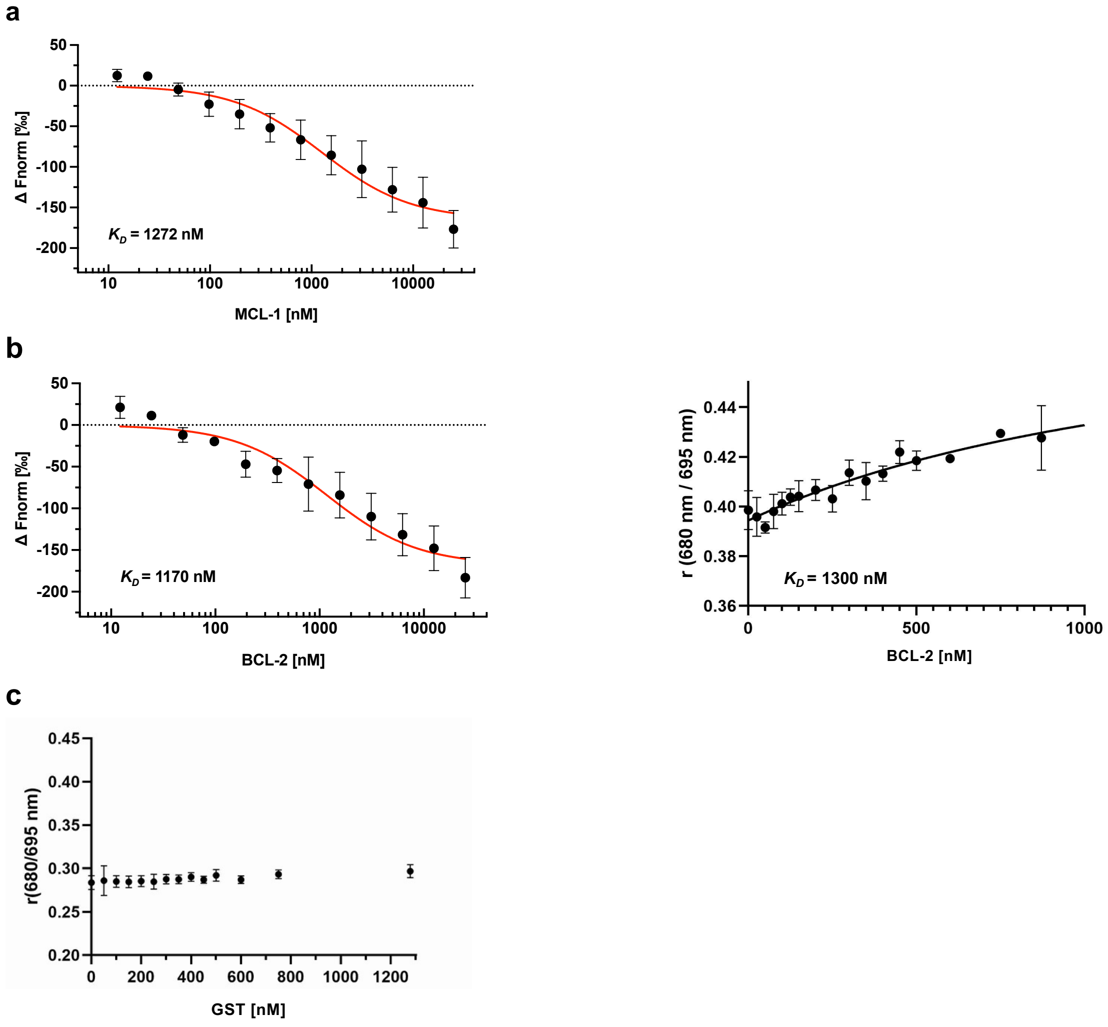

New Supplementary Fig. 18. Dose-response curves of the binding of the proteins MCL-1, BCL-2, and glutathione-S-transferase to Cy5.5-labelled peptide.
*Microscale thermophoresis dose-response curves for the binding of Cy5.5-labelled B-017 to the human proteins (**a**) MCL-1 and (**b, left**) BCL-2. A constant concentration of 200 nM fluorescence-labelled B-017 was incubated with serial dilutions of each protein (12.2–25,000 nM). Normalised fluorescence changes were plotted as a function of protein concentration. To determine binding affinities (K_D), nonlinear regression analysis was*

performed using a one-site binding model. Data points represent the mean ± SD from three biological replicates (n = 3), and solid red lines show the fitted binding curves. ***b, c,*** *Fluorescence anisotropy measurement (680/695 nm) with titration of increasing human BCL-2 (**b, right**) or glutathione-S-transferase (GST) concentrations (**c**) to 100 nM Cy5.5-labelled B-017. All titrations were performed in n = 3 technical replicates and data points are shown as means ± SD based on the three experiments (n = 3). Binding curves of the averaged data were fitted with GraphPad Prism 10.0 (GraphPad) using the quadratic binding equation for a one-site specific binding model and K_D values are given as fit of the averaged data points ± standard deviation of the fit.*

To address the safety of B-017 as a key criterion of a therapeutical agent, we recorded animal body weights and performed toxicity analysis including histological observation of tissue and organs after a 2-week intravenous injection of B-017 at dosages of 3, 6, and 12 mg kg^{-1} day^{-1} followed by a two-week recovery period in female and male rats. The study was performed by WuXi AppTec Co., Ltd, and was conducted in compliance with Good Laboratory Practice standards specified by ICH guidelines.

*Overall, the administration of B-017 to female and male Wistar Han rats once daily for 2 weeks by intravenous injection at all three dosages was well tolerated. The rats showed no body weight loss during the dosing or recovery phases of the B-017 treatment. B-017-related change in body weight was limited to lower body weight gain in male rats at a dosage of 12 mg/kg/day during the dosing phase, compared to the concurrent control group. There were significant differences in body weight gain from day 4 to 14 (day 4 to day 7: -48.4%; days 11 to 14: -48.9%). This resulted in a slightly reduced mean body weight in male rats. However, at the end of the recovery phase, the above changes balanced out and all body weight values in males at a dose of 12 mg kg^{-1} day^{-1} were comparable to the concurrent control data. Other fluctuations in body weight gain were consistent with those observed in untreated rats. The data are now shown in **new Supplementary Fig. 19**.*

*In addition, no B-017-related changes were detected in serum-chemistry and urine analysis parameters, and no visible lesions were observed in examined various tissues and organs, including the heart, liver, and brain were observed in either sex (**Supplementary Tables 5-10**). During the dosing phase, microscopic changes were identified as subcutaneous haemorrhage, subcutaneous inflammation of mixed cells, and/or cutaneous/subcutaneous/muscular necrosis in the injection site in rats. Minimal or mild subcutaneous haemorrhage was noted in perivascular areas of 5 high-dose animals (2 males and 3 females), one mid-dose male, and none in the low-dose and control groups, an indication of B-017-related injection site change. Minimal to marked subcutaneous inflammation of mixed cell with or without necrosis was identified in the cutaneous, subcutaneous, adjacent muscular fibers, and/or surround the vein. The incidence and severity were dose dependent. In addition, the necrosis and/or necrotizing inflammation in the injection site were exclusively observed in animals of the high-dose groups. Compared with the incidence in the control group, the increased incidence and severity of lesions in the injection site were likely B-017 related. At the end of a 2-week recovery period, B-017-related changes were only present in the injection site of one single high-dose animal. This rat had minimal subcutaneous haemorrhage and moderate necrotizing inflammation of mixed cells in the cutaneous, subcutaneous and adjacent muscle. No lesions in the injection site were noted in the high-dose females, an indication of a full recovery in female rats. The data are now shown in **new Supplementary Tables 9 and 10**.*

*B-017-related changes in haematology were limited to male rats at 12 mg kg^{-1} day^{-1} at the end of the dosing phase with increased WBC (77.70%, $p \leq 0.001$), neutrophils count (208.67%, $p \leq 0.01$) and neutrophils percentage (65.5%) compared with concurrent control data. These haematological changes were correlated to microscopic changes of subcutaneous haemorrhage, subcutaneous inflammation of mixed cells, and/or cutaneous/subcutaneous/muscular necrosis in the injection site. However, all haematological changes had resolved at the end of recovery phase. The data are now shown in **new Supplementary Tables 11 and 12**.*

Regarding the toxicokinetics of B-017, initial and maximum plasma concentration (C_0, C_{max}), time to reach C_{max} (T_{max}), $T_{1/2}$ and area under the plasma concentration-time curve from time zero to 24h-hour postdose ($AUC_{0\text{-}24h}$) were calculated. After single (Day 1) or repeated (Day 14) intravenously injection of B-017 to male and female rats, T_{max} values for B-017 were observed at 0.1 h postdose. $T_{1/2}$ values for

B-017 were ranged from 0.3 to 0.6 hour. No marked sex difference in systemic exposure (AUC_{0-24h} and C_0) to B-017 was observed at all dose levels. As the dosage increased from 3 to 12 mg/kg/day, the systemic exposure (AUC_{0-24h} and/or C_0) to B-017 increased dose-proportionally in both sexes on Day 1 and Day 14. The data are now shown ***in new Supplementary Table 13****.*

In conclusion, the No Observed Adverse Effect Level (NOAEL) for rats is 12 mg kg^{-1} day^{-1}. The corresponding AUC_{0-24h} and C_0 of B-017 on Day 14 at NOAEL were 4,210 h ng mL^{-1} and 9,890 ng ml^{-1} for males, and 4,950 h ng mL^{-1} and 10,700 ng mL^{-1} for females, respectively.

*We have amended the results as follows (****lines 402-418****):*

"B-017 did not cause toxicity as initially inferred by monitoring the body weight of female and male rats administered 0, 3, 6 and 12 mg kg^{-1} body weight for 14 days, followed by a 14-day recovery phase. The rats showed no body weight loss during the dosing or recovery phases of the B-017 treatment (Supplementary Fig. 19a). In addition, no B-017-related changes were detected in serum-chemistry and urine analysis parameters, and no visible lesions were observed in examined various tissues and organs, including the heart, liver, or brain, in either sex (Supplementary Tables 5-10). During the dosing phase, treatment-related transient microscopic findings were confined to the injection site and included subcutaneous haemorrhage mixed-cells inflammation, and necrosis of the skin, subcutaneous tissue and underlying muscle in both female and male rats (Supplementary Tables 9 and 10). These local findings were accompanied by haematological changes, but these remained transient (Supplementary Tables 11 and 12). All these observations had fully resolved by the end of the recovery phase. Systemic exposure to B-017 increased proportionally with dose, with no sex-related differences observed and a terminal half-life ranging from 0.3 to 0.6 h (Supplementary Table 13). Taken together, the demonstrated appreciable target specificity and favourable safety profile support the suitability of B-017 as a potential therapeutic agent."

*The results and the methods have been added to the Results (****lines 402-418****) and Methods section (****lines 658-668, 1218-1249****) and text to the Abstract (****lines 56, 57, 59****) and Discussion sections (****line 607****) in the revised manuscript.*

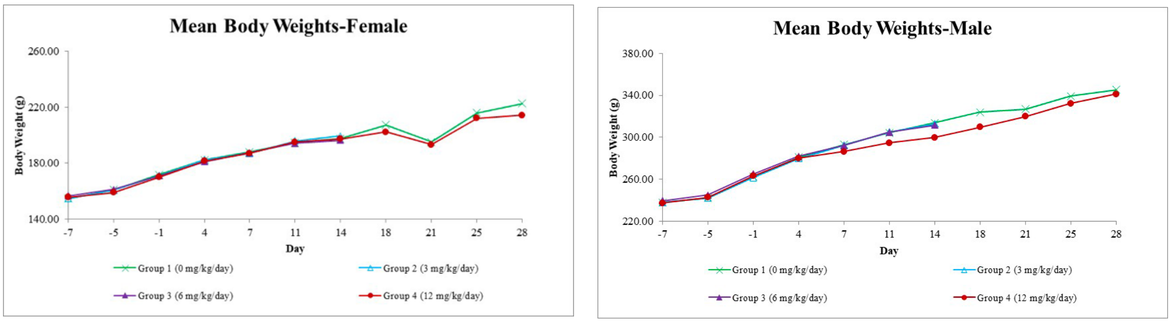

Supplementary Fig. 19. Impact of B-017 on body weight in female and male rats. *The animals received four different doses of B-017 (0, 3, 6 or 12 mg kg^{-1} body weight) for 14 days, followed by a 14-day recovery phase. Neither sex showed any loss of body weight during the dosing or recovery phases of the B-017 treatment.*

8. In vivo distribution of the peptide: Current data only show macroscopic cardiac distribution (Extended Data Fig. 17a). It is recommended to provide distribution data for B-017 in other key target organs, particularly the brain and liver. This could include measurements of tissue concentration or visualization of its distribution at the cellular level within tissues using immunofluorescence.

*We thank the Reviewer for this recommendation. In mouse myocardial I/R, we examined the uptake of fluorescence-labelled B-017 (1.6 mg kg^{-1} body weight) administered via intracardiac injection 5 min before reperfusion in various organs 5 min after reperfusion using the BMG FLUOstar Omega Microplate Reader. The signal distribution in the organs examined was found to be as follows: B-017 – 70.1% in the liver, 11.5% in the spleen, 10.2% in the plasma, and 8.2% in the heart. In mouse brain I/R, fluorescence-labelled B-017 (1.6 mg kg^{-1} body weight) were administered intravenously at the onset of ischaemia. The uptake was examined after 30 min of ischaemia. The micrographs showed uptake of the peptide. We have added the results and the method to the Results (**line 442**) and Methods sections (**lines 1346-1356**). The data are now shown in **new Supplementary Fig. 20b** of the revised manuscript.*

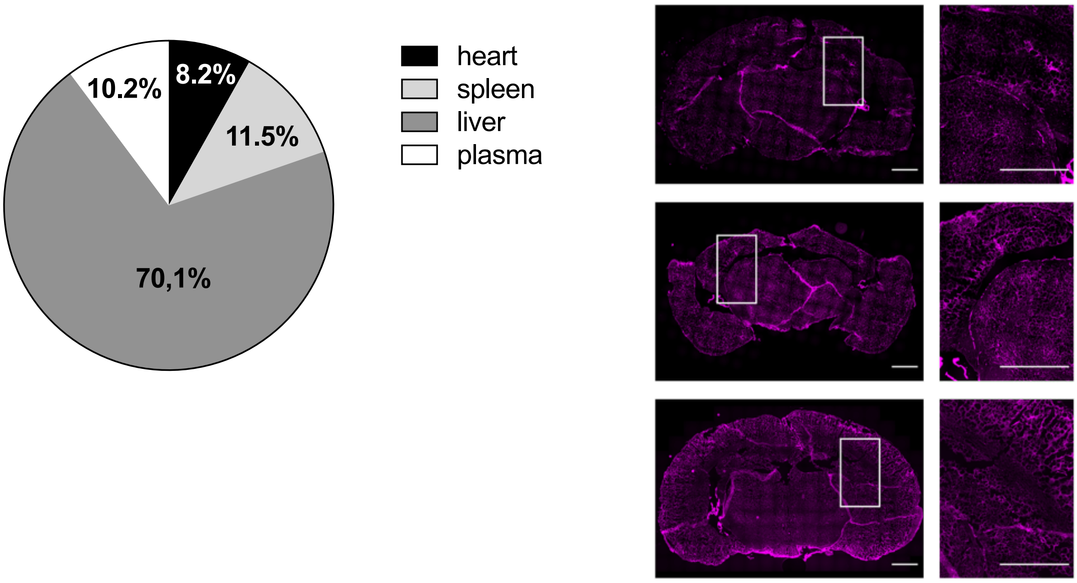

New Supplementary Fig. 20b: *Uptake of Lys(5(6)-FAM-labelled B-017 in different mouse organs at 5 min reperfusion following 30 min of myocardial ischaemia. The peptide was administered into the LV cavity 5 min before reperfusion (**left**) (n = 3 mice). Representative micrographs showing uptake of Lys(5(6)-FAM-labelled B-017 in the brain at 30 min ischaemia. The peptide was administered intravenously at the onset of ischaemia (**right**) (n = 3 mice). (Scale bars 1,000 µm).*

Reviewer #2 (Remarks to the Author):

Comments for Authors

Hendgen-Cotta et al. performed a wide range of experiments to design an inhibitor of BAX/BNIP3 to suppress death signaling in vitro and in vivo, with the goal of attenuating I/R injury. They employed a variety of cellular and animal models to demonstrate the efficacy of their therapeutic peptide, B-017. The notion of reverse engineering is certainly interesting. The authors also made a great effort explaining their logic, as well as the details of the experiments. The potential application areas, including myocardial I/R, cerebral I/R, and liver transplantation, of BNIP3/BAX inhibition are also of clinical interest. However, the current study, as presented, suffers from insufficient data to support their conclusions, as well as concerns regarding the proposed mechanism of action of B-017. Detailed comments are listed below.

We thank Reviewer 2 for all suggestions. They greatly helped us to improve the quality of our work.

Fig. 1a: BNIP3-BAX/BAK PLA experiment in Bak-/- and Bax-/- MEFs. First of all, validation of the absence of the two proteins in MEFs is lacking. Then, the authors discussed the lack of BNIP3-BAX signal in Bax-/- MEFs as a dependence of BAK1 orientation on BAX (a reference should be cited). While this is certainly possible, the authors did not rule out the possibility that BAX deletion impacts BAK1 expression, and vice versa. A simple experiment would be to use Western blotting to detect the BAX and BAK1 expression in Bak-/- and Bax-/- MEFs, respectively. In addition, the authors did not discuss the reduced BNIP3-BAK PLA signal in Bak-/- MEFs.

We have used Western blotting to analyse BAX and BAK1 expression in $Bak^{-/-}$ and $Bax^{-/-}$ MEFs. The results confirmed the expression of BAX in $Bak^{-/-}$ MEFs and of BAK1 in $BAX^{-/-}$ MEFs. We have included the Western blot images in ***new Supplementary Fig. 1b.*** *We have also included the method in the Methods section (**lines 685-691**). Regarding our suggestion that the orientation of BAK1 may depend on the presence of BAX, we have clarified the relevant sentence: "One potential explanation could be that the orientation of BAK1, which is required for a close proximity to BNIP3, may depend on the presence of BAX." (**lines 126-128**). Regarding the reduced BNIP3-BAK PLA signal in $Bax^{-/-}$ MEFs, we amended the relevant sentence: "The observed localisation of BNIP3 and BAX was also evident, albeit to a slightly lesser extent, in MEFs lacking BAK1 (Fig. 1a). This may be due to the absence of BAK1." (**lines 123-125**).*

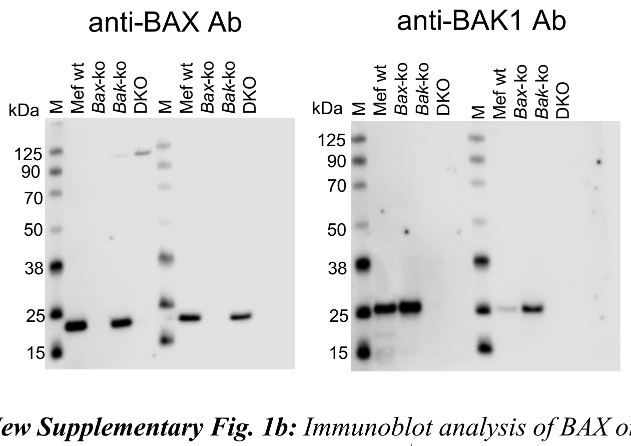

New Supplementary Fig. 1b: *Immunoblot analysis of BAX or BAK expression in wild-type (wt) mouse embryonic fibroblasts (MEFs), Bax knockout ($Bax^{-/-}$) MEFs, Bak knockout ($Bak^{-/-}$) MEFs and $Bax^{-/-}$/$Bak^{-/-}$ MEFs (DKO using the anti-BAX antibody (1:1,000, C2772, Cell Signaling; anti-rabbit HRP, 1:20.000) and the anti-BAK1 antibody (1:1,000, 12105, Cell Signaling). (n = 2 independent experiments).*

The use of circular dichroism spectroscopy is not explained, which is not a standard tool to determine protein tertiary structure. This method evaluates chirality, which is distinct from protein structure. The authors state that "recombinant hBNIP3 exhibited a comparable tertiary structure to recombinant hBAX as determined by CD spectroscopy (Extended Data Fig. 7a)" (Lines 158-160). However, their information was not shown side by side, and could not be compared. Also, is CD sufficient to tell tertiary structure? From the data shown in Extended Data Fig. 7b, it is only a statistical summary of the secondary structures within a protein.

*We thank the Reviewer for pointing out this typo, which we have corrected in the mentioned sentence (**line 164**). We only discuss the secondary structure composition based on the far-UV CD spectrum, not the tertiary structure. To facilitate understanding for the reader, we added " [...], yielding the percentages of a-helices, ß-sheets and random coil." to the Methods section (**lines 831-832**).*

*We added the overlay of the CD spectra of BAX and BNIP3 in the **new Supplementary Fig. 7b** to allow for side-by-side comparison as requested.*

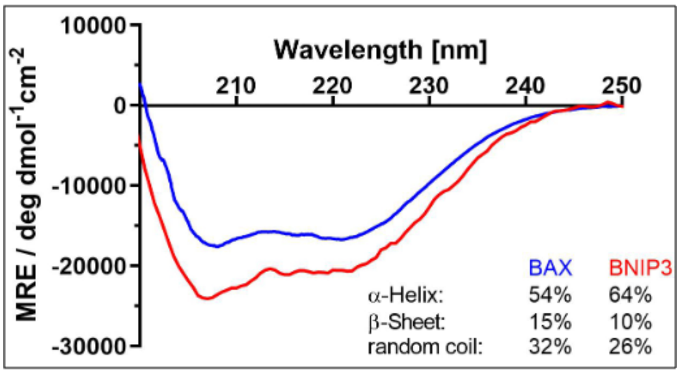

***New Supplementary Fig. 7b:** Overlay of the CD spectra of BAX and BNIP3.*

Fig. 2b: The in vitro BAX activity assay, as explained in the Methods, is essentially immunoprecipitation using an anti-BAX (6A7) antibody. If Lane 6 (left blot) is input, then it should be labeled as such. Why are there so many bands for BAX? The IgG is applied to the wrong group. It should be used instead of 6A7, where peptides/proteins are added to activate BAX. A negative control is also lacking (a protein/peptide that is known to not activate BAX). Lanes 3 and 4 are exactly the same. In Lane 2, it is not clear why IgG is added in addition to an anti-BAX (6A7) antibody. The right blot also lacks a negative control, such that we cannot rule out that it is inherently activated.

We apologize for the typographical errors in the description of the Western blot image. We have corrected these and labelled Lane 6 as 'input'. IgG was only applied to lane 5. Lanes 3 and 4 represent different concentrations of BNIP3-Peptide$_{1\text{-}49}$ (lane 3: 5µM, lane 4: 50µM). The bands in the Western blot image represent monomeric, dimeric and oligomeric species of BAX arising from SDS-Page and immunoblotting conditions (no heating of sample and use of Tween).
*To rule out the inherent activation of BAX, we performed additional in vitro BAX activity assays using bovine serum albumin and BCL$_{XL}$, a member of the antiapoptotic BCL-2 family, which is known not to activate BAX. Immunoprecipitation using the anti-BAX antibody 6A7 revealed no BAX activation. Additionally, no BAX insertion in the mitochondrial outer membrane was observed. These results and methods have been included in the Results (**lines 221-222, 226**) and Methods section (**line 1024**) of the revised manuscript. The data are now shown in **new Supplementary Fig. 10d, e**.*

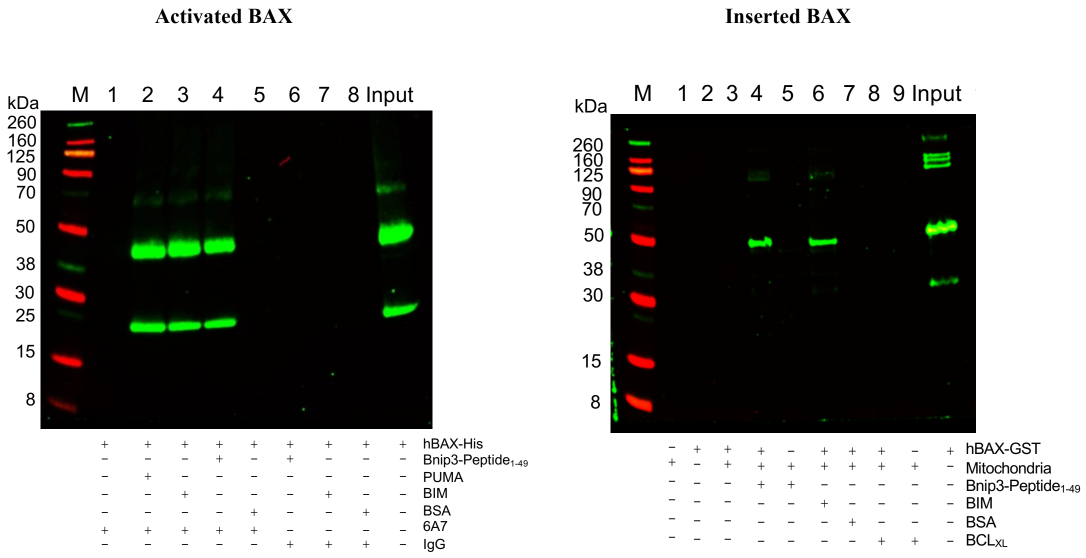

New Supplementary Fig. 10d and e: *Impact of bovine serum albumin (BSA, 5 µM) and the antiapoptotic protein BCL_{XL} (500 nM) on the 1st and 2nd activation steps of BAX. Staurosporine (STS), BNIP3-$Peptide_{1-49}$ ($Peptide_{1-49}$), PUMA and BIM were used as stimulation agents. Immunoblot analyses of activated* ***(d)*** *and inserted BAX* ***(e)*** *showed no activation of BAX upon treatment with BSA and BCL_{XL}.*

The main logical flaw of this study is the rationale for designing a BNIP3 antagonist starting with a truncated BNIP3 peptide. The BNIP3 peptide 1-49 was shown to interact with BAX in Fig. 2a and 2b. Then, the authors started to use screen binding of mBNIP3 with mBNIP3 N-terminal peptides (starting Line 288), which has no basis. Why is BNIP3 supposed to interact with BNIP3 in an N-terminal fashion? Preceding data focused on showing BNIP3 interaction with BAX. In addition, the authors stated that "BNIP3 is known to form a stable homodimer via the coiled-coil formation of its C-terminal helix" (Line 189), and not the N-terminus.
In addition, given that the N-terminus of BNIP3 was shown to activate BAX, it would be logical to devise an inhibitor to this protein-protein interaction (either something that covers the 1-49 part of BNIP3 or binding to the corresponding BAX hotsopt without activating it), and not determining the sequence of BNIP3 amino acids that binds to BNIP3, as was done in this study. Furthermore, peptide 1-49 was shown to activate BAX (Fig. 2b). What is the rationale for using it as a starting material to design an antagonist, which has the opposite function? For the determination of the antagonist in Fig.2, BAX activity measurements were not shown. (did not appear until Fig.3b). Then, this part of the results section cannot use a title containing 'antagonist'.
It is also not clear why the peptide also derived from a BAX-activating peptide, and then matched the inactive form (Line 307). Does it bind active and inactive BAX equally? Or this there a binding preference?

We understand how our current structure and wording may have contributed to the confusion, and we have revised the text to improve clarity. Historically, we set out to identify an inhibitor for BNIP3, so we investigated the BNIP3 N-terminus. This region is known to stabilise the BNIP3 dimer, in addition to the interaction via its C-terminal transmembrane domain. It is also important for BNIP3 function (Kubli et al.,2008, Am J Physiol. Heart Circ Physiol). Furthermore, a hydrophobic stretch with a high propensity for protein-protein interactions is located within the first 49 BNIP3 residues. Such bi- or multipartite interaction interfaces exist in some proteins (e.g. Survivin), and their parts can work synergistically, function independently, or compete with each other. At the same time, we found that full-length BNIP3 and BAX interact in vitro and in cultured cells under unstimulated conditions. In silico analyses revealed two interfaces, one of which was the N-terminus. We then wondered whether the full BNIP3 (1-49) N-terminus would represent a functional domain in BNIP3 and would be sufficient to activate BAX, which we demonstrated using the BNIP3-$peptide_{1-49}$.

After narrowing down the peptide sequence that interacts with BNIP3, we then discovered that the peptide also interacts with BAX and is able to potently block the BAX:BNIP3 activating interaction and its resulting downstream effects, resulting in effective protection of the heart.
*Fig. 2e shows that the mutated B-017 sequence with F (WVELHF**F**N; turquoise bar) instead of S gives a greater response, equaling a higher fraction bound and thus binding with higher affinity to BAX than the BNIP3 wt sequence (WVELHF**S**N; medium grey bar).*
*The concept that a reduced or altered binding motif can compete with its natural counterpart is not new; for enzymes, it is common to use substrate analogs as inhibitors. Those analogs bind tightly to the active site, but aren't turned over, thus competing with the native substrate binding, which inhibits substrate turnover. Our design and structural logic follows the same principle: using a modified, tightly binding version of the native interacting sequence that binds but cannot activate BAX, which consequently leads to inhibition instead of activation, as it competes with the native activator. We have incorporated a respective phrase in the Results section (**lines 239-241**).*
*With regard to the comment that the part of the results section relating to the development of a peptide from the identified activation domain should not the term 'antagonist' in its title, we agree with the Reviewer and have deleted this term (**line 202**).*

In Line 327 and 333, the authors mentioned the binding of B-017 to BAX/BNIP3 to form a ternary complex, which was not mentioned earlier, and the data do not support their conclusions. First of all, there is no computational model nor experimental validation of the BAX/BNIP3/B-017 complex.
Secondly, if such a ternary complex existed, how does it work? The authors showed that B-017 binds both BNIP3 and BAX (Line 320). But if BNIP3 binds to BAX using its N-terminus, then how can a N-term-derived BNIP3 peptide bind to them? This is not logically possible.
Furthermore, If BNIP3 does not need stimulus to bind BAX (Fig. 1b), then is BAX constitutively active? Does B-017 compete with BNIP3 for BAX binding? In Fig. 3d, it seems that B-017 competes with BNIP3. Is its affinity higher? All of these questions are not clarified.

*We thank the Reviewer for these insightful questions and apologize for any unclear wording or incomplete explanations in the current version. Indeed, the binding of B-017 to BAX/BNIP3 do not yield inevitably a ternary complex. However, under unstimulated conditions, BNIP3 and BAX physically interact (Fig.1 and Supplementary Figs. 1c, 4a-e, 5a-d), with BAX remaining in its inactive state, as evidenced by immunoprecipitation using the anti-BAX antibody 6A7 incubated with recombinant GST-BAX and recombinant BNIP3, followed by Western blotting using the anti-BAX antibody 2D2, which demonstrated that BAX is not activated (**new Supplementary Fig. 5e**). In silico analyses revealed two interfaces, one of which was the N-terminus. Due to its higher affinity, B-017 displaces the N-terminus of BNIP3 by binding to BAX. This is caused by hotspot mimicry of key hydrophobic residues in the native sequence, as well as the substitution of serine for phenylalanine, making B-017 more hydrophobic than the wild-type sequence. This is evidenced by the inhibitory effect of B-017 in vitro, when it is administered simultaneously with the BAX-activating N-terminus of BNIP3, i.e. BNIP3-peptide$_{1-49}$ (Fig. 3). In addition, Fig. 2e shows that the mutated B-017 sequence with phenylalanine (F; WVELHF**F**N; turquoise bar) instead of serine (S) produces a greater response, equaling a higher fraction bound. Thus, it binds with higher affinity to BAX than the BNIP3 wild-type sequence (WVELHF**S**N; medium grey bar). We have incorporated a respective phrase in the Discussion section (**lines 588-589**).*
*B-017 exerts its protective effect by preventing the initial activation step of BAX. This is supported by the fact that B-017 only provides protection when administered before or concomitantly with the stimulus, whereas delayed administration (e.g. 10 min after reperfusion onset) fails to reduce infarct size (Reviewer-only Fig. 1). This temporal requirement indicates that B-017 must bind to BAX in its inactive conformation to exert its protective ability, which is consistent with the presence of inactive BAX under baseline conditions. The result on the inactive state of BAX under unstimulated conditions has been included in the Results section (**lines 149-150**) of the revised manuscript. The data are now shown in **new Supplementary Fig. 5e**. We have also incorporated a phrase in the Discussion section (**line 588**) and a schematic diagram (**new Supplementary Fig. 25a**) to address this point.*

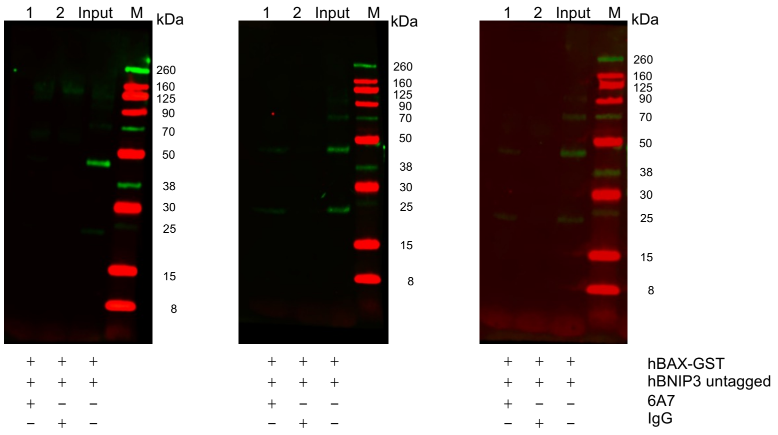

New Supplementary Fig. 5e. *Western blot analysis of the activation status of BAX within the BNIP3/BAX complex. Recombinant untagged human BNIP3 (hBNIP3) and human BAX-GST (hBAX-GST) were incubated simultaneously with 6A7 antibody and IgG control. Western blot analysis following SDS-PAGE using the anti-BAX antibody 2D2 showed that BAX is in its inactive state within the BNIP3/BAX complex.*

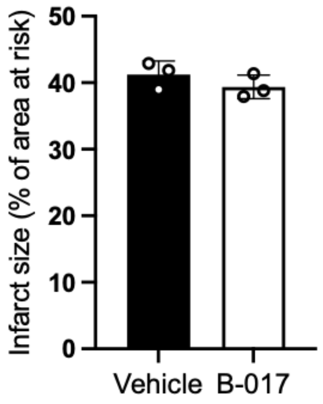

Reviewer-only Fig. 1. *Therapeutic response to B-017 in C57Bl/6J mice during reperfusion. Mice were exposed to 30 min of ischaemia, followed by 24 h of reperfusion. B-017 was administered intracardially 10 min after reperfusion commenced. Infarct size measurement (infarcted tissue per AAR) showed that B-017 exhibited no efficacy when administered after the reperfusion-induced heart injury. (n = 3 C57Bl/6J mice).*

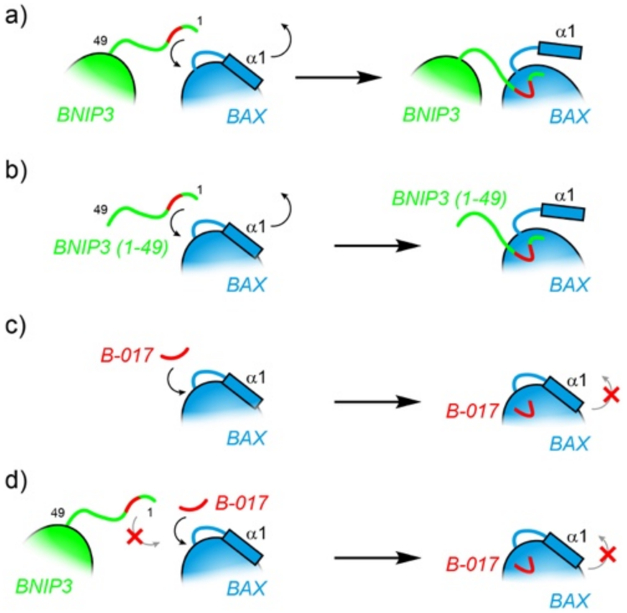

New Supplementary Fig. 25a. *Schematic diagram showing the proposed mechanism of B-017 illustrating* ***(a)*** *the functional domain of full-length BNIP3 activating BAX leading to exposure of helix α1,* ***(b)*** *binding of the functional domain of BNIP3 as BNIP3-peptide$_{1-49}$ activating BAX leading to exposure of helix α1* ***(c)*** *binding of B-017 to BAX with no activation of BAX, and* ***(d)*** *competition of B-017 with the BNIP3 activating domain, preventing the activation of BAX.*

Fig. 3b. Staurosporine (STS) is used to stimulate cell death. But this experiment was an in vitro (cell-free) assay. What is the rationale for using STS? Even if it were a cellular assay, why use STS if BNIP3-BAX interact at base level (Fig. 1b)? Does STS enhance BNIP3-BAx interaction (not shown in the data)?

The rationale for using STS was its activation of BAX in the cell-free assay as a positive control for experimental approaches involving BAX activators, such as BIM, PUMA, BH3-mimetics, and BNIP3-Peptide$_{1-49}$. These activators likely bind to different sites in BAX (Fig. 3b, c). In cellular assays and in vivo, where BNIP3/BAX interaction already exists, I/R injury, doxorubicin as well as staurosporine, cause an imbalance in the phosphorylation of proteins towards a dephosphorylated state. This kind of change in BNIP3 has been shown to switch its activity from mitophagy to apoptosis (Zhu et al., 2013, J Biol Chem; Liu & Frazier, 2015, PlosOne), indicating the mechanism through which BNIP3 activates BAX within the BNIP3/BAX complex.

Line 448 "It can be inferred that BAX may also be activated indirectly through the inhibition of BCL-2." This is not evident in the data shown. BCL2 did not appear in any preceding experiment.

*We apologise for not providing references for this statement and have added the relevant publication by Chen et al., 2015, Nat Cell Biol and Vitale et al., 2023, Cell Death Differ accordingly (****line 325****).*

Fig. 3d How does B-017 suppress apoptosis in the absences of Bax? Off-target effect? The authors briefly touched upon this point in line 458. But what are the implications? B-017 also lowered the signal in BAX/BAK double-KO cells, suggesting additional mechanisms. From the degree of the B-017 effect, it almost seems that it is a stronger inhibitor of BAK1 than of BAX (3d right, 3e left and middle). Do the authors have any comments regarding the specificity/selectivity of B-017?

*We agree that the effect of B-017 could also additionally be achieved via BAK1 as discussed in the Results section (**lines 332-342, formerly lines 455-465**). This could be due to the fact that BAK1 belongs to the BCL-2 protein family, whose proteins are structurally related, and peptides are able to bind to structurally related proteins. Therefore, and to address the similar comments of Reviewer #1 and Reviewer #3 response, we performed off-target analyses using microscale thermophoresis and fluorescence anisotropy. First, we titrated fluorescence-labelled B-017 with increasing concentrations of the anti-apoptotic BCL-2 family members human (h) MCL-1 and hBCL-2, which inhibit the oligomerisation required for the formation of the MOMP by activated and already inserted BAX. With K_D values of approximately 1.3 µM for hMCL-1 and hBCL-2, B-017 bound these proteins with a 4-fold lower affinity than it bound hBAX (K_D = 303 nM). This residual binding is likely attributable to the general propensity of peptides to interact with proteins that share relatively high structural similarity, which is the case for members within the BCL-2 protein family (London et al., 2010, Structure). For example, BCL-2 and BAX display a root mean square deviation (RMSD) of approximately 1.9 Å to 2.2 Å (Petros et al., 2001, PNAS), indicating that they have similar structures. On the other hand, and in support of this interpretation, no binding of B-017 was detected to the structurally unrelated protein glutathione-S-transferase. Together, this points to an unlikely occurrence of side effects due to off-target responses. We have added these new results and the respective methods to the Results (**lines 387-401**) and Methods sections (**lines 853-854, 866-881**), and text to the Abstract (**lines 56, 57, 59**). The data are now shown in **new Supplementary Fig. 18** of the revised manuscript. Since all MST and fluorescence anisotropy measurements were performed with tagged recombinant proteins, except the anisotropic evaluation of B-017 binding to BAX, we have replaced Fig 2f with the results achieved with tagged BAX for unification (**new Figure 2f, right panel**). The comparability of the two methods used is demonstrated by the identical K_D values obtained for BCL-2 (**new Supplementary Fig. 18b**).*

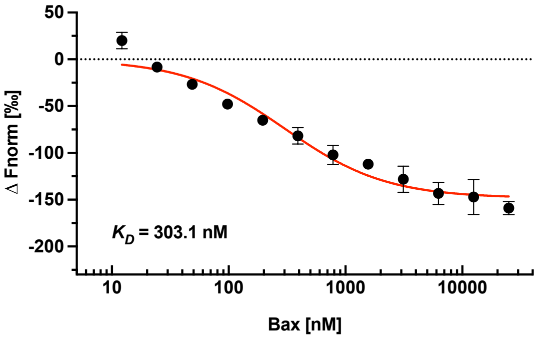

New Fig. 2f: *Microscale thermophoresis dose-response curve of the binding of the protein BAX to Cy.5.5-labelled peptide. A constant concentration of 200 nM fluorescence-labeled peptide was incubated with serial dilutions of BAX (12.2–25,000 nM). Normalised fluorescence changes were plotted as a function of protein concentration. To determine binding affinities (K_D), nonlinear regression analysis was performed using a one-site binding model. Data points represent the mean ± SD from three biological replicates (n = 3), and solid red lines show the fitted binding curves.*

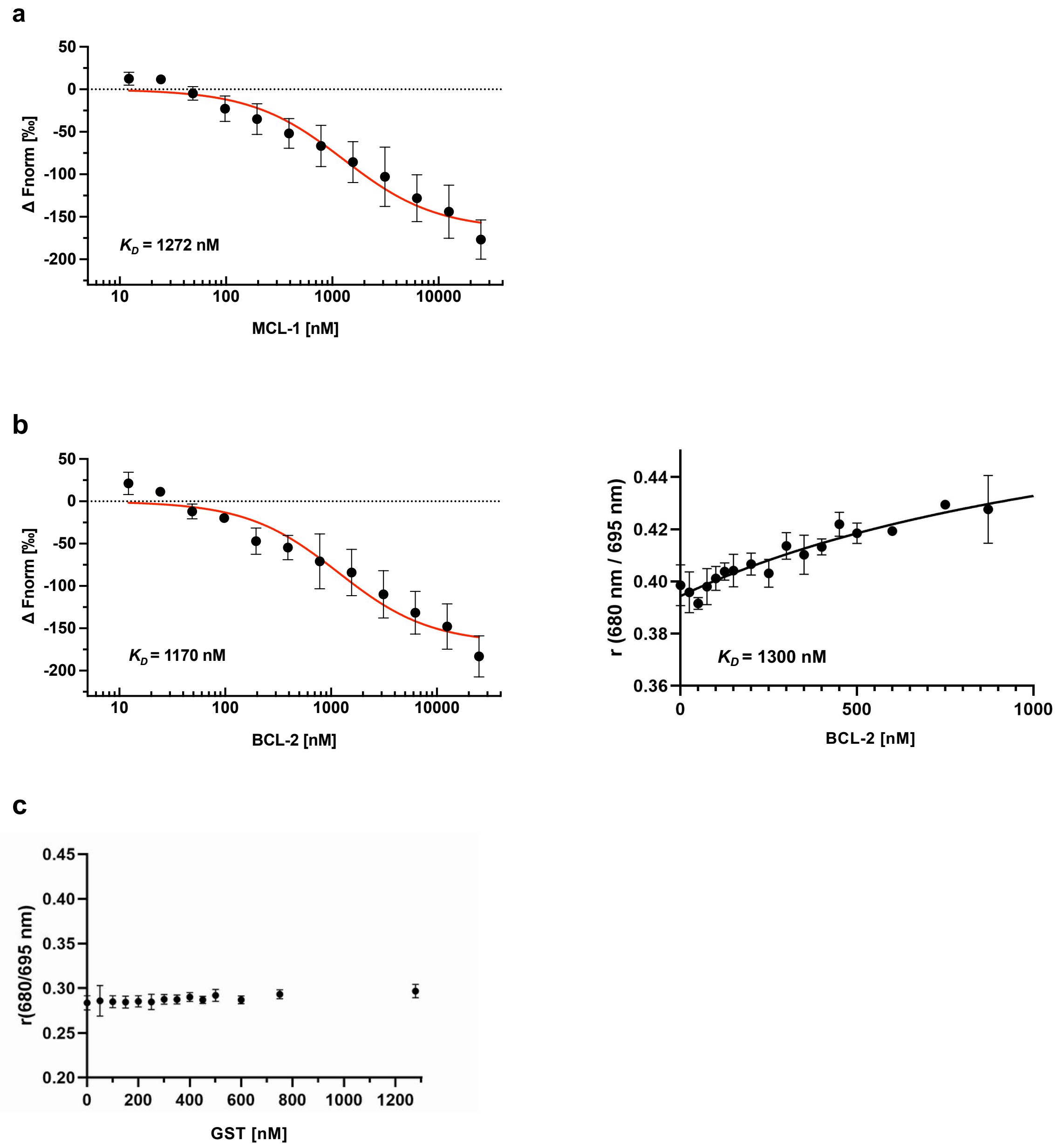

New Supplementary Fig. 18. Dose-response curves of the binding of the proteins MCL-1, BCL-2, and glutathione-S-transferase to Cy5.5-labelled peptide.
*Microscale thermophoresis dose-response curves for the binding of Cy5.5-labelled B-017 to the human proteins (**a**) MCL-1 and (**b, left**) BCL-2. A constant concentration of 200 nM fluorescence-labelled B-017 was incubated with serial dilutions of each protein (12.2–25,000 nM). Normalised fluorescence changes were plotted as a function of protein concentration. To determine binding affinities (K_D), nonlinear regression analysis was performed using a one-site binding model. Data points represent the mean ± SD from three biological replicates (n = 3), and solid red lines show the fitted binding curves. **b, c,** Fluorescence anisotropy measurement (680/695 nm) with titration of increasing human BCL-2 (**b, right**) or glutathione-S-transferase (GST) concentrations (**c**) to 100 nM Cy5.5-labelled B-017. All titrations were performed in n = 3 technical replicates and data points are shown as means ± SD based on the three experiments (n = 3). Binding curves of the averaged data were fitted with GraphPad Prism 10.0 (GraphPad) using the quadratic binding equation for a one-site specific binding model and K_D values are given as fit of the averaged data points ± standard deviation of the fit.*

On a sidenote, the legend for Fig. 3d right indicates n = 2 in knockout MEFs but the figure shows 3 data points. N=2 is clearly not sufficient for statistical analysis.

We apologise for the typo and have corrected the figure legend to n=3.

Extended data Fig. 14e why is the baseline lower in the BCL2-/- group compared to WT?

We agree that visually, the baseline in the BCL2$^{-/-}$ group appears smaller than in the WT group. However, this difference is not statistically significant and thus represent normal data point fluctuations.

Fig. 4. The use of cellular impedance as a measure/surrogate of cell viability/death is not warranted. In addition to cell attachment, impedance can also change with cell size, shape, etc. Common cell viability assays should provide additional information.

Although impedance reflects multiple biophysical properties, the primary factor in adherent cell cultures is the number of attached, viable cells. Cell death results in characteristic and reproducible decreases in impedance due to detachment, morphological collapse, and loss of membrane integrity. These relationships have been extensively validated against conventional viability assays (Ke et al., Methods Mol. Biol., 2011; 740, 33–43).

Line 638. The pig model is a good model for investigation. But why were very few data gained from this experiment? What happened to the pigs later? Any data?

Unfortunately, this experiment did not yield additional data, as the pigs were euthanized to allow assessment of infarct size. The TTC staining method used does not allow further processing of the heart tissue for use in other experiments.

Line 649. Dogs were used for toxicity measurement at baseline. Why change models so frequently (and then back to mice)?

*In accordance with the ICH guidelines, we used dogs for the preliminary safety evaluation. As requested by the other Reviewers, the full results of the toxicity studies have been included in the manuscript (**lines 402-418**).*

The authors continued to use a wide variety models to test their peptide, which is good in the sense that it shows various potentially applications, but lack in-depth analysis and consistency. Too many cell, animal and disease models were used in general.

We addressed the translational aspect in accordance with ICH guidelines by using different human cell lines and animals. The disease models reflect ischaemia/reperfusion damage to different organs.

Fig. 5a and 5d. Lack of sham control. Fig. 5d. 'Reduction in cTnI was accompanied by minor mitochondrial swelling' It seems the other way around, i.e. reduced swelling. Fig. 5e. Fragementation not visible. This is how it normally looks like in vivo, and also depends on the specific site in the cell. The authors should provide additional images showing larger areas, as well as rigorous quantification.

*We have included the sham control data relating to Fig. 5a and d in **new Supplementary Fig. 20e** and in **new Fig. 5b (line 454).** Regarding Fig. 5d, we have amended the statement as follows: 'The results demonstrated reduced mitochondrial swelling, accompanied by a significant decrease in serum concentrations of cardiac troponin (cTnI) - a marker of cardiomyocyte necrosis that is utilised clinically for diagnosing myocardial infarction - in the B-017 treated group, compared to the control group (Fig. 6a, b) (**lines 527-530**). The conclusion related to mitochondrial fragmentation, we have removed from the manuscript.*

Finally, on a broader scale, how does B-017 compare to established Bax inhibitors? How does Bax or BNIP3 depletion itself impact I/R?

To date, there are no published data on the efficacy of BAX inhibitors or genetic ablation of BAX in myocardial ischaemia/reperfusion in vivo. The well-established small-molecule allosteric BAX inhibitor BAI1 inhibits the mitochondrial translocation of BAX, but, unlike B-017, it does not affect the early conformational change of BAX (Amgalan et al., 2020, Nat. Cancer). To assess the impact of Bnip3 depletion on I/R injury and to elucidate the relationship between B-017's mechanism and BNIP3/BAX as mentioned by Reviewers 1 and 3, we have conducted myocardial I/R injury in Bnip3 knockout mice. We found that Bnip3 deletion generally protected cardiac tissue from I/R injury, as evidenced by a 51% reduction in infarct size compared to wild-type mice. However, intracardiac treatment of Bnip3 knockout mice with B-017 five minutes before the onset of reperfusion provided no additional protection. This finding supports the proposed relationship. We have added the results and the method to the Results ***(lines 516-524)*** *and Methods sections* ***(line 1118)****. The data are now shown in* ***new Supplementary Fig. 23a*** *of the revised manuscript.*

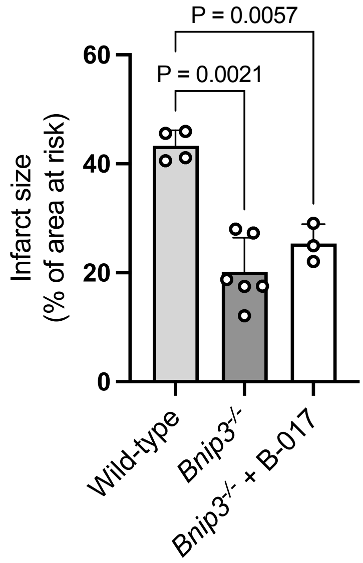

New Supplementary Fig. 23a: *Therapeutic response to the intracardial injection of B-017 in Bnip3 knockout (Bnip3$^{-/-}$) mice, compared with untreated wild-type and Bnip3$^{-/-}$ mice, in ischaemia/reperfusion. Mice were exposed to 30 min of ischaemia, followed by 24 h of reperfusion. Infarct size measurement (infarcted tissue per AAR) showed that Bnip3 deletion generally protected cardiac tissue from I/R injury, as evidenced by a 51% reduction in infarct size compared to wild-type mice. B-017 treatment provided no additional protection (n = 4 C57Bl/6J mice, n = 3-6 Bnip3$^{-/-}$ mice, one-way ANOVA).*

Minor comments.

1. Overall, the figure legends are severely overloaded. Many of the method details and results descriptions in the legends should go in the Methods.

We have carefully reviewed the figure legends and adapted them to align with the guidelines of Nature Communications.

2. Line 144 mBNIP3-His?

We corrected mBNIP3-His in ***line 146****.*

3. Labeling of Extended Data Fig. 5a is not sufficiently clear to the reader. Does IPCr mean IgG control? What do the * in the figure indicate? What antibody was used for IP in each blot?

We apologise for the oversight. We have now clarified the labelling in Supplementary Fig. 5a accordingly.

- *IPCr (IP control) does indeed refer to the IgG control. We have changed this accordingly.*
- *The asterisks (*) indicating the bands corresponding to the protein of interest at the expected molecular weight, as detected by Western blotting, have been deleted.*
- *As indicated in the Methods section, the antibody used for the immunoprecipitation is the anti-FLAG antibody (clone M2; Sigma-Aldrich, #F1804).*

4. Color scale missing in Extended Data Fig. 11b.

We have added the colour scale in Supplementary Fig. 11b.

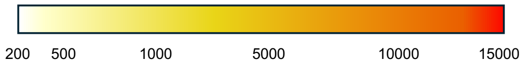

5. Fig. 3a: were proteins normalized to total protein? Repeated-measures ANOVA should be used instead of one-way ANOVA. N=3 (legend) or n=4 (figure)?

The proteins were normalised to total protein, as shown in formerly Extended Data Fig. 14a (now Source Data file). For statistical analyses of the four independent experiments, we used repeated-measures ANOVA and changed ***Fig. 3a*** *accordingly. Regarding the statement on the number of independent experiments, we apologise for the typo and have corrected the figure legend to n = 4.*

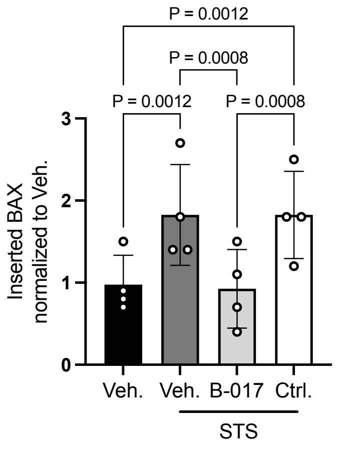

New Fig. 3a: *Effect of B-017 on mitochondrial-induced cell death signalling characterised by BAX insertion into the mitochondrial outer membrane. Wild-type mouse embryonic fibroblasts (MEFs, 3x10^{6} cells) were exposed to staurosporine (2 µM) and B-017 (146 µM), vehicle, or negative control peptide (Ctrl., 146 µM). To assess insertion of BAX, mitochondria were isolated after 4 h of incubation and subjected to alkaline extraction to remove loosely attached BAX. Immunoblot analyses showed that B-017 prevents BAX insertion. (n = 4 independent experiments, repeated-measures ANOVA).*

6. Figure quality needs to be improved in many cases. For example, Fig. 3c left blot, Fig. 5e TEM images, Fig.5a TTC/Evans Blue stains, etc.

Where appropriate, we have improved the quality of the figures accordingly.

7. Line 613. Rationale for using 12 month-old mice? Normally younger mice are used.

*We apologise for the typo and have corrected the age to 12 weeks (**line 428**).*

Reviewer #3 (Remarks to the Author):

The manuscript presents a compelling reverse-engineering approach to develop a mitochondrial protective peptide, B-017, derived from BNIP3. This comprehensive study integrates structural modeling with in vitro, cellular, and in vivo models of ischemia/reperfusion injury. The innovative concept of targeting the BNIP3-BAX/BAK1 axis to simultaneously inhibit apoptosis and necrosis holds significant potential. However, to meet the rigorous standards of Nature Communications, substantial revisions are necessary, particularly concerning data presentation, statistical rigor, mechanistic clarity, and depth of evidence.

We thank Reviewer 3 for all suggestions as they greatly have helped to improve the quality of our work.

Major Concerns:

1. Experiments should be conducted to compare the protective effects of B-017 in wild-type versus Bnip3-/- mice subjected to I/R injury. A significant reduction or abolition of protection in Bnip3-/- mice would confirm BNIP3 as the essential target. If feasible, studies in Bnip3 and Bax double-knockout models (cell lines or mice) are recommended. The loss of B-017's efficacy in these models would provide the strongest evidence for its dependence on the BNIP3-BAX axis.

We thank the Reviewer for this important remark. A similar request was also levelled by Reviewer #1. To further elucidate the relationship between B-017's mechanism and BNIP3/BAX as mentioned by the Reviewer, we have conducted myocardial I/R injury in Bnip3 knockout mice treated with B-017. Bnip3 and Bax double-knockout models are unfortunately not available. We found that Bnip3 deletion generally protected cardiac tissue from I/R injury, as evidenced by a 51% reduction in infarct size compared to wild-type mice. However, intracardiac treatment of Bnip3 knockout mice with B-017 five minutes before the onset of reperfusion provided no additional protection. This finding supports the proposed relationship. We have added the results and the method to the Results ***(lines 516-524)*** *and Methods sections (****lines 1118****). The data are now shown in* ***new Supplementary Fig. 23a*** *of the revised manuscript.*

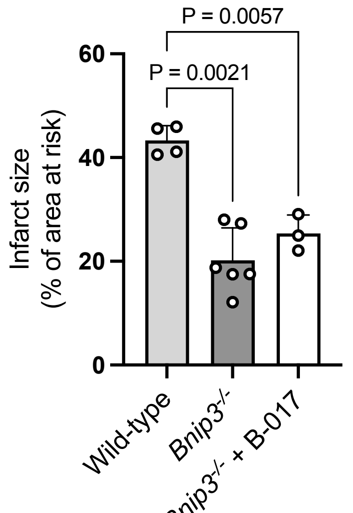

New Supplementary Fig. 23a: *Therapeutic response to the intracardial injection of B-017 in Bnip3 knockout (Bnip3$^{-/-}$) mice, compared with untreated wild-type and Bnip3$^{-/-}$ mice, in ischaemia/reperfusion. Mice were exposed to 30 min of ischaemia, followed by 24 h of reperfusion. Infarct size measurement (infarcted tissue per AAR) showed that Bnip3 deletion generally protected cardiac tissue from I/R injury, as evidenced by a 51% reduction in infarct size compared to wild-type mice. B-017 treatment provided no additional protection (n = 4 C57Bl/6J mice, n = 3-6 Bnip3$^{-/-}$ mice, one-way ANOVA).*

2. All typographical and numerical inconsistencies, especially in figure legends, must be corrected. For instance, the dose of B-017 in Fig. 5a should be reconciled, as it is currently reported variably as 8 mg/kg and 0.8 mg/kg.

We have carefully checked our manuscript and corrected all typographical and numerical inconsistencies.

3. Sham-operated control groups should be included in all in vivo experiments (Fig. 5 and related supplements) to properly establish baseline conditions and injury-specific effects.

We have included the sham-operated control groups in the experiments on mouse myocardial and brain ischaemia/reperfusion and left ventricular function. The data are now shown in ***new Supplementary Figs. 20e and 22a and new Fig. 5b (line 454)****. Regarding the myocardial ischaemia/reperfusion experiments in pigs, we would like to refer in accordance with the 3R principle to published results, that confirm that sham operations have no influence on infarct size (Sun et al., 2010, The Heart Surg Forum).*

4. A schematic diagram should be included to summarize the proposed mechanism of B-017, illustrating its interactions with BNIP3 and BAX/BAK1 and the resulting mitochondrial protection pathway.

We appreciate the Reviewer's suggestion and have conceived schematic diagrams ***in new Supplementary Fig. 25a and b (lines 591, 605)*** *to provide an overview of the proposed mechanism of B-017 and the mitochondrial protection pathway.*

a **b**

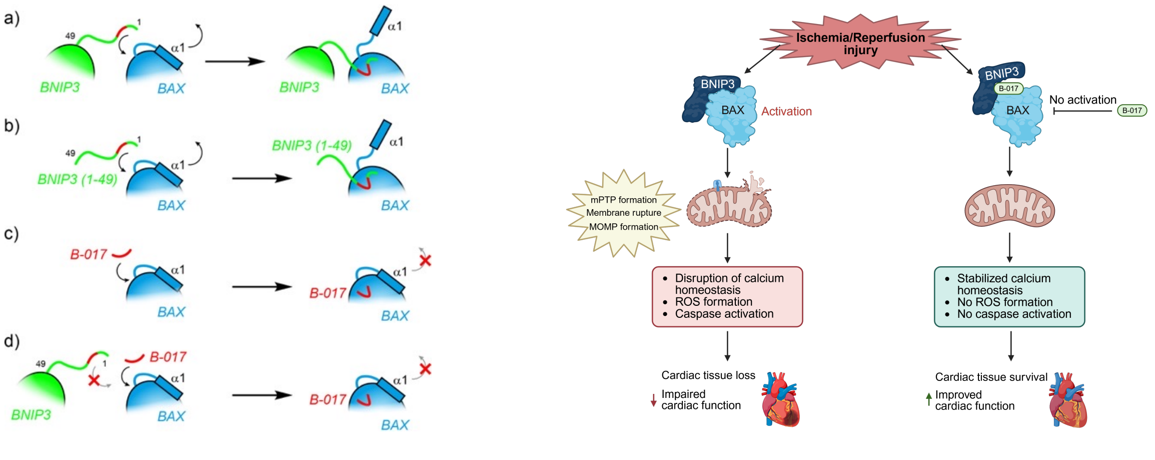

New Supplementary Fig. 25a. *Schematic diagram showing the proposed mechanism of B-017 illustrating* ***(a)*** *the functional domain of full-length BNIP3 activating BAX leading to exposure of helix α1,* ***(b)*** *binding of the functional domain of BNIP3 as BNIP3-peptide$_{1-49}$ activating BAX leading to exposure of helix α1* ***(c)*** *binding of B-017 to BAX with no activation of BAX, and* ***(d)*** *competition of B-017 with the BNIP3 activating domain, preventing the activation of BAX.* ***b****, Schematic diagram showing the impact of B-017 on the mitochondrial protection pathway. BNIP3 plays a crucial role in BAX-induced mitochondrial damage. Direct activation of BAX by BNIP3 leads to the perturbation of the mitochondrial membranes, resulting in mitochondrial outer membrane rupture or pore formation in the mitochondrial outer membrane. These culminates in the initiation of apoptotic and necrotic cell death, which has a dramatic effect on cardiac function. (mPTP, mitochondrial permeability transition pore; MOMP, mitochondrial outer membrane pore).*

5. The metabolomics analysis in Fig. 5i should be strengthened by directly linking specific metabolic alterations—such as changes in cycle intermediates—to the observed improvements in mitochondrial function and protection, thereby providing deeper biological insight.

*Thank you for this suggestion. In the B-017–treated group, the principal TCA cycle intermediates elevated relative to both the control and sham groups were succinate, fumarate, malate, and α-ketoglutarate (a key rate-determining intermediate of the TCA cycle). Consistent with this shift, amino acid metabolism closely linked to TCA cycle function was also modulated by B-017, as reflected by higher levels of proline, serine, and asparagine compared with the non-treated and sham groups. Given the tight coupling between myocardial TCA cycle activity, ATP production, and contractile performance particularly in the setting of post–I/R LV dysfunction these coordinated increases in TCA intermediates and amino acids suggest a compensatory metabolic response that likely contributes to preserved contractile function. This interpretation is further supported by the enhanced ATP generation observed with B-017 treatment. We have added a paragraph to the Results section of the revised manuscript to clarify these points (**lines 546-547, 549-560**).*

6. All figures must be re-exported at high resolution with complete and clear labels. For example, the incomplete y-axis label in Fig. 2e should be corrected, and the clarity of Fig. 2f should be enhanced.

According to the Reviewer's remark, we have re-exported the figures at a high resolution, complete with clear and comprehensive labels.

7. The specificity of B-017 should be assessed by evaluating potential off-target interactions with other BCL-2 family proteins (e.g., BCL-2, MCL-1) using binding assays such as SPR or ITC.

*We thank the Reviewer for these important points. To address specificity, we performed off-target analyses using microscale thermophoresis and fluorescence anisotropy. First, we titrated fluorescence-labelled B-017 with increasing concentrations of the anti-apoptotic BCL-2 family members human (h) MCL-1 and hBCL-2, which inhibit the oligomerisation required for the formation of the MOMP by activated and already inserted BAX. With K_D values of approximately 1.3 µM for hMCL-1 and hBCL-2, B-017 bound these proteins with a 4-fold lower affinity than it bound hBAX (K_D = 303 nM). This residual binding is likely attributable to the general propensity of peptides to interact with proteins that share relatively high structural similarity, which is the case for members within the BCL-2 protein family (London et al., 2010, Structure). For example, BCL-2 and BAX display a root mean square deviation (RMSD) of approximately 1.9 Å to 2.2 Å (Petros et al., 2001, PNAS), indicating that they have similar structures. On the other hand, and in support of this interpretation, no binding of B-017 was detected to the structurally unrelated protein glutathione-S-transferase. Together, this points to an unlikely occurrence of side effects due to off-target responses. We have added these new results and the respective methods to the Results (**lines 387-401**) and Methods sections (**lines 853-854, 866-881**), and text to the Abstract (**lines 56, 57, 59**). The data are now shown in **new Supplementary Fig. 18** of the revised manuscript. Since all MST and fluorescence anisotropy measurements were performed with tagged recombinant proteins, except the anisotropic evaluation of B-017 binding to BAX, we have replaced Fig 2f with the results achieved with tagged BAX for unification (**new Figure 2f, right panel**). The comparability of the two methods used is demonstrated by the identical K_D values obtained for BCL-2 (**new Supplementary Fig. 18b**).*

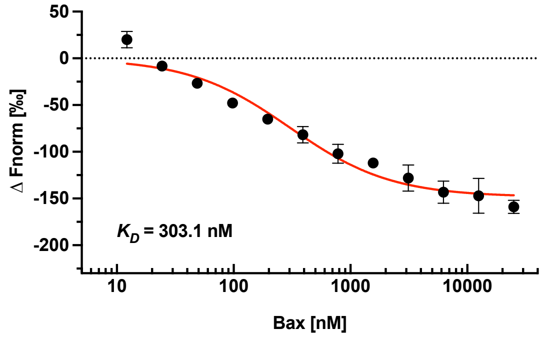

New Fig. 2f: *Microscale thermophoresis dose-response curve of the binding of the protein BAX to Cy.5.5-labelled peptide. A constant concentration of 200 nM fluorescence-labeled peptide was incubated with serial dilutions of BAX (12.2–25,000 nM). Normalised fluorescence changes were plotted as a function of protein concentration. To determine binding affinities (K_D), nonlinear regression analysis was performed using a one-site binding model. Data points represent the mean ± SD from three biological replicates (n = 3), and solid red lines show the fitted binding curves.*

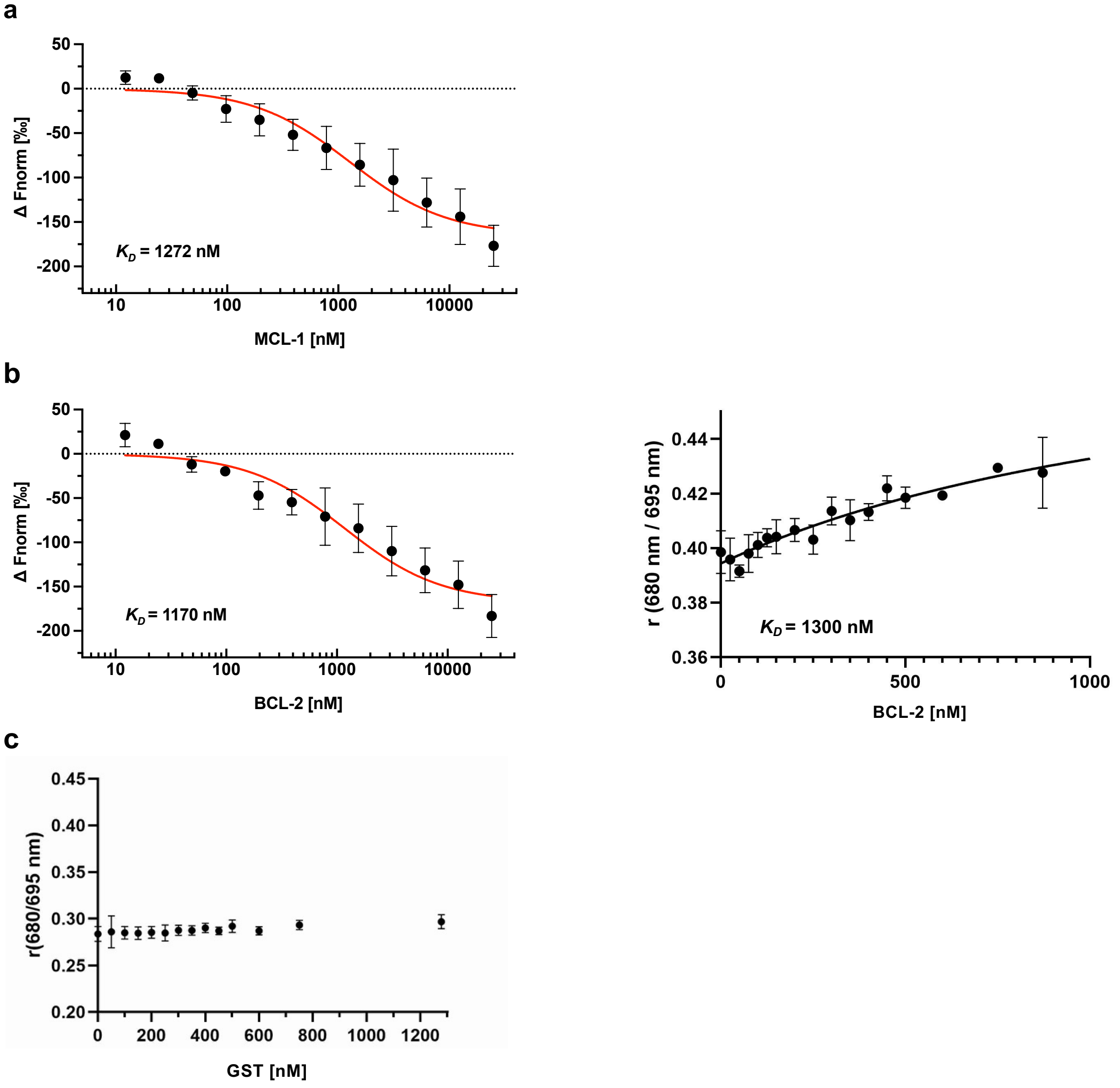

New Supplementary Fig. 18. Dose-response curves of the binding of the proteins MCL-1, BCL-2, and glutathione-S-transferase to Cy5.5-labelled peptide.

*Microscale thermophoresis dose-response curves for the binding of Cy5.5-labelled B-017 to the human proteins **(a)** MCL-1 and **(b, left)** BCL-2. A constant concentration of 200 nM fluorescence-labelled B-017 was incubated with serial dilutions of each protein (12.2–25,000 nM). Normalised fluorescence changes were plotted as a*

function of protein concentration. To determine binding affinities (K_D), nonlinear regression analysis was performed using a one-site binding model. Data points represent the mean ± SD from three biological replicates (n = 3), and solid red lines show the fitted binding curves. ***b, c,*** *Fluorescence anisotropy measurement (680/695 nm) with titration of increasing human BCL-2 (**b, right**) or glutathione-S-transferase (GST) concentrations (**c**) to 100 nM Cy5.5-labelled B-017. All titrations were performed in n = 3 technical replicates and data points are shown as means ± SD based on the three experiments (n = 3). Binding curves of the averaged data were fitted with GraphPad Prism 10.0 (GraphPad) using the quadratic binding equation for a one-site specific binding model and K_D values are given as fit of the averaged data points ± standard deviation of the fit.*

8. A comprehensive safety profile is required, including an evaluation of organ toxicity, immune responses, and key pharmacokinetic parameters (e.g., plasma stability, tissue distribution, clearance, and half-life) to robustly support the therapeutic potential of B-017.

Thank you for this suggestion, which was also made by another Reviewer. To follow your advice and to address the safety of B-017 as a key criterion of a therapeutical agent, we recorded animal body weights and performed toxicity analysis including histological observation of tissue and organs after a 2-week intravenous injection of B-017 at dosages of 3, 6, and 12 mg kg^{-1} day^{-1} followed by a two-week recovery period in female and male rats. The study was performed by WuXi AppTec Co., Ltd, and was conducted in compliance with Good Laboratory Practice standards specified by ICH guidelines.

*Overall, the administration of B-017 to female and male Wistar Han rats once daily for 2 weeks by intravenous injection at all three dosages was well tolerated. The rats showed no body weight loss during the dosing or recovery phases of the B-017 treatment. B-017-related change in body weight was limited to lower body weight gain in male rats at a dosage of 12 mg/kg/day during the dosing phase, compared to the concurrent control group. There were significant differences in body weight gain from day 4 to 14 (day 4 to day 7: -48.4%; days 11 to 14: -48.9%). This resulted in a slightly reduced mean body weight in male rats. However, at the end of the recovery phase, the above changes balanced out and all body weight values in males at a dose of 12 mg kg^{-1} day^{-1} were comparable to the concurrent control data. Other fluctuations in body weight gain were consistent with those observed in untreated rats. The data are now shown in **new Supplementary Fig. 19**.*

*In addition, no B-017-related changes were detected in serum-chemistry and urine analysis parameters, and no visible lesions were observed in examined various tissues and organs, including the heart, liver, and brain were observed in either sex (**Supplementary Tables 5-10**). During the dosing phase, microscopic changes were identified as subcutaneous haemorrhage, subcutaneous inflammation of mixed cells, and/or cutaneous/subcutaneous/muscular necrosis in the injection site in rats. Minimal or mild subcutaneous haemorrhage was noted in perivascular areas of 5 high-dose animals (2 males and 3 females), one mid-dose male, and none in the low-dose and control groups, an indication of B-017-related injection site change. Minimal to marked subcutaneous inflammation of mixed cell with or without necrosis was identified in the cutaneous, subcutaneous, adjacent muscular fibers, and/or surround the vein. The incidence and severity were dose dependent. In addition, the necrosis and/or necrotizing inflammation in the injection site were exclusively observed in animals of the high-dose groups. Compared with the incidence in the control group, the increased incidence and severity of lesions in the injection site were likely B-017 related. At the end of a 2-week recovery period, B-017-related changes were only present in the injection site of one single high-dose animal. This rat had minimal subcutaneous haemorrhage and moderate necrotizing inflammation of mixed cells in the cutaneous, subcutaneous and adjacent muscle. No lesions in the injection site were noted in the high-dose females, an indication of a full recovery in female rats. The data are now shown in **new Supplementary Tables 9 and 10**.*

B-017-related changes in haematology were limited to male rats at 12 mg kg^{-1} day^{-1} at the end of the dosing phase with increased WBC (77.70%, $p \leq 0.001$), neutrophils count (208.67%, $p \leq 0.01$) and neutrophils percentage (65.5%) compared with concurrent control data. These haematological changes were correlated to microscopic changes of subcutaneous haemorrhage, subcutaneous inflammation of mixed cells, and/or cutaneous/subcutaneous/muscular necrosis in the injection site. However, all

haematological changes had resolved at the end of recovery phase. The data are now shown in ***new Supplementary Tables 11 and 12****.*

Regarding the toxicokinetics of B-017, initial and maximum plasma concentration (C_0, C_{max}), time to reach C_{max} (T_{max}), $T_{1/2}$ and area under the plasma concentration-time curve from time zero to 24h-hour postdose (AUC_{0-24h}) were calculated. After single (Day 1) or repeated (Day 14) intravenously injection of B-017 to male and female rats, T_{max} values for B-017 were observed at 0.1 h postdose. $T_{1/2}$ values for B-017 were ranged from 0.3 to 0.6 hour. No marked sex difference in systemic exposure (AUC_{0-24h} and C_0) to B-017 was observed at all dose levels. As the dosage increased from 3 to 12 mg/kg/day, the systemic exposure (AUC_{0-24h} and/or C_0) to B-017 increased dose-proportionally in both sexes on Day 1 and Day 14. The data are now shown ***in new Supplementary Table 13****.*

In conclusion, the No Observed Adverse Effect Level (NOAEL) for rats is 12 mg kg^{-1} day^{-1}. The corresponding AUC_{0-24h} and C_0 of B-017 on Day 14 at NOAEL were 4,210 h ng mL^{-1} and 9,890 ng ml^{-1} for males, and 4,950 h ng mL^{-1} and 10,700 ng mL^{-1} for females, respectively.

We have added the results as follows ***(lines 402-418)****:*

"B-017 did not cause toxicity as initially inferred by monitoring the body weight of female and male rats administered 0, 3, 6 and 12 mg kg^{-1} body weight for 14 days, followed by a 14-day recovery phase. The rats showed no body weight loss during the dosing or recovery phases of the B-017 treatment (Supplementary Fig. 19a). In addition, no B-017-related changes were detected in serum-chemistry and urine analysis parameters, and no visible lesions were observed in examined various tissues and organs, including the heart, liver, or brain, in either sex (Supplementary Tables 5-10). During the dosing phase, treatment-related transient microscopic findings were confined to the injection site and included subcutaneous haemorrhage mixed-cells inflammation, and necrosis of the skin, subcutaneous tissue and underlying muscle in both female and male rats (Supplementary Tables 9 and 10). These local findings were accompanied by haematological changes, but these remained transient (Supplementary Tables 11 and 12). All these observations had fully resolved by the end of the recovery phase. Systemic exposure to B-017 increased proportionally with dose, with no sex-related differences observed and a terminal half-life ranging from 0.3 to 0.6 h (Supplementary Table 13). Taken together, the demonstrated appreciable target specificity and favourable safety profile support the suitability of B-017 as a potential therapeutic agent."

The results and the methods have been added to the Results ***(lines 402-418****) and Methods section* ***(lines 658-668, 1218-1249****) and text to the Abstract* ***(lines 56, 57, 59****) and Discussion sections* ***(line 607****) in the revised manuscript.*

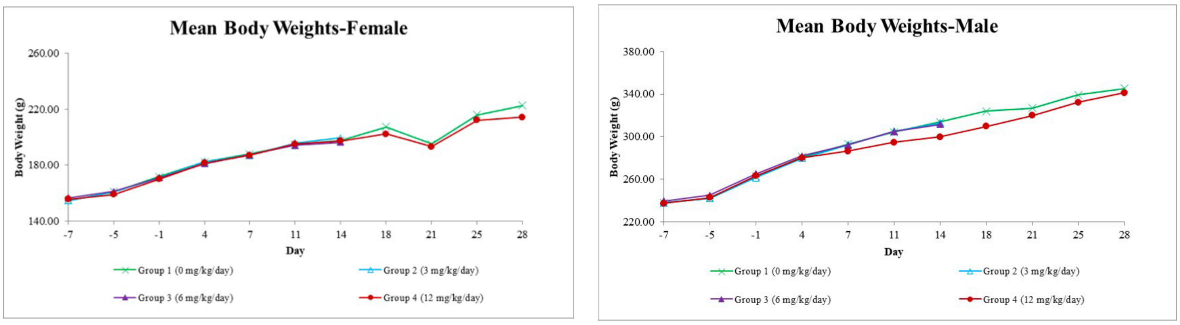

Supplementary Fig. 19. Impact of B-017 on body weight in female and male rats. *The animals received four different doses of B-017 (0, 3, 6 or 12 mg kg^{-1} body weight) for 14 days, followed by a 14-day recovery phase. Neither sex showed any loss of body weight during the dosing or recovery phases of the B-017 treatment.*

9. Testing B-017 in chronic disease models involving BNIP3/BAX dysregulation (e.g., chronic heart failure, neurodegeneration, or NASH) is recommended to explore its potential for long-term efficacy.

*Following up on the Reviewer's suggestion, we evaluate the long-term efficacy of B-017 exclusively on the development of heart failure. The mice were exposed to 50 min of ischaemia following by a reperfusion duration up to 28 days. The mice received either B-017 or the negative control peptide intraperitoneally only after the onset of I/R on day 1, day 3, day 5 and day 7. This approach showed that the group treated with B-017 experienced an improvement in LVEF by day 3, with recovery by day 28. In contrast, the control group showed no change in LVEF from day 1 to day 28. We have added the results, the methods and text to the Results (**lines 486-491**), Methods (**lines 1208-1210**), and Discussion section (**line 595**). The data are now shown in **new Fig. 5f** of the revised manuscript.*

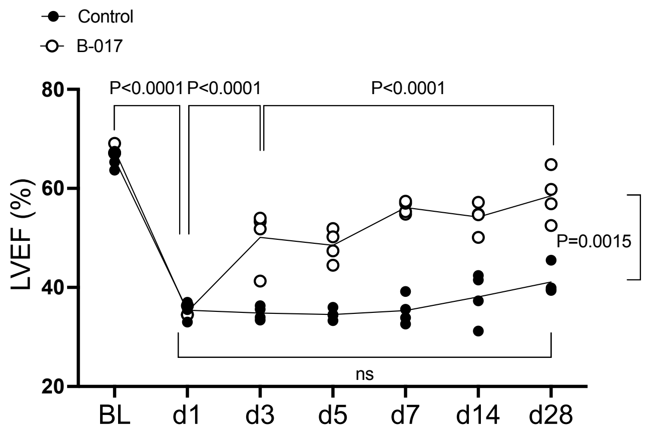

***New Fig. 5f:** Therapeutic response to B-017 in the development of heart failure. Mice were subjected to 50 min of ischaemia and 28 days of reperfusion. They were injected intraperitoneally with either negative control peptide (Ctrl.,) or B-017 (0.8 mg kg^{-1} BW, each) on d1, d3, d5, and d7 after the onset of reperfusion. Changes in LVEF from d1 to d28 were calculated using the Simpson's method. (n = 4 C57BL/6J mice per group). Two-way ANOVA.*

10. High-resolution respirometry should be performed to quantitatively assess mitochondrial oxygen consumption rates in cells or tissues treated with B-017 following I/R injury. This direct functional assessment would strongly support the conclusion of mitochondrial protection.

*We thank the Reviewer for the valuable suggestion. Although this approach was not applied, mitochondrial function was evaluated by measuring tissue ATP levels as a functional indicator of cellular energy production in the area at risk of mice exposed to 30 min of ischaemia followed by 30 min of reperfusion. The mice were treated intracardially 5 min before reperfusion, either with the negative control peptide (Ctrl.) or with B-017. Sham-operated mice served as controls. Under B-017 treatment, the ATP production was significantly enhanced. Given the development of left ventricular dysfunction following I/R injury, the enhanced ATP generation under B-017 treatment may reflect a compensatory metabolic response that contributes to preserved contractile function. This is supported by the observed elevation of TCA cycle intermediates and amino acid levels under B-017 treatment. We have added the results and the method to the Results (**lines 549-560**) and Methods (**lines 1316-1318**) sections. The data are now shown in **new Fig. 6h** of the revised manuscript.*

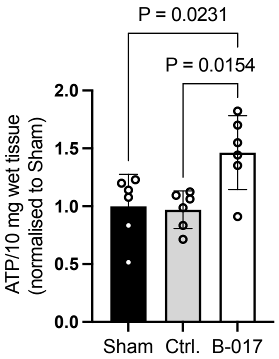

New Fig. 6h: *ATP level in the area at risk of mice exposed to 30 min of ischaemia followed by 30 min of reperfusion. The mice were treated intracardially 5 min before reperfusion either with the negative control peptide (Ctrl., 0.8 mg kg^{-1}BW) or B-017 (0.8 mg kg^{-1}BW). Sham operated mice served as controls. (n=6 C57Bl/6J mice per group).*

References cited in the point-by-point response

1. Amgalan, D. *et al.* A small-molecule allosteric inhibitor of BAX protects against doxorubicin-induced cardiomyopathy. *Nat Cancer* **1**, 315-328 (2020).

2. Chen, H.C. *et al.* An interconnected hierarchical model of cell death regulation by the BCL-2 family. *Nat Cell Biol* **17**, 1270-1281 (2015).

3. Dörner, A. *et al.* Mitochondria isolated from hearts subjected to ischemia/reperfusion benefit from adenine nucleotide translocase 1 overexpression. *Membranes* **11**, 836 (2021).

4. Ke, N. *et al.* The xCELLigence system for real-time and label-free monitoring of cell viability. *Methods Mol Biol* **740**, 33–43 (2011).

5. Kubli, D.A. *et al.* BNIP3 functions as a mitochondrial sensor of oxidative stress during myocardial ischemia and reperfusion. *Am J Physiol Heart Circ Physiol* **295**, H2025-H2031 (2008).

6. London, N. *et al.* The structural basis of peptide-protein binding strategies. *Structure* **18**, 188-199 (2010).

7. Liu, K.E. & Frazier W.A. Phosphorylation of the BNIP3 C-Terminus inhibits mitochondrial damage and cell death without blocking autophagy. *PlosOne* **10**:e0129667(2015).

8. Petros, A.M. *et al.* Solution structure of the antiapoptotic protein BCL-2. *Proc Natl Acad Sci U S A* **98**, 3012-3017 (2001).

9. Sun, H. *et al.* Ischemic postconditioning inhibits apoptosis after acute myocardial infarction in pigs. *The Heart Surg Forum* **13**, E305-310 (2010).

10. Vitale, I. *et al.* Apoptotic cell death in disease – Current understanding of the NCCD 1023. *Cell Death Differ* **30**, 1097-1154 (2023).

11. Zhu, Y. *et al.,* Modulation of serines 17 and 24 in the LC3-interacting region of BNIP3 determines pro-survival mitophagy versus apoptosis. *J Biol. Chem* **288**,1099-1113 (2013).

Point-by-point response to Reviewers related to the revised manuscript NCOMMS-25-61408A

Manuscript Title: **Reverse engineering of BNIP3 identifies a mitochondrial protective peptide by Hendgen-Cotta et al.**

Reviewer' Comments & Author Responses

<u>Reviewers' Comments:</u>

We thank the Reviewers for their thorough examination of the revised manuscript and the constructive remarks. Below, you will find a point-by-point reply to each of the Reviewers' comments, with comments shown in **bold**.

Reviewer #1 (Remarks to the Author):

The authors have addressed my previous concerns thoroughly with substantial new data. I appreciate their efforts. However, a few minor issues remain:

We thank the Reviewer for their positive feedback.

1. Supplementary Fig. 22a (neurological function): The data presentation is unclear. Please specify what the data points represent, add statistical information, and present as a scatter plot with median.

We have now presented the results as a scatter dot plot with median with interquartile range and p value and included the Bederson score sheet in new ***Supplementary Fig. 22a****.*

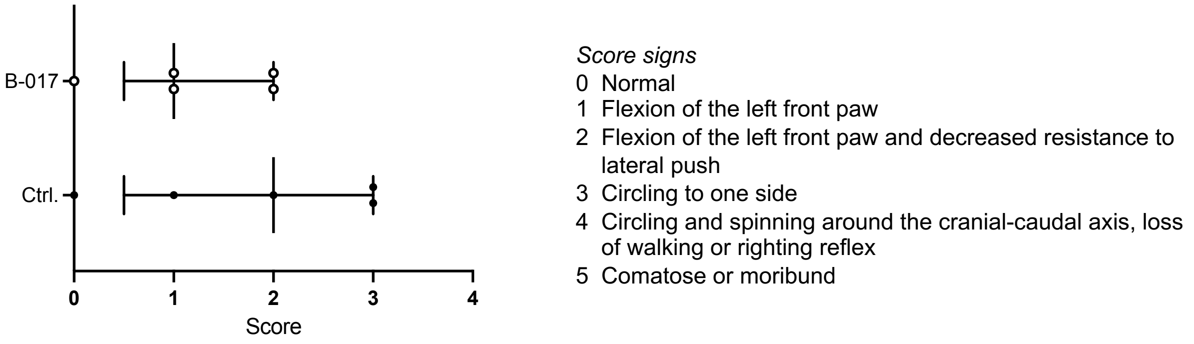

Supplementary Fig. 22a*: The Bederson Score assessment 24 hours postoperative in control group and B-017 treatment group tended to show fewer neurological deficits under B-017 treatment. (n = 5 C57BL/6J male mice per group, Student's t-test, P = 0.4112).*

2. Supplementary Fig. 23c (mitochondrial cytochrome c): There is a discrepancy: legend says lanes 3-5 are I/R, but blot labels them as sham. Please clarify. The representative blot images mentioned in the rebuttal are not included in the revised Supplementary Figures. Please provide them alongside the cytosolic data. In addition, the mitochondrial cytochrome c blot lacks a mitochondrial loading control (e.g., VDAC1/COX IV) to verify equal loading across lanes.

We apologise for the incorrect description of the lanes 3-5 in the legend. As indicated by the label on the blot, lanes 3-5 represent the sham operated group. The blot images referred to in the rebuttal have

been included in the revised Supplementary Information, alongside the blot showing the loading control ANT1.

3. The ANT1 Western blot images mentioned in the rebuttal are also missing from the Supplementary figures. Please include them.

We have included the mentioned ANT1 Western blot images related to the cytosolic cytochrome c data in the Source Data file.

4. Fig. 6a (mitochondrial swelling): The figure shows that the Ctrl peptide group has lower mitochondrial swelling, while the B-017 group shows higher swelling. This appears to contradict the statement that B-017 prevents swelling. Please clarify this inconsistency.

*To obtain the degree of mitochondrial swelling due to osmotic influx, we measured the optical mitochondrial density. A higher value of density therefore indicates less swelling. We added a clarifying sentence to the legend (**line 1858**): **'Higher optical density indicates less swelling.'***

I have reviewed the authors' response to Reviewer 3.

We thank Reviewer #1 for evaluating our response to the suggestions from Reviewer #3 in the revised manuscript.

Overall, the authors have made substantial efforts and added considerable new data. I appreciate their work.

We thank the Reviewer for their positive feedback.

However, a few issues remain that should be addressed or acknowledged:

1. Mechanism diagram (Supplementary Fig. 25b) is incomplete. It only depicts the heart, but the study also includes brain ischemia and liver ischemia models. Please expand the diagram accordingly.

Thank you for bringing this up. We have now expanded the diagram to brain and liver.

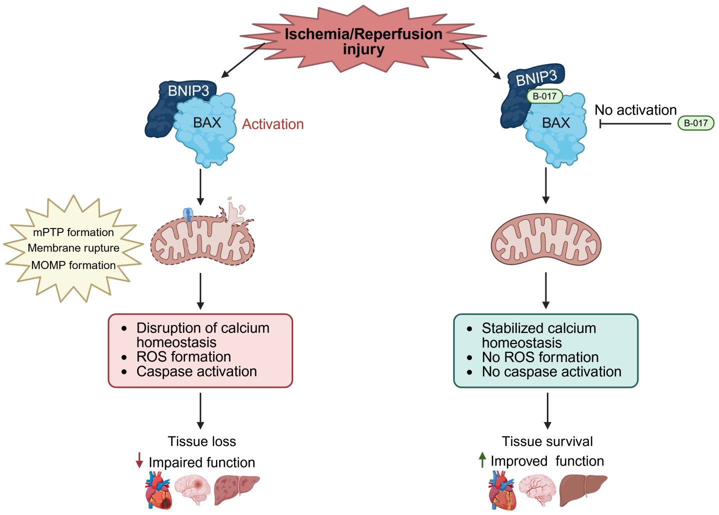

***Supplementary Fig. 25. Mode of action of B-017. b**, Schematic diagram showing the impact of B-017 on the mitochondrial protection pathway. BNIP3 plays a crucial role in BAX-induced mitochondrial damage. Direct activation of BAX by BNIP3 leads to the perturbation of the mitochondrial membranes, resulting in mitochondrial outer membrane rupture or pore formation in the mitochondrial outer membrane. These culminates in the initiation of apoptotic and necrotic cell death, which has a dramatic effect on cardiac function. (mPTP, mitochondrial permeability transition pore; MOMP, mitochondrial outer membrane pore). Created in BioRender. Roth, A. (2026). BioRender.com/XE29MK0Y32*

2. Sham controls are still missing. Especially key figures including Fig. 5b, 5d/e, and particularly Fig. 5f lack sham-operated controls. Without a sham group, it is difficult to assess baseline function or the degree of recovery.

The sham controls related to Fig. 5b are already included in Supplementary Fig. 20e, alongside the therapeutic response to various control treatments and B-017. The sham controls related to left ventricular function were included as an insert in Fig. 5d. With regard to Fig. 5f, we would like to refer to the sham controls depicted in Fig. 5d. We acknowledge that sham-operated controls for the strain analyses are not included due to technical limitations.

3. The chronic heart failure study (Fig. 5f) is underpowered. Only LVEF is shown without other cardiac function indices. Besides, standard endpoints such as histological assessment of fibrosis (e.g., Masson's trichrome) and heart weight/tibia length ratio are not provided.

If these data are not available, the authors should acknowledge these as limitations.

*We agree with the Reviewer that the assessment of the LVEF is only an initial indication that B-017 may be beneficial in limiting chronic heart failure, and we have acknowledged this in the Results section of the revised manuscript (**lines 487-488**): **'This is an initial indication that B-017 may also be beneficial in limiting chronic heart failure.'***

Reviewer #2 (Remarks to the Author):

The authors have addressed most of my concerns. The manuscript has much improved in terms of clarity.

We would like to express our gratitude to the Reviewer for their positive statement.
